# Tracing the COVID-19 spread pattern in India through a GIS-based spatio-temporal analysis of interconnected clusters

**DOI:** 10.1038/s41598-023-50933-4

**Published:** 2024-01-08

**Authors:** Mousumi Gupta, Arpan Sharma, Dhruva Kumar Sharma, Madhab Nirola, Prasanna Dhungel, Ashok Patel, Harpreet Singh, Amlan Gupta

**Affiliations:** 1 Department of Computer Applications, Sikkim Manipal Institute of Technology, Sikkim Manipal University, Majitar, 737136 India; 2 Department of Pharmacology, Sikkim Manipal Institute of Medical Sciences, Sikkim Manipal University, Tadong Campus, Gangtok, 737102 India; 3 Kusuma School of Biological Sciences, Indian Institute of Technology, Delhi, 110016 India; 4 Division of Biomedical Informatics, Indian Council of Medical Research, Delhi, 110029 India; 5 Department of Transfusion Medicine, Jay Prabha Medanta Super Speciality Hospital, Patna, 800020 India

**Keywords:** Medical research, Signs and symptoms

## Abstract

Spatiotemporal analysis is a critical tool for understanding COVID-19 spread. This study examines the pattern of spatial distribution of COVID-19 cases across India, based on data provided by the Indian Council of Medical Research (ICMR). The research investigates temporal patterns during the first, second, and third waves in India for an informed policy response in case of any present or future pandemics. Given the colossal size of the dataset encompassing the entire nation’s data during the pandemic, a time-bound convenience sampling approach was employed. This approach was carefully designed to ensure a representative sample from advancing timeframes to observe time-based patterns in data. Data were captured from March 2020 to December 2022, with a 5-day interval considered for downloading the data. We employ robust spatial analysis techniques, including the Moran’s I index for spatial correlation assessment and the Getis Ord Gi* statistic for cluster identification. It was observed that positive COVID-19 cases in India showed a positive auto-correlation from May 2020 till December 2022. Moran’s I index values ranged from 0.11 to 0.39. It signifies a strong trend over the last 3 years with $r^2$ of 0.74 on order 3 polynomial regression. It is expected that high-risk zones can have a higher number of cases in future COVID-19 waves. Monthly clusters of positive cases were mapped through ArcGIS software. Through cluster maps, high-risk zones were identified namely Kerala, Maharashtra, New Delhi, Tamil Nadu, and Gujarat. The observation is: high-risk zones mostly fall near coastal areas and hotter climatic zones, contrary to the cold Himalayan region with Montanne climate zone. Our aggregate analysis of 3 years of COVID-19 cases suggests significant patterns of interconnectedness between the Indian Railway network, climatic zones, and geographical location with COVID-19 spread. This study thereby underscores the vital role of spatiotemporal analysis in predicting and managing future COVID-19 waves as well as future pandemics for an informed policy response.

## Introduction

The SARS-CoV-2 virus outbreak rapidly spread across the globe in late 2019, causing the COVID-19 pandemic. India was hardest-hit in terms of both the number of cases and fatalities. Despite the widespread impact of COVID-19 in India, the pattern of occurrence of its waves has been a subject of debate among researchers. Various research studies have been conducted with the aim of bringing valuable insights about the COVID-19 outbreak in India^1–5^. On a general consideration, academic research has always been useful for developing a policy response that may have helped the Government in reducing the impact of disease transmission. A variety of measures were implemented by the government of India to control the spread of the virus and mitigate its impact. These measures included the nationwide lockdown in March 2020, followed by a phased reopening of the economy, and the launch of several vaccination drives^6^. At the time of preparation of this manuscript, future preparedness of policy response is likely to be certain because of availability of various academic research.

The spatiotemporal approach has provided valuable insights into the occurrence pattern of COVID-19^3,7,8^. In 2020, a study analyzed the spatiotemporal pattern of the spread of coronavirus in India during the period from January 30th to June 20th, 2020^3^. This study included an evaluation of spatial clustering, identification of hotspots, spatial heterogeneity and homogeneity, spatial trends, and the direction of COVID-19 cases through the application of spatial statistical analysis. The authors observed that during the study period there existed 17 epicenters in India. Another study used data from 30th January to 21st March 2020 to understand the pattern of occurrence of COVID-19 in India^8^. Their analysis was focused on understanding spatial patterns of disease clustering using global spatial autocorrelation techniques. Further, local spatial autocorrelation was also observed using Getis-Ord Gi* statistics. Authors observed that disease clusters existed positively. Clusters were mainly concentrated in the central and western regions of India. It was also observed that the northeastern part of India had a low rate of clusters.

A study further attempted to analyze the pattern of clustering using Moran’s I index^7^. Authors used data from April 2020 to January 2021. It was observed that in India, Moran’s I index value was greater than 0.10 for temporal data for all months. This is indicative of positive auto correlation, meaning there is a clustering pattern. A positive autocorrelation, as a general rule, indicates that more COVID-19 cases will likely be found in the vicinity of an original hotspot. In another study, Moran’s I index was used to understand COVID-19 deaths and their relation with Normalised Differential Vegetation Index (NDVI) Values^9^. The authors used temporal data till the peak of the second COVID-19 wave in India. It was observed that the NDVI values were positively correlated with death. This is indicative of a negative relationship between greenness and COVID-19-related deaths. It means higher greenness is likely to contribute to a lesser number of COVID-19-related deaths.

Several case studies utilizing spatial analysis have provided valuable insights into COVID-19 spread globally. A study on spatial analysis of COVID-19 clusters in New York City identified areas like eastern Brooklyn with low testing rates but high positive test proportions, suggesting inadequate testing and high case burden in socioeconomically disadvantaged neighborhoods^10^. These clusters were associated with marginalized populations lacking health insurance and reliant on public transportation. The study recommended directing testing and healthcare resources to underserved hotspots.

Another study analyzed the nexus between population density and COVID-19 cases across south Indian states^11^. Using Pearson’s correlation and response surface methodology, the research found strong positive correlations between density and infections in Tamil Nadu, Kerala, Karnataka and Telangana. This highlights population density as a key explanatory factor for COVID-19 transmission in these states. However, Andhra Pradesh showed a more complex dynamic, indicating density alone does not determine contagion.

Research on the spatiotemporal pattern of COVID-19 spread in Brazil revealed rapid, extensive propagation across municipalities^12^. Cluster analysis showed deaths were faster which could be attributed to surveillance limitations. Trajectories of geographic centers indicated spread from São Paulo toward northern states. Despite Brazil’s public health system, ineffective and inequitable responses enabled transmission, resulting in severe outcomes among vulnerable populations.

From these studies, it can be understood that spatial statistics can play a key role in understanding insights into COVID-19’s spread. However, the major problem is data access and the requirement for a system capable of handling a huge number of datasets. In India itself, until the time of preparation of the present manuscript, amount of the total number of data i.e., COVID-19 testing is 917,395,199 with multiple variables (Homepage, Indian Council of Medical Research, https://www.icmr.gov.in/). To overcome computational constraints posed by the massive national dataset, we employed structured time-bound convenience sampling to capture representative snapshots of the advancing pandemic. This approach efficiently analyzed temporal and spatial trends from March 2020 to December 2022 while balancing spatial statistics validity and data size. Convenience sampling enabled insightful analysis despite data access limitations.

This study presents a novel approach by utilizing time-bound convenience sampling to expedite the identification of spatial patterns in the COVID-19 disease spread. It makes important contributions to knowledge of spatiotemporal disease analysis and pandemic management. Comprehensively analyzing nationwide COVID-19 patterns over three critical years, this research overcomes big data constraints retaining spatio-temporal validity for robust insights. The multi-year geospatial analysis provides vital evidence on infectious disease dynamics to inform public health policymaking for mitigating future outbreaks in India and other large countries. The objective for selecting a subset of data from the whole dataset is to avoid time-consuming analysis. By differentiating itself from existing studies in India, it offers academically significant insights into the non-random nature of disease spread, thus contributing to a deeper understanding of the epidemiology and aiding in the development of targeted intervention strategies. The granular analysis of COVID-19 diffusion across local climate and transit factors reveals targeted insights within India’s unique geography. Statistical identification of intensifying hotspots facilitates localized response alignment. This integrated methodology strengthens outbreak investigation, extending global models with actionable district-level intelligence for strategic pandemic management.

## Results

### Clustering pattern of positive cases in India

**Figure 1:**
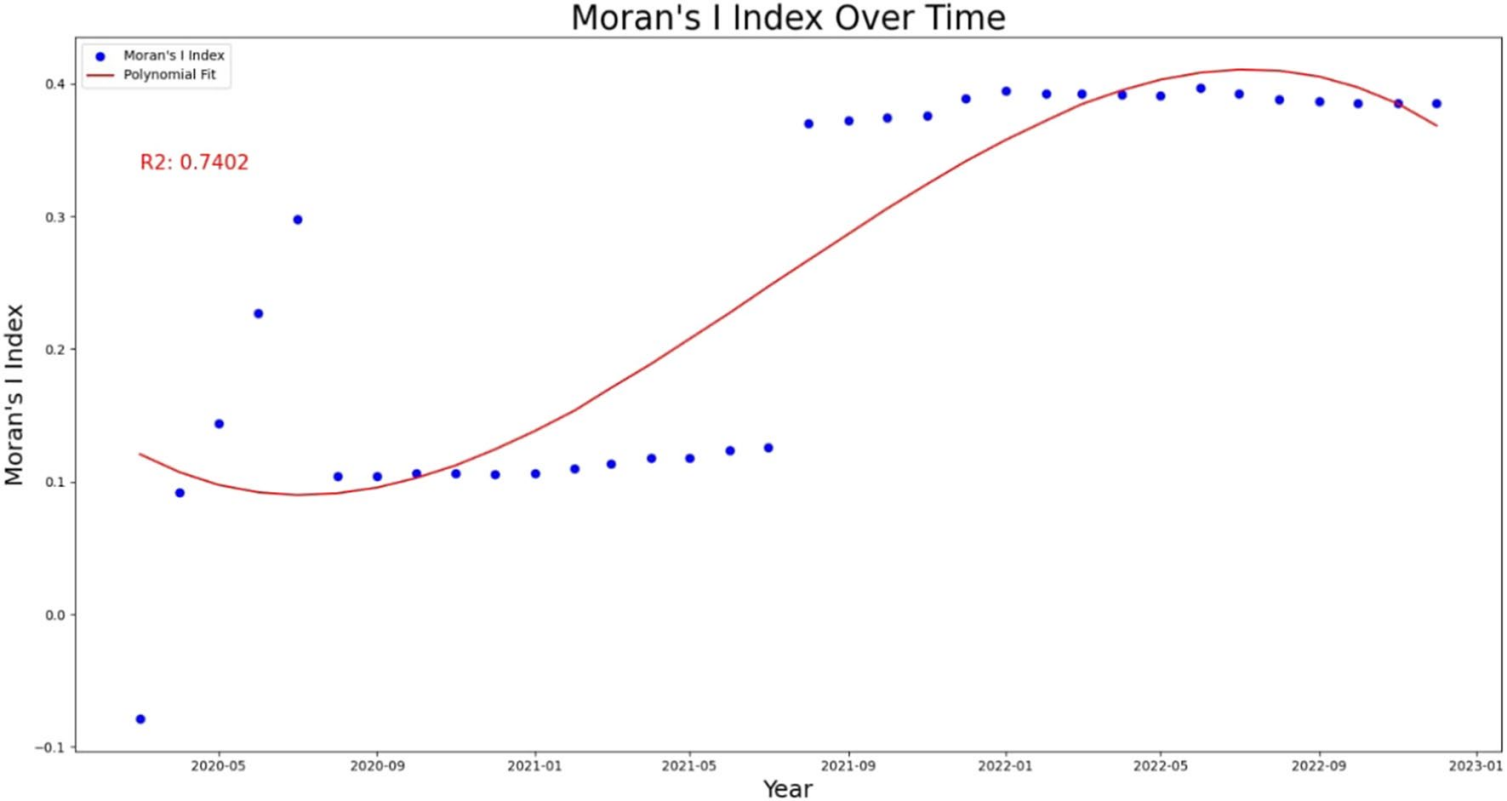
Moran’s I index trend over time.

COVID-19-positive cases in India showed a positive auto-correlation from May 2020 till date. Similarly, the null hypothesis that assumed the pattern of COVID-19 disease is random remained rejected since the same month as the evaluated p-value was found zero for all the months after this point. Moran’s, I index values ranged from 0.11 to 0.39. 3rd-order polynomial regression showed a strong trend in Moran’s I index value over the last 3 years. $r^2$ was observed to be 0.74 which shows that the spread of COVID-19 in India was not a chance of random Event. A similar study in Malaysia showed Moran’s I index value of 0.43^13^. The monthly global Moran’s Index estimation is presented in Supplementary Folder 1, supplementary files, and folders are available on the following link 10.5281/zenodo.7981043 with an open access. The correlation of Moran’s I index over monthly data is presented in Fig. 1.

### Interconnectedness of COVID-19 cases in railways network

It has been found that the spread of COVID-19 is mainly because of close contact and travel^14^. In India, the most preferred way of transport is by Railway. In the year 2022, approximately 8.6 billion passengers traveled through Railways. It is comparatively higher than other preferred networks like roadways and airways. While acknowledging the fact that railways experienced periods of low operations or temporary shutdowns during the initial phase of the pandemic, we attempt to understand how it impacted the overall spread in 3 years.

**Figure 2:**
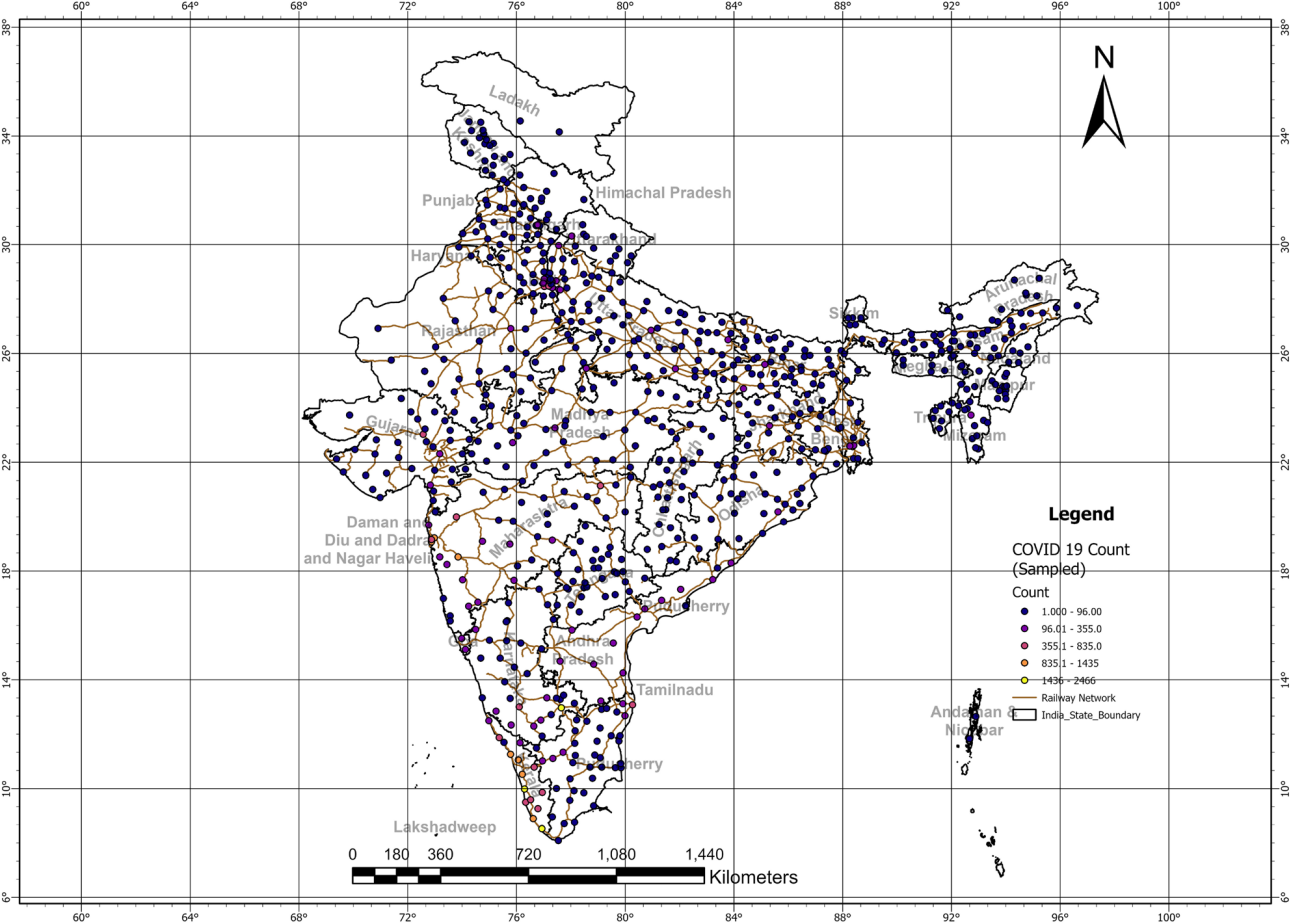
COVID-19 district counts (clusters) and interconnectedness of disease spread with the railway network.

The map shows that the spread of positive case clusters was connected well with the railway network (Fig. 2). Railways contribute an essential portion of India’s Gross Domestic Product and hence any decision related to Railways would directly impact the economy. (Invest India, https://www.investindia.gov.in/sector/railways, Retrieved 25th February 2023). Sampled aggregated clusters of COVID-19-positive cases were overlayed on Railways network lines in ArcGIS to understand the connectedness of the spread over three waves. A map is prepared for district-wise clusters of COVID-19 cases in India showing significant interconnectedness (Fig. 2). It has been found that Kolkata, Mumbai, and Kerala have relatively higher positive cases and it has been noticed that these areas are well connected with railway networks and road networks. However, the impact on the road network or a highly secured airport network is not that much more prominent than the railway network. Figure 2 shows the railway network overlaid with positive clusters on the map.

For a better understanding of spread pattern proportional clusters maps were prepared (Supplementary Folder 2). It was observed that until April 2020 there was no proper pattern for COVID-19 spread. This is supported by Moran’s I index values (Fig. 1). The areas having a higher number of cases at a given time are expected to have a higher probability in the near future also. It has been noticed that the districts in Maharashtra and Kerala had the highest proportion of COVID-19-positive cases during maximum times with interconnectedness between districts. A similar trend was observed previously in China by another study^15^. For this reason, careful interpretation is made for better understanding of clustering pattern of the disease spread.

### Detailed interpretation of clustering pattern

The analysis of COVID-19 cases in India has revealed that certain states such as Kerala, Maharashtra, and Tamil Nadu have reported the highest proportion of cases, while hilly areas located in the Himalayan part of India have reported relatively lower numbers of positive cases. This observation is consistent with previous studies that have shown that the spread of the virus is often influenced by factors such as population density, travel, and economic activity^16–18^. For further investigation of spatial patterns for COVID-19 cases in India, Moran’s I Index value trend (Fig. 1) was interpreted. The observation revealed that the maximum times Moran’s I Index values for COVID-19 cases in India were positive, indicating that there is a significant spatial clustering of cases. This suggests that areas with high COVID-19 cases are located close to each other in space.

The positive Moran’s I Index value trend (Fig. 1) is consistent with the initial findings, which showed that coastal regions in India, such as Kerala, Maharashtra, and Tamil Nadu, have consistently reported higher numbers of COVID-19 cases for three waves. And it has been noticed that there is also a variation in the cases reported in the Southern part and Northern parts of India.

High-clustered cases were observed in Kerala in 17 out of 33 months and in Maharashtra in 13 out of 33 months. In contrast, the northern states of India have had a relatively lower number of COVID-19 cases and have experienced lesser size clusters compared to the southern states. The observation reveals that any state above Maharashtra has observed clusters 16 times out of 33 months, though the intensity of positive cases is relatively lower than in southern states. These findings suggest that the spread of COVID-19 has been more prevalent in the southern states of India, with Kerala and Maharashtra being particularly vulnerable.

**Figure 3:**
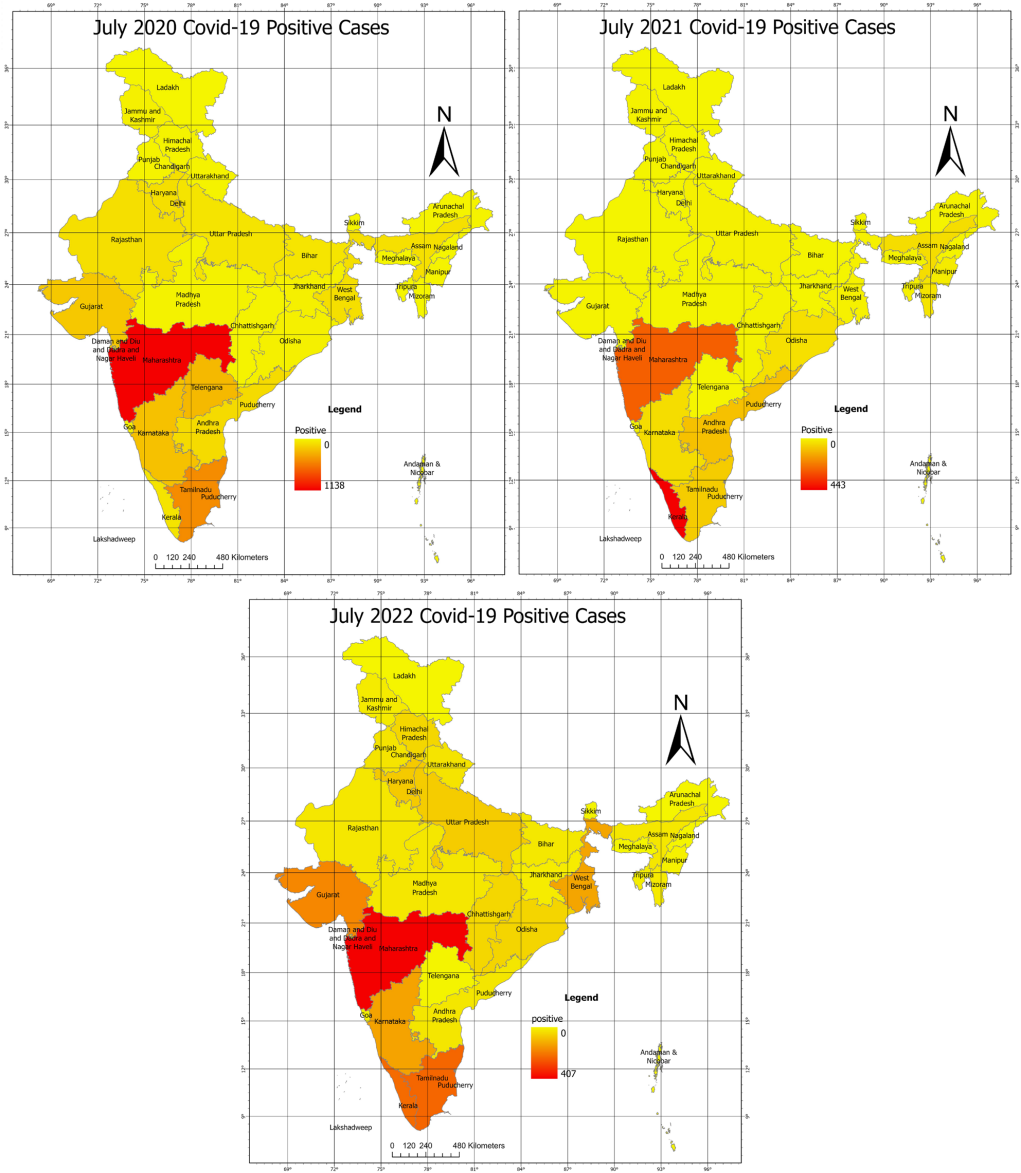
Comparative maps of COVID-19 sampled data based on quantile for (**a**) July 2020, (**b**) July 2021, and (**c**) July 2022. High-resolution maps for all months are available in Supplementary Folder 4.

**Figure 4:**
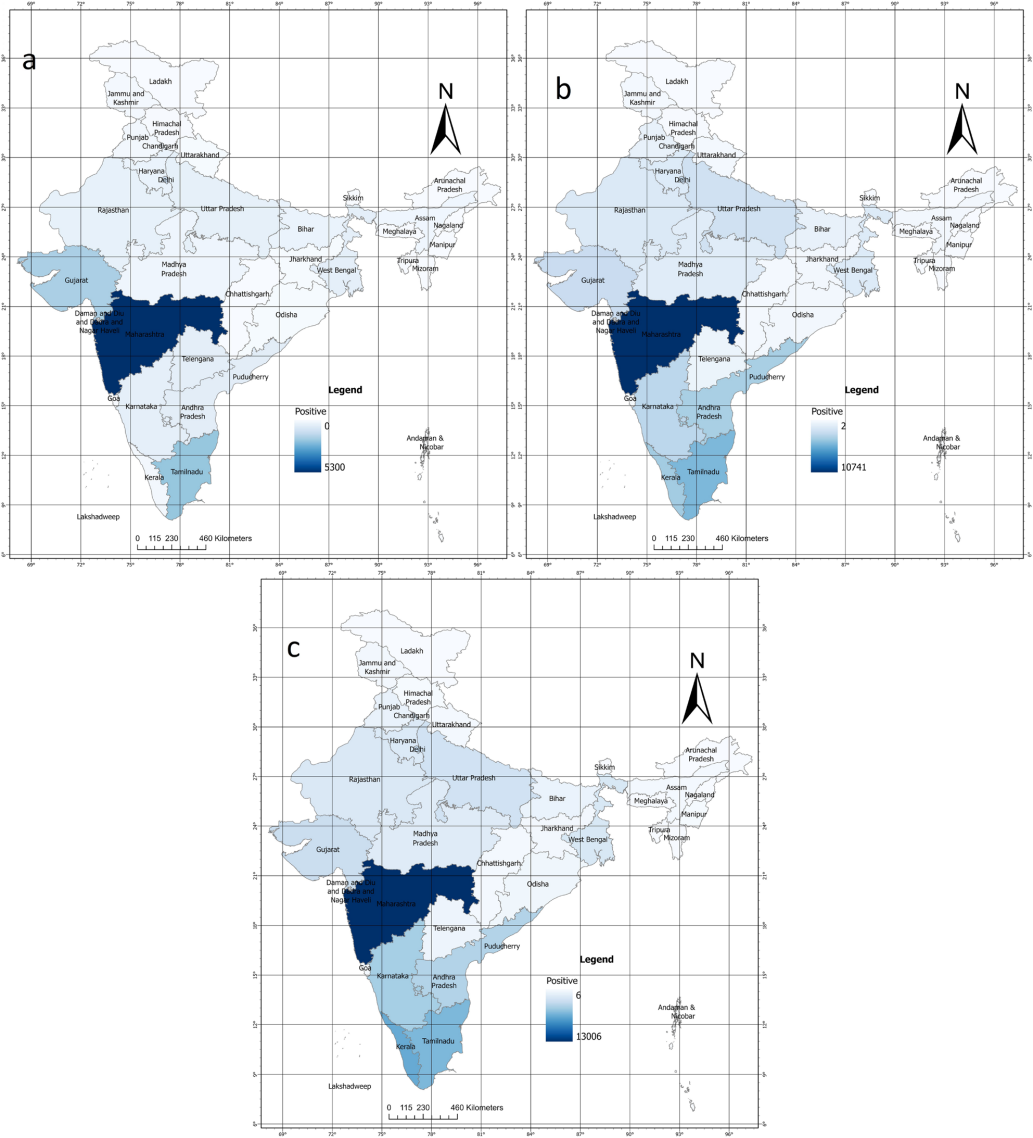
Aggregate COVID-19 positive sampled clusters maps of (**a**) July 2020, (**b**) July 2021, (**c**) July 2022.

**Figure 5:**
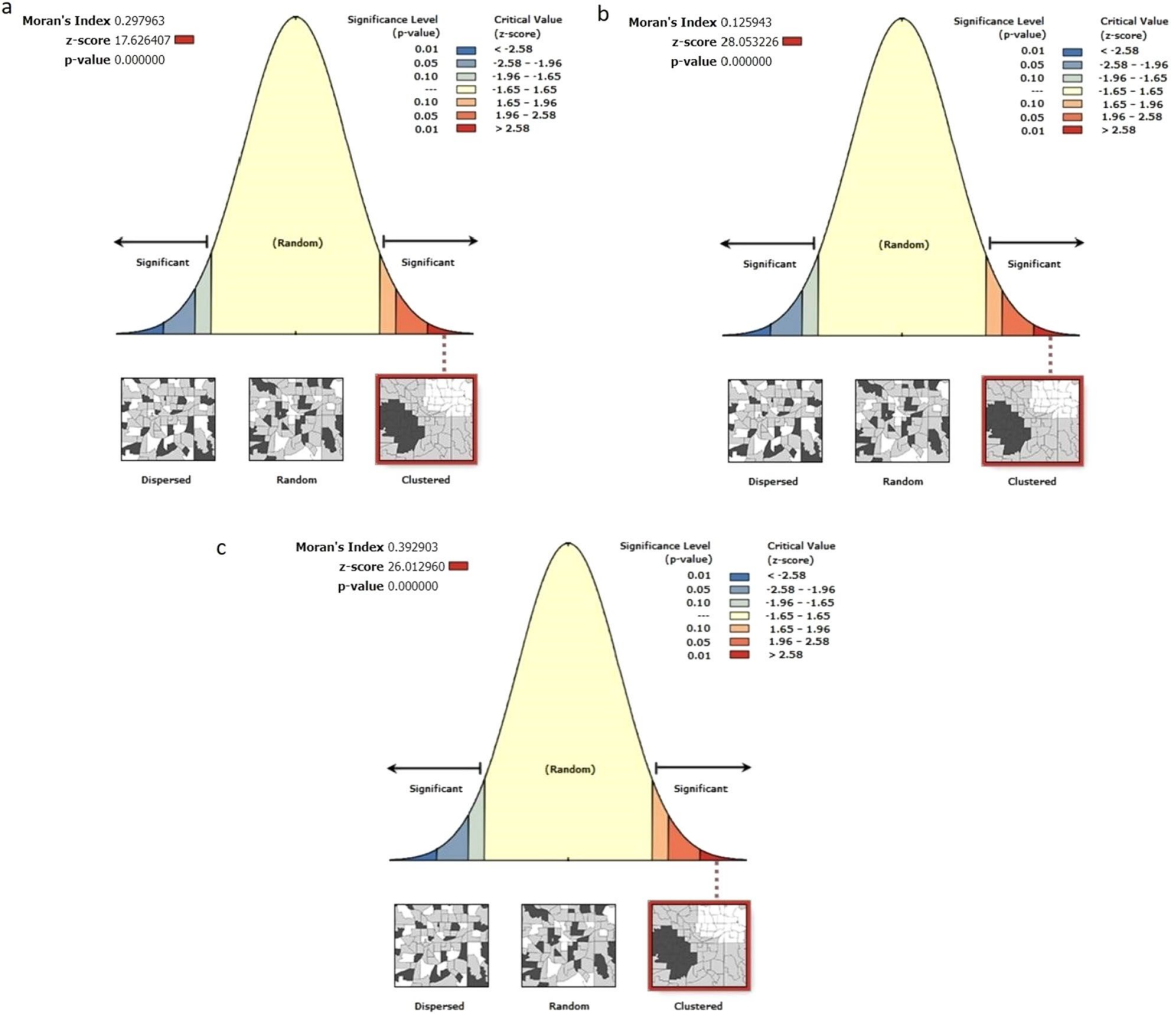
Moran’s I Index estimation results from ArcGIS pro for (**a**) July 2020, (**b**) July 2021, (**c**) July 2022 in India.

Figures 3 and 4 shows the clustering intensity in different regions for the months of July 2020, July 2021, and July 2022. In July 2020 and July 2021, the clusters were significant, as indicated by Moran’s I Index values of 0.30 and 0.13, respectively. Actual Moran’s I Index estimates are in Fig. 5. These results are consistent with the previous understanding in the same study that the southern states of India have had a higher incidence of COVID-19 cases and clustering. However, in July 2022, the positive case clusters were seen in different parts of India like West Bengal, although, the positive case clusters in the Southern region remained similar.

The Moran’s I Index value of 0.39 for July 2022 was comparatively higher than July 2021 and July 2020 and it has been seen that there is a significant spread in its surroundings with the increase of Moran I Index value. Whereas the lower values of Moran’s, I index show a low spread in the surrounding states. Similar kinds of observations are made with Getis ord Gi* statistics, wherein high-risk zones were observed in southern states for maximum times. The results highlight the importance of continuous monitoring of the spatial patterns of COVID-19 cases and the use of tools such as the Moran’s Index to identify regions that are particularly vulnerable to the spread of the disease. All other maps are presented in Supplementary Folder 4. This occurrence of high clustering of cases in coastal regions especially in the southern region of India could be attributed to a range of factors, including higher population density, travel, and greater economic and railways as stated earlier. Moreover, the positive Moran’s I Index value trend also suggests that there may be other underlying spatial factors driving the spread of COVID-19 in India.

The spatial relationship within climatic zones was also found. The most affected areas based on climate zone clustering were Tropical Monsoon, Tropical Savanna, Arid, Steppe, and Hot region. Cold regions like montane climate areas had a lower number of cases. Positive cases in these zones agree with the pattern of Moran’s I index values.

All the information on the climatic zone is available in Supplementary Table 2 and the top three contributing states from each month starting from March 2020 to December 2022 is available in Supplementary Table 3. A previous study observed that air temperature, humidity, solar radiation, wind speed indicators, and PM2.5 had a significant association with the COVID-19 newly infected cases in 2020–2021^19^. This study complements their findings as there could exist a relationship between the spread of COVID-19 and climatic variables.

### Pattern of symptoms, pre-medical condition and CT value in COVID-19 patients in India

The analysis of the common symptoms experienced by COVID-19 patients in India has provided valuable insights into the clinical presentation of the disease in the country. The study estimated that the most common symptoms occurring were Abdominal Pain, Breathlessness, Sore Throat, Cough, Fever, Nasal Discharge, Body Pain, Loss of taste, Loss of smell, Cold, General Weakness, Nausea, and Vomiting. Of these symptoms, Fever, Cough, and Breathlessness accounted for more than 77% of the COVID-19 cases in India, with an increasing trend observed in the number of positive cases from 2020 to 2022.

These findings highlight the importance of early identification and management of these symptoms in the context of COVID-19. The study also identified the major pre-medical conditions reported by COVID-19 patients in India, which included Chronic Renal Disease, Diabetes, Heart Disease, Hypertension, Malignancy, and Obesity. More than 90% of patients with pre-medical conditions reported having heart disease, chronic kidney disease, hypertension, and diabetes. These results have significant implications for the management and treatment of COVID-19 in India, particularly in the context of vulnerable populations with pre-existing medical conditions. The findings underscore the need for targeted interventions to address the underlying health conditions that increase the risk of severe COVID-19 outcomes.

The analysis of the cycle threshold value of RTPCR in COVID-19 positive cases in India has revealed interesting findings regarding the viral load in patients over time. The study categorized the CT values into three conditions, with a CT value of less than 25 indicating a high viral load, a CT value of 25–35 indicating a medium viral load, and a CT value above 35 indicating a low viral load (Table 1). The analysis found that in COVID-19-positive cases with valid CT values, there was a high viral load in all 3 years—2020, 2021, and 2022. This indicates that the viral load in COVID-19 patients has remained consistently high over time. The study also found that there was a positive spatial autocorrelation between viral load and positive cluster connectedness, with a Moran’s I Index value of 0.37. The analysis also found that viral load increased in patients while the number of positive cases decreased from 2020 to 2022. This suggests that even as the overall number of COVID-19 cases in India decreased over time, the viral load in individual patients remained high. These findings have significant implications for the management and treatment of COVID-19 in India. The positive spatial autocorrelation between viral load and positive cluster connectedness highlights the need for targeted interventions to address the clustering of COVID-19 cases in specific geographic regions.

**Table 1:** Viral load characteristic in a sampled dataset.

Viral load condition	2020 (%)	2021 (%)	2022 (%)
High	49.44	62.11	62.81
Medium	46.47	37.08	35.29
Low	4.09	0.81	1.90

## Discussion

**Figure 6:**
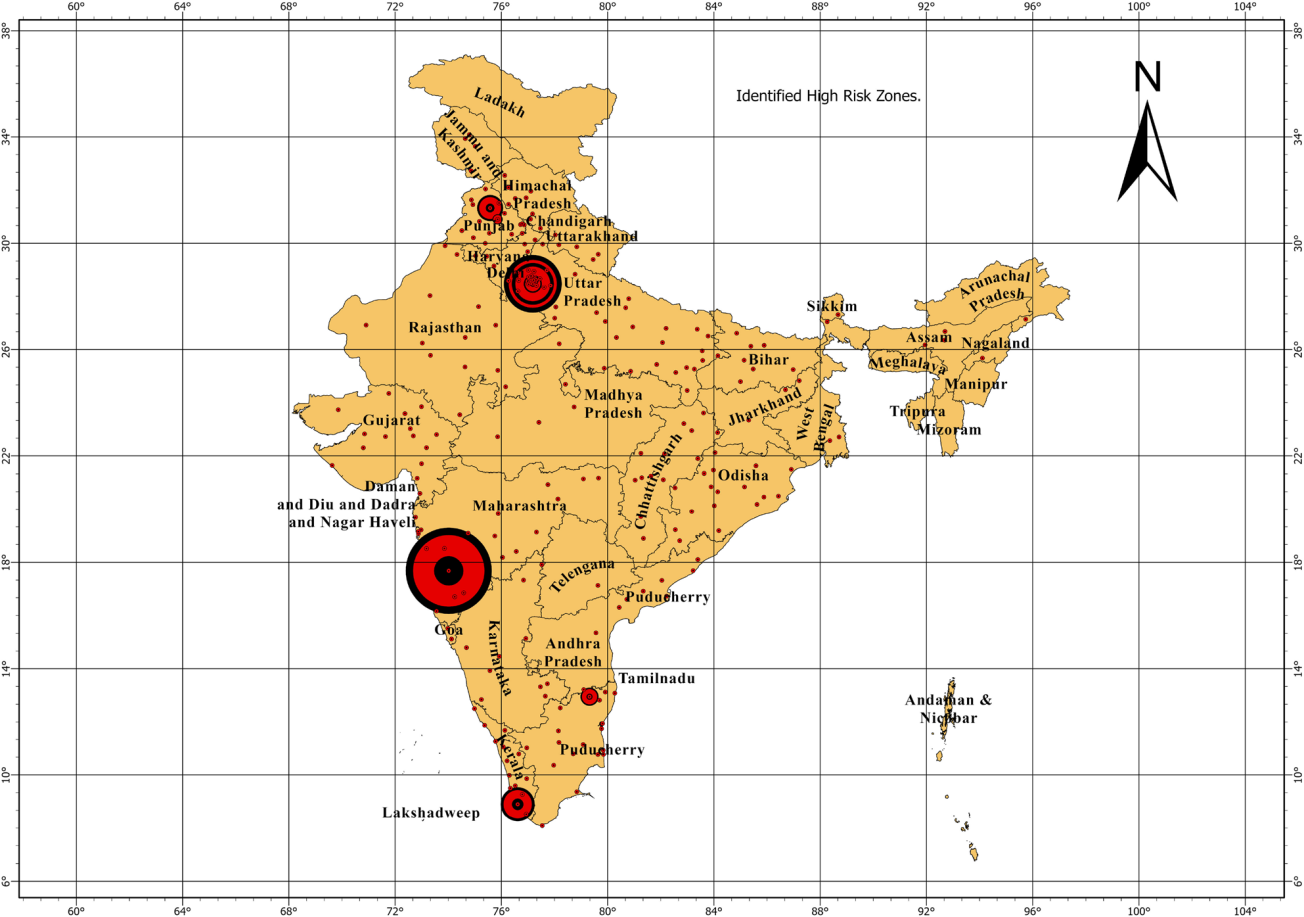
Highest number of cases were observed in the locations which had higher number of cases in the past. Concentric circles by ascending shows intensity of occurrence of positive cases.

This study has given a scientific understanding that high spatial autocorrelation could be responsible for the similar number of cases around the places having higher COVID-19 cases. This means that the areas surrounding the districts or states with high COVID-19 cases are more likely to have similar numbers of cases. A similar observation was made recently during the last week of March 2023 and April 2023 in India. There was a rise in COVID-19 cases in this timeframe in India. It was observed that high cases were reported in the states having high risks as identified in this study. Out of 9355 daily cases reported on 27th April 2023, 10:09 AM, Kerala, New Delhi, and Maharashtra contributed the highest with 2199, 1040, and 784 new daily cases respectively. (Source: My Government Portal, Government of India, https://www.mygov.in/covid-19/, retrieved 27th April 2023). To further understand the quality of this study, based on time bound convenience sampling approach, data is downloaded for Apr 1, 2023, Apr 6, 2023, Apr 11, 2023, Apr 16, 2023, Apr 21, 2023, and, Apr 26, 2023. Here also similar kind of trend was observed, thus validating the interpretations (Fig. 6).

As per the above observation, if cases rose sharply in a few districts located along major railway routes and clustered in hot, humid climate zones, this could signify viral spread along transit networks and environmental conditions conducive to transmission. The spike in these districts may lead to wider diffusion across connected regions in future. This may be applicable to future pandemics also. It underscores the importance of strategic mobility restrictions and tailored protocols for high-risk zones evidenced in the spatial analysis.

In India, decisions taken by authorities at the regional level have played a crucial role in controlling the spread of the COVID-19 pandemic. The measures taken by the government in the state, such as lockdowns, contact tracing, and mass testing, helped to limit the spread of the virus in the areas nearby the high-risk zones^20,21^. Though, for a highly populated nation like India, it is important to consider the travel networks, especially the Indian railways.

Railways networks are considered the backbone of Indian economy. Indian Railways is one of the largest Railway networks in the world. It transports millions of passengers and tons of goods across the country every day. Indian railway connects various parts of the country, like rural areas, to urban centers. This helps in the overall economic development of the country. The COVID-19 positive cluster’s interconnectedness with the Railway network is an important factor to consider in informed policy decisions for nations. Meanwhile, the other important factors which need to be considered are climatic zones and geographical locations. However, a more important thing to consider as specified in this study is regular monitoring and interpretation of Moran’s I Index at a particular time.

Periodic Moran’s I analysis would detect emerging COVID-19 spatial clusters indicating active viral spread between proximal districts. Monitoring trends in the index’s z-scores would identify intensifying hotspots requiring interventions like testing, restrictions, and healthcare surges. This data-driven approach allows targeted response alignment with shifting transmission patterns, facilitating efficient resource allocation. Updating the index enables measuring policy impacts on localized outbreaks too.

## Conclusion

The findings of this study provide evidence that spatiotemporal analysis can be an effective tool for understanding the spatial patterns of COVID-19 disease spread. The study found that there is a positive correlation in positive COVID-19 cases in India from May 2020 until the present day. This trend was observed across different states in India. It successfully identified high-risk zones such as Kerala, Maharashtra, New Delhi, Tamil Nadu, and Gujarat. It was observed that these high-risk zones were mostly located near coastal areas and hotter climatic zones. By understanding the spatial patterns of disease spread, governments can take targeted measures to prevent the further spread of the virus, such as implementing localized lockdowns, increasing testing in high-risk areas, and providing additional medical resources to affected regions. Future research could investigate the effectiveness of measures such as proper vigilance in railway traffic or increasing sanitation measures in railway stations and on trains in reducing the spread of COVID-19.

Routine spatial tracking enables preemptive, localized response alignment as viral hotspots shift, facilitating targeted resource allocation. Strategic implementation of mobility restrictions can balance pandemic control with economic impacts. Environmentally-tailored protocols are needed to adjust for climate variations between regions. Regular cluster identification via tools like the Moran’s I Index allows responsive mitigation as outbreak epicenters move. A data-driven, nimble yet measured approach is key for policymakers to strategically manage future waves in this diverse nation.By identifying viral hotspots and mobility networks, the analysis enables targeted restrictions and resource deployment to emerging clusters. Climate zone correlation allows localized protocols tailored to zone-specific transmission risks. Regular statistical monitoring via Moran’s I promotes agile response alignment as disease landscapes shift. These data-driven spatial insights inform strategic pandemic preparedness policies and planning for rapid, focused mitigation. Overall, the geospatial tracking and analysis empowers stakeholders with knowledge to address outbreaks efficiently.

By elucidating data-driven insights into how infections spread geographically, this methodology equips researchers with techniques to uncover trends, correlations and risk factors. In turn, these findings can inform predictive models, surveillance strategies, and targeted policies for future pandemic preparedness and control. Thus, combining robust geographic information systems with epidemiological datasets provides a pathway for impactful and actionable future research. Although, this study was confined to spatial linking of cases to mobility and climate zones, necessitating incorporation of genetics, demographics, and economic factors in future modelling. Expanding spatial scale would offer a broader perspective, and longitudinal tracking would reveal long-term diffusion trends. Integrating machine learning prediction with spatial analytics could strengthen outbreak forecasting and surveillance.

## Methods

### Study procedure, participants, and ethics

The present study is based on the data received from the Indian Council of Medical Research (ICMR). A retrospective cross-sectional study of positive COVID-19 cases reported to ICMR was carried out to visualize and predict the COVID-19 disease spread in India. Since this project was entirely dependent on the COVID-19 data of India that is available with ICMR, a Government Organization, permission from ICMR for the utilization of COVID-19 data was sought prior to the commencement of the study through a research proposal. All methods were carried out in accordance with relevant guidelines and regulations after the approval of the research proposal by ICMR under project ID 2021-6393. All experimental protocols were approved by ICMR under the same project ID for visualization of COVID-19 using GIS maps and prediction of disease spread through a machine learning approach.

Since the research utilized retrospective data which were anonymized and encrypted and did not involve any kind of patient intervention or interaction with the patients, there was no scope for obtaining informed consent considering the nature of the study. The research proposal was presented before the Project Review Committee (PRC) of ICMR in New Delhi, for assessment of scientific and ethical aspects of the study, and necessary approval was obtained. Subsequent to the approval of the protocol, ICMR provided funding for the study along with the anonymized encrypted data without patient identifiers. Hence, the data provided under ICMR project ID 2021-6393 didn’t have any personal identification information like name, images, exact addresses, videos, phone numbers, email ids, etc. Personal information is encoded and ICMR solely owns the original data, authors only own the encrypted data after an agreement of confidentiality. Our research protocol has taken care of every aspect of ethical principles and regulatory norms and has adhered to the principles of the Declaration of Helsinki 1975 and its later amendments.

### Data selection and sampling

The dataset includes COVID-19 testing information along with the test results. Significant variables in the dataset were the Cycle Threshold (CT) Value, Symptoms, and Pre-Medical condition of every patient in districts and states of India. Among these variables, CT values indicated the viral load in patients whose testing has been done through real-time PCR (RT-PCR), and the other indicated symptoms like fever, cough, breathlessness, etc., and pre-medical conditions like Chronic Renal Disease, Heart Disease, and Hypertension, etc. Only the reported data related to Symptoms and Pre-Medical conditions are considered in this analysis after the rejection of NULL values by utilizing the pandas module within the Python environment.

These variables are included as CT Values provide insights into viral load and transmission potential in positive cases across India. Symptom analysis characterizes the clinical presentation and epidemiological patterns of COVID-19. Pre-existing conditions help identify vulnerable populations with higher risks of complications. Together these key variables offer crucial understanding of the virological, clinical, and epidemiological profile of the pandemic across the nation.

A time-bound convenience sampling approach is used for data download. The datasets were downloaded from last week of March 2020 till the last week of December 2022 from the ICMR COVID-19 data bank with an ideal batch size. The ideal batch size refers to the number of COVID-19 cases downloaded in each 5-day sampling interval, optimized by ICMR’s server to achieve computational feasibility. In each batch, information on 0–15,000 random test results was downloaded via ICMR’s server based on the day when cases were confirmed while using the Postman Application Programming Interface. This automated approach optimized batch sizes for spatial pattern analysis. In this time-bound convenience sampling approach, 5 days gap is considered to reject the repeat cases. From this approach, we get 93.39% unique cases in total downloaded data (based on Table 2 data). Hence, data acquisition has ensured a minimal amount of risk by not including cases that may have already been resolved or new cases that have not yet been identified. A major objective for choosing this sampling method is to understand the underlying patterns in a specified time frame.

**Table 2:** Characteristics of a sampled dataset.

Year	Number of tests	Number of positive cases	Number of negative cases	Positive to test ratio	Unique positive patients	Unique cases (%)	Rejected samples
2020	392,950	45,144	337,605	0.15	39,597	87	10,201
2021	643,116	28,666	609,041	0.04	28,414	99.12	5409
2022	697,756	18,067	674,864	0.03	18,014	99.70	4825

The dataset used in this study was taken from 738 Districts of India, as mapped in Google Maps Application Programming Interface (API), details are provided in Supplementary Table 1. The district data is first converted into latitude and longitude through API and a Microsoft SQL server. APIs from Google allow the integration of data with Google Maps. The Geocoded coordinates are not exact addresses but the central location of the district. Hence, the locations are only indicative of central district locations.

From this approach, the peak duration for COVID-19-positive cases in India was correctly identified. As per the reports, the peak months of the first wave in India were in September 2020, in the second wave the peak month was April 2021 and in the third wave, the peak month was January 2022. As per the observations during our sampled data visualization process, we also observed that COVID-19 positive cases rose to 70.04% during peak months of the first wave which was near September 2020. This increase was significantly reduced in later months with a sharp decline in December 2020. While in April 2021, there was a rise of 65.13%, and in January 2022, there was increase of 91.60%. These percentage rises are higher compared to the rest of the months. It has been visualized that there was a slight increase in COVID-19 cases in India during June–July 2022. This validates the sampling method.

Table 1 shows the estimates for the sampled dataset. The total number of test results is 1,733,822 out of which 91,877 are positive cases and 1,621,510 are negative cases. 86,304 positive cases are unique or have no repetition in the dataset i.e. 93.39% of the positive cases sample. “Unique positive patients” in Table 1 represent the total number of unique ICMR-ID. As ICMR had assigned a unique id for each individual patient. Based on Table 1 and standard equations we achieved a confidence interval > 99% and a margin of error less than 1% for this time-bound approach.

### Spatio-temporal analysis

**Figure 7:**
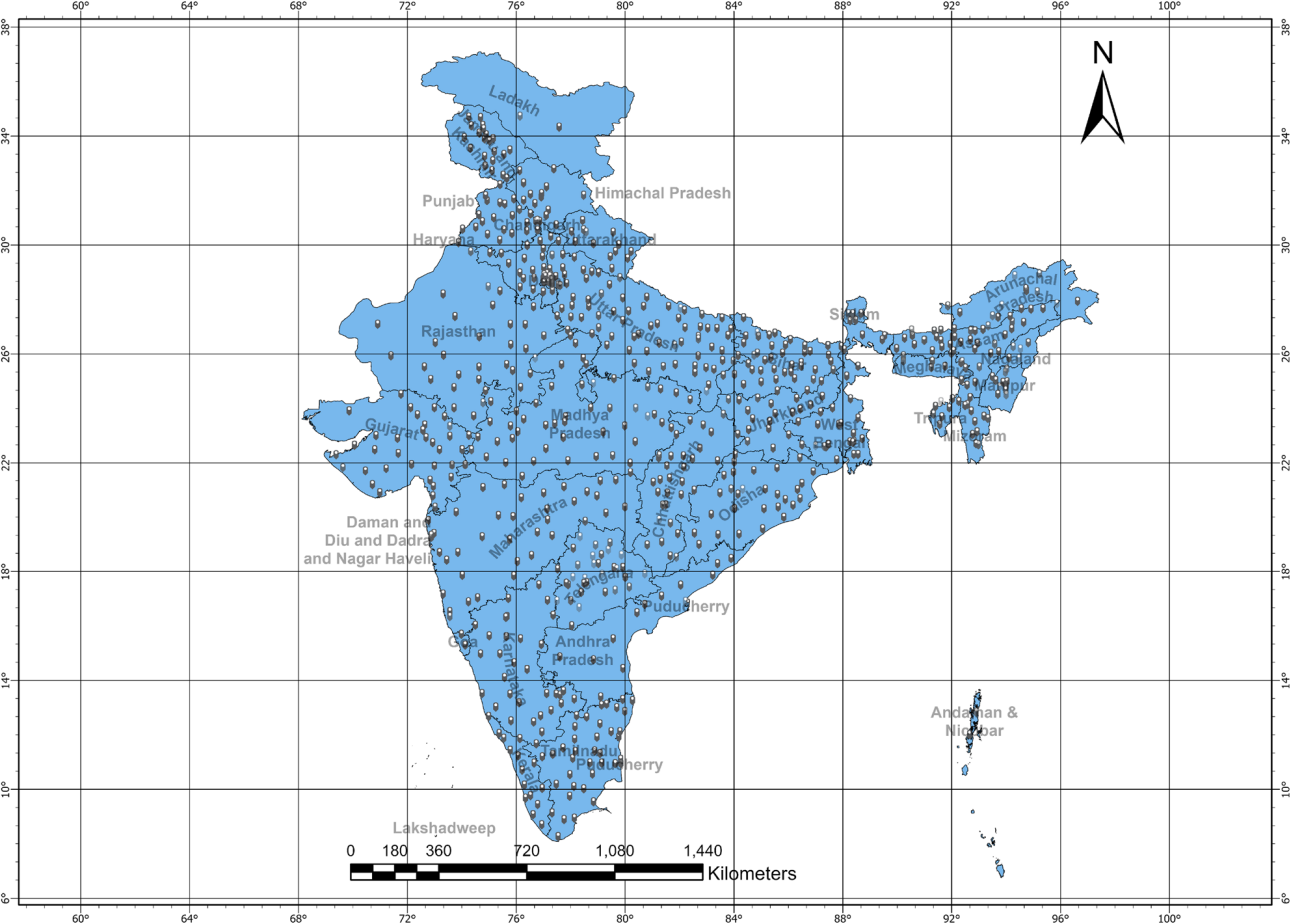
Outline of Indian Sub-Continent with state boundaries and district locations. A list of districts and latitude–longitude information is in Supplemental Table 1.

The study area for this study is whole of India (Fig. 7) with all states and districts. South zones beyond the state of Maharashtra and the north zone above it were assessed for spatial clustering. The first case of COVID-19 in India was detected on January 27, 2020, in the state of Kerala^22^ who had returned from Wuhan, China on January 23, 2020. Since then, there has been a rapid spread of COVID-19 in all the states and districts of India. All the states and districts were affected by the virus. This led the Government to implement a lockdown policy promptly to stop the disease’s spread. Till the time of preparation of this manuscript, India has observed three large waves of COVID-19 that has impacted millions of lives. It has severely impacted the economy, healthcare, and education. India has different climatic zones such as Tropical Rainforest Climate, Tropical Monsoon Climate, Subtropical Climate, Arid Climate, Himalayan Climate, and Alpine Climate. Many studies have observed that there is a relation between climatic zones and the rate of COVID-19 spread in India^23,24^.

Spatial statistics was carried out on variables like monthly count, CT Value, symptoms, and pre-medical condition of every patient. Since the data were sampled and a null hypothesis is designed to make the study more concrete the clustering pattern-related characteristics of data is random. This ensures that on a rejection of the null hypothesis we can achieve a pattern of COVID-19 spread. Moran’s, I index is based on a similar null hypothesis. Moran’s, I index is a measure of spatial autocorrelation that assesses whether neighboring observations of a variable are more alike (positive spatial autocorrelation) or more different (negative spatial autocorrelation) than expected by chance. Value of Moran’s I index ranges from − 1 to 1, where − 1 indicates perfect dispersal and 1 indicates perfect clustering^25^. Moran’s I index is widely used in spatial statistics to assess the degree of clustering or dispersion of a variable in space^26^. So, Moran’s I index (global) was considered due to this reason and to understand the intensity of the clustering pattern.

1 $$\begin{aligned} I = \frac{{n \sum \nolimits _{i=1}^{n} \sum \nolimits _{j=1}^{n} \left[ W_{ij}(y_i-\bar{y})(y_j-\bar{y}) \right] }}{{\sum \nolimits _{i=1}^{n} \sum \nolimits _{j=1}^{n} W_{ij} \left[ \sum \nolimits _{i=1}^{n} (y_i - \bar{y}) \right] ^2}} \end{aligned}$$

where n is the number of districts, $y_i$, $y_j$ is the cluster for positive COVID cases for the districts *i* and *j*. $\bar{y}$ is the mean for positive cases and $W_{ij}$ is the weight index for the district i relative to j in the spatial domain. Here, spatial weights represent the closeness of the districts and the mean and deviations are estimated for the number of positive cases per district.

For the creation of hotspot maps, Getis Ord Gi* statistics is used in ArcGIS. A Getis-Ord Hot Spot Analysis (also known as Gi* statistic) is a spatial analysis technique used to identify statistically significant spatial clusters of high or low values (hot spots and cold spots) of a given attribute in a geographic dataset. This method takes into account the spatial distribution of the data and the degree of spatial autocorrelation to calculate a Z-score and p-value for each feature in the dataset, indicating whether it is part of a significant cluster or not. The result is a thematic map showing the locations and sizes of the hot spots and cold spots, providing insights into the spatial patterns and trends of the attribute being analyzed. Monthly COVID-19 maps were prepared and an overall map for the whole nation has been created to understand the most impacted areas during the last 3 years.

After the evaluation of the Moran I index from sampled raw data, the Railway network map was overlaid on an aggregate map for interpretation of the spatial pattern.The railway network shapefile was overlaid on the COVID-19 case map to combine the spatial data layers, enabling joint visualization and analysis. This overlay highlights case clusters along major transit routes, aiding interpretation of how railway connectivity likely enabled viral spread between the clusters linked centers. The combined map allows discernment of critical network links associated with cases versus isolated areas. Also, the climate zones were assessed to understand which zones contributed to a higher percentage of COVID-19 cases in India. The railway network was analyzed to assess whether population mobility along transit routes influenced the spatial spread of COVID-19 infections across India. Climate zones offered broad environmental demarcations to examine if temperature extremes in hotter or colder regions, impact viral transmission and seasonal case trends. Together these geographic factors provided insights into how human movement and ambient weather conditions may have modulated the progression of the pandemic. There are a number of literature found who have explored the relationship between climate and COVID-19 disease spread^2,19,27–29^. The relationship between climate and COVID-19 is a complex and multifaceted topic that has been studied by researchers in various disciplines. One of the most studied aspects is the relationship between temperature and humidity and the spread of the virus. Some studies have suggested that higher temperatures and humidity levels may decrease transmission of the virus, while colder and drier conditions may increase transmission^30–32^

Close contact and mobility are known facilitators of transmission. In India, railways are the predominant mode carrying  8.6 billion passengers in 2022, which is comparatively high exceeding other networks like road and air. Railways are also known as the backbone of the Indian economy (Invest India, https://www.investindia.gov.in/sector/railways, Retrieved 25th February 2023). Overlaying the railway map with case clusters revealed notable alignment, implying transit routes enabled diffusion across proximate districts. This warrants attention given rail’s outsized GDP share; interventions could significantly impact the economy while informing policy response. Further, COVID-19 cases were mapped in different climate zones to enable visual analysis of transmission trends across different climate zones across the study area using ArcGIS pro software. Delineating cases by zone provides insights to guide targeted interventions based on type of the environment. Along with the overlays, the Getis-Ord Gi* tool was used to calculate hotspot/coldspot clustering based on the magnitude of COVID-19 cases in nearby districts. It identifies statistically significant hotspots and coldspots through z-scores and p-values. This enabled data-driven delineation of spatial clusters, which were visualized as hot and cold spots on maps to analyze diffusion patterns. In summary, the Getis-Ord Gi* statistics quantitatively detected clustering and then hotspot mapping visualized the spatial patterns, This links the statistical analysis to geographic interpretation.

### Supplementary Information

Supplementary Information 1.

Supplementary Information 2.

Supplementary Information 3.

Supplementary Information 4.

Supplementary Table 1.

Supplementary Table 2.

Supplementary Table 3.

## Data Availability

The data that support the findings of this study are available from ICMR but restrictions apply to the availability of these data, which were used under license for the current study, and so are not publicly available. However, these data are available from Harpreet Singh (Division of Biomedical Informatics, Indian Council of Medical Research, email: icmrhqds@sansad.nic.in) upon reasonable request and with permission of ICMR. Maps generated are available on the Zenodo repository with the following link 10.5281/zenodo.7981043.
