# Supplementary figures and images for: Tracing the COVID-19 spread pattern in India through a GIS-based spatio-temporal analysis of interconnected clusters (part 1 of 2)
Source: Sci Rep. 2024 Jan 8;14:847. doi: 10.1038/s41598-023-50933-4 (PMC10774287; doi:10.1038/s41598-023-50933-4)

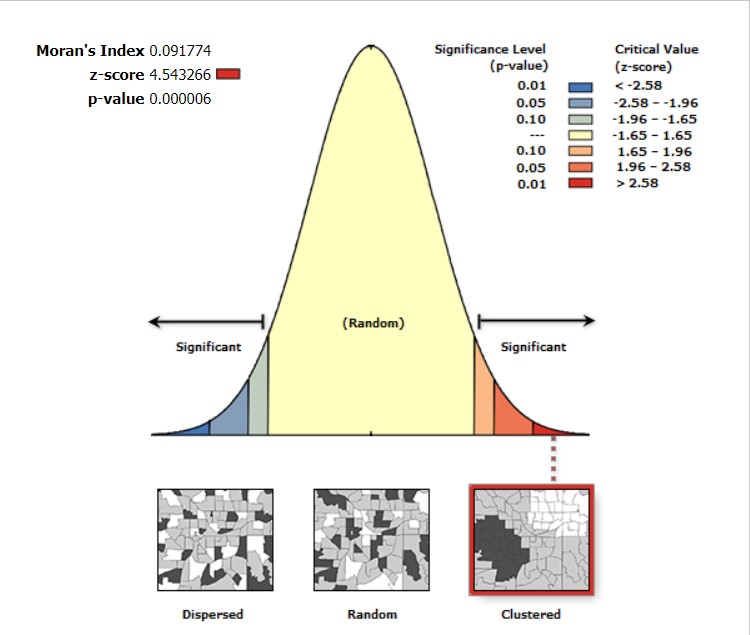

Supplement: Supplementary file 1 — Supplementary Information 1. [file 41598_2023_50933_MOESM1_ESM.zip › April 2020.jpg]

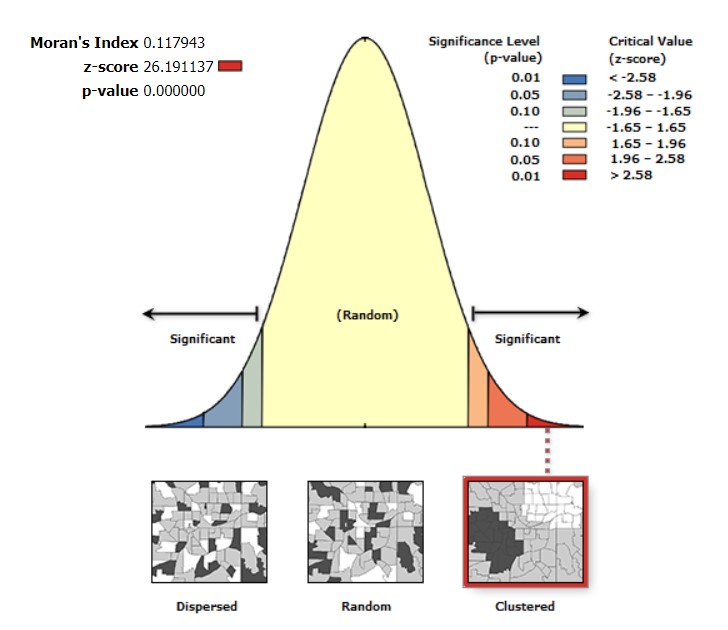

Supplement: Supplementary file 1 — Supplementary Information 1. [file 41598_2023_50933_MOESM1_ESM.zip › April 2021.jpg]

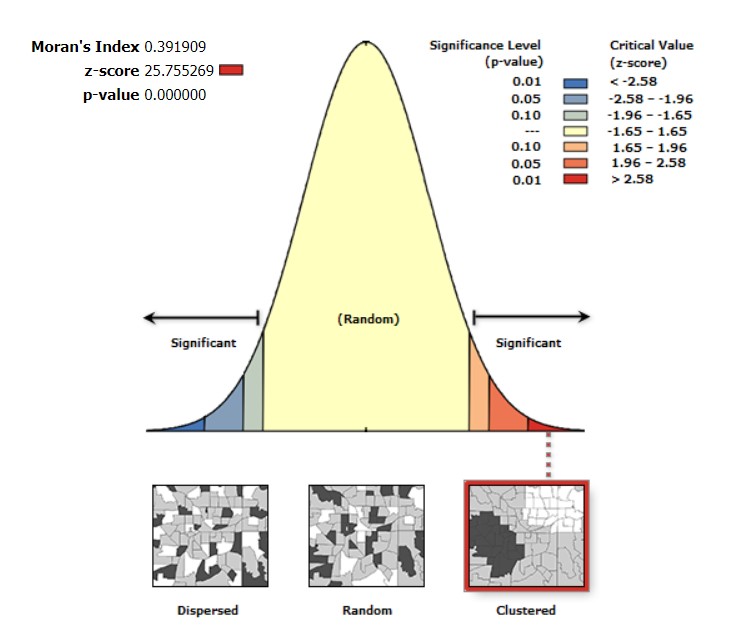

Supplement: Supplementary file 1 — Supplementary Information 1. [file 41598_2023_50933_MOESM1_ESM.zip › April 2022.jpg]

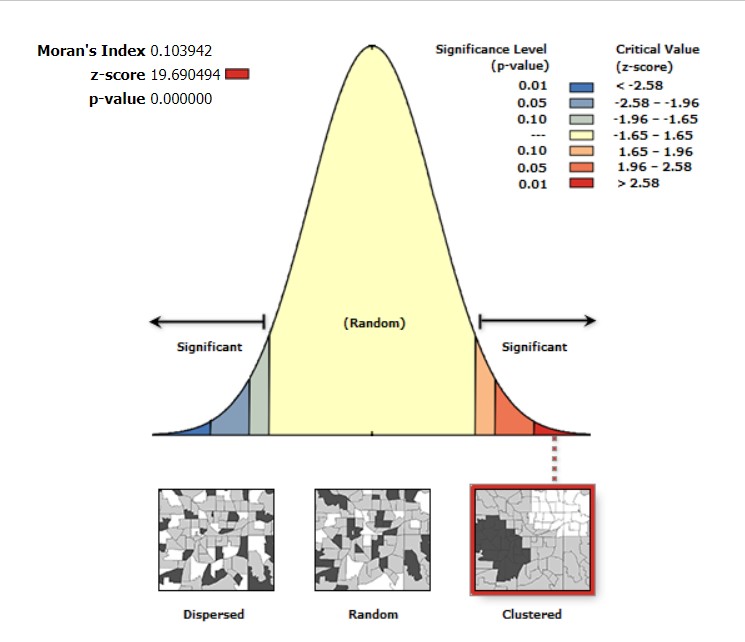

Supplement: Supplementary file 1 — Supplementary Information 1. [file 41598_2023_50933_MOESM1_ESM.zip › August 2020.jpg]

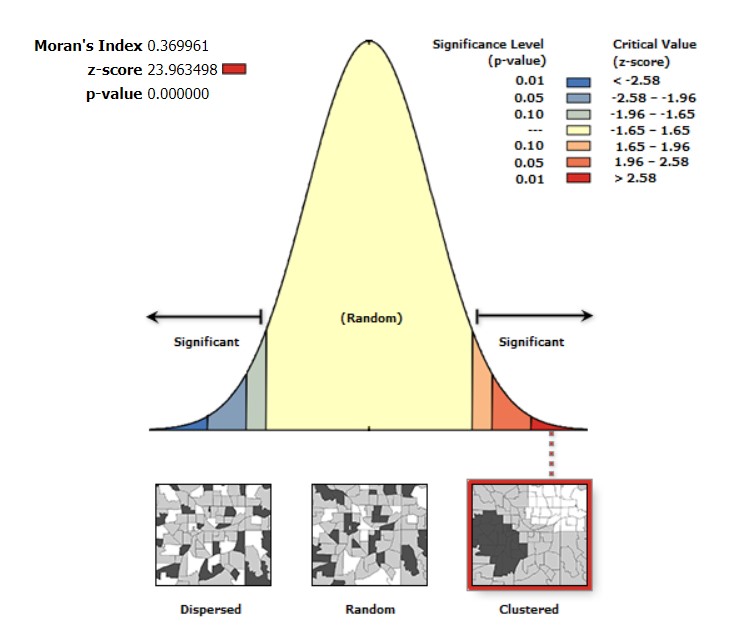

Supplement: Supplementary file 1 — Supplementary Information 1. [file 41598_2023_50933_MOESM1_ESM.zip › August 2021.jpg]

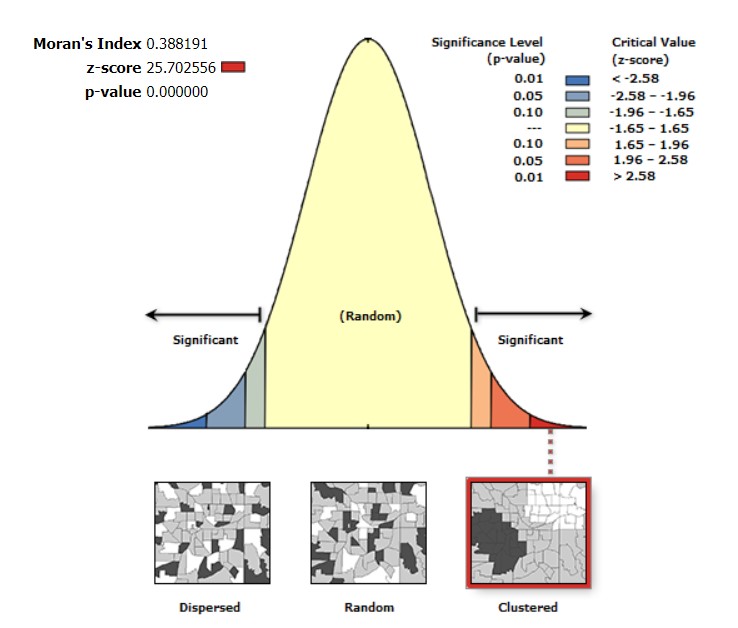

Supplement: Supplementary file 1 — Supplementary Information 1. [file 41598_2023_50933_MOESM1_ESM.zip › August 2022.jpg]

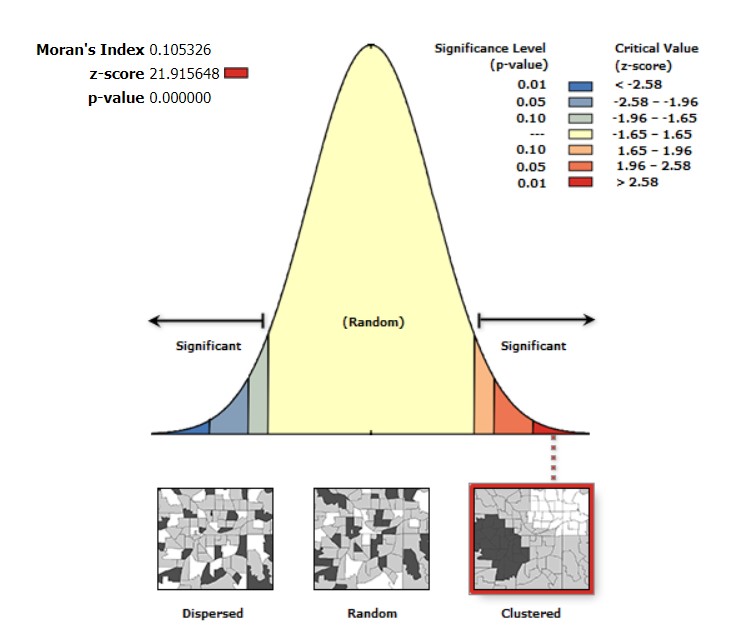

Supplement: Supplementary file 1 — Supplementary Information 1. [file 41598_2023_50933_MOESM1_ESM.zip › December 2020.jpg]

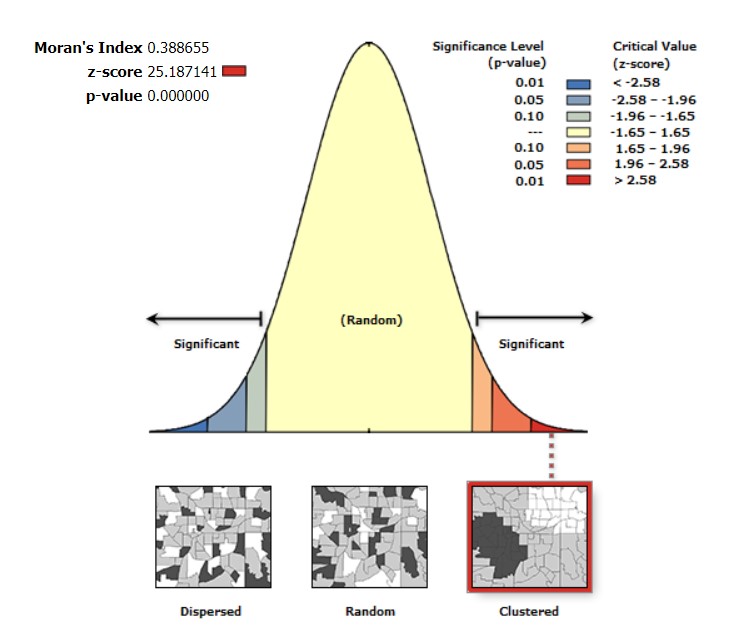

Supplement: Supplementary file 1 — Supplementary Information 1. [file 41598_2023_50933_MOESM1_ESM.zip › December 2021.jpg]

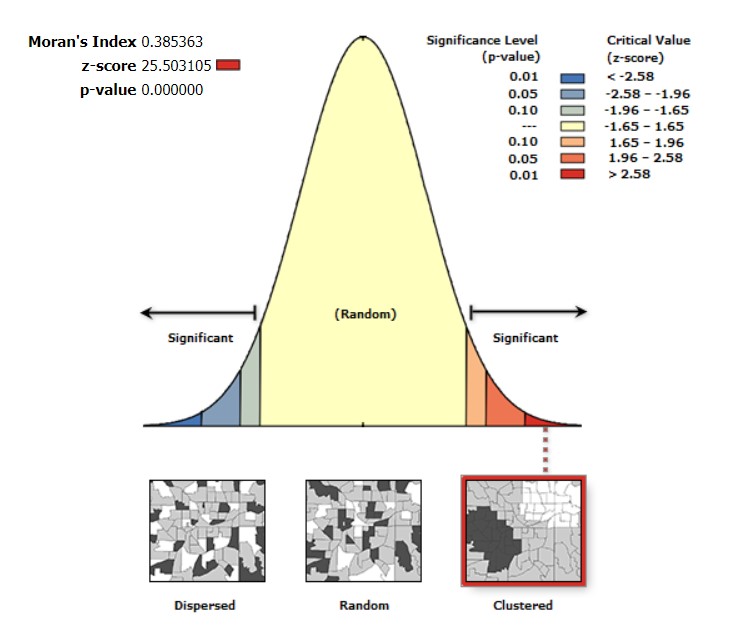

Supplement: Supplementary file 1 — Supplementary Information 1. [file 41598_2023_50933_MOESM1_ESM.zip › December 2022.jpg]

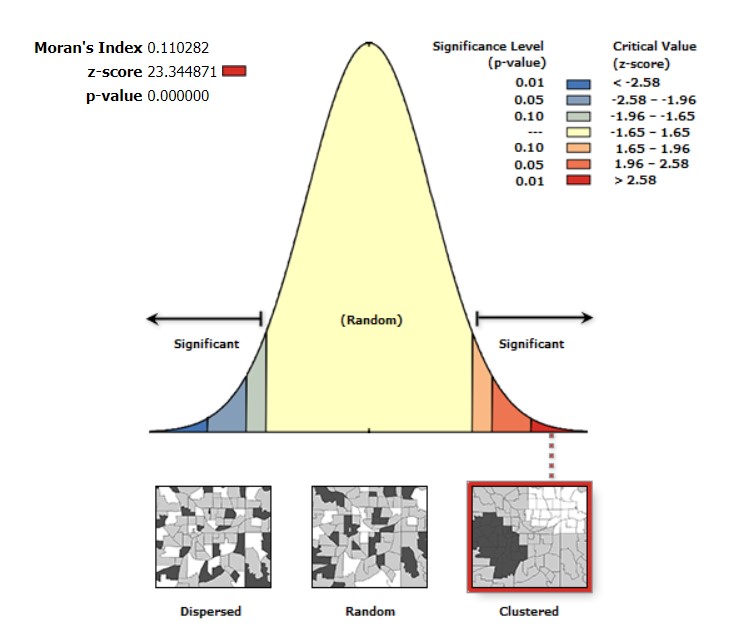

Supplement: Supplementary file 1 — Supplementary Information 1. [file 41598_2023_50933_MOESM1_ESM.zip › February 2021.jpg]

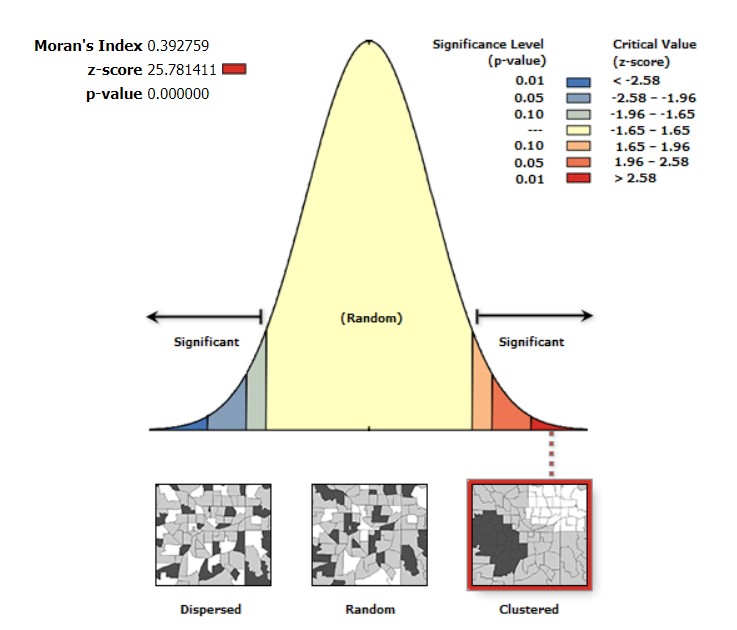

Supplement: Supplementary file 1 — Supplementary Information 1. [file 41598_2023_50933_MOESM1_ESM.zip › February 2022.jpg]

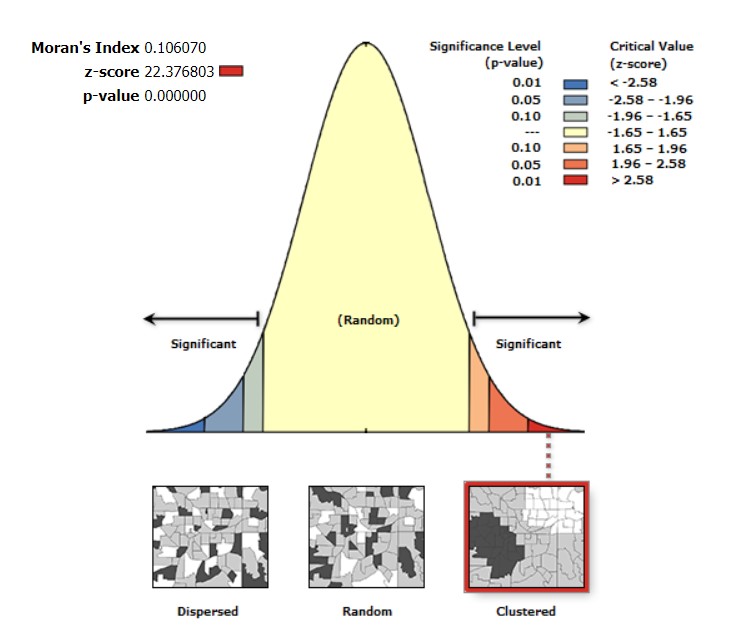

Supplement: Supplementary file 1 — Supplementary Information 1. [file 41598_2023_50933_MOESM1_ESM.zip › January 2021.jpg]

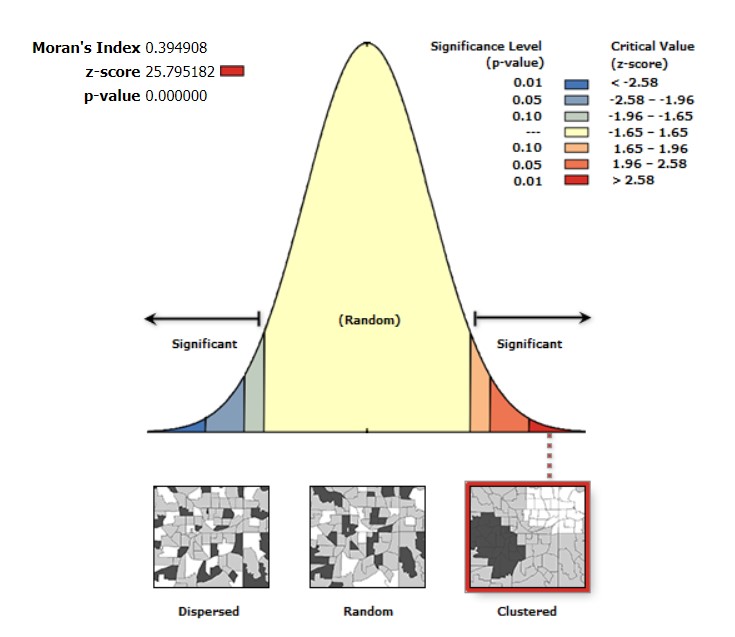

Supplement: Supplementary file 1 — Supplementary Information 1. [file 41598_2023_50933_MOESM1_ESM.zip › January 2022.jpg]

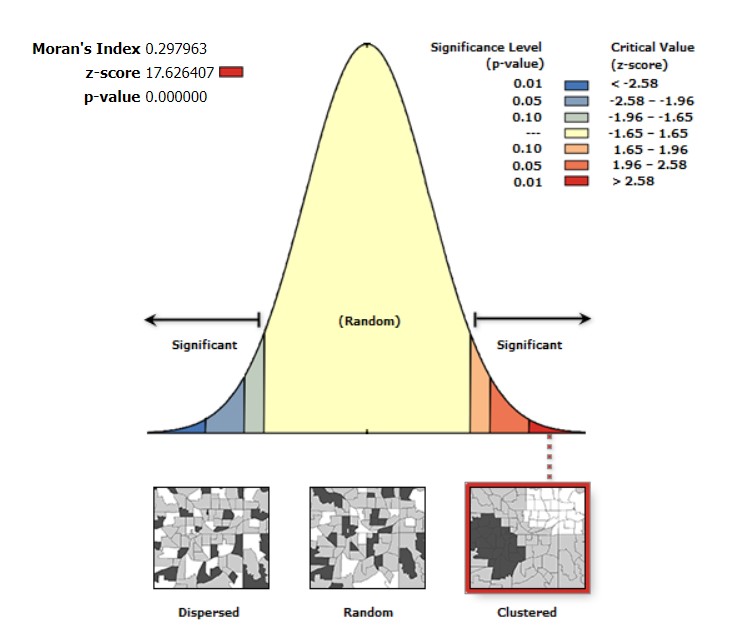

Supplement: Supplementary file 1 — Supplementary Information 1. [file 41598_2023_50933_MOESM1_ESM.zip › July 2020.jpg]

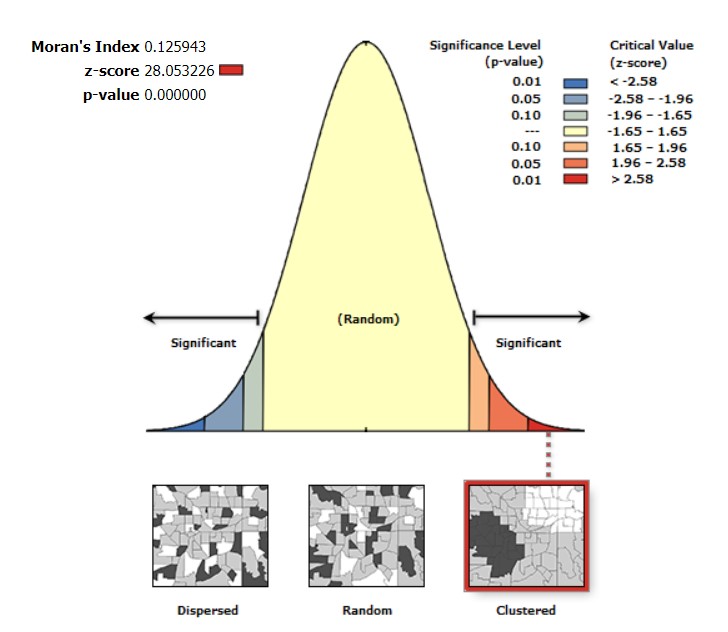

Supplement: Supplementary file 1 — Supplementary Information 1. [file 41598_2023_50933_MOESM1_ESM.zip › July 2021.jpg]

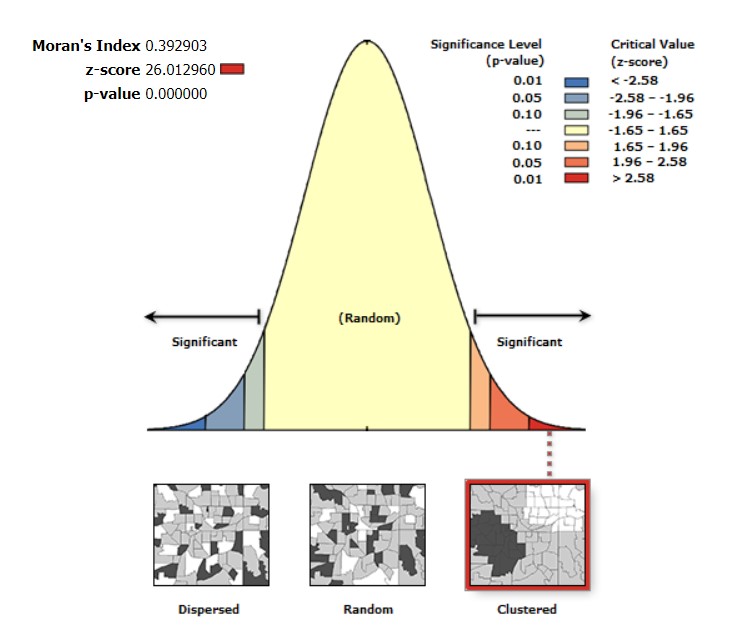

Supplement: Supplementary file 1 — Supplementary Information 1. [file 41598_2023_50933_MOESM1_ESM.zip › July 2022.jpg]

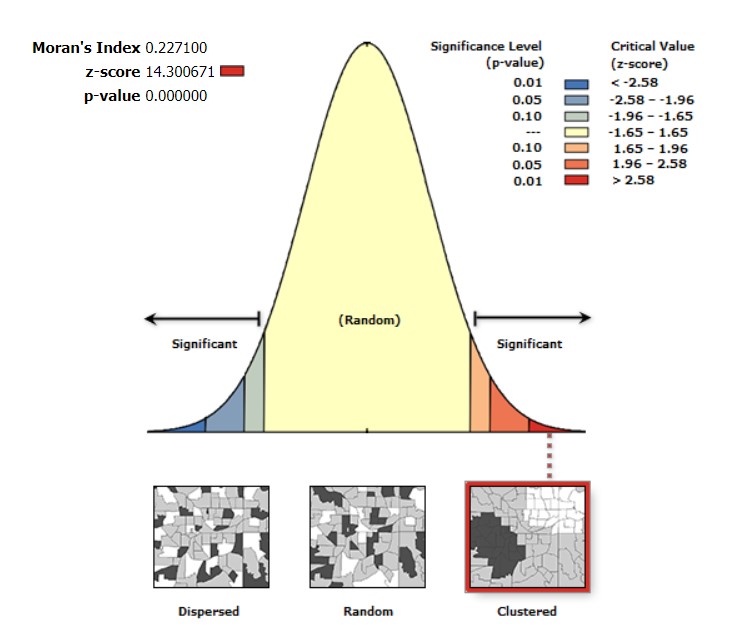

Supplement: Supplementary file 1 — Supplementary Information 1. [file 41598_2023_50933_MOESM1_ESM.zip › June 2020.jpg]

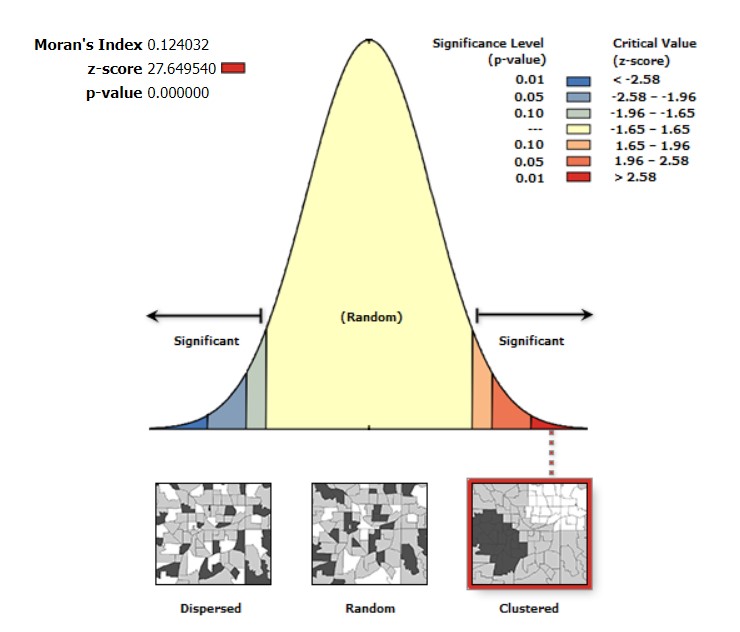

Supplement: Supplementary file 1 — Supplementary Information 1. [file 41598_2023_50933_MOESM1_ESM.zip › June 2021.jpg]

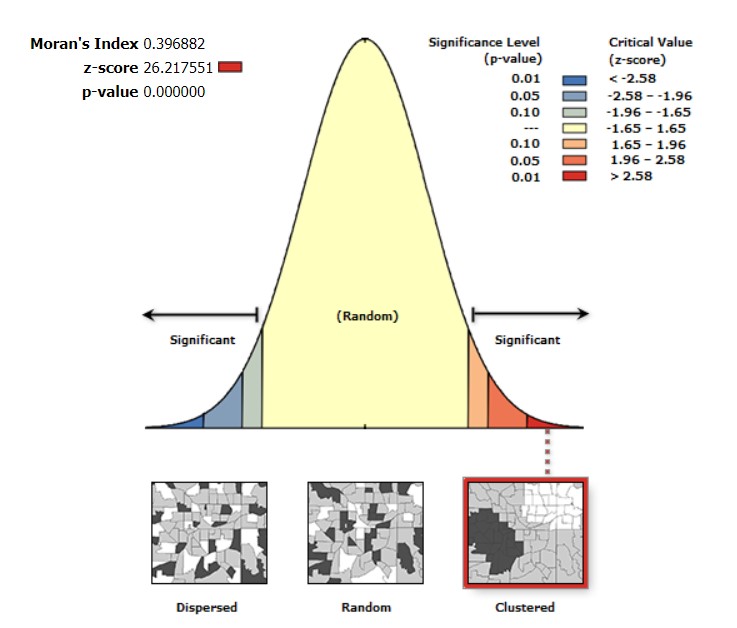

Supplement: Supplementary file 1 — Supplementary Information 1. [file 41598_2023_50933_MOESM1_ESM.zip › June 2022.jpg]

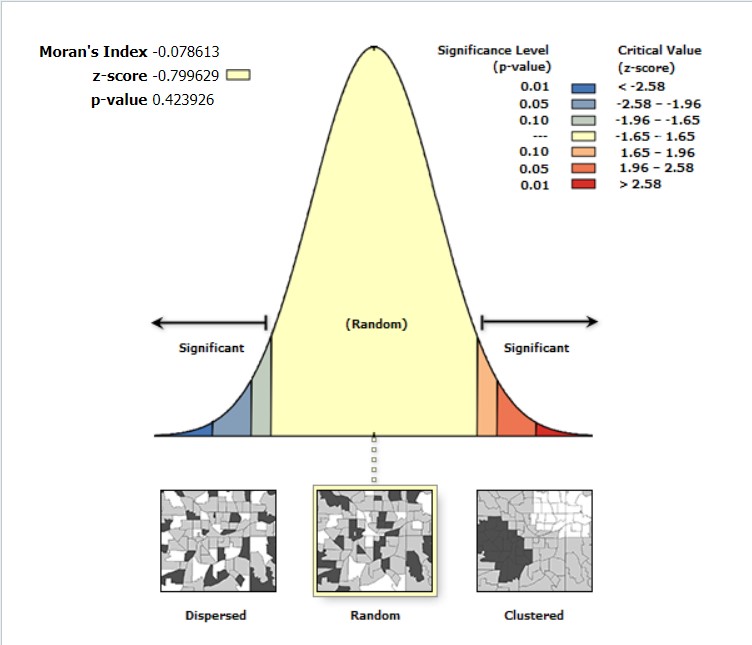

Supplement: Supplementary file 1 — Supplementary Information 1. [file 41598_2023_50933_MOESM1_ESM.zip › March 2020.jpg]

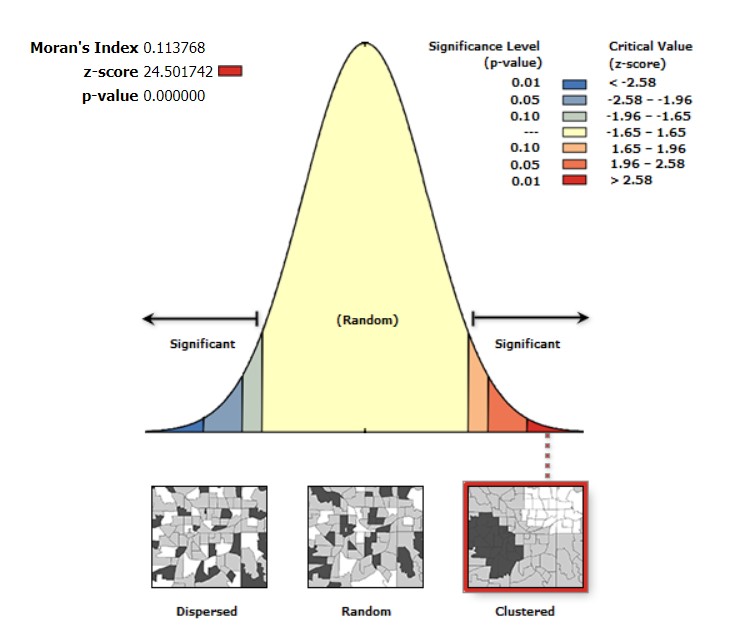

Supplement: Supplementary file 1 — Supplementary Information 1. [file 41598_2023_50933_MOESM1_ESM.zip › March 2021.jpg]

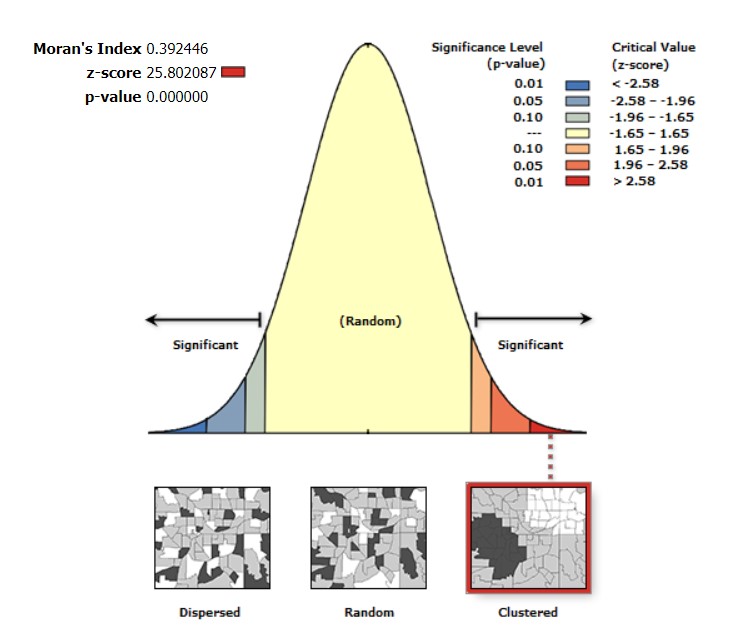

Supplement: Supplementary file 1 — Supplementary Information 1. [file 41598_2023_50933_MOESM1_ESM.zip › March 2022.jpg]

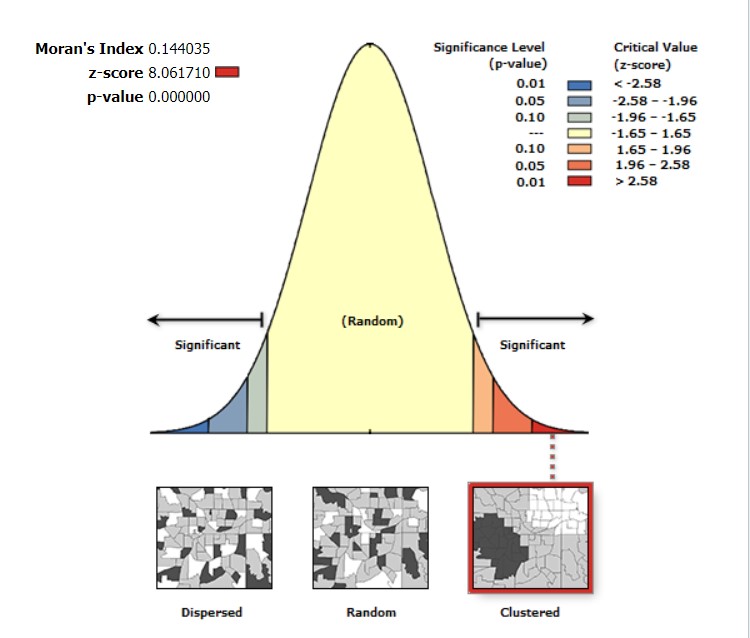

Supplement: Supplementary file 1 — Supplementary Information 1. [file 41598_2023_50933_MOESM1_ESM.zip › May 2020.jpg]

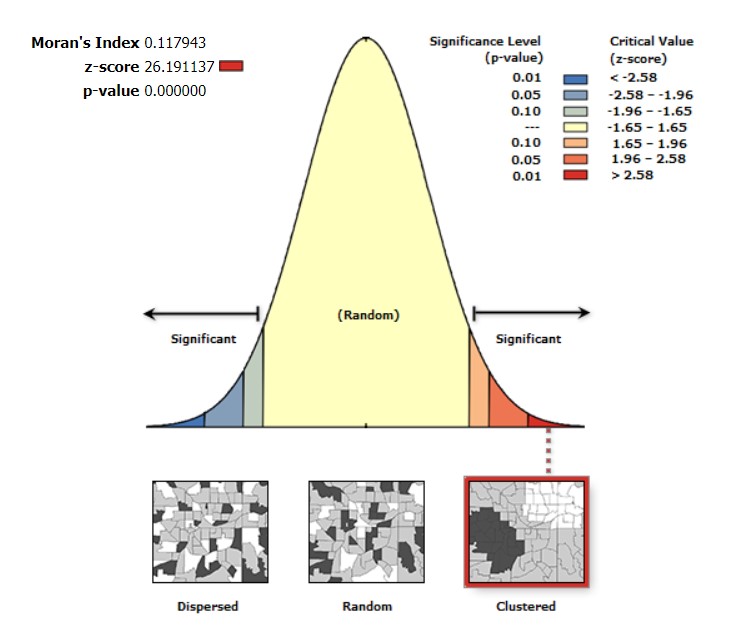

Supplement: Supplementary file 1 — Supplementary Information 1. [file 41598_2023_50933_MOESM1_ESM.zip › May 2021.jpg]

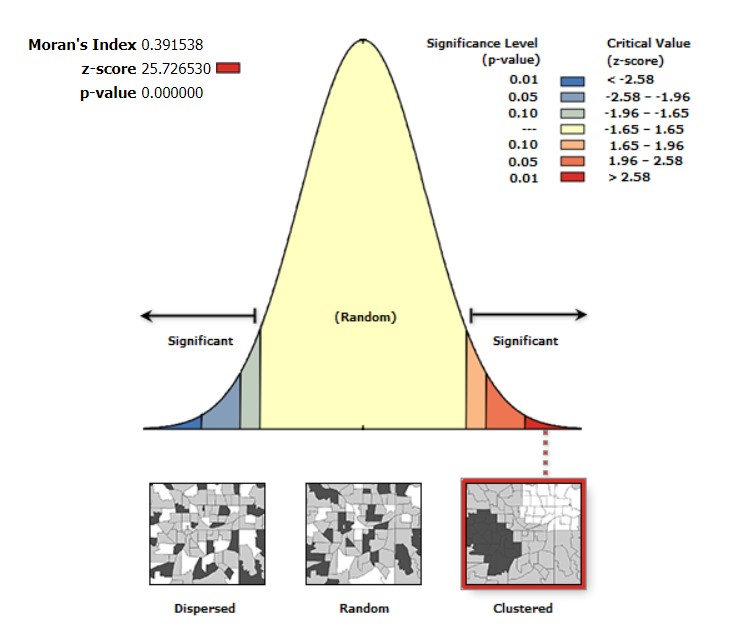

Supplement: Supplementary file 1 — Supplementary Information 1. [file 41598_2023_50933_MOESM1_ESM.zip › May 2022.jpg]

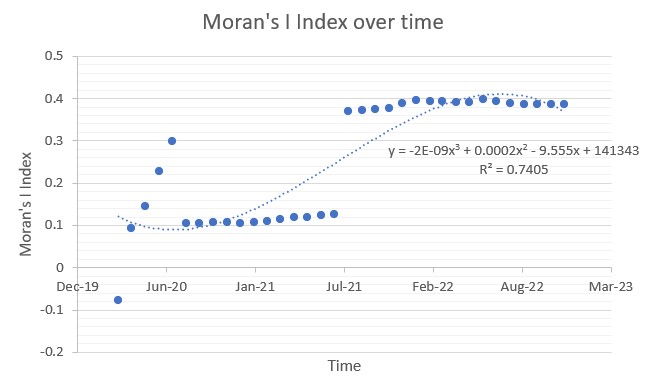

Supplement: Supplementary file 1 — Supplementary Information 1. [file 41598_2023_50933_MOESM1_ESM.zip › Moran's I Index Correlation.jpg]

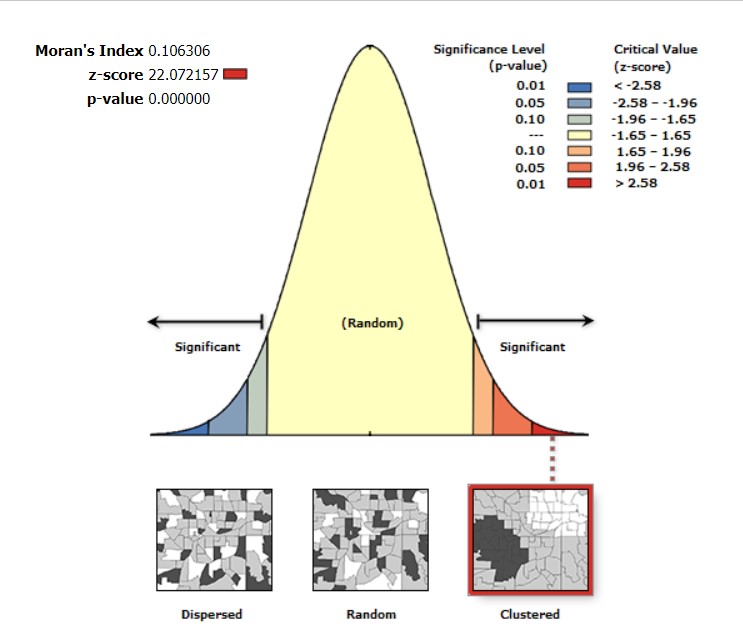

Supplement: Supplementary file 1 — Supplementary Information 1. [file 41598_2023_50933_MOESM1_ESM.zip › November 2020.jpg]

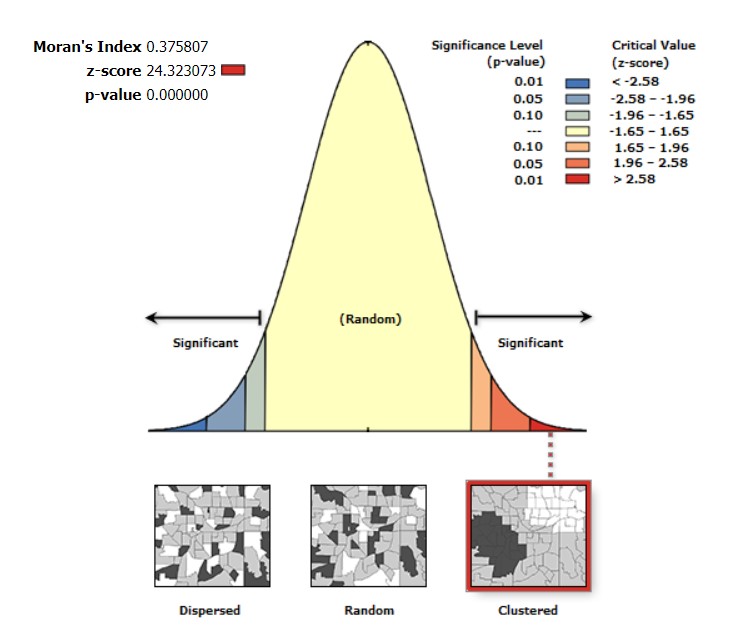

Supplement: Supplementary file 1 — Supplementary Information 1. [file 41598_2023_50933_MOESM1_ESM.zip › November 2021.jpg]

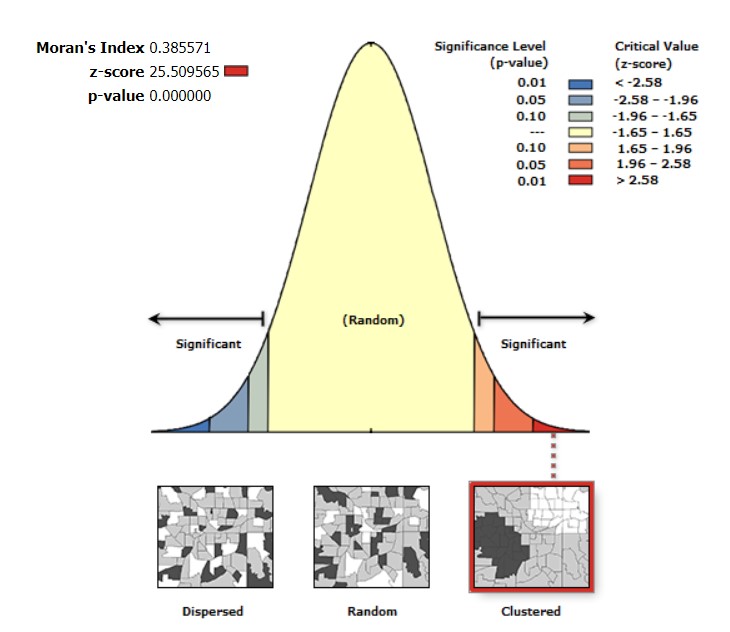

Supplement: Supplementary file 1 — Supplementary Information 1. [file 41598_2023_50933_MOESM1_ESM.zip › November 2022.jpg]

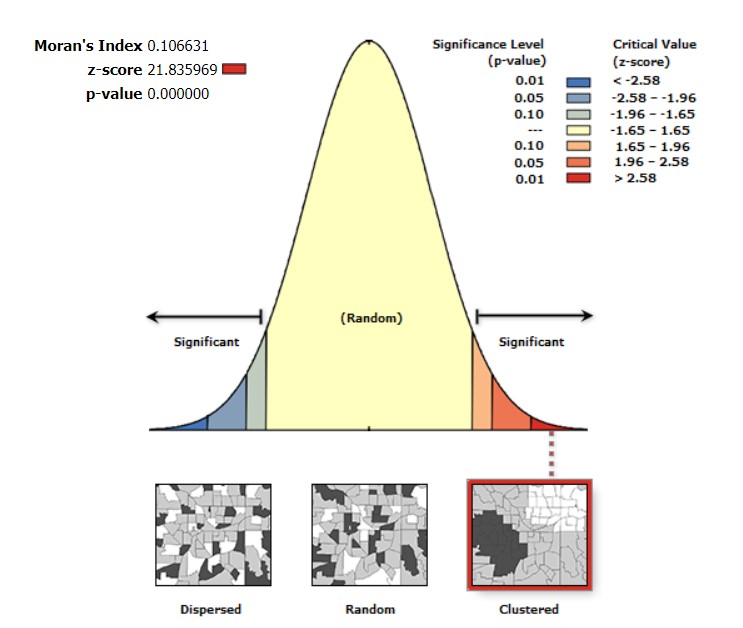

Supplement: Supplementary file 1 — Supplementary Information 1. [file 41598_2023_50933_MOESM1_ESM.zip › October 2020.jpg]

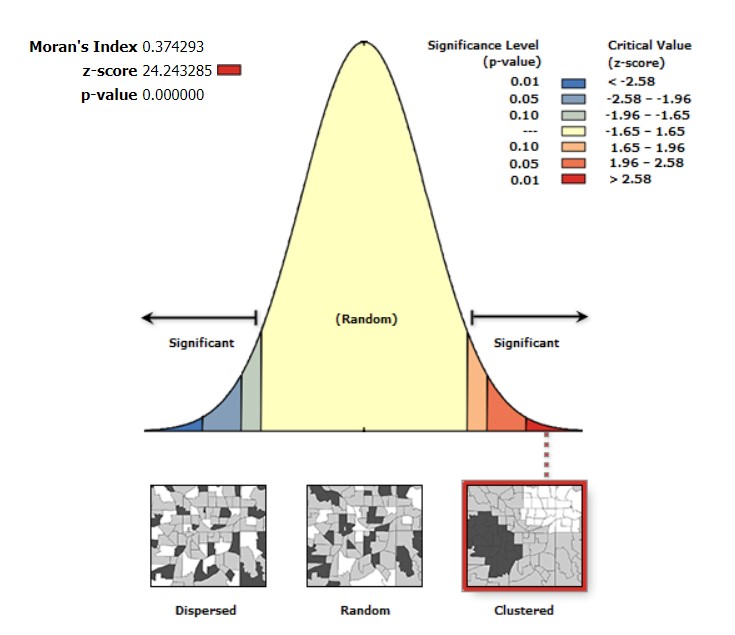

Supplement: Supplementary file 1 — Supplementary Information 1. [file 41598_2023_50933_MOESM1_ESM.zip › October 2021.jpg]

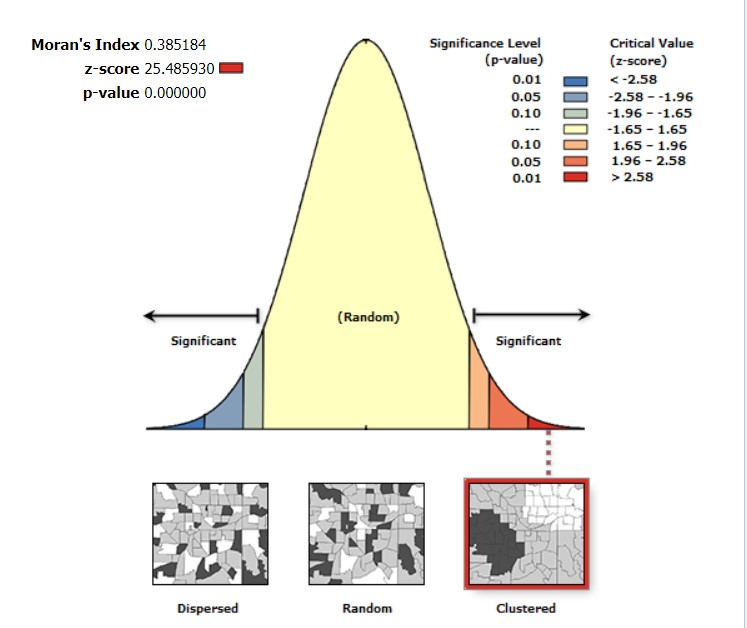

Supplement: Supplementary file 1 — Supplementary Information 1. [file 41598_2023_50933_MOESM1_ESM.zip › October 2022.jpg]

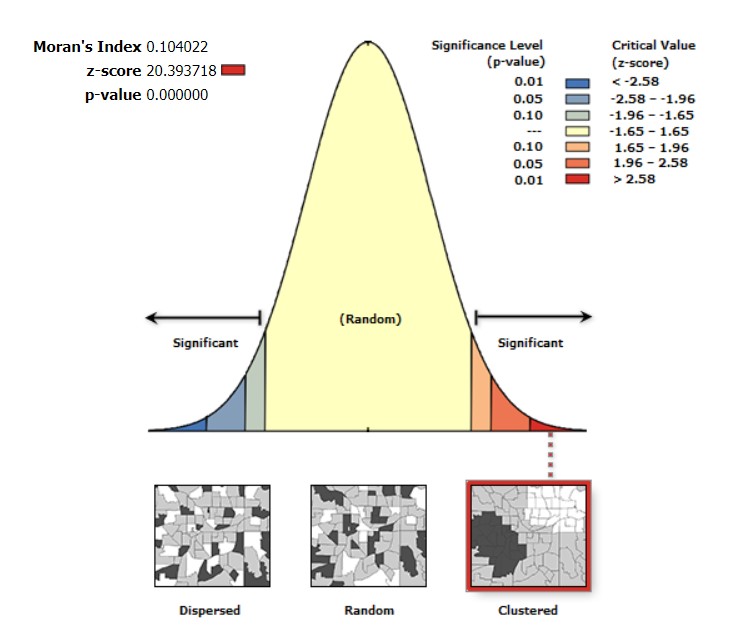

Supplement: Supplementary file 1 — Supplementary Information 1. [file 41598_2023_50933_MOESM1_ESM.zip › September 2020.jpg]

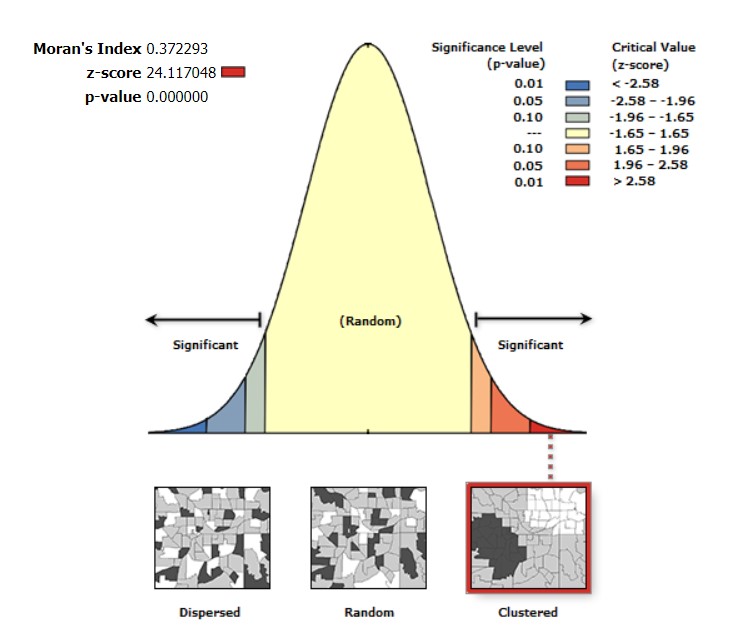

Supplement: Supplementary file 1 — Supplementary Information 1. [file 41598_2023_50933_MOESM1_ESM.zip › September 2021.jpg]

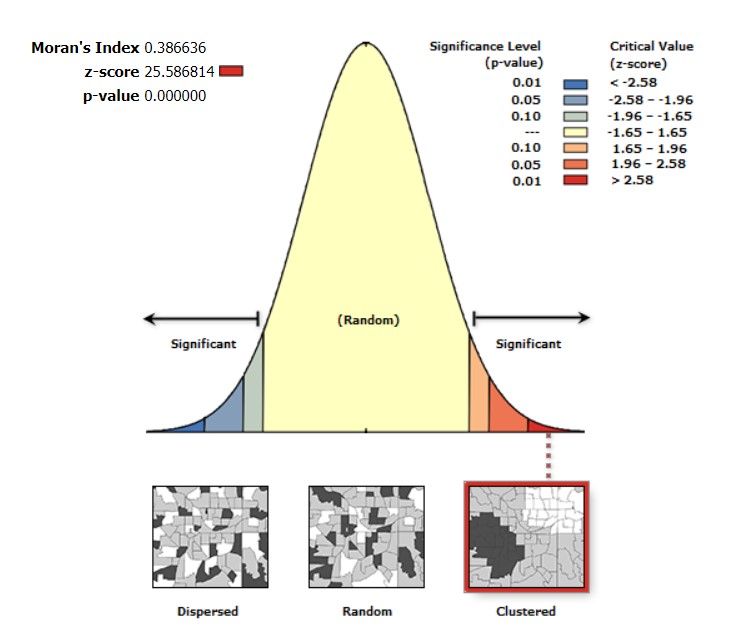

Supplement: Supplementary file 1 — Supplementary Information 1. [file 41598_2023_50933_MOESM1_ESM.zip › September 2022.jpg]

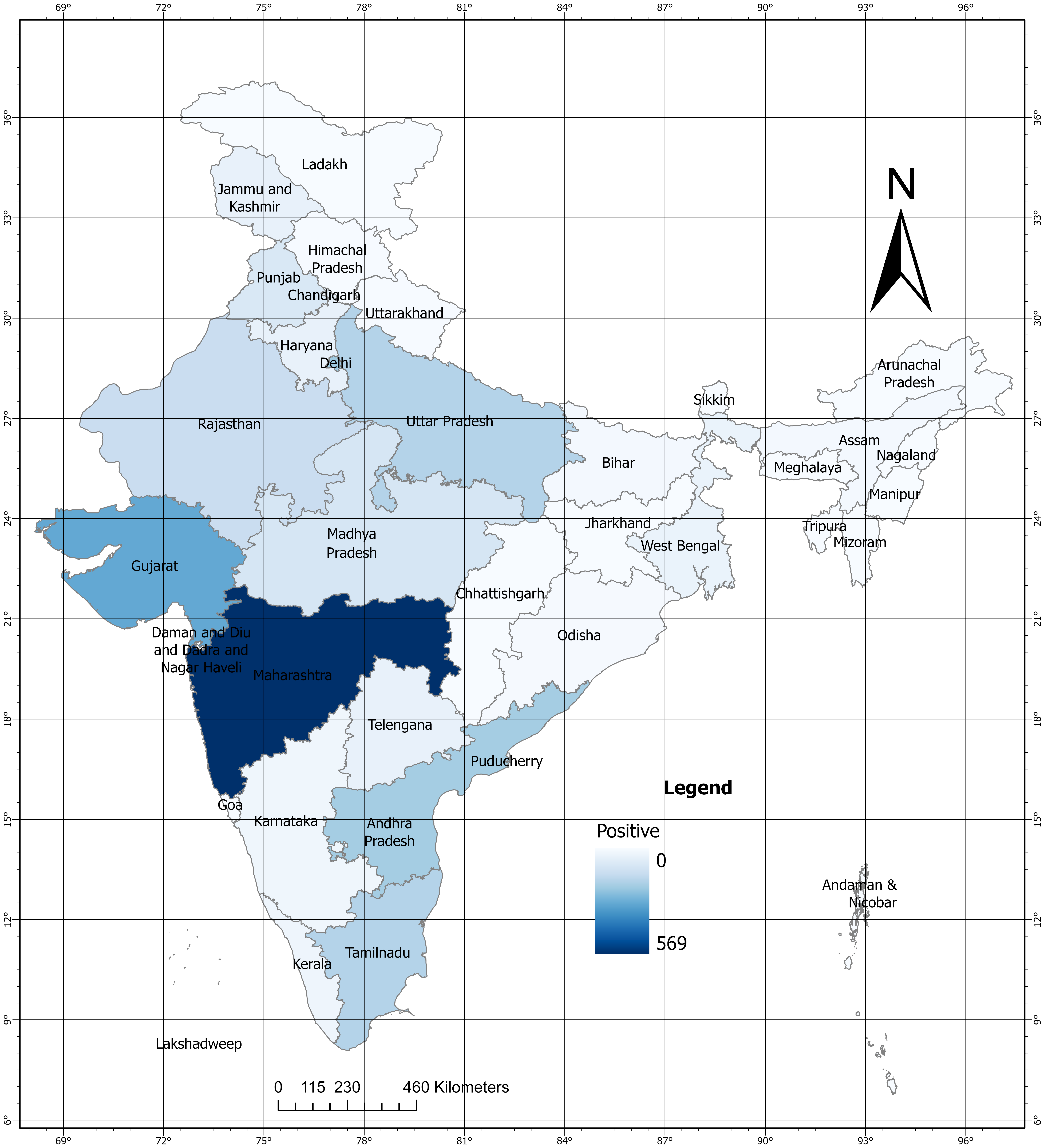

Supplement: Supplementary file 2 — Supplementary Information 2. [file 41598_2023_50933_MOESM2_ESM.zip › April 2020.png]

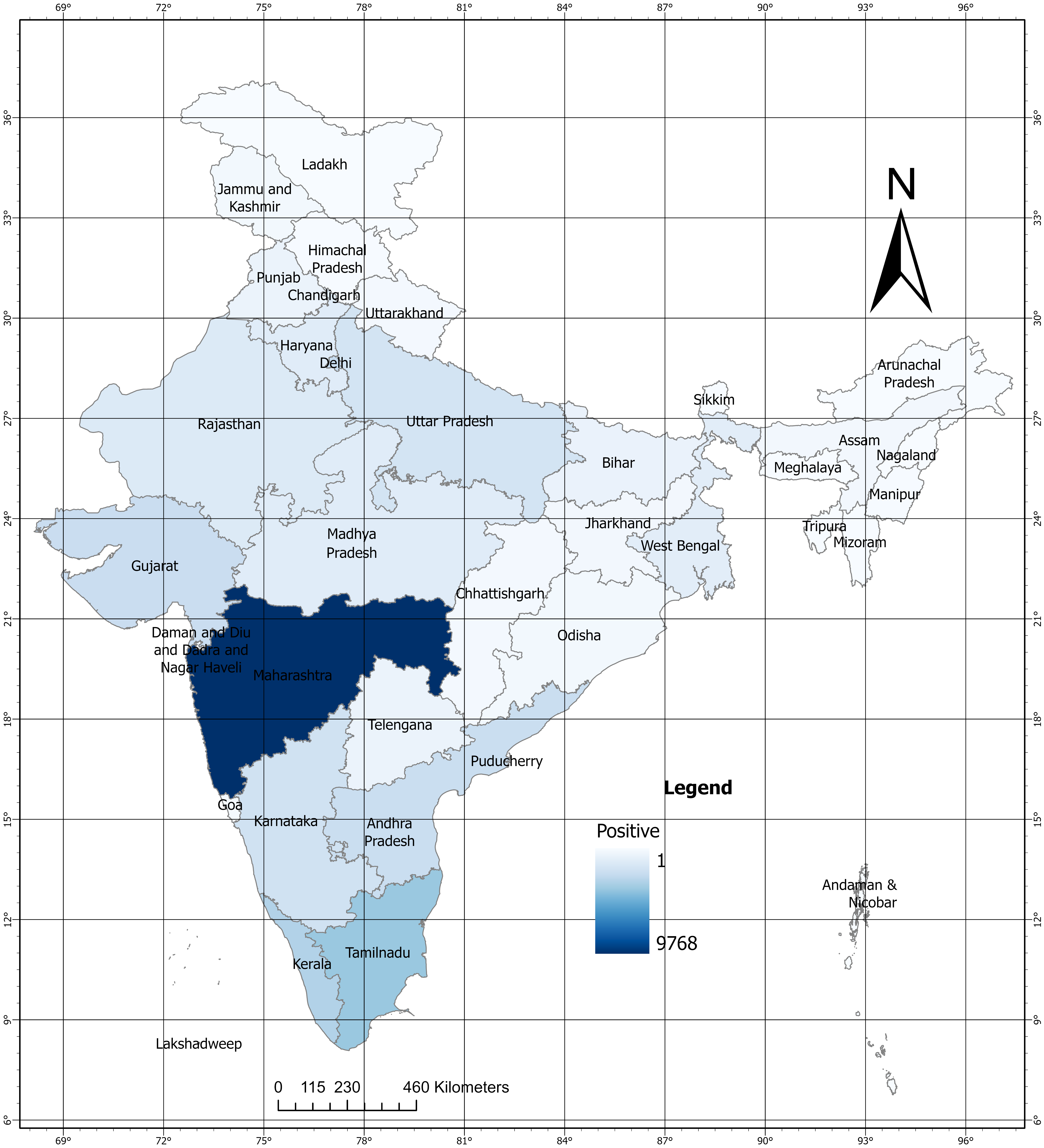

Supplement: Supplementary file 2 — Supplementary Information 2. [file 41598_2023_50933_MOESM2_ESM.zip › April 2021.png]

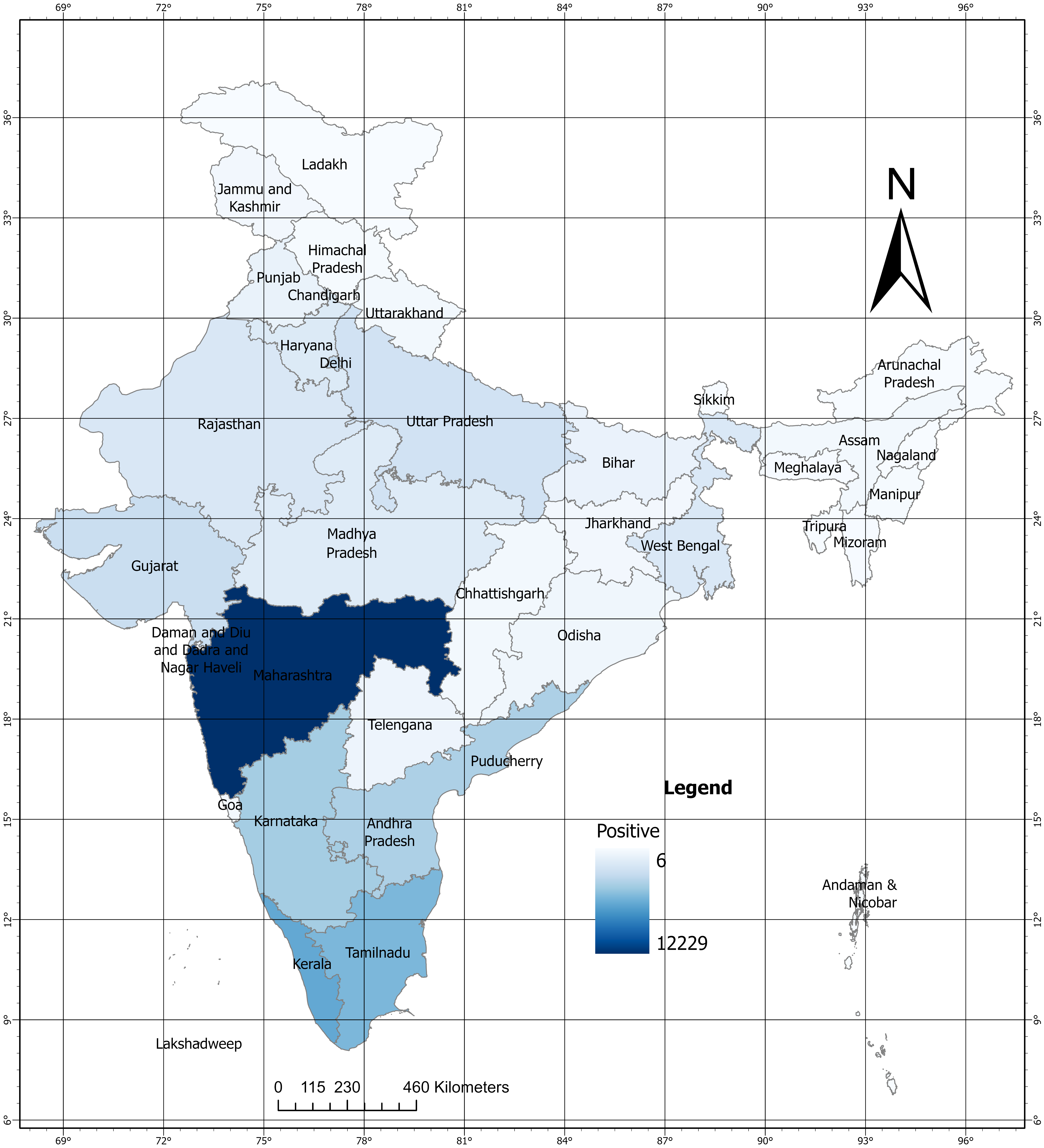

Supplement: Supplementary file 2 — Supplementary Information 2. [file 41598_2023_50933_MOESM2_ESM.zip › April 2022.png]

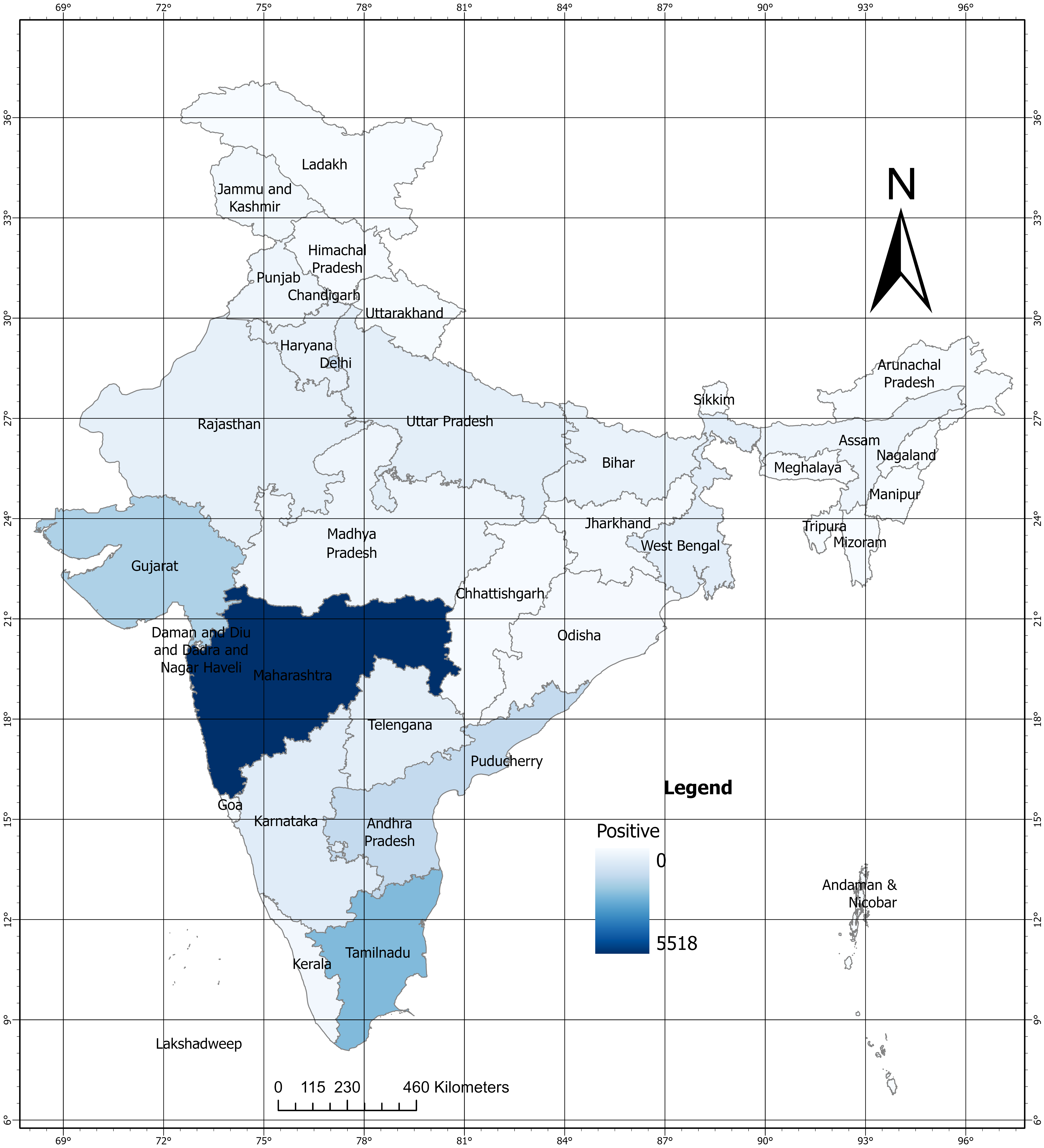

Supplement: Supplementary file 2 — Supplementary Information 2. [file 41598_2023_50933_MOESM2_ESM.zip › Aug 2020.png]

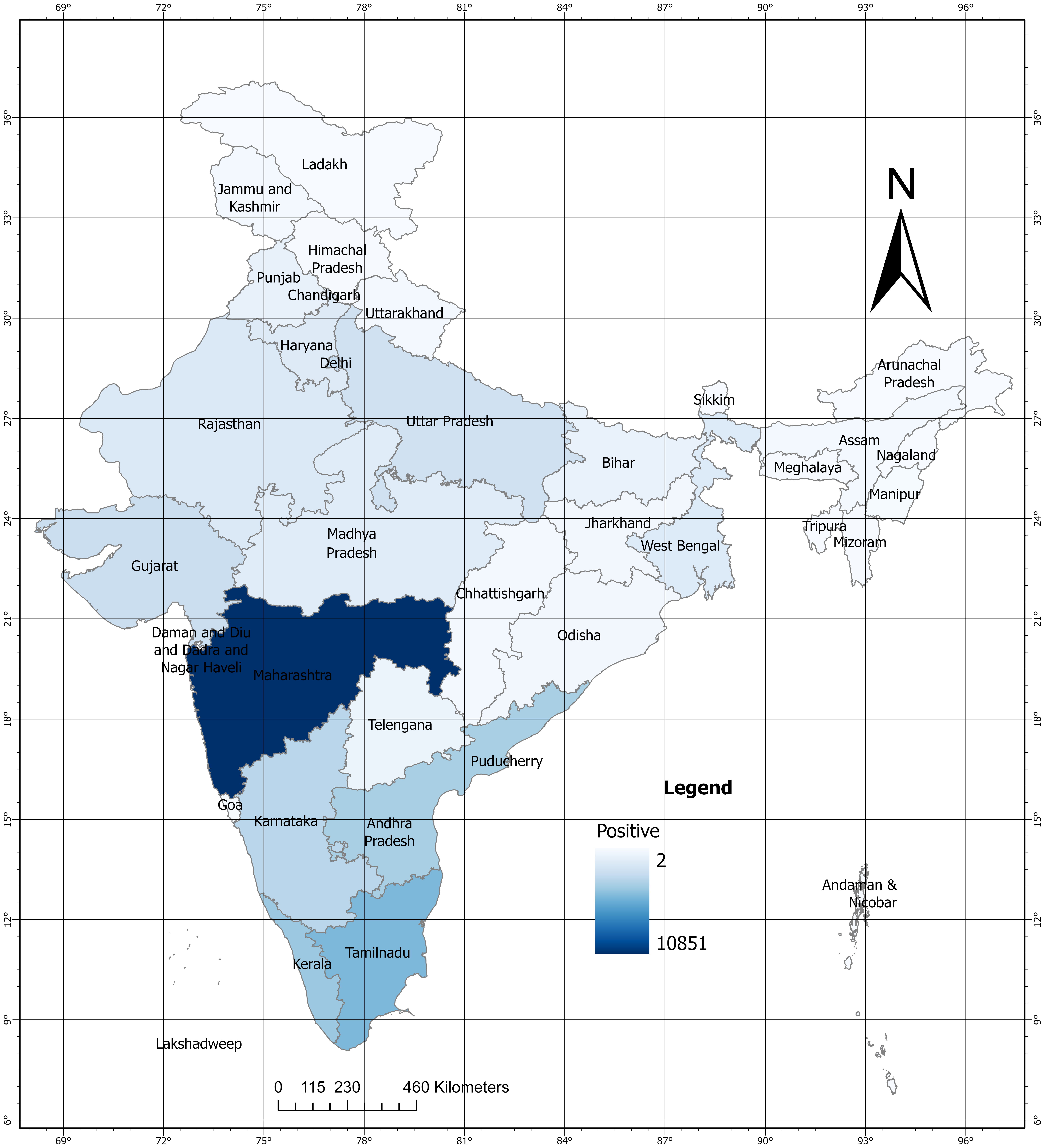

Supplement: Supplementary file 2 — Supplementary Information 2. [file 41598_2023_50933_MOESM2_ESM.zip › Aug 2021.png]

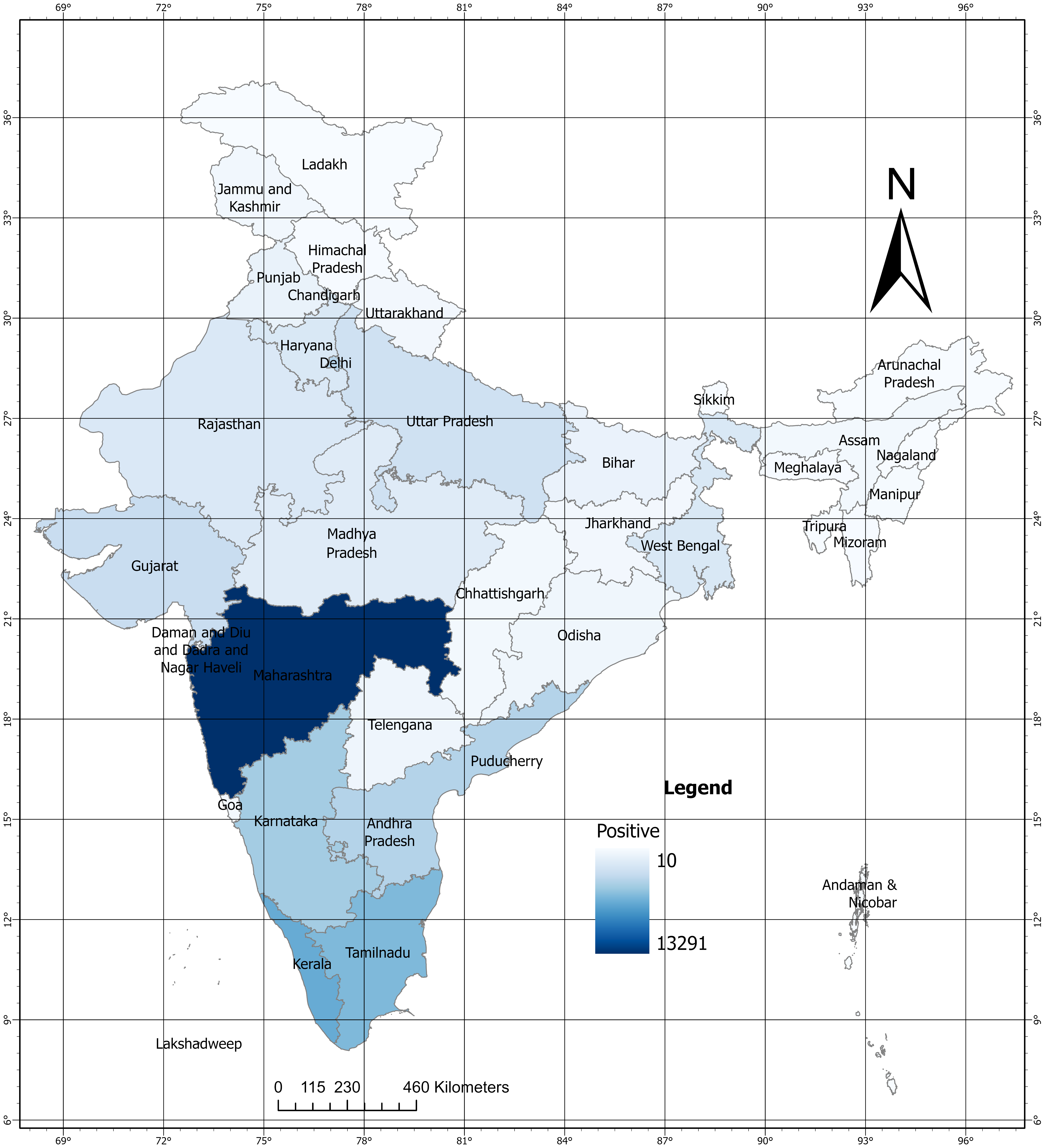

Supplement: Supplementary file 2 — Supplementary Information 2. [file 41598_2023_50933_MOESM2_ESM.zip › Aug 2022.png]

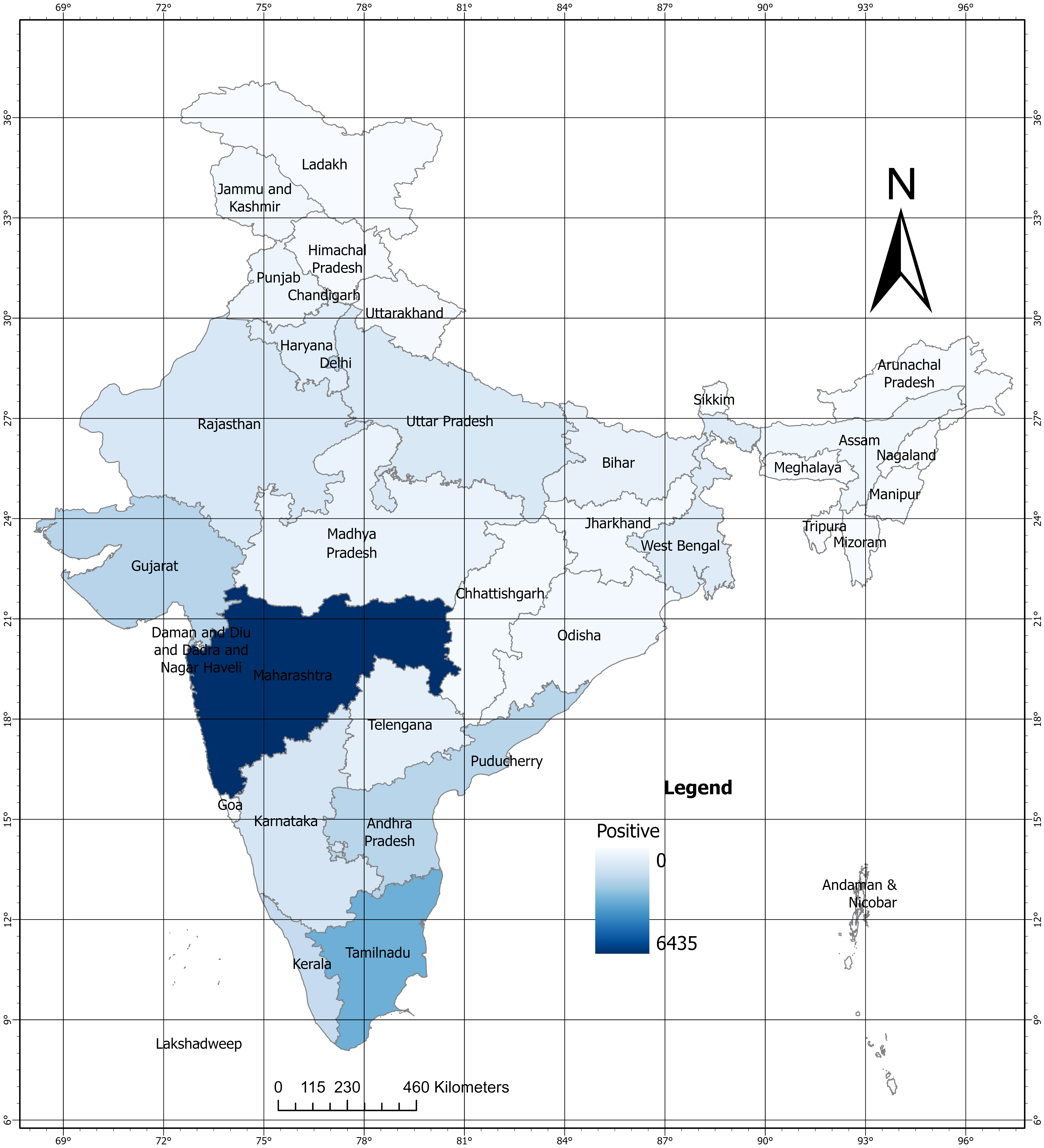

Supplement: Supplementary file 2 — Supplementary Information 2. [file 41598_2023_50933_MOESM2_ESM.zip › Dec 2020.png]

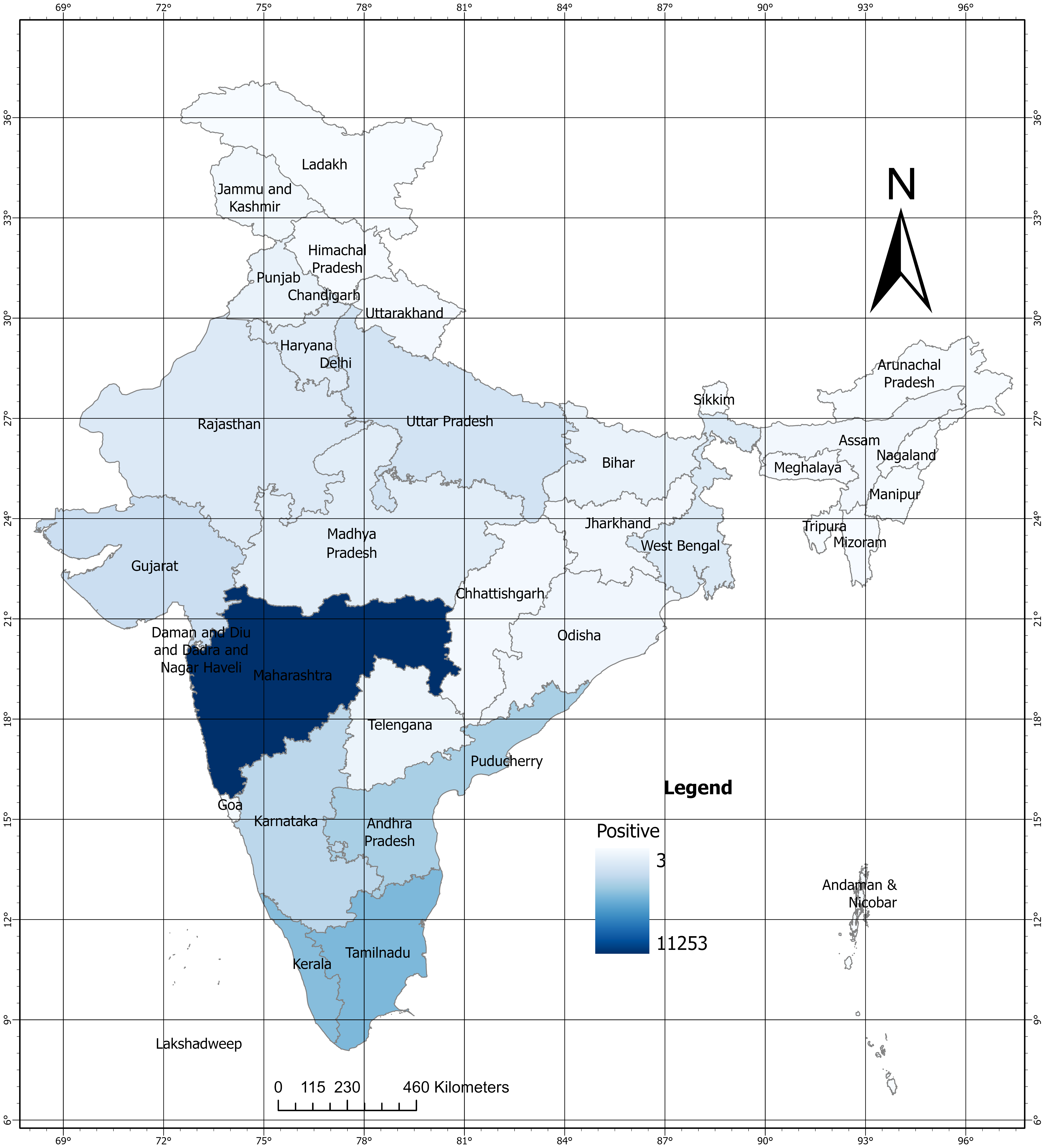

Supplement: Supplementary file 2 — Supplementary Information 2. [file 41598_2023_50933_MOESM2_ESM.zip › Dec 2021.png]

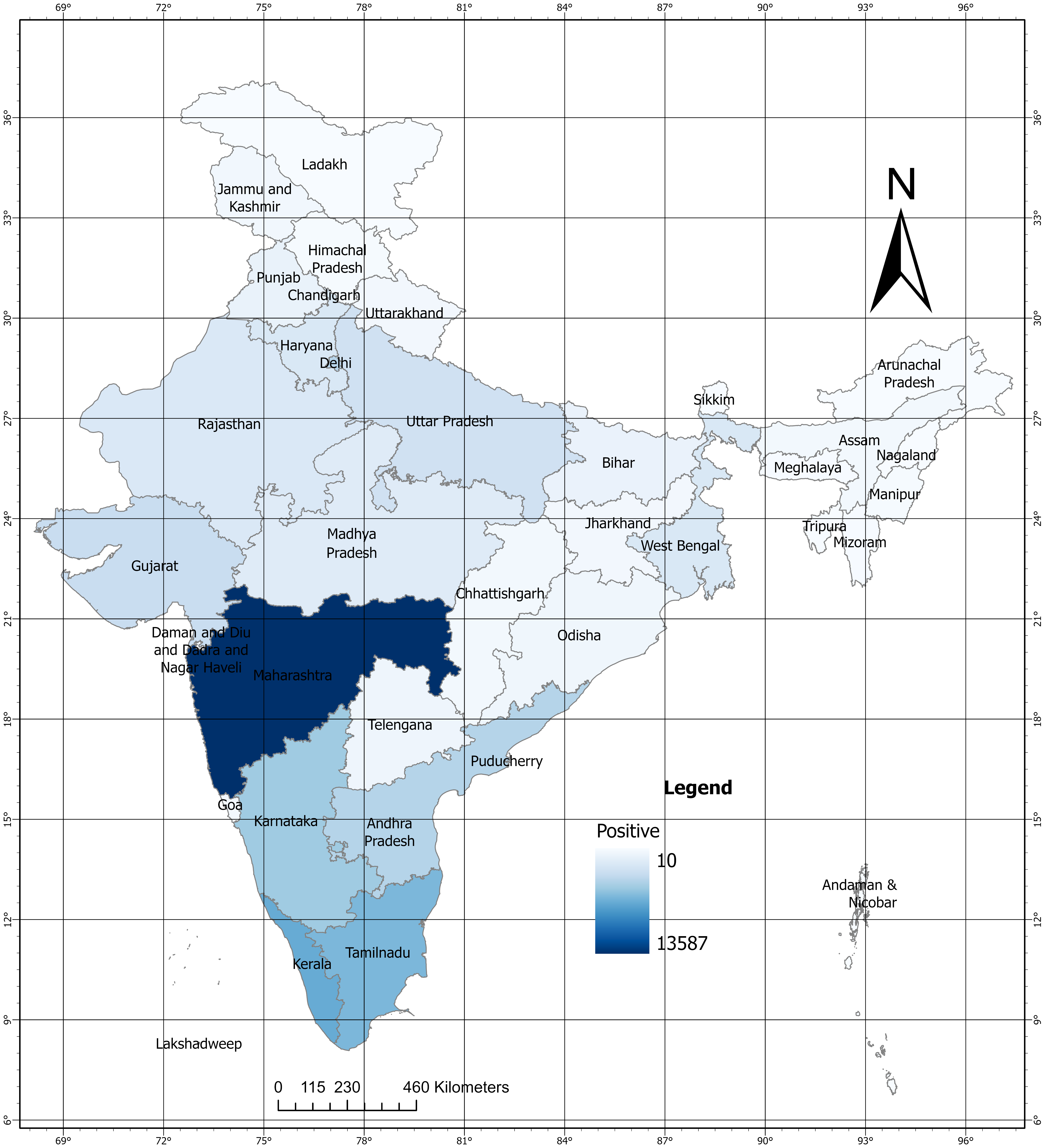

Supplement: Supplementary file 2 — Supplementary Information 2. [file 41598_2023_50933_MOESM2_ESM.zip › Dec 2022.png]

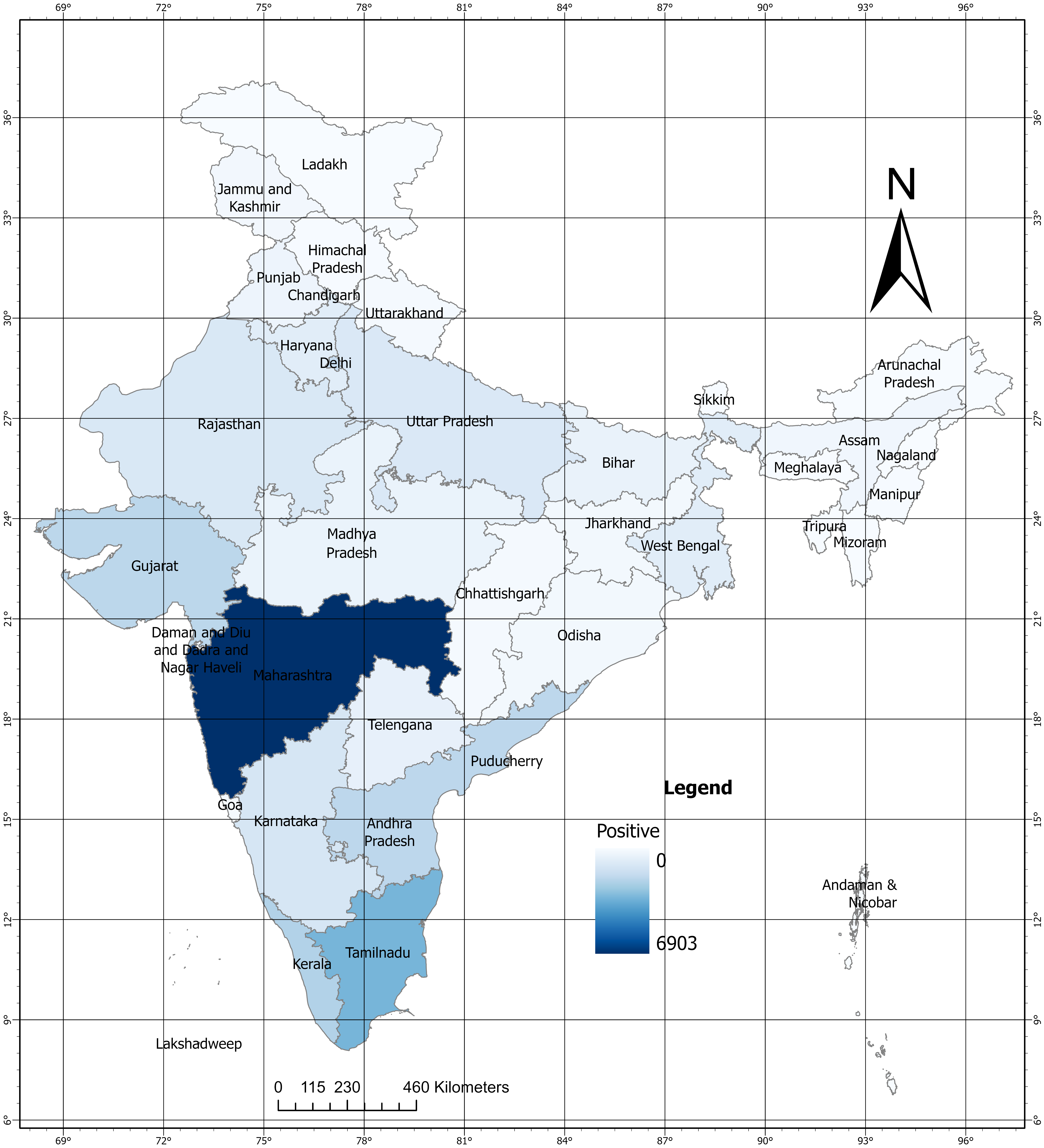

Supplement: Supplementary file 2 — Supplementary Information 2. [file 41598_2023_50933_MOESM2_ESM.zip › Feb 2021.png]

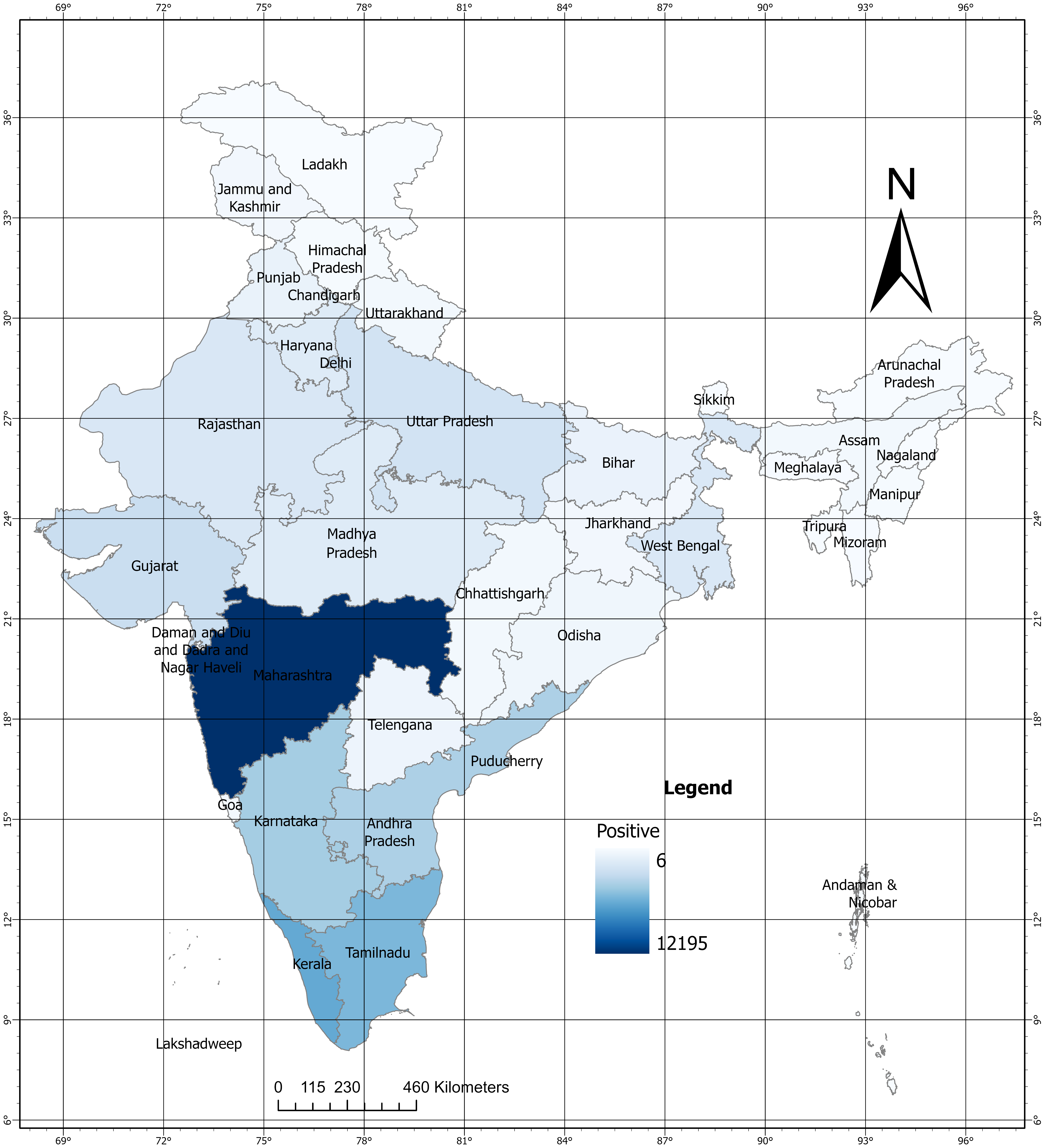

Supplement: Supplementary file 2 — Supplementary Information 2. [file 41598_2023_50933_MOESM2_ESM.zip › Feb 2022.png]

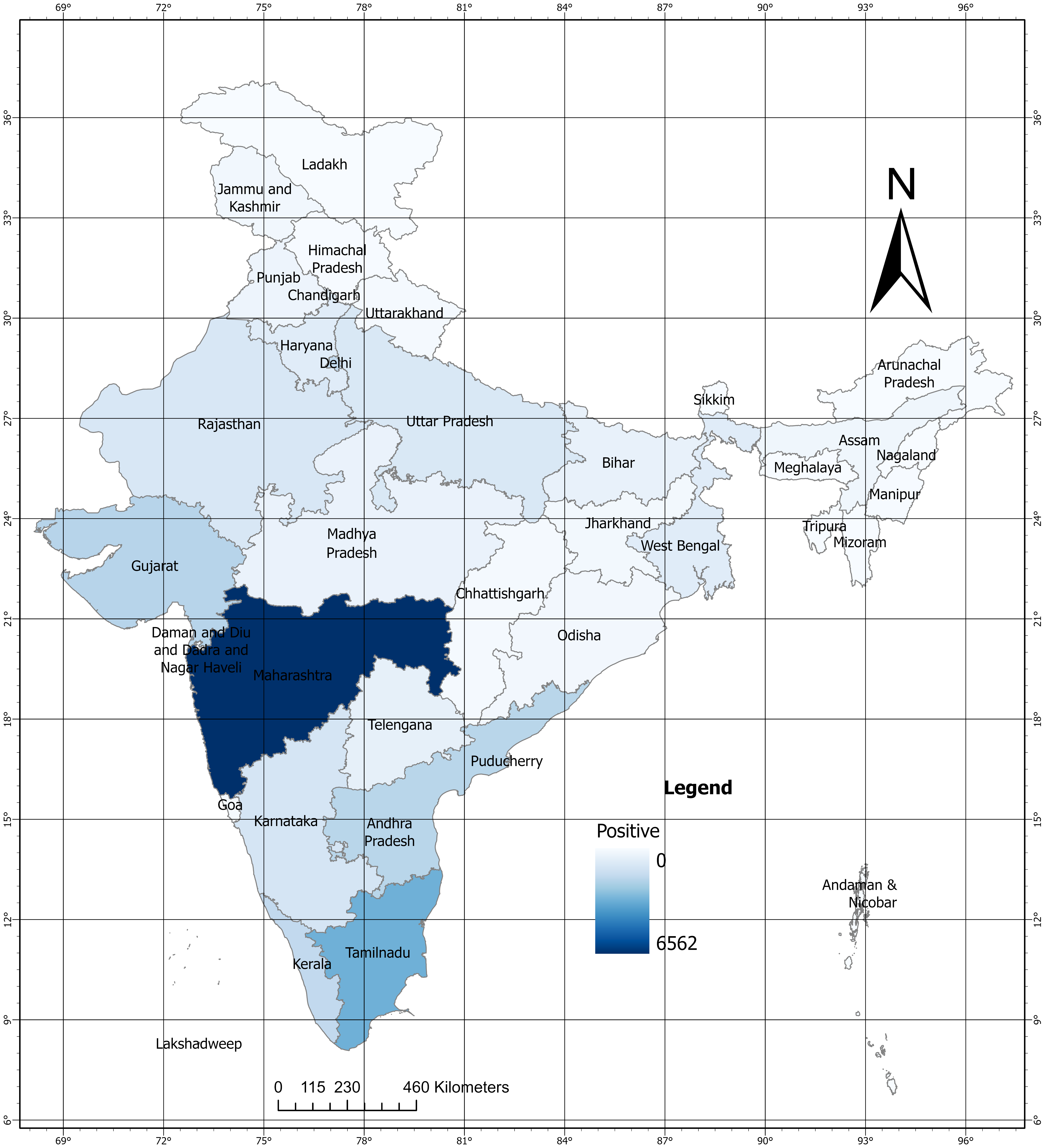

Supplement: Supplementary file 2 — Supplementary Information 2. [file 41598_2023_50933_MOESM2_ESM.zip › Jan 2021.png]

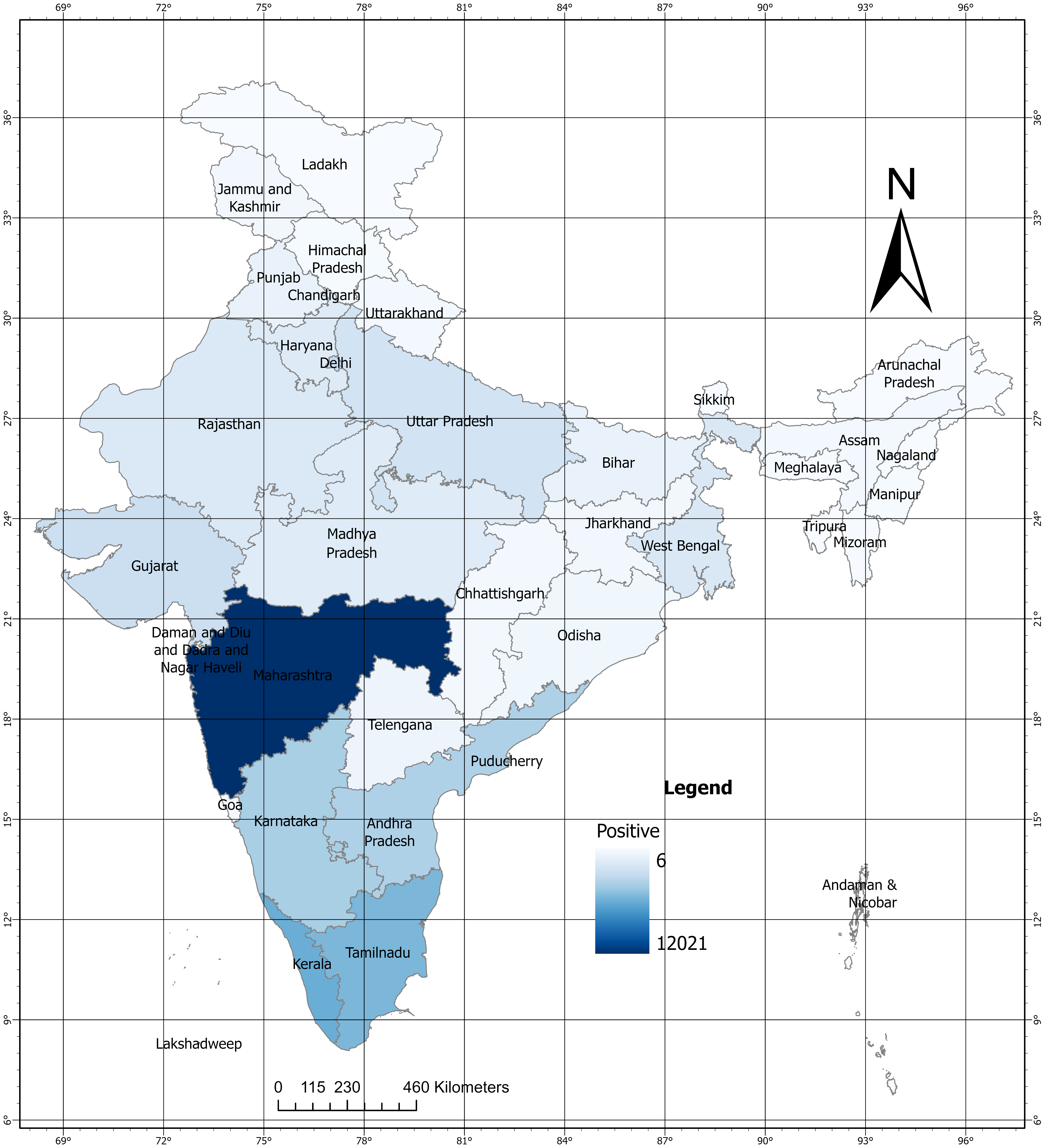

Supplement: Supplementary file 2 — Supplementary Information 2. [file 41598_2023_50933_MOESM2_ESM.zip › Jan 2022.png]

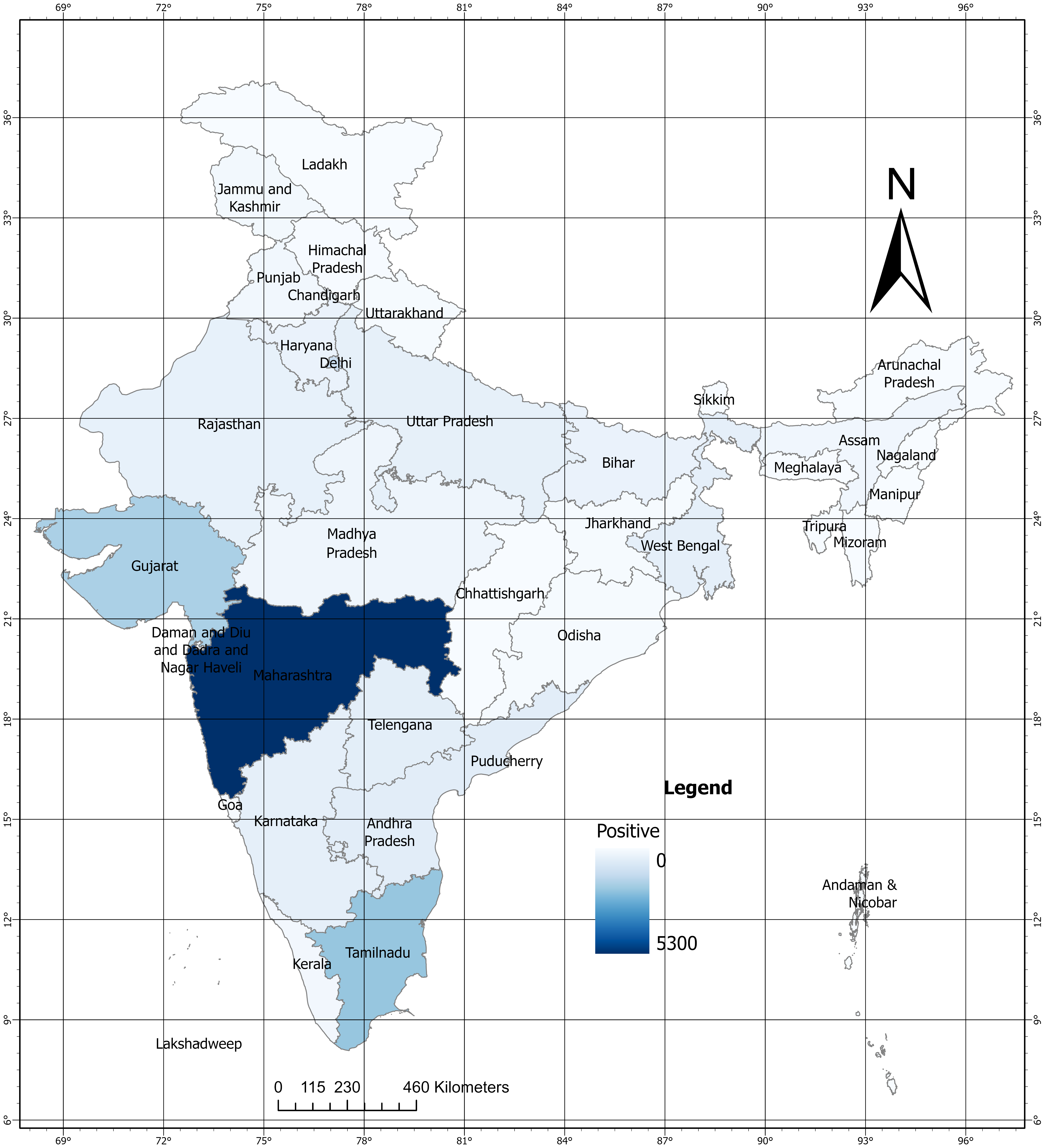

Supplement: Supplementary file 2 — Supplementary Information 2. [file 41598_2023_50933_MOESM2_ESM.zip › July 2020.png]

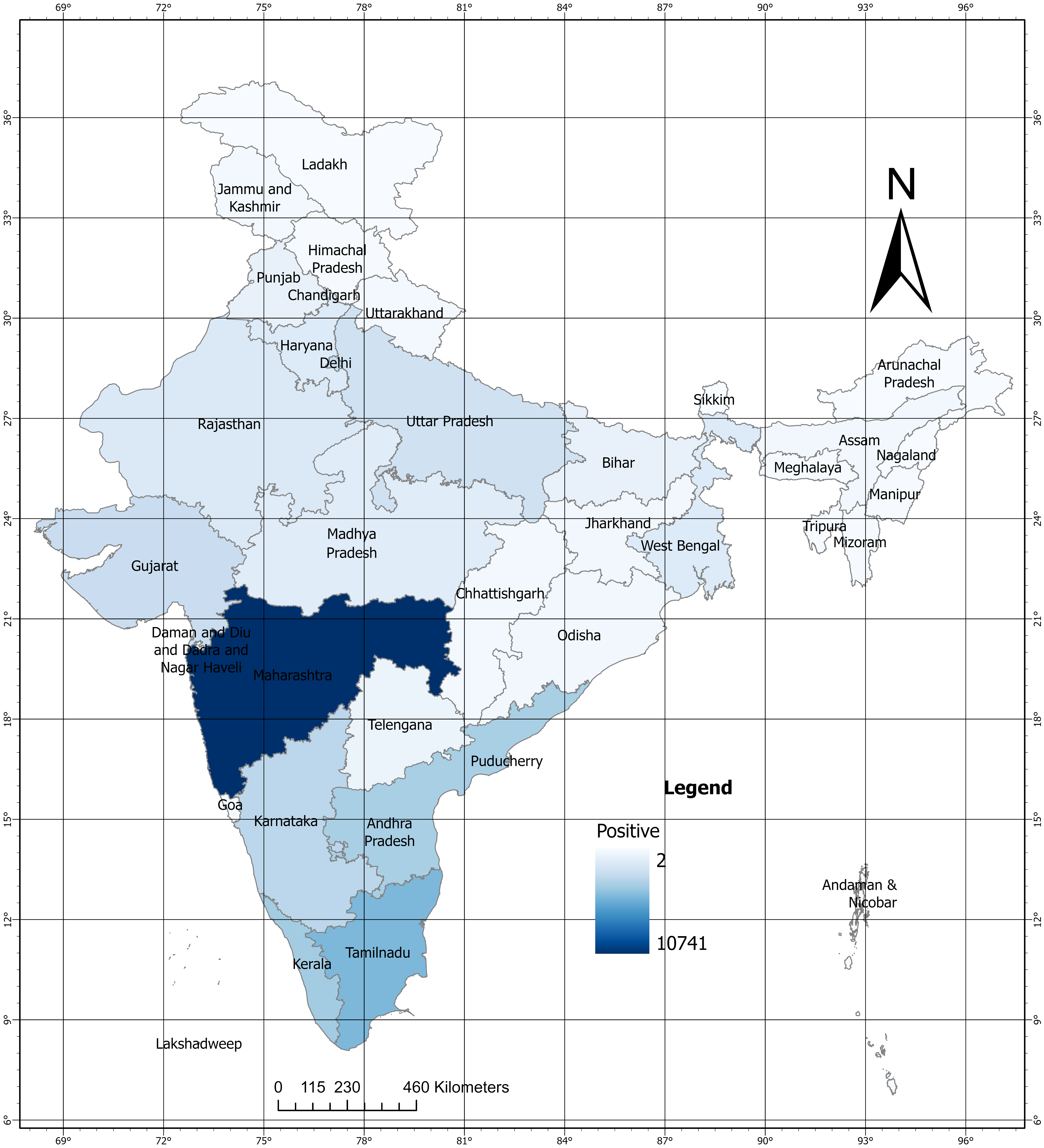

Supplement: Supplementary file 2 — Supplementary Information 2. [file 41598_2023_50933_MOESM2_ESM.zip › July 2021.png]

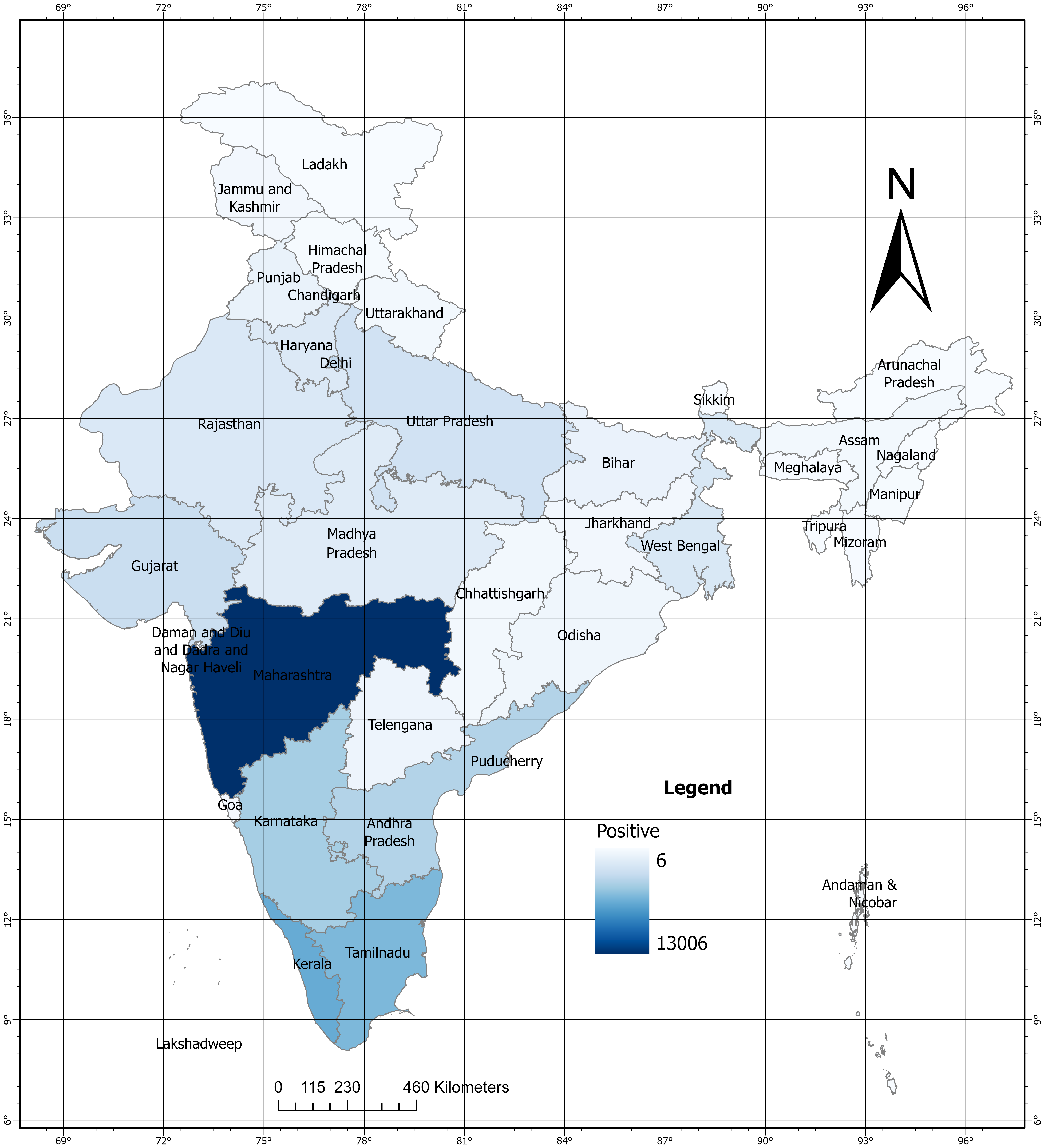

Supplement: Supplementary file 2 — Supplementary Information 2. [file 41598_2023_50933_MOESM2_ESM.zip › July 2022.png]

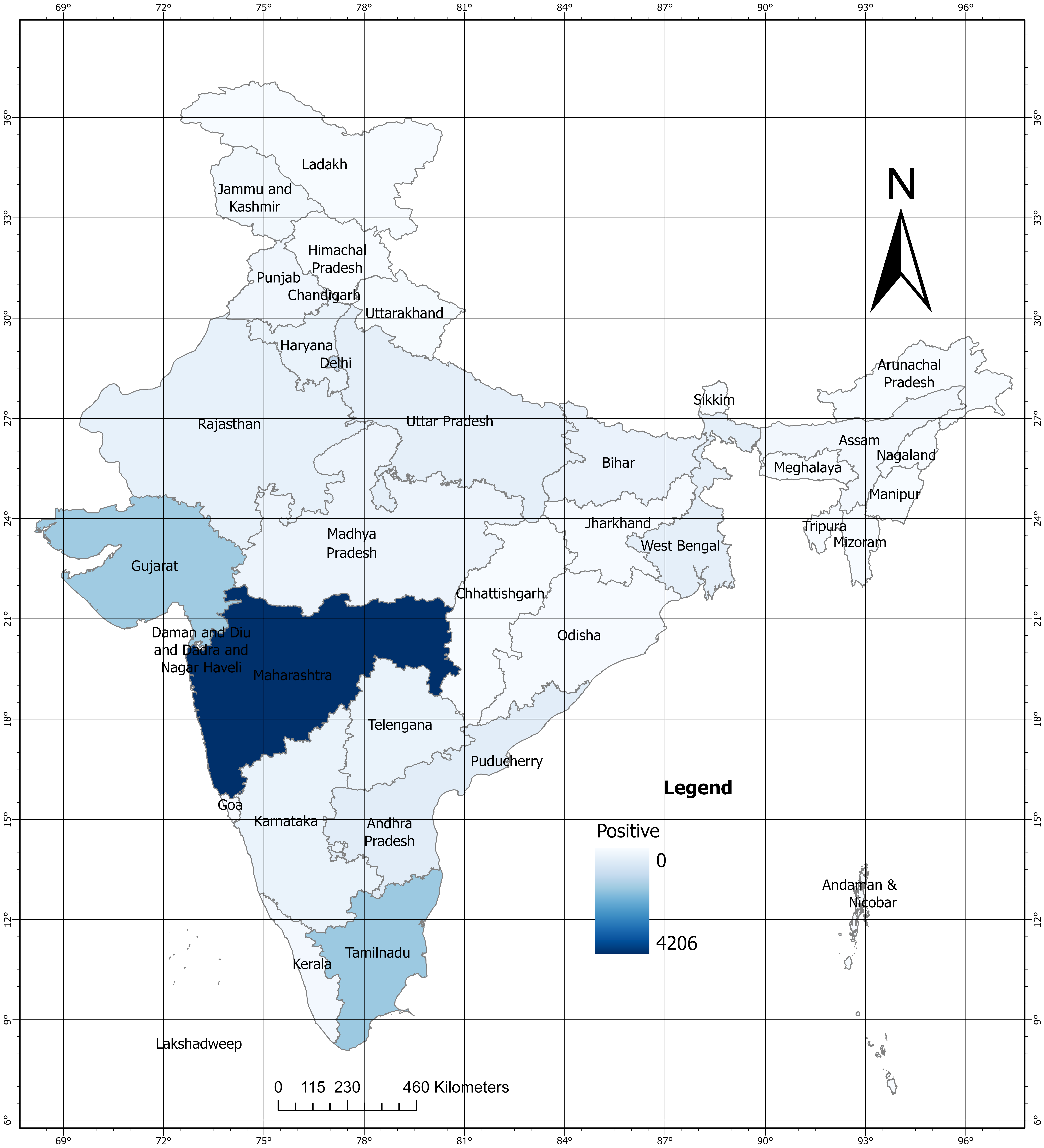

Supplement: Supplementary file 2 — Supplementary Information 2. [file 41598_2023_50933_MOESM2_ESM.zip › June 2020.png]

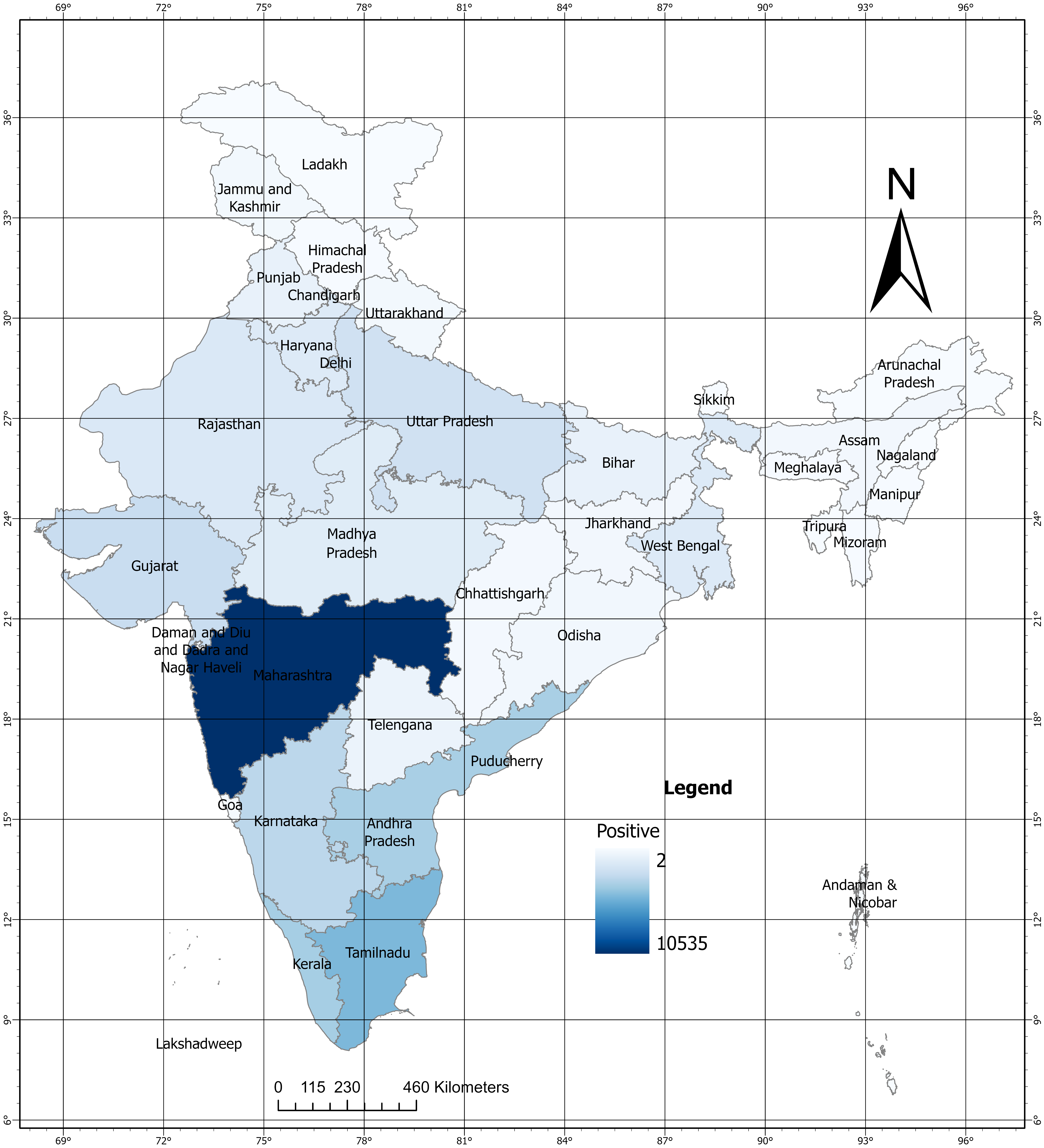

Supplement: Supplementary file 2 — Supplementary Information 2. [file 41598_2023_50933_MOESM2_ESM.zip › June 2021.png]

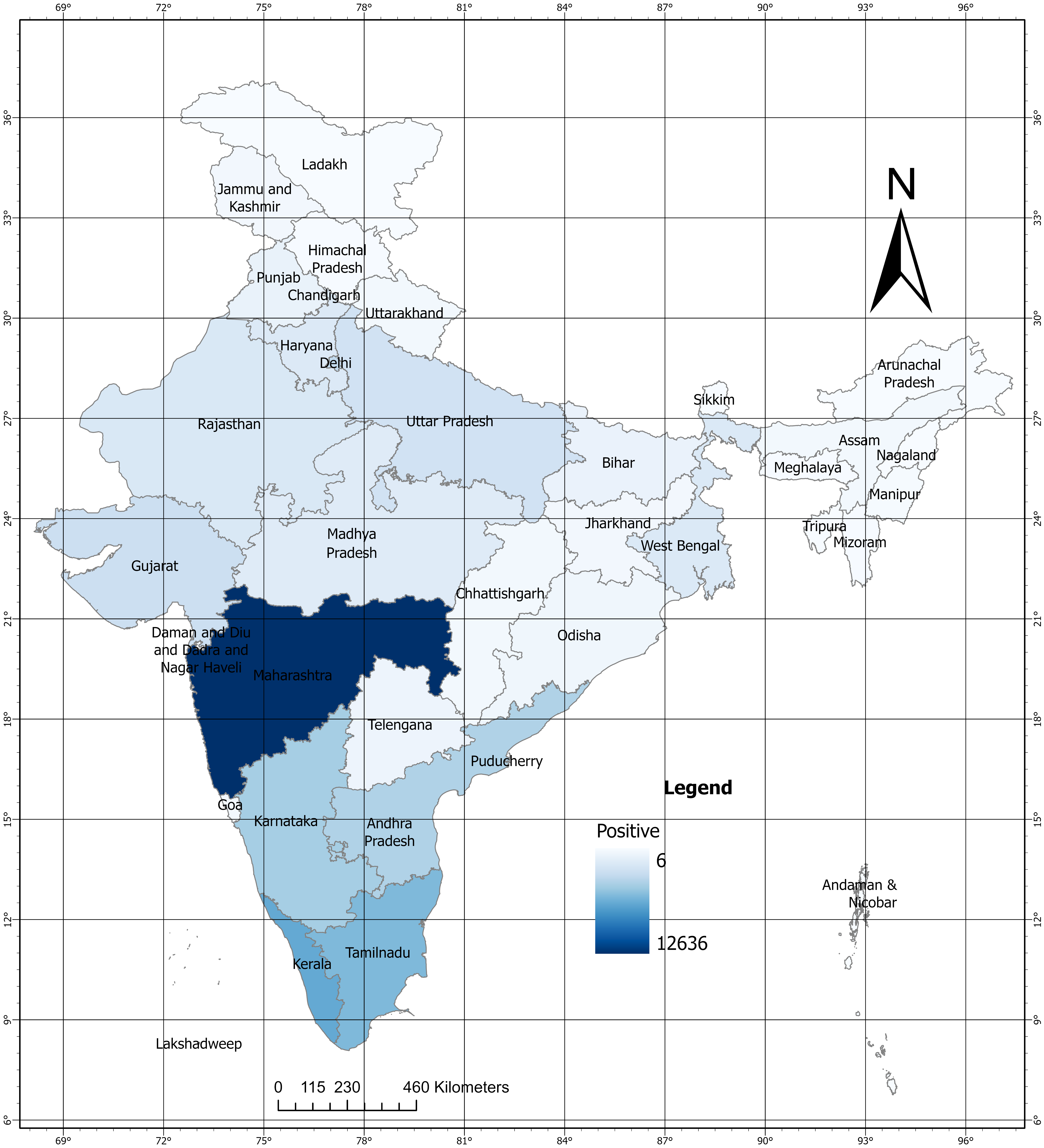

Supplement: Supplementary file 2 — Supplementary Information 2. [file 41598_2023_50933_MOESM2_ESM.zip › June 2022.png]

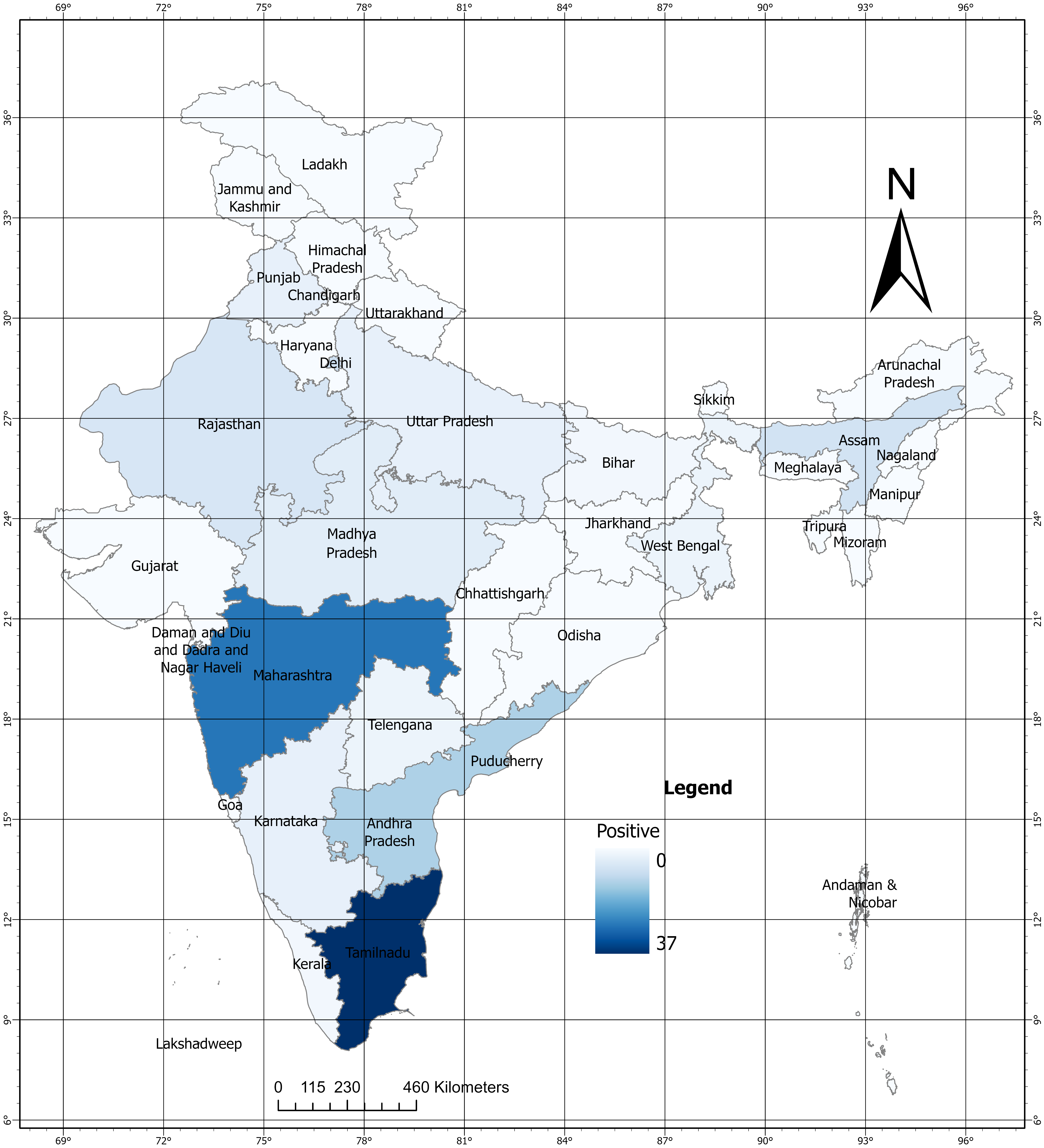

Supplement: Supplementary file 2 — Supplementary Information 2. [file 41598_2023_50933_MOESM2_ESM.zip › March 2020.png]

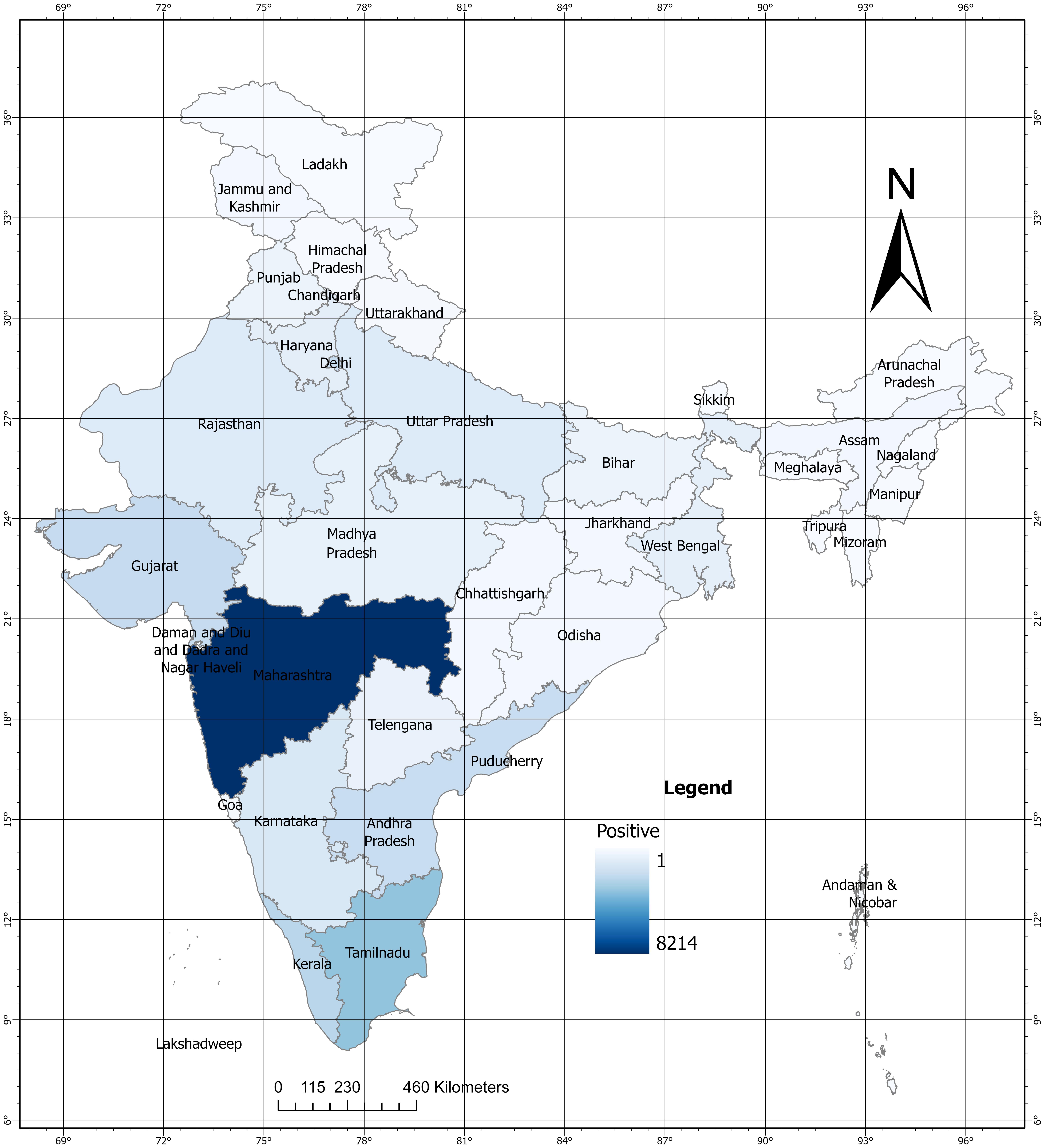

Supplement: Supplementary file 2 — Supplementary Information 2. [file 41598_2023_50933_MOESM2_ESM.zip › March 2021.png]

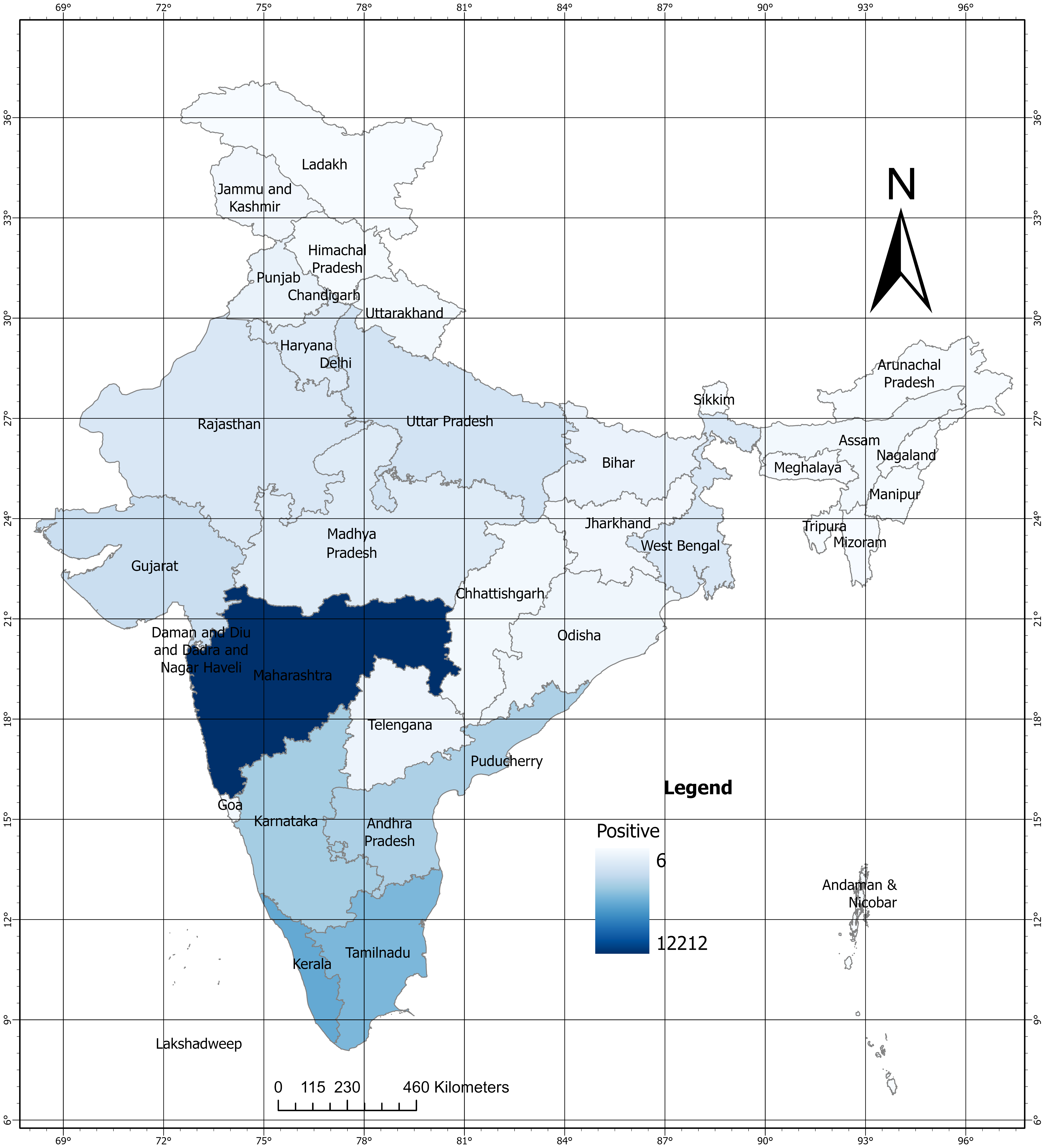

Supplement: Supplementary file 2 — Supplementary Information 2. [file 41598_2023_50933_MOESM2_ESM.zip › March 2022.png]

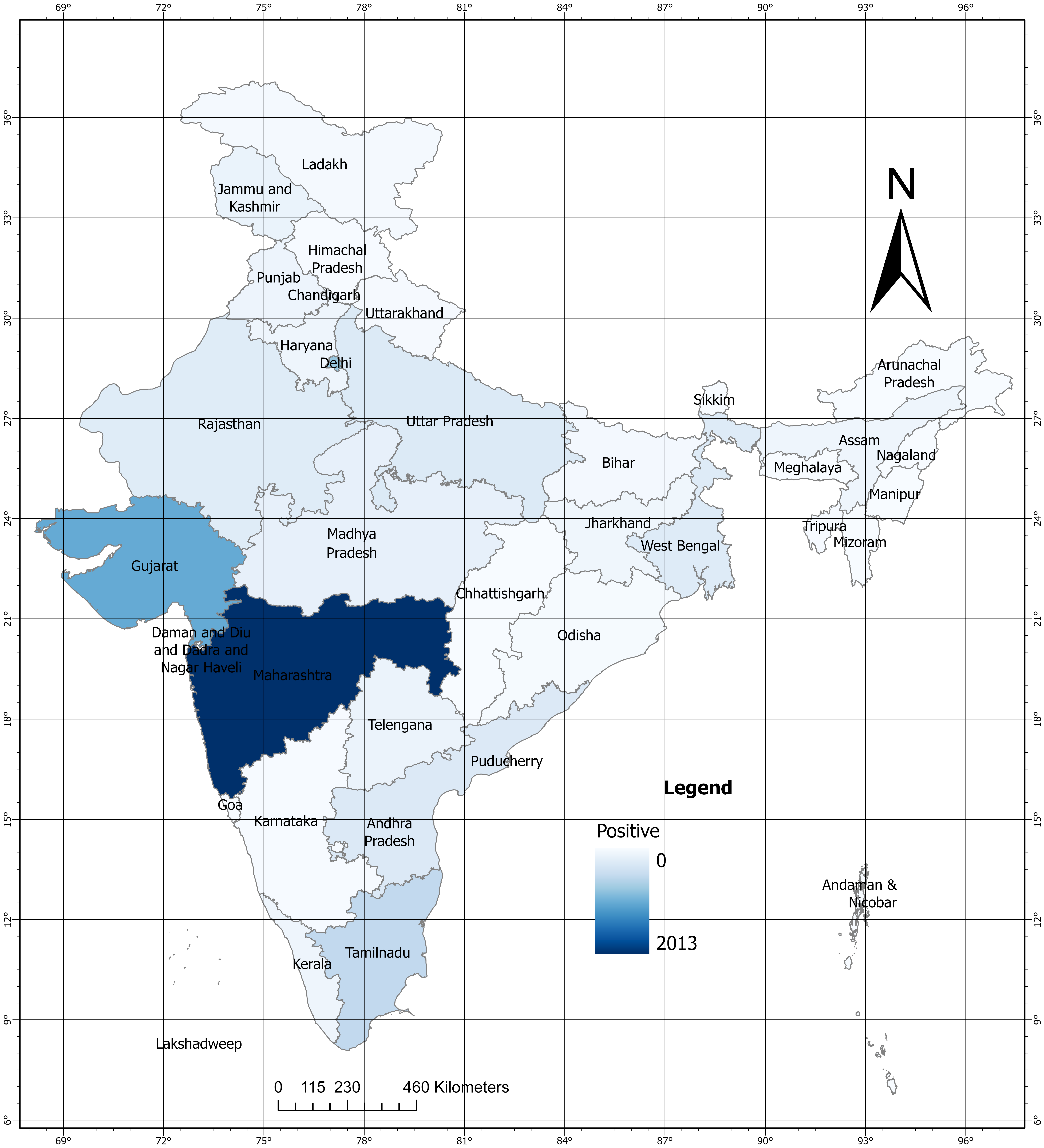

Supplement: Supplementary file 2 — Supplementary Information 2. [file 41598_2023_50933_MOESM2_ESM.zip › May 2020.png]

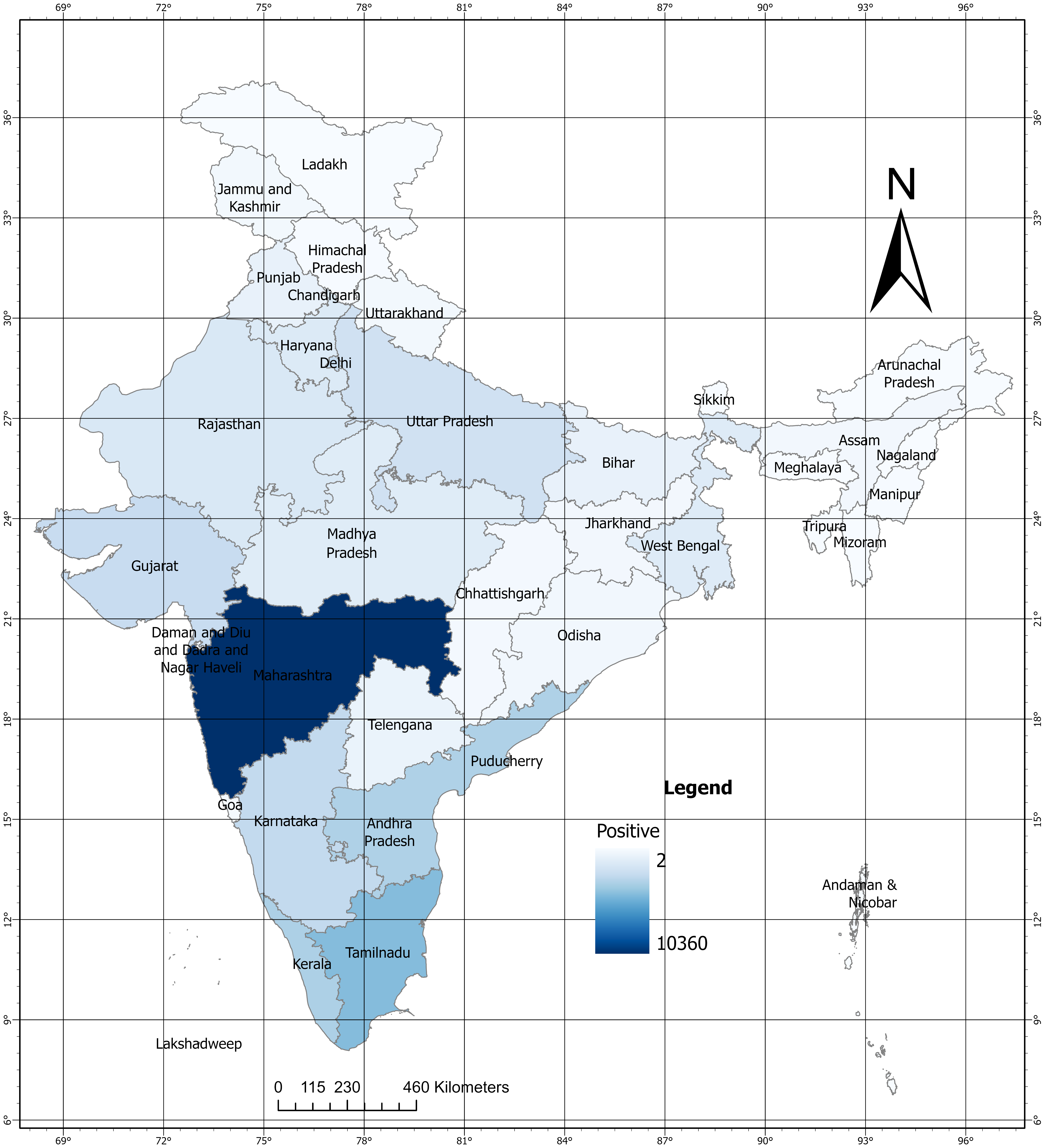

Supplement: Supplementary file 2 — Supplementary Information 2. [file 41598_2023_50933_MOESM2_ESM.zip › May 2021.png]

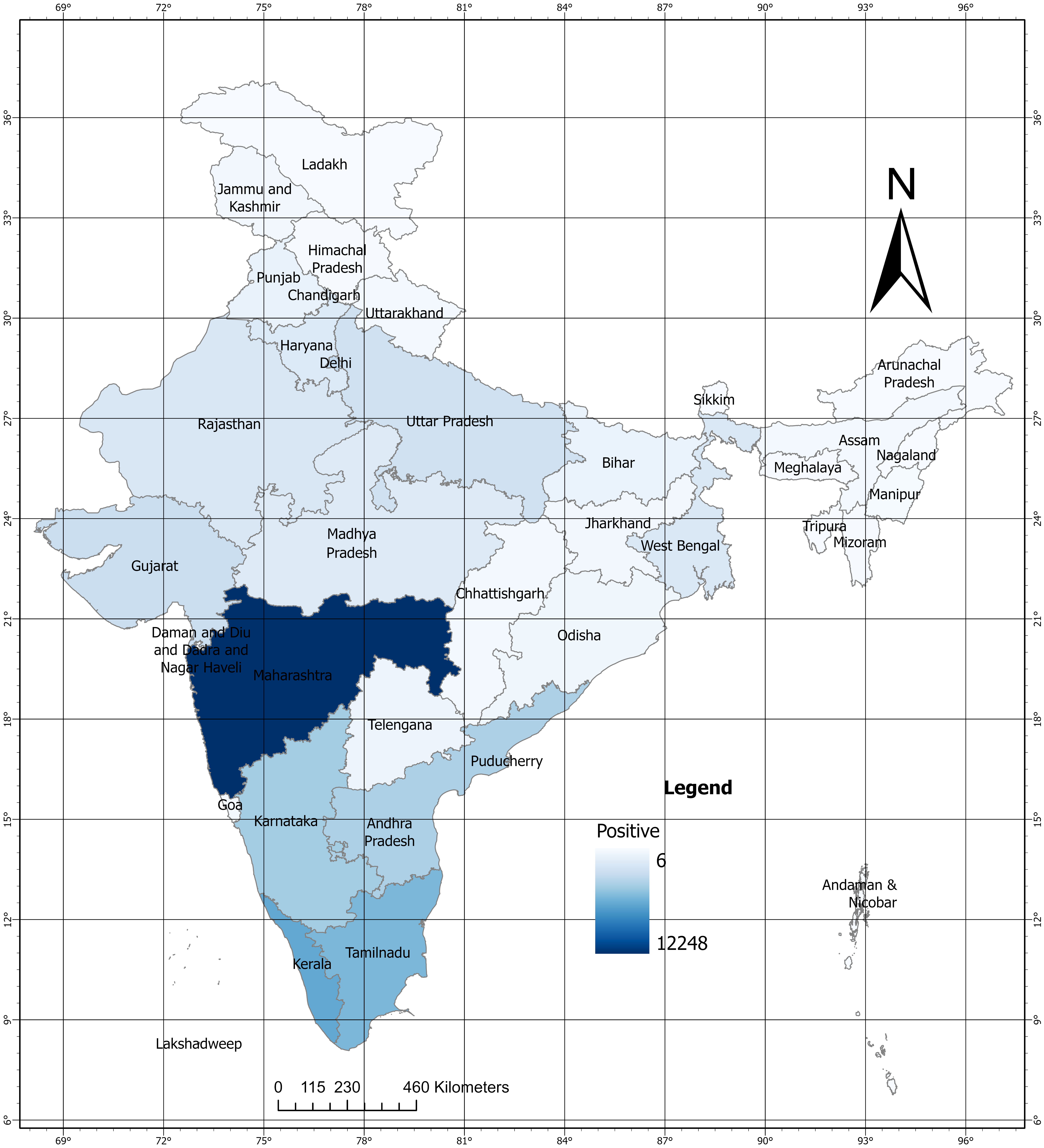

Supplement: Supplementary file 2 — Supplementary Information 2. [file 41598_2023_50933_MOESM2_ESM.zip › May 2022.png]

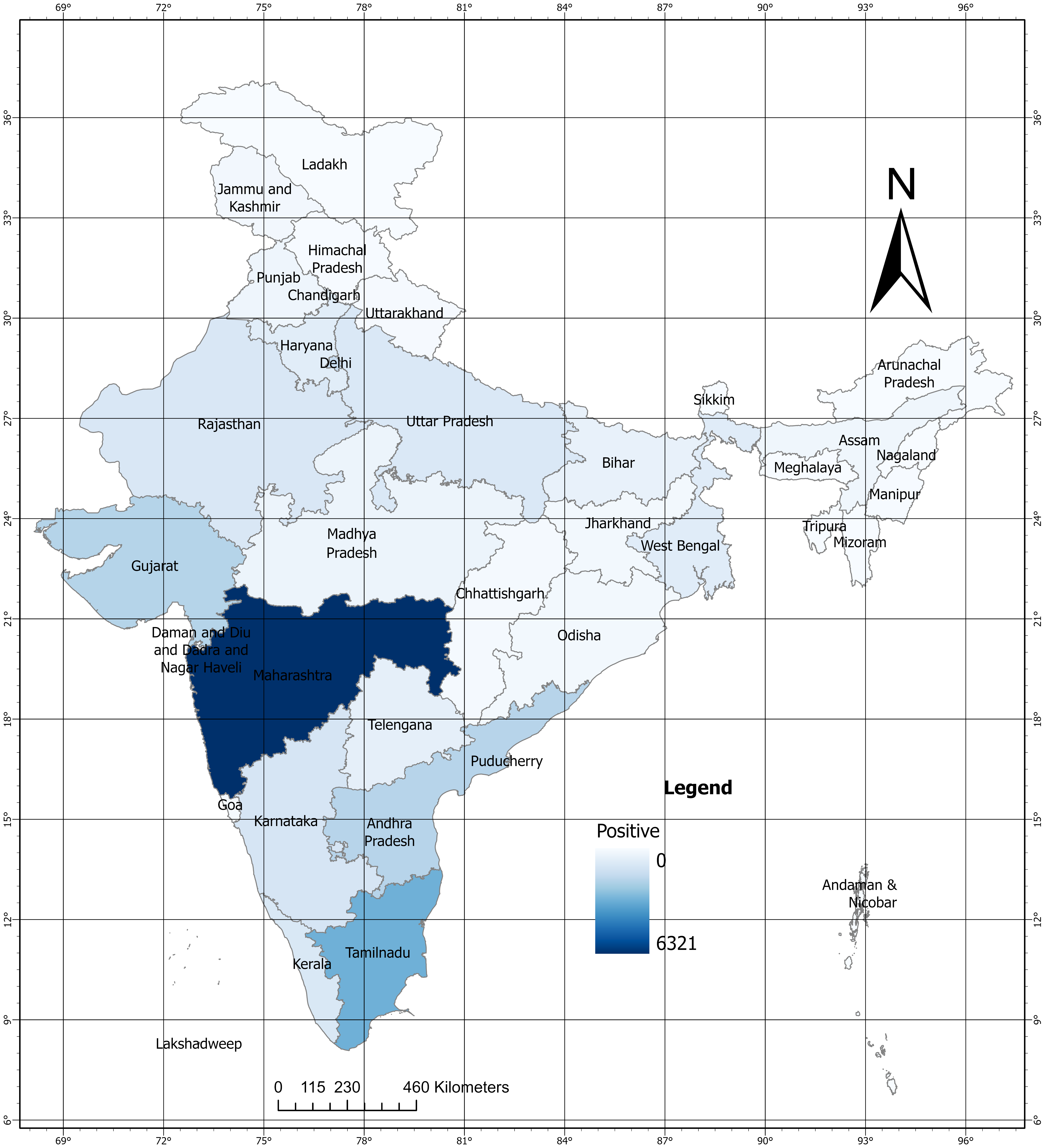

Supplement: Supplementary file 2 — Supplementary Information 2. [file 41598_2023_50933_MOESM2_ESM.zip › Nov 2020.png]

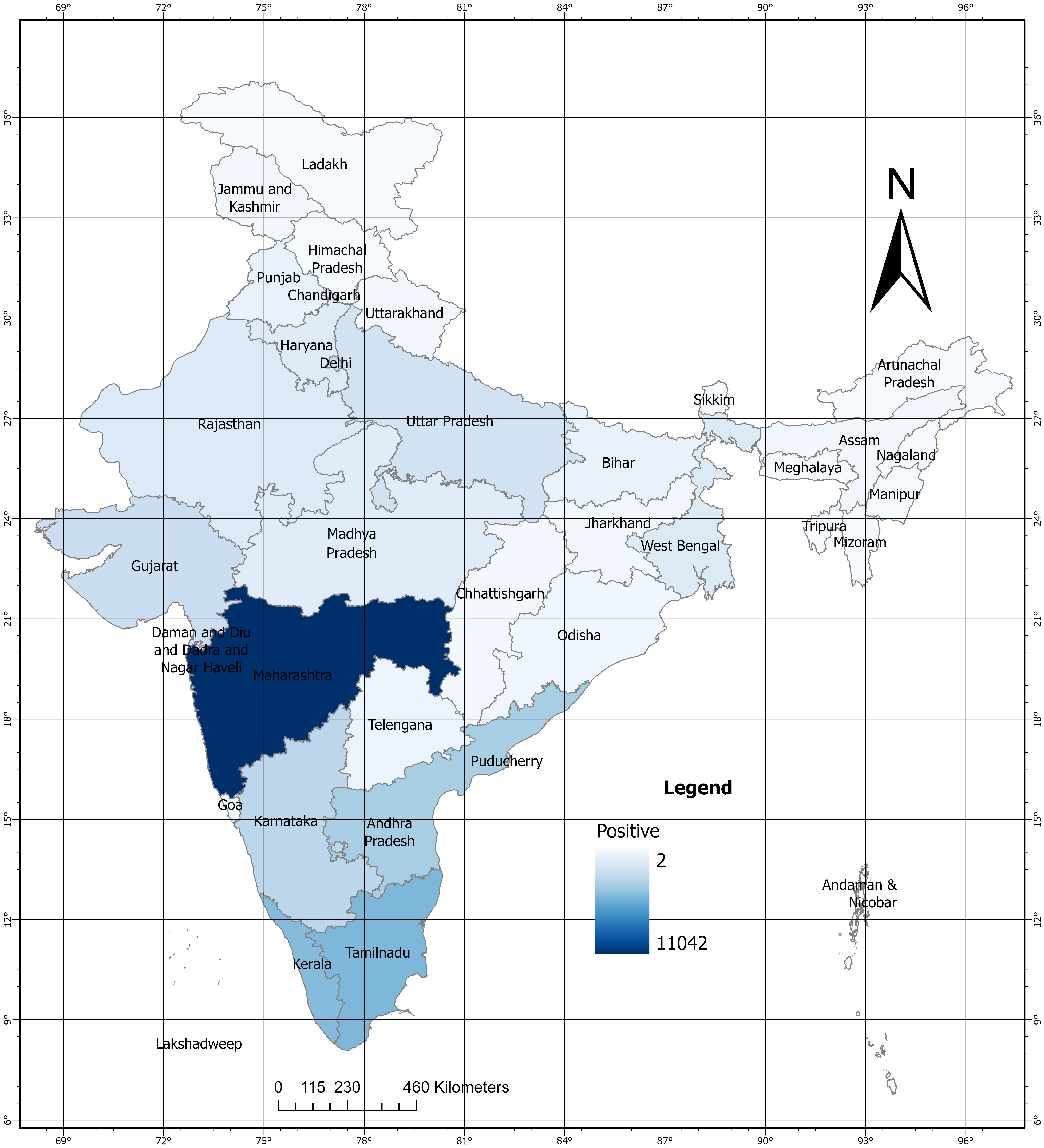

Supplement: Supplementary file 2 — Supplementary Information 2. [file 41598_2023_50933_MOESM2_ESM.zip › Nov 2021.png]

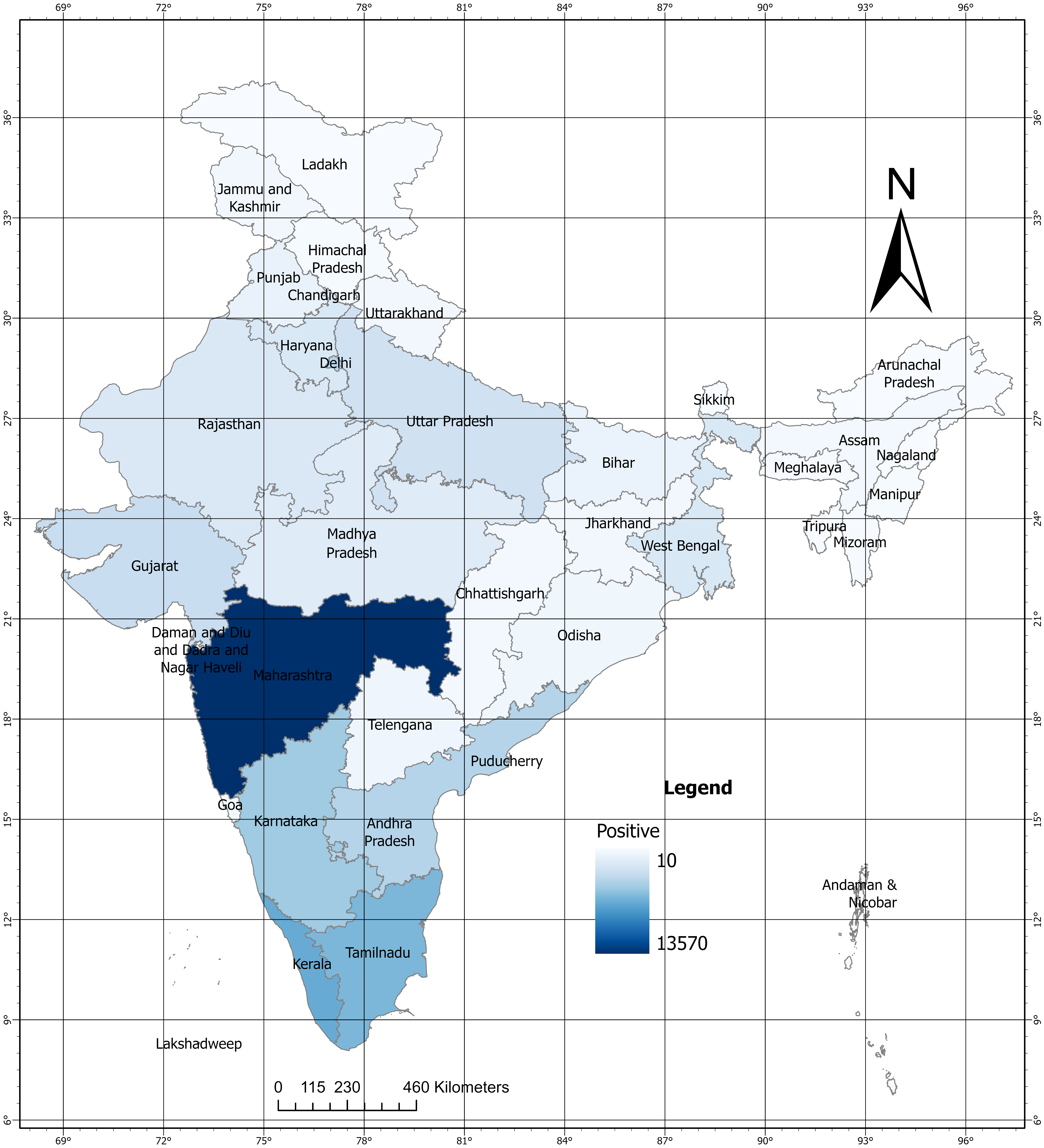

Supplement: Supplementary file 2 — Supplementary Information 2. [file 41598_2023_50933_MOESM2_ESM.zip › Nov 2022.png]

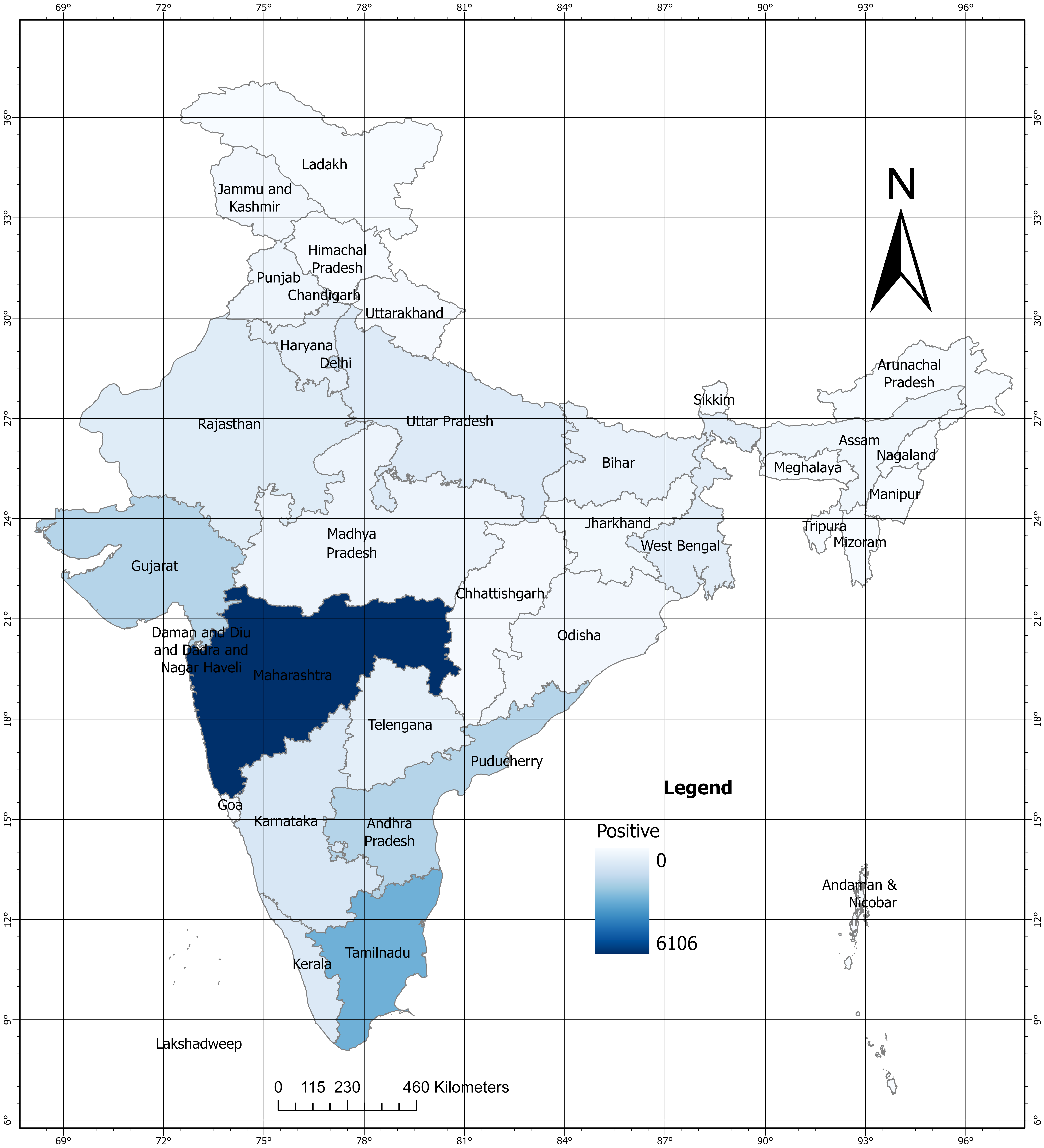

Supplement: Supplementary file 2 — Supplementary Information 2. [file 41598_2023_50933_MOESM2_ESM.zip › Oct 2020.png]

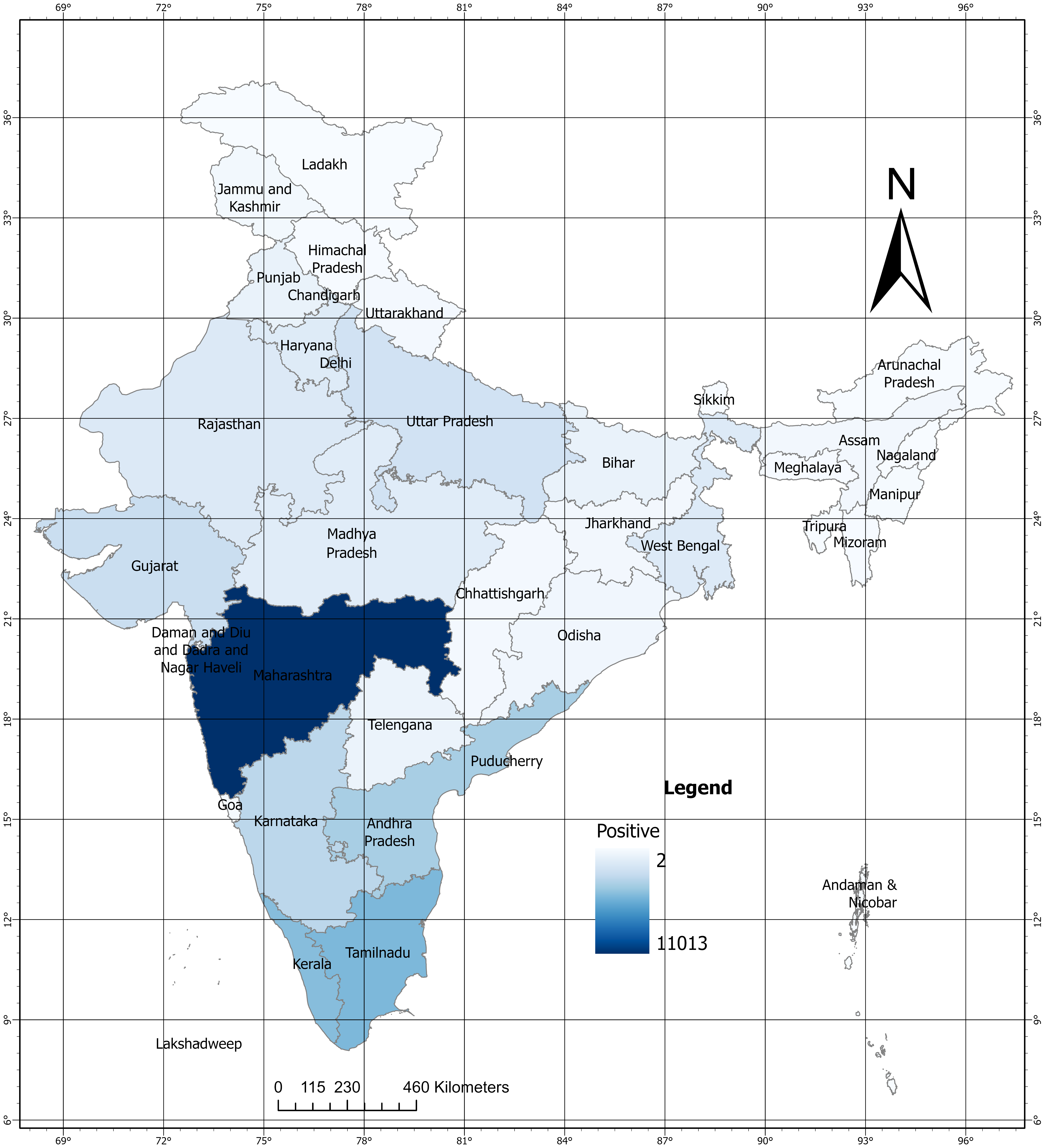

Supplement: Supplementary file 2 — Supplementary Information 2. [file 41598_2023_50933_MOESM2_ESM.zip › Oct 2021.png]

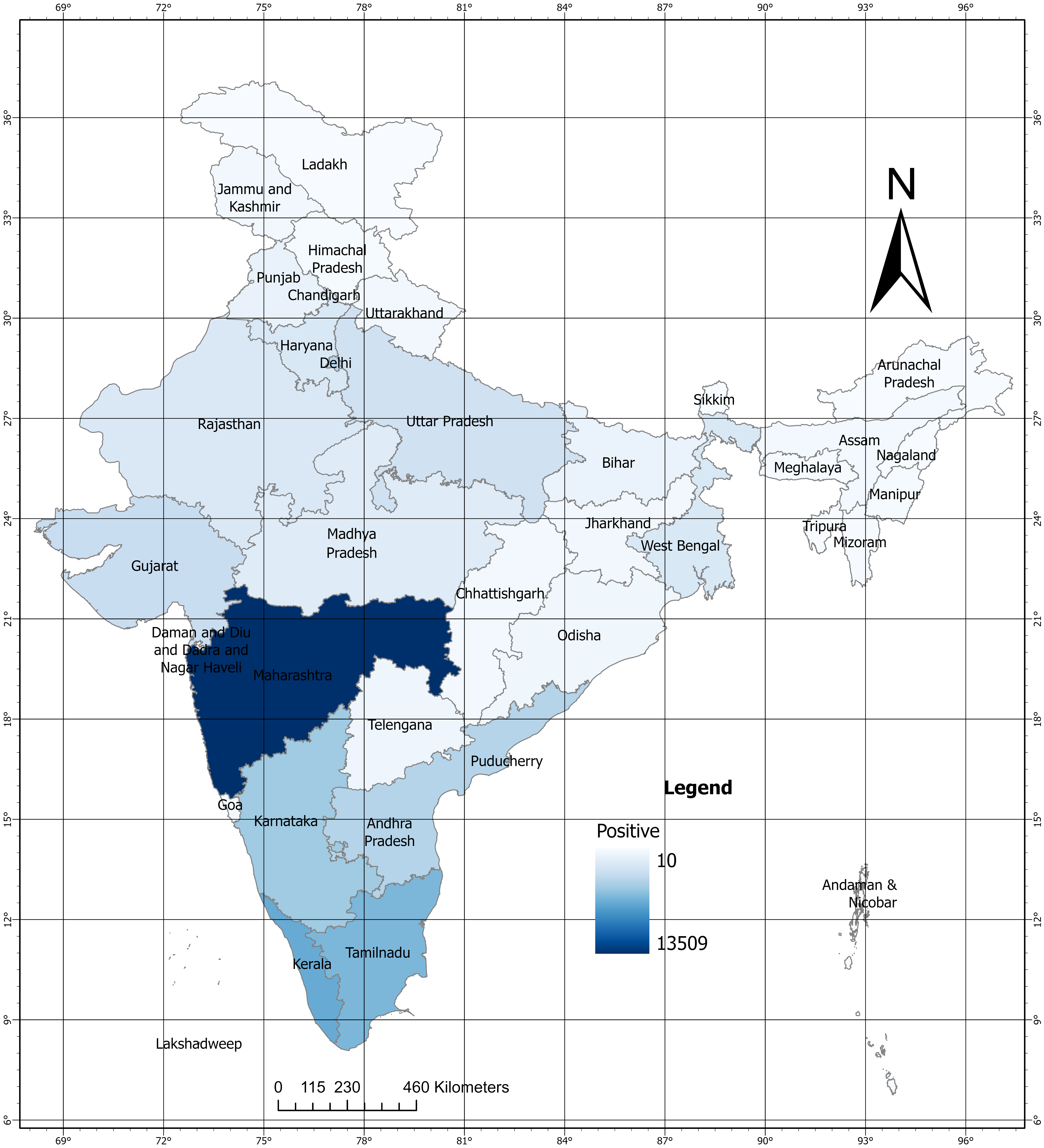

Supplement: Supplementary file 2 — Supplementary Information 2. [file 41598_2023_50933_MOESM2_ESM.zip › Oct 2022.png]

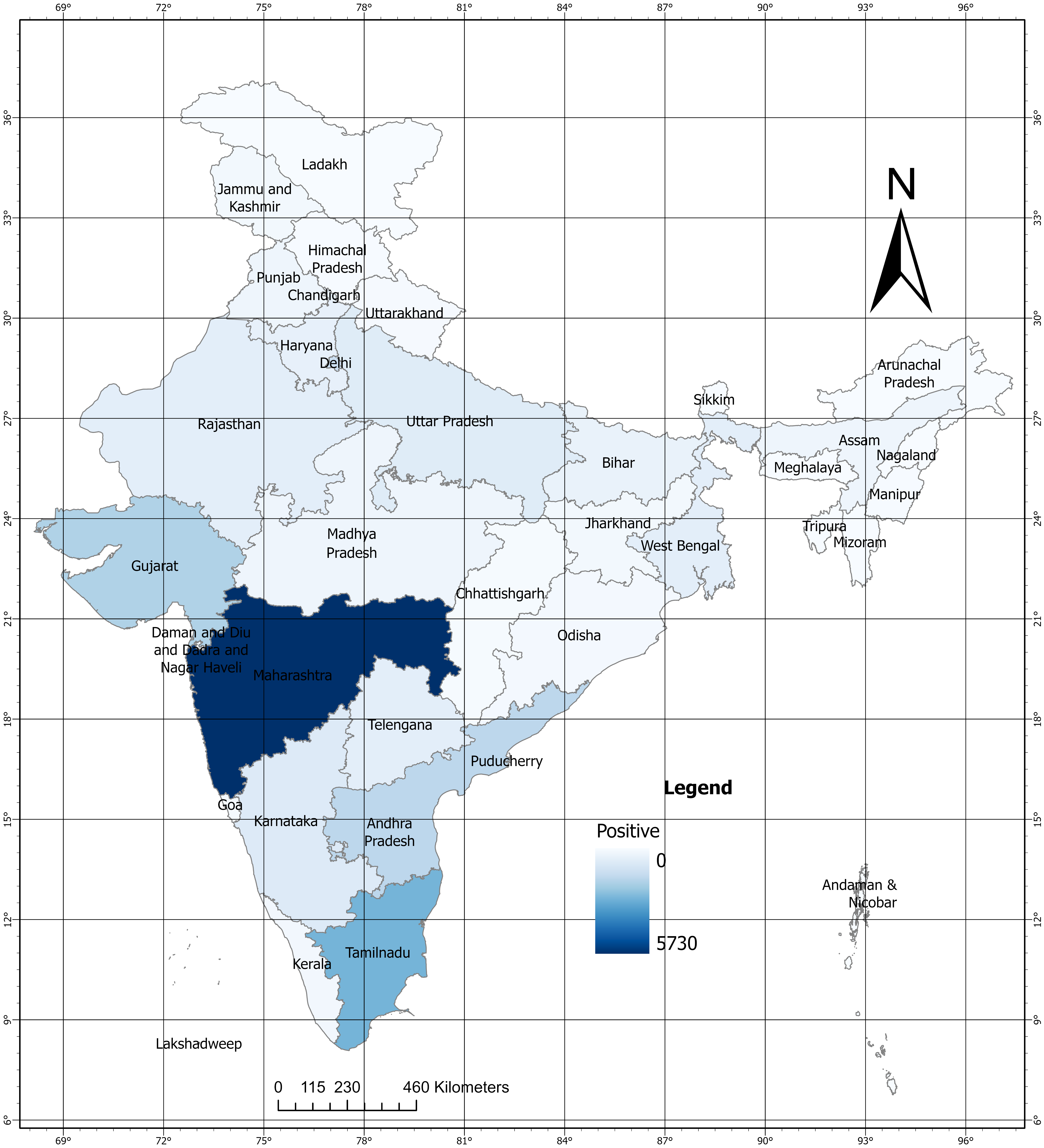

Supplement: Supplementary file 2 — Supplementary Information 2. [file 41598_2023_50933_MOESM2_ESM.zip › Sept 2020.png]

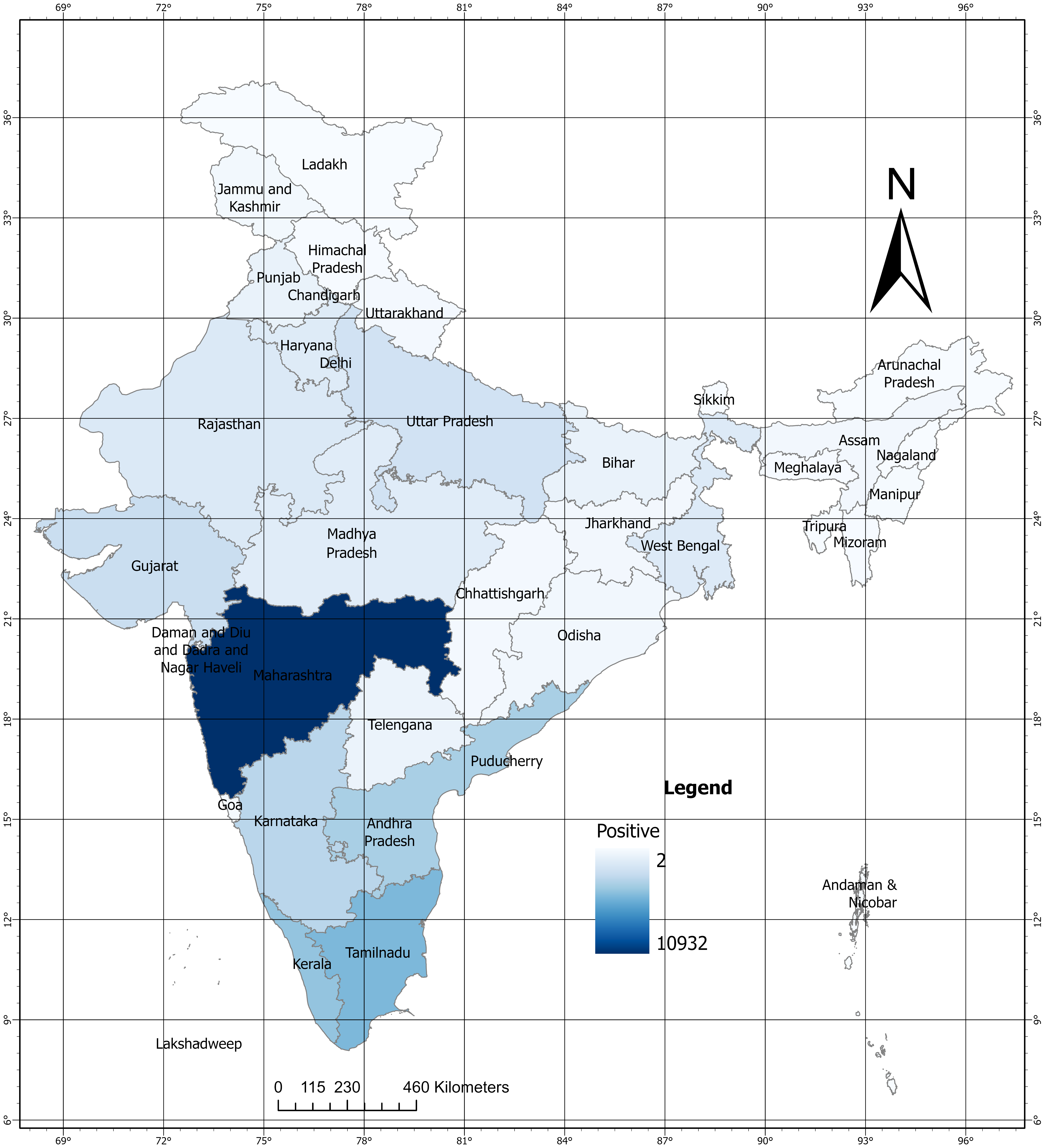

Supplement: Supplementary file 2 — Supplementary Information 2. [file 41598_2023_50933_MOESM2_ESM.zip › Sept 2021.png]

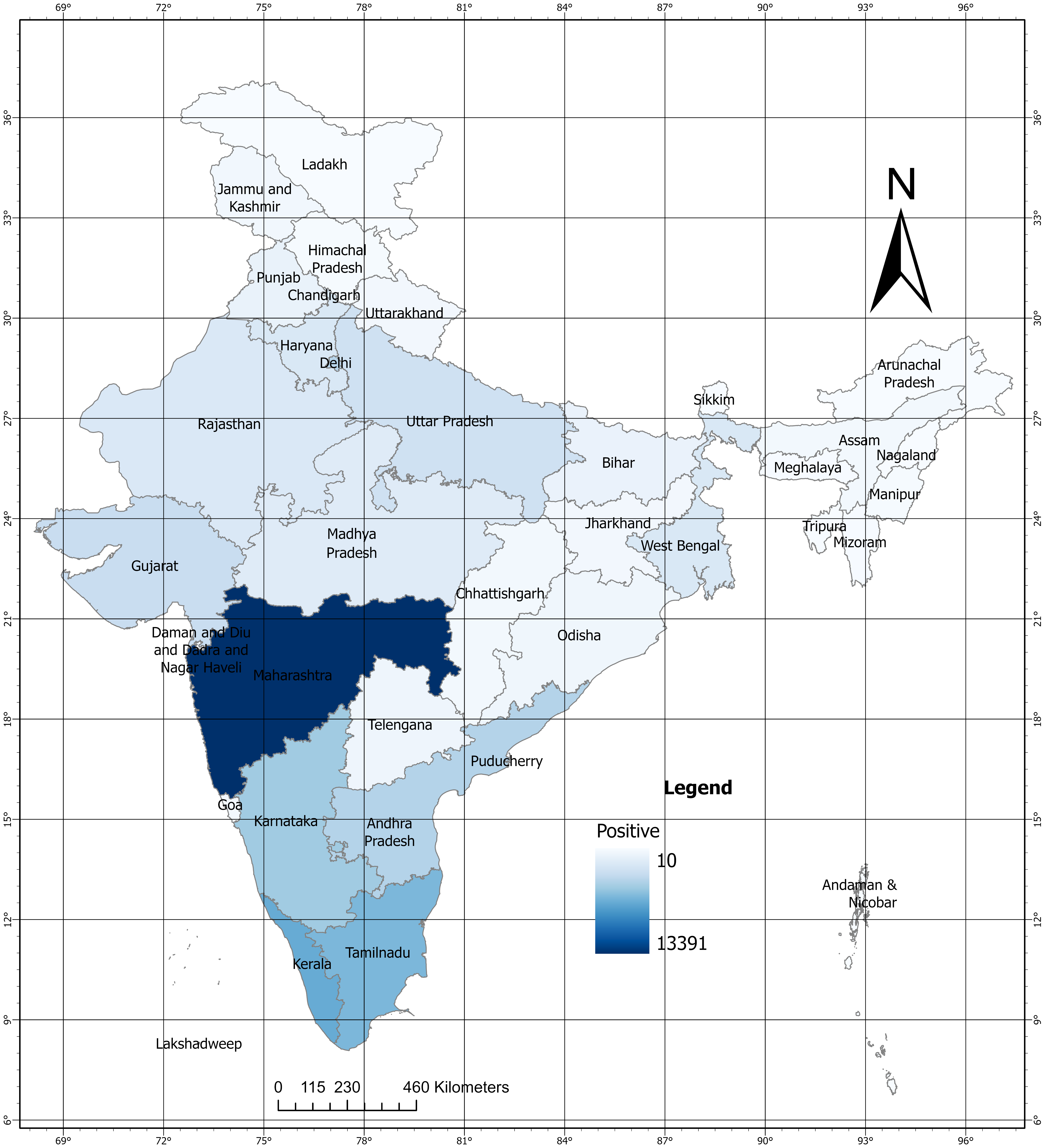

Supplement: Supplementary file 2 — Supplementary Information 2. [file 41598_2023_50933_MOESM2_ESM.zip › Sept 2022.png]

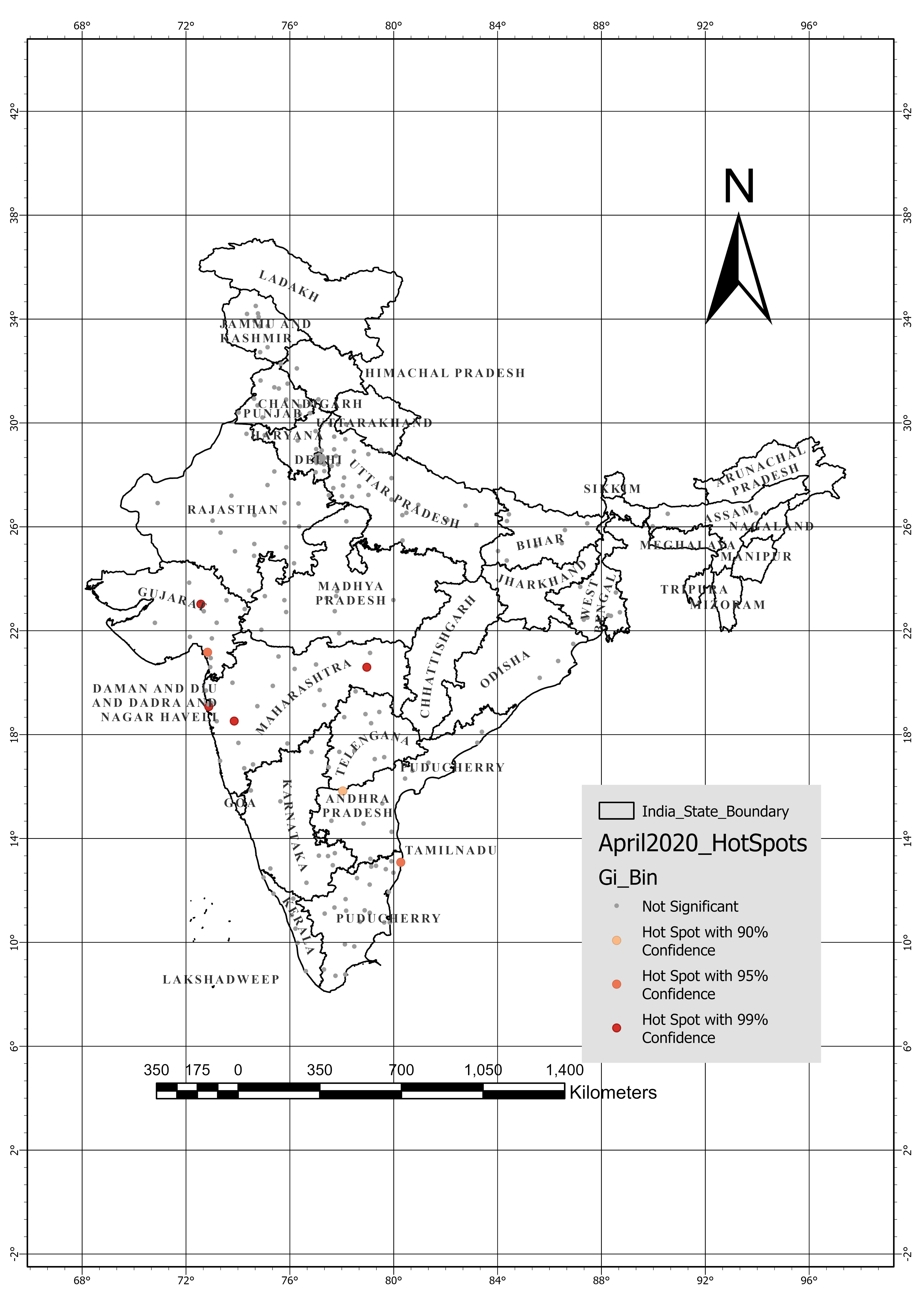

Supplement: Supplementary file 3 — Supplementary Information 3. [file 41598_2023_50933_MOESM3_ESM.zip › April 2020.jpg]

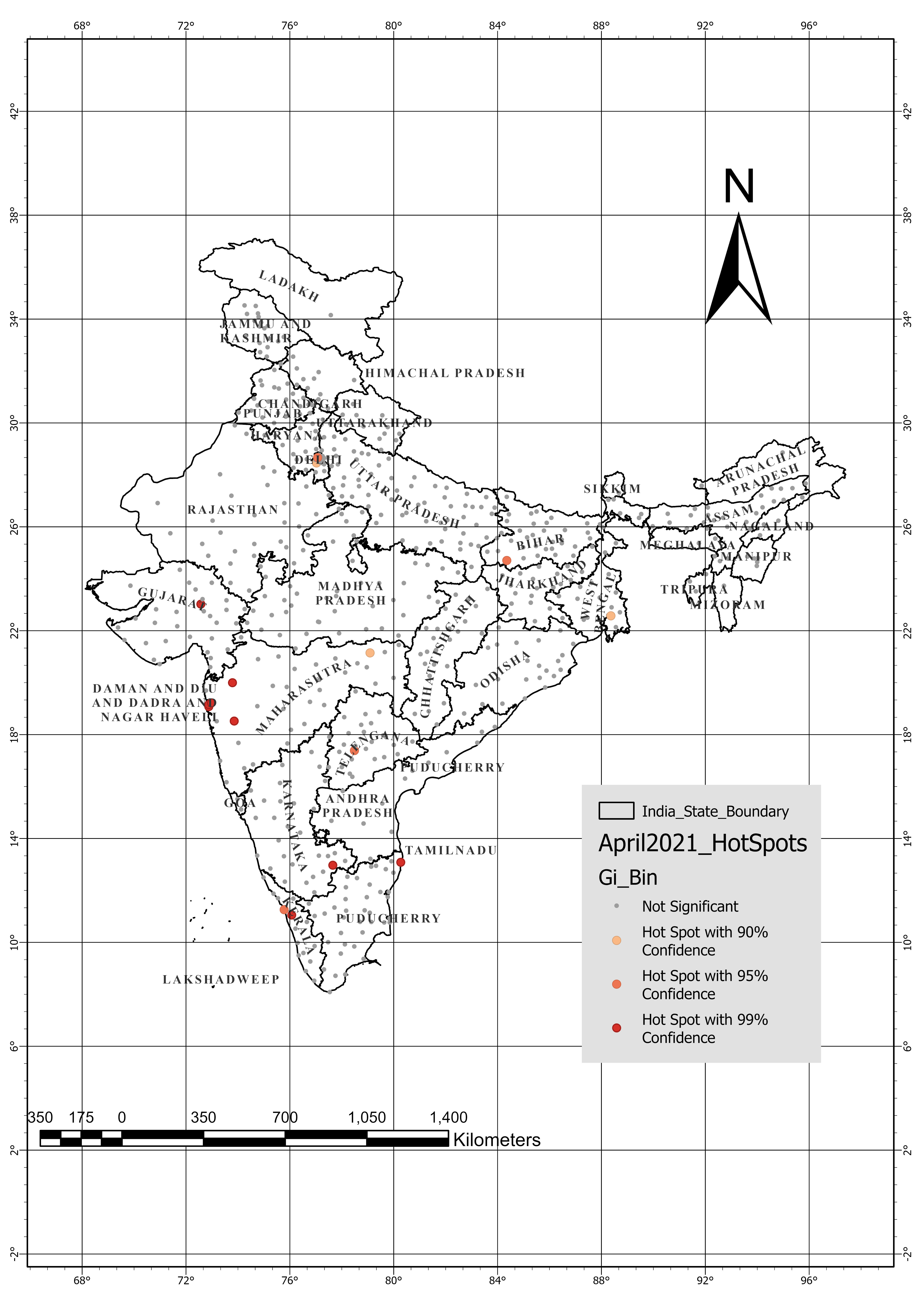

Supplement: Supplementary file 3 — Supplementary Information 3. [file 41598_2023_50933_MOESM3_ESM.zip › April 2021.jpg]

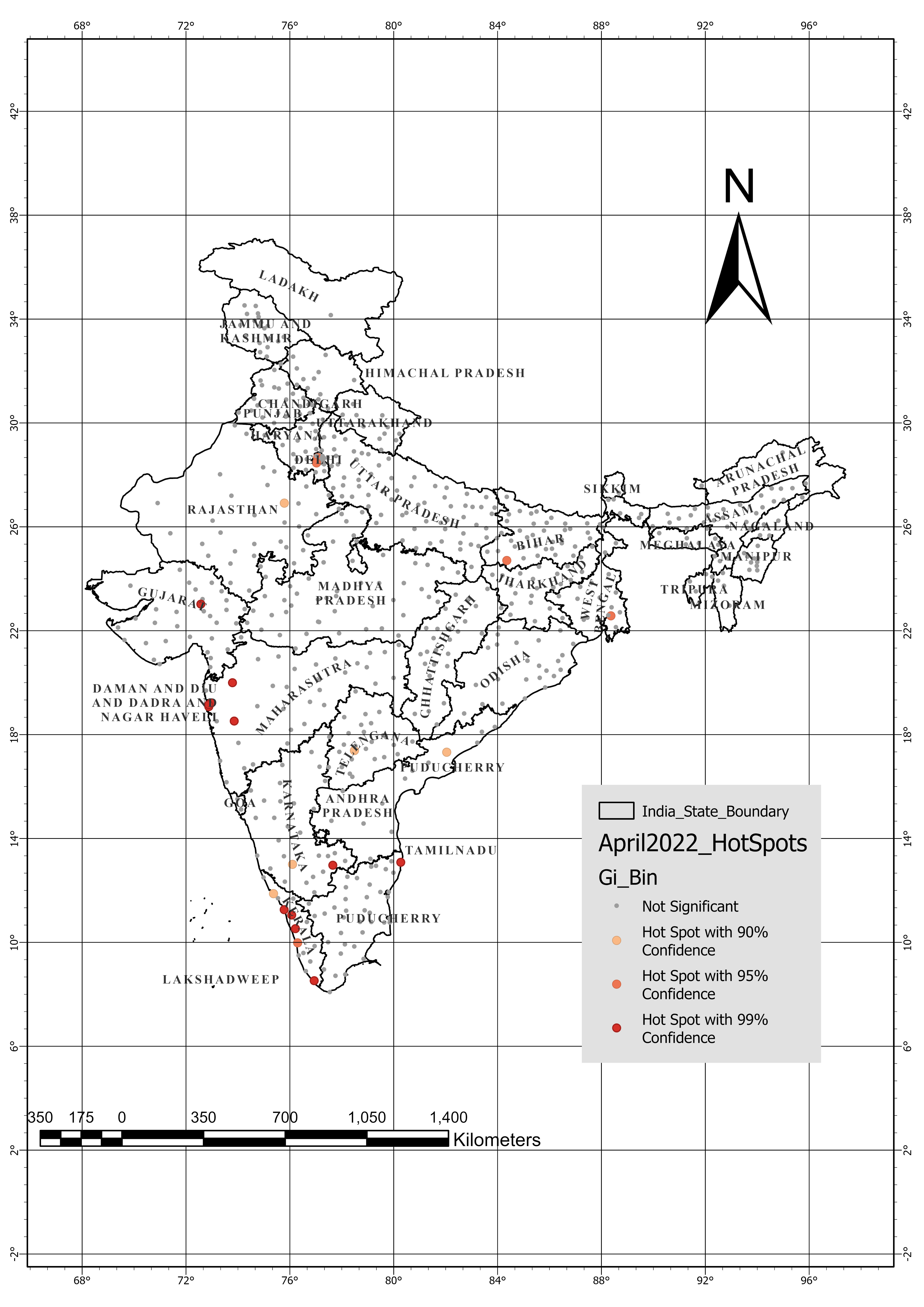

Supplement: Supplementary file 3 — Supplementary Information 3. [file 41598_2023_50933_MOESM3_ESM.zip › April 2022.jpg]

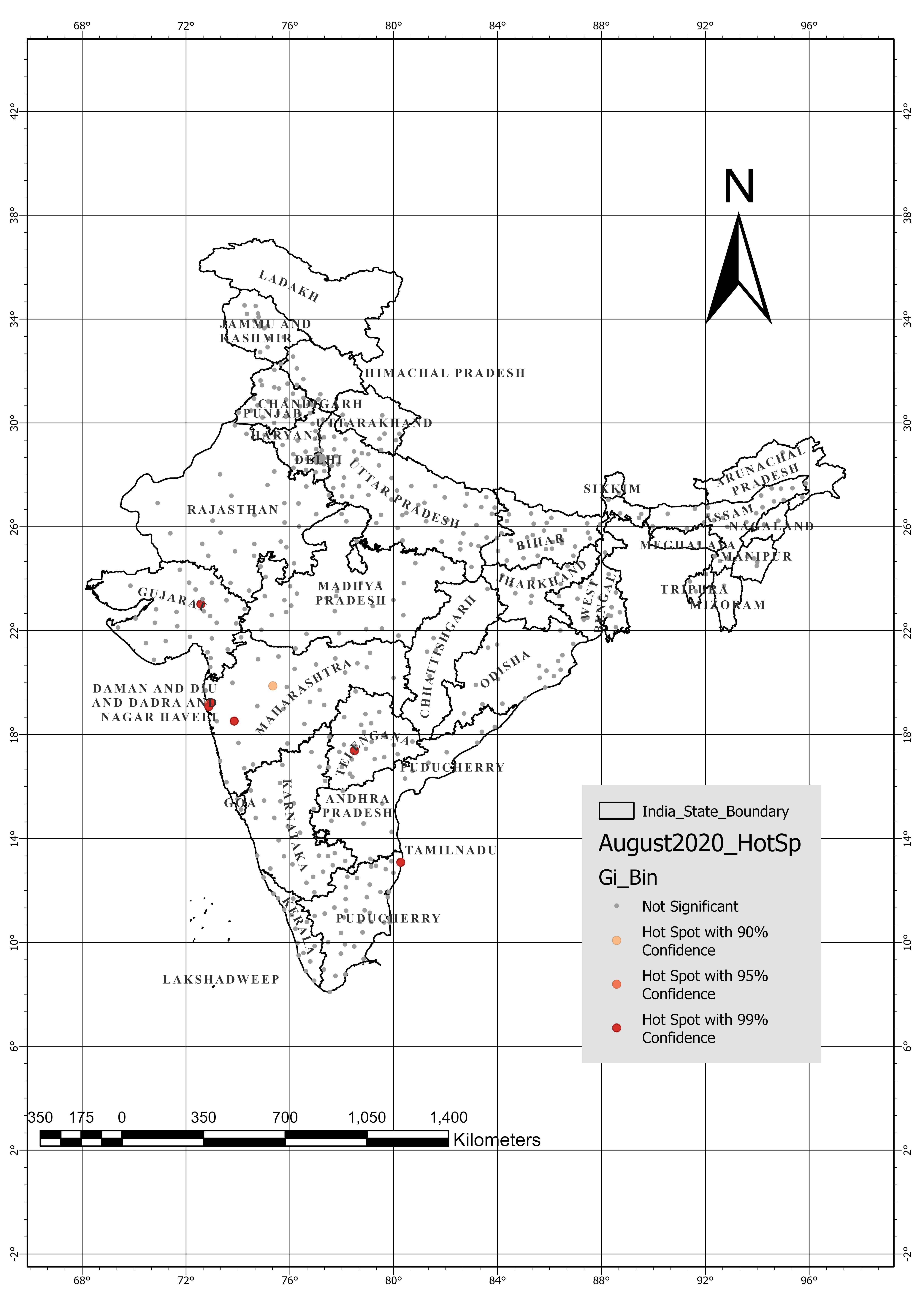

Supplement: Supplementary file 3 — Supplementary Information 3. [file 41598_2023_50933_MOESM3_ESM.zip › August 2020.jpg]

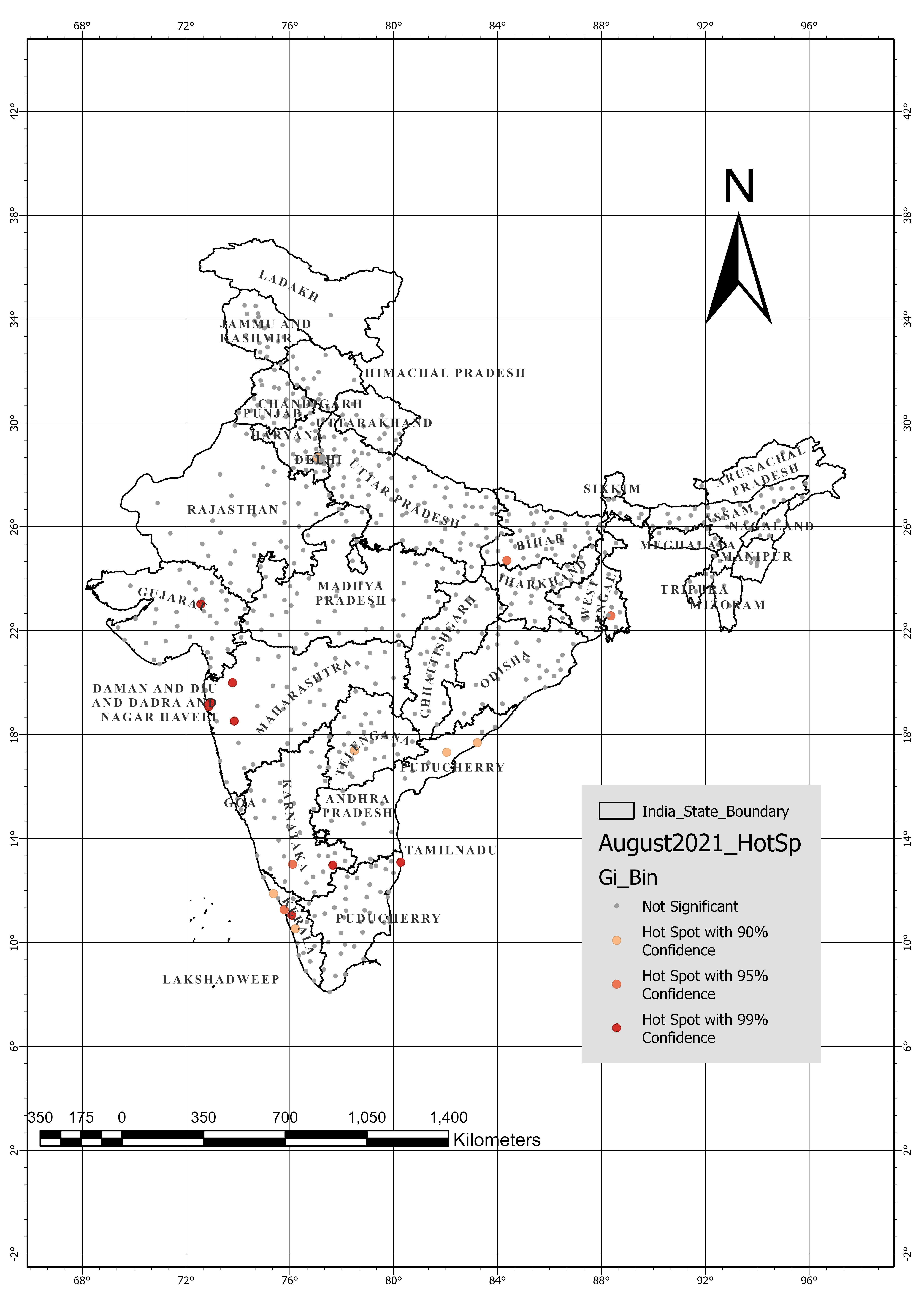

Supplement: Supplementary file 3 — Supplementary Information 3. [file 41598_2023_50933_MOESM3_ESM.zip › August 2021.jpg]

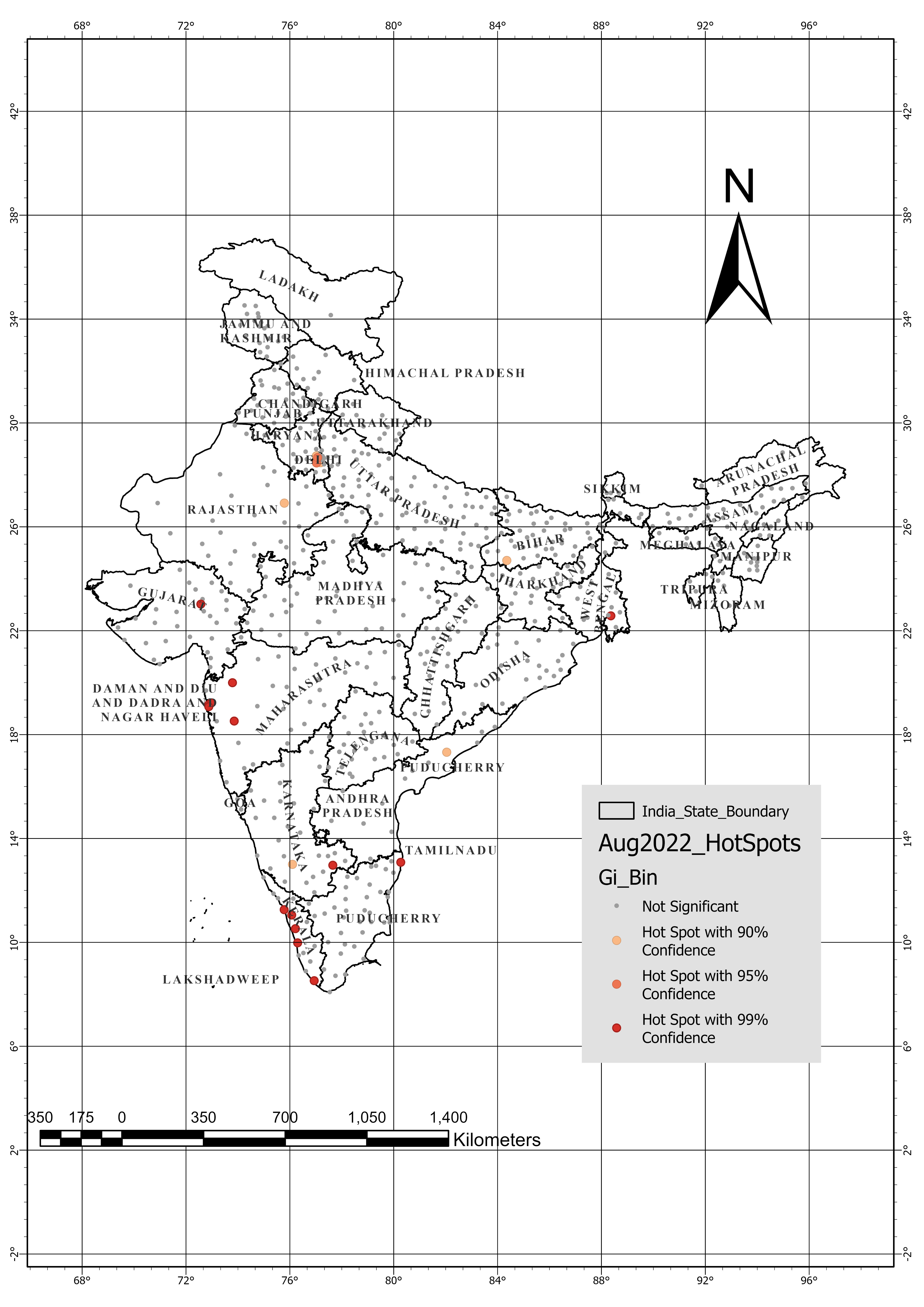

Supplement: Supplementary file 3 — Supplementary Information 3. [file 41598_2023_50933_MOESM3_ESM.zip › August 2022.jpg]

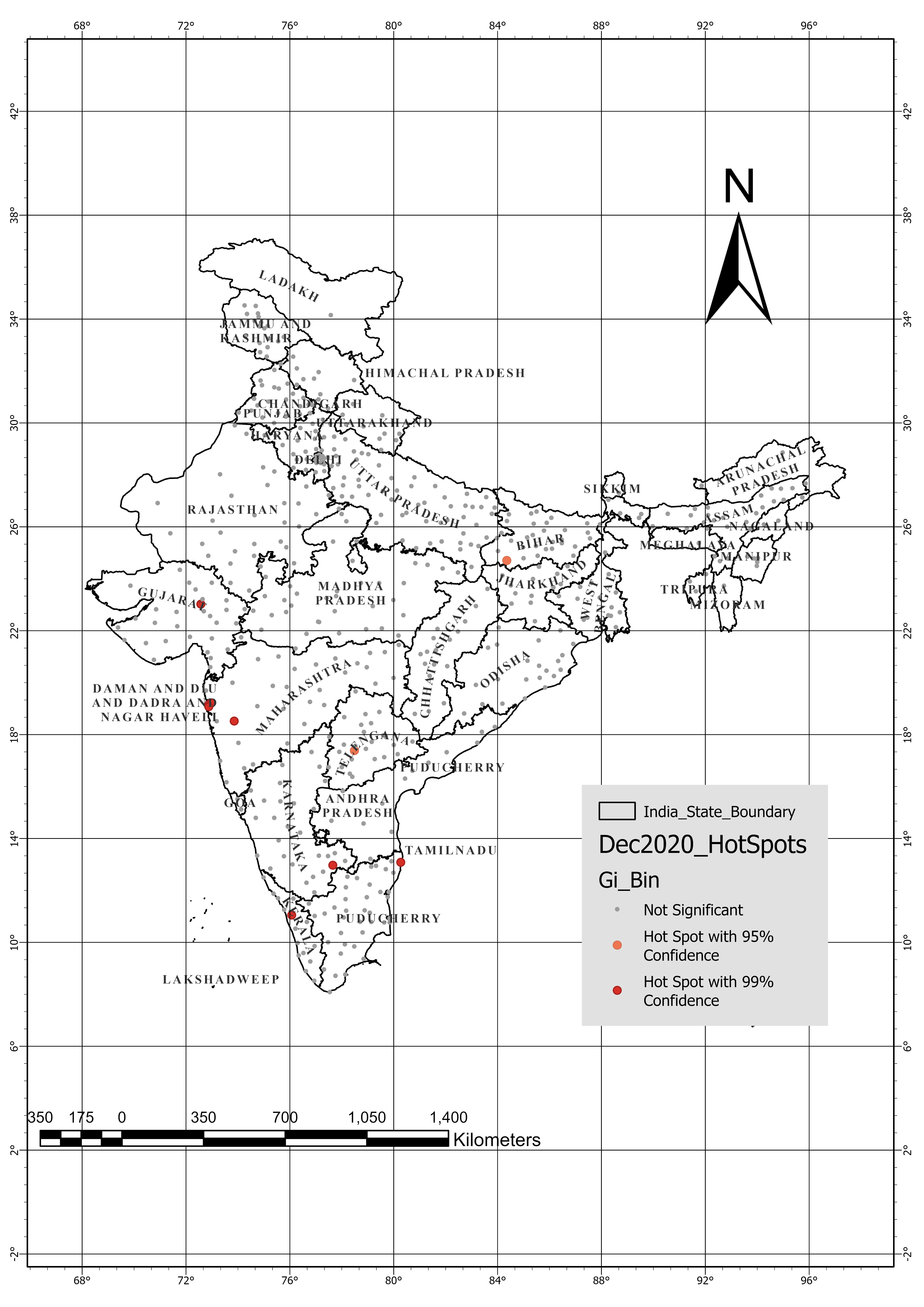

Supplement: Supplementary file 3 — Supplementary Information 3. [file 41598_2023_50933_MOESM3_ESM.zip › December 2020.jpg]

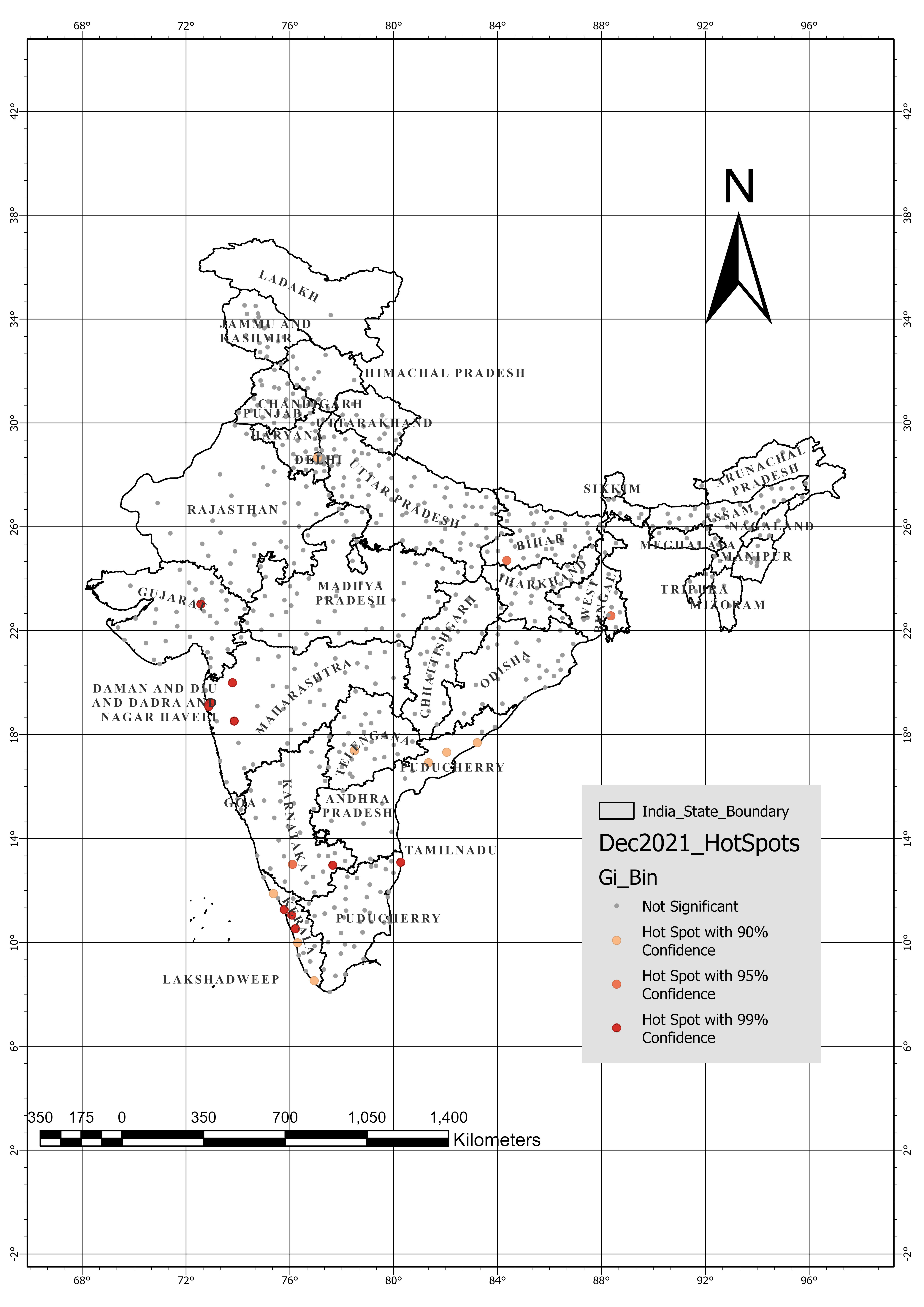

Supplement: Supplementary file 3 — Supplementary Information 3. [file 41598_2023_50933_MOESM3_ESM.zip › December 2021.jpg]

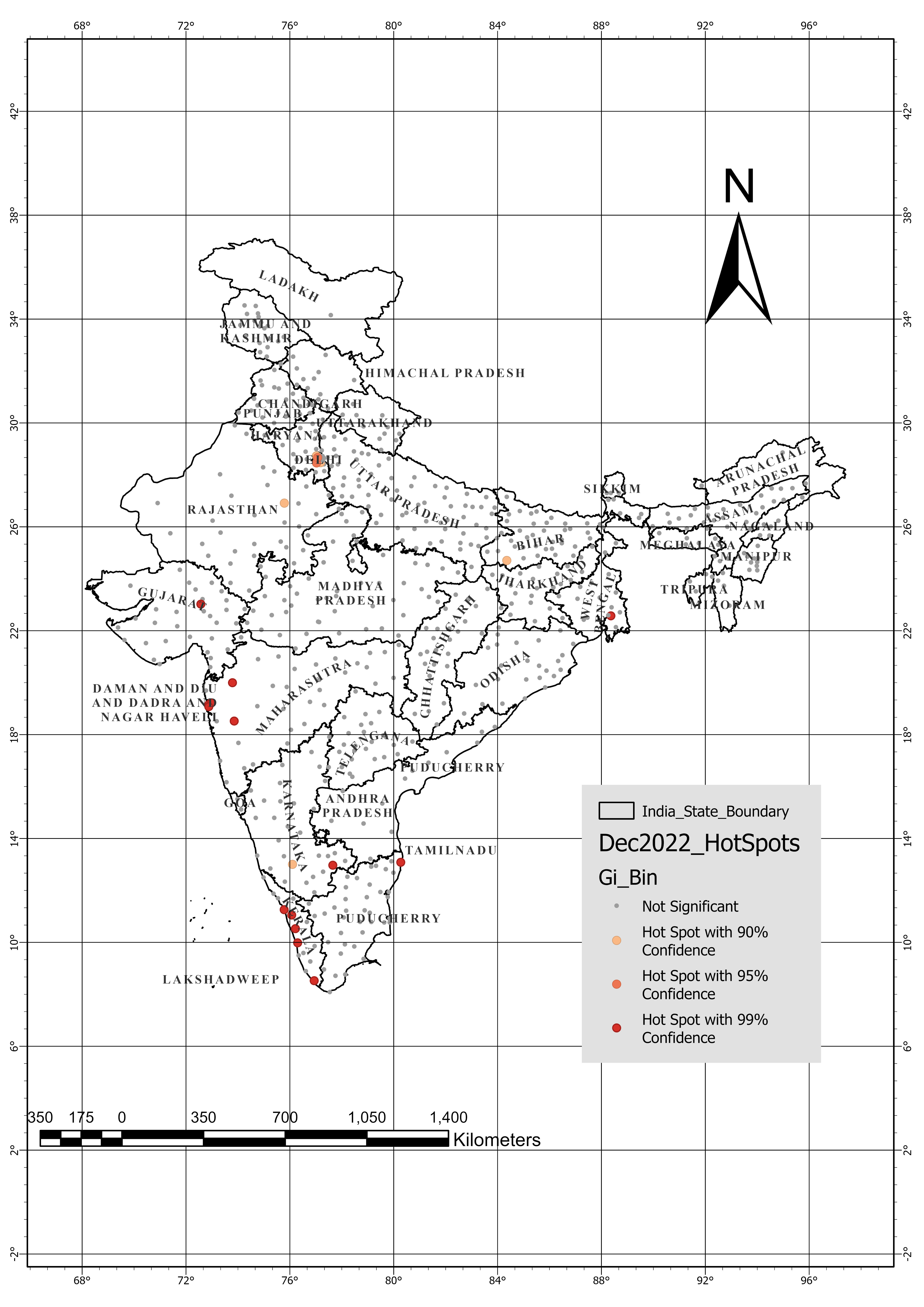

Supplement: Supplementary file 3 — Supplementary Information 3. [file 41598_2023_50933_MOESM3_ESM.zip › December 2022.jpg]

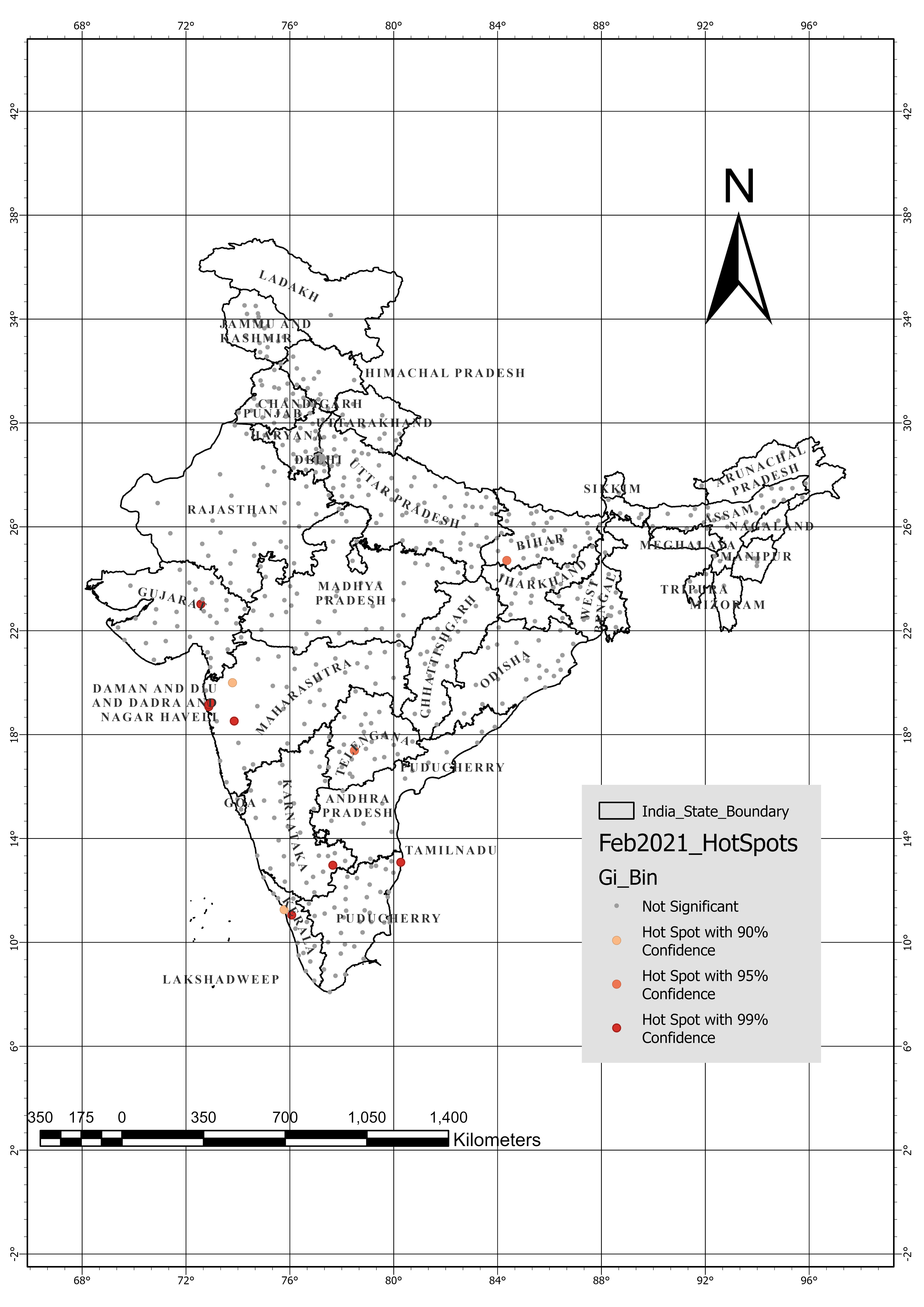

Supplement: Supplementary file 3 — Supplementary Information 3. [file 41598_2023_50933_MOESM3_ESM.zip › February 2021.jpg]

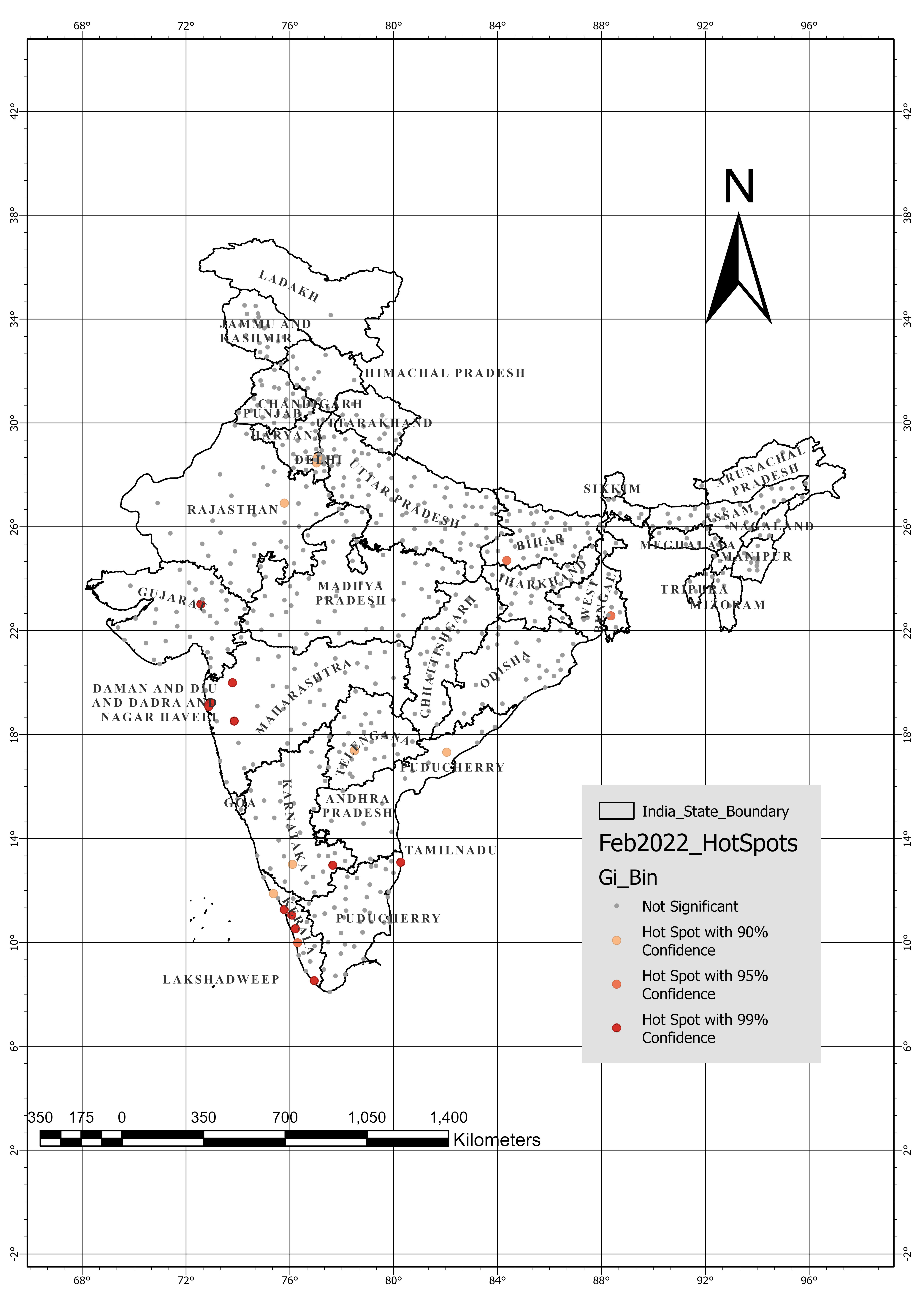

Supplement: Supplementary file 3 — Supplementary Information 3. [file 41598_2023_50933_MOESM3_ESM.zip › February 2022.jpg]

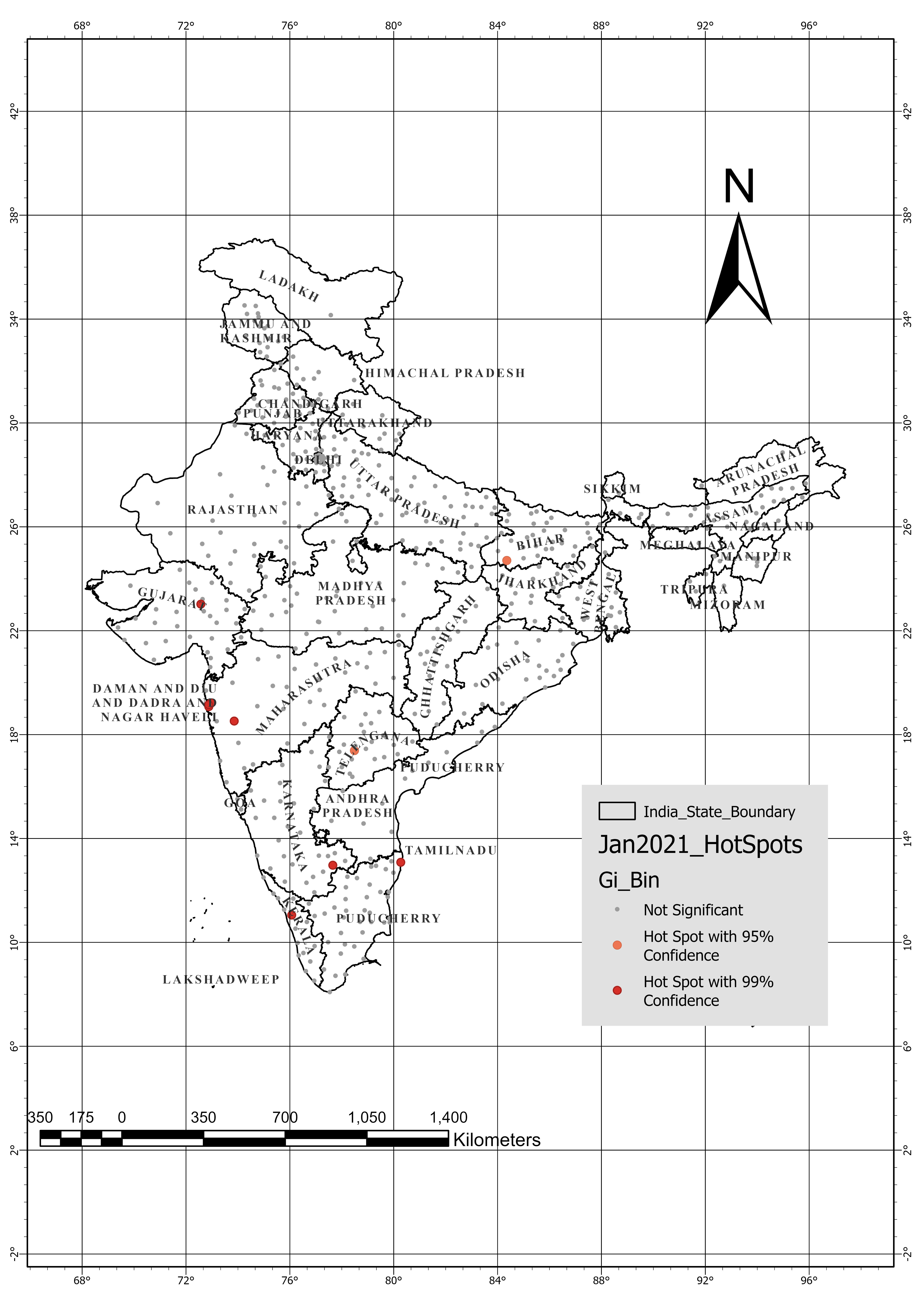

Supplement: Supplementary file 3 — Supplementary Information 3. [file 41598_2023_50933_MOESM3_ESM.zip › January 2021.jpg]

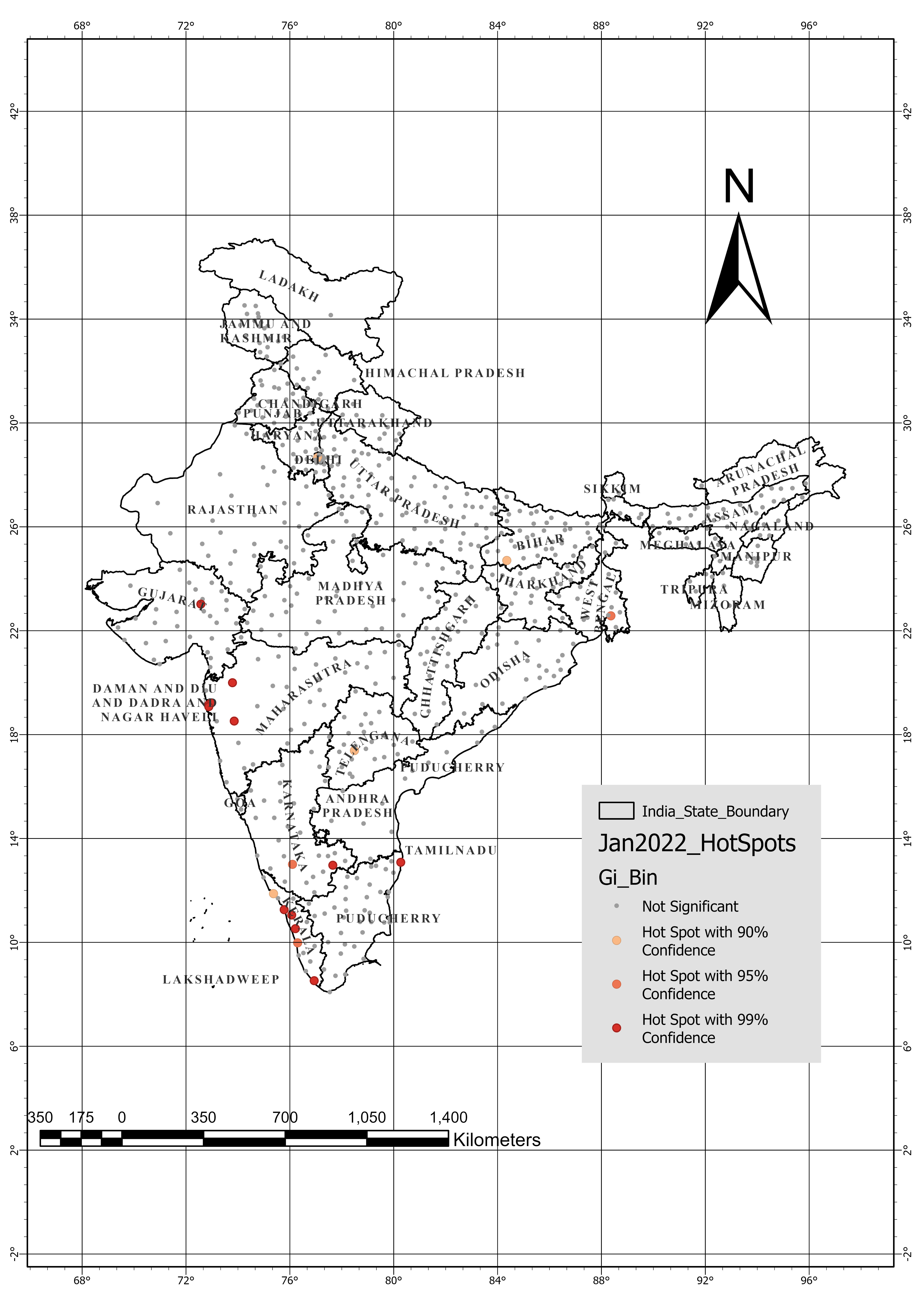

Supplement: Supplementary file 3 — Supplementary Information 3. [file 41598_2023_50933_MOESM3_ESM.zip › January 2022.jpg]

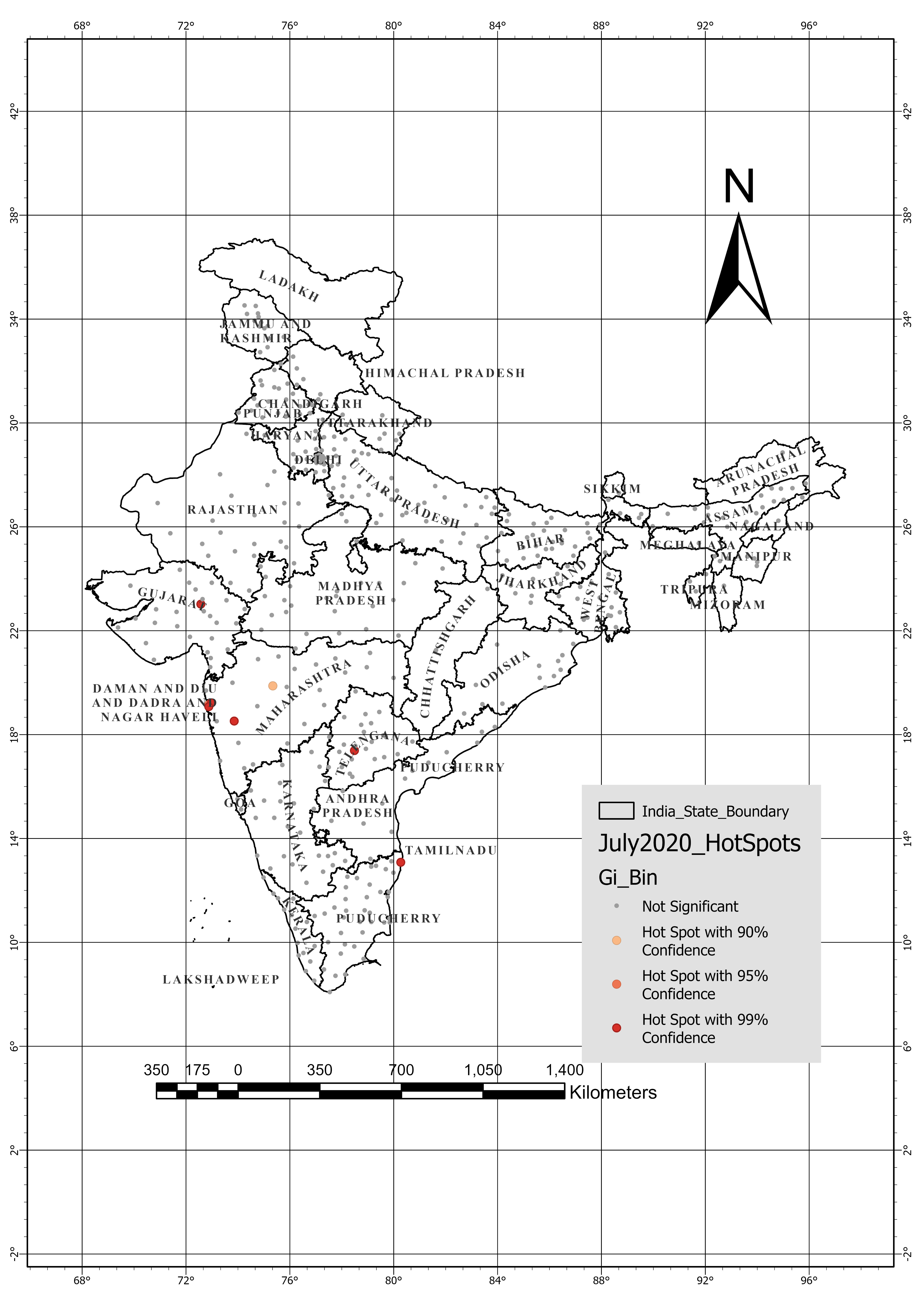

Supplement: Supplementary file 3 — Supplementary Information 3. [file 41598_2023_50933_MOESM3_ESM.zip › July 2020.jpg]

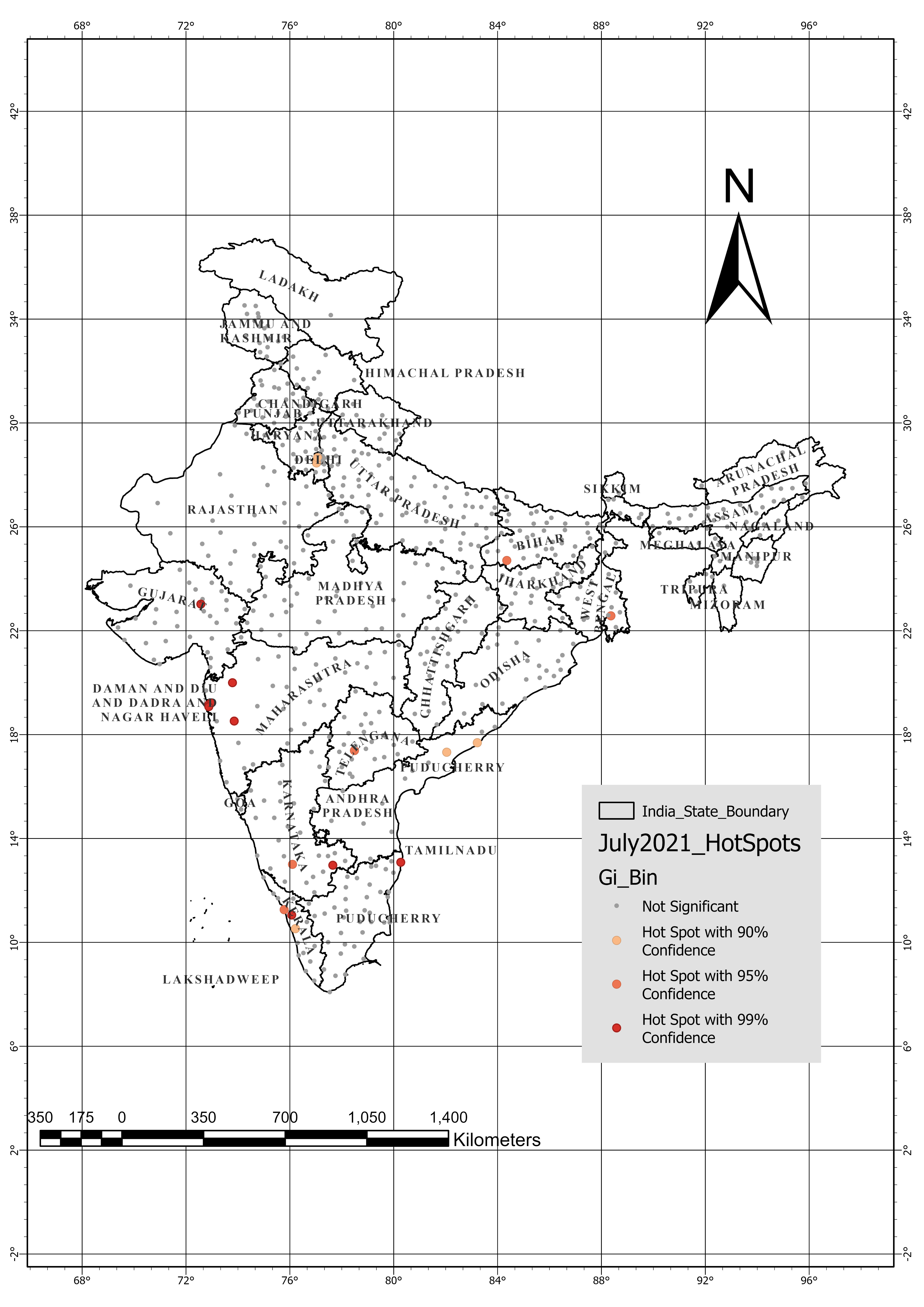

Supplement: Supplementary file 3 — Supplementary Information 3. [file 41598_2023_50933_MOESM3_ESM.zip › July 2021.jpg]

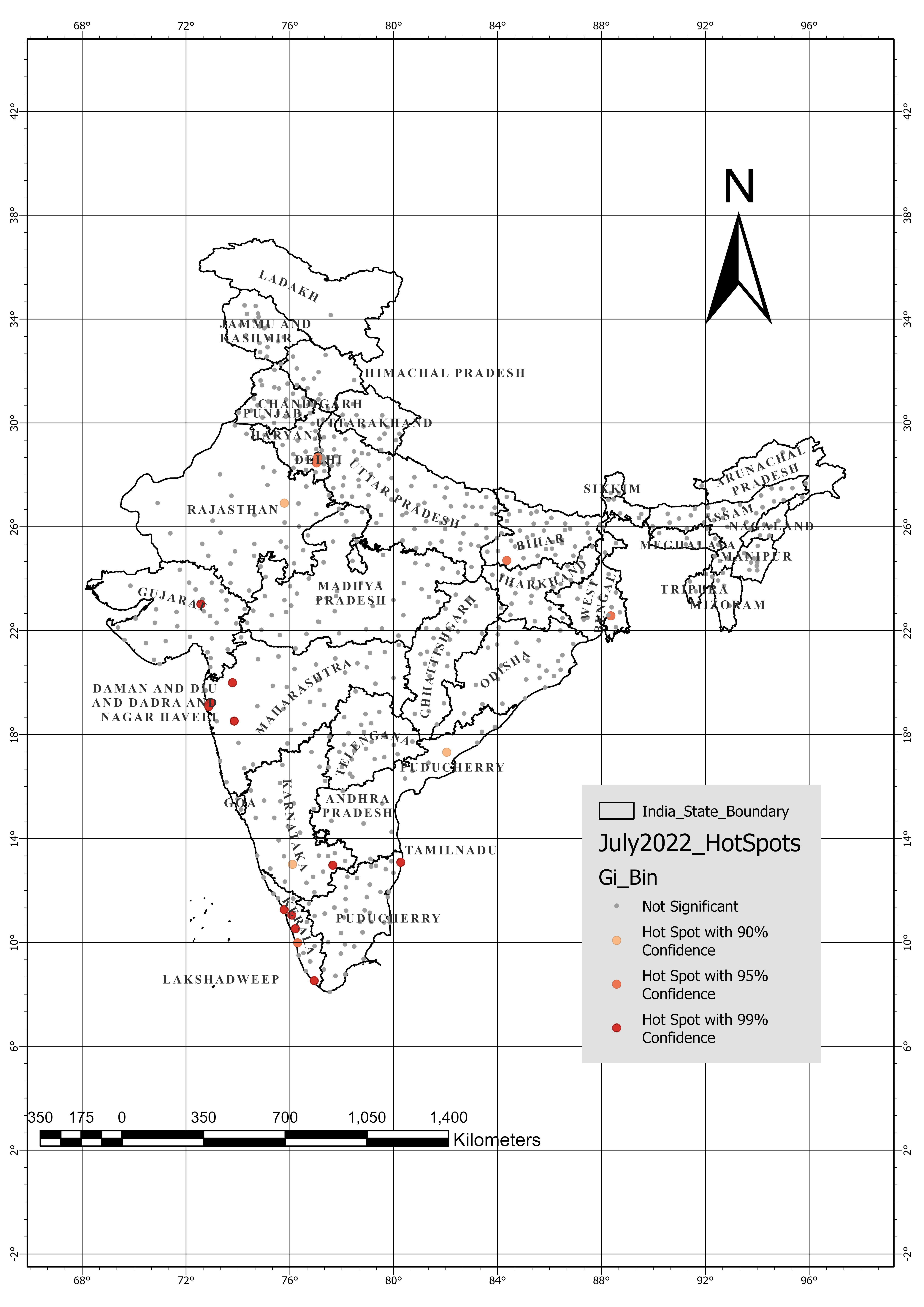

Supplement: Supplementary file 3 — Supplementary Information 3. [file 41598_2023_50933_MOESM3_ESM.zip › July 2022.jpg]

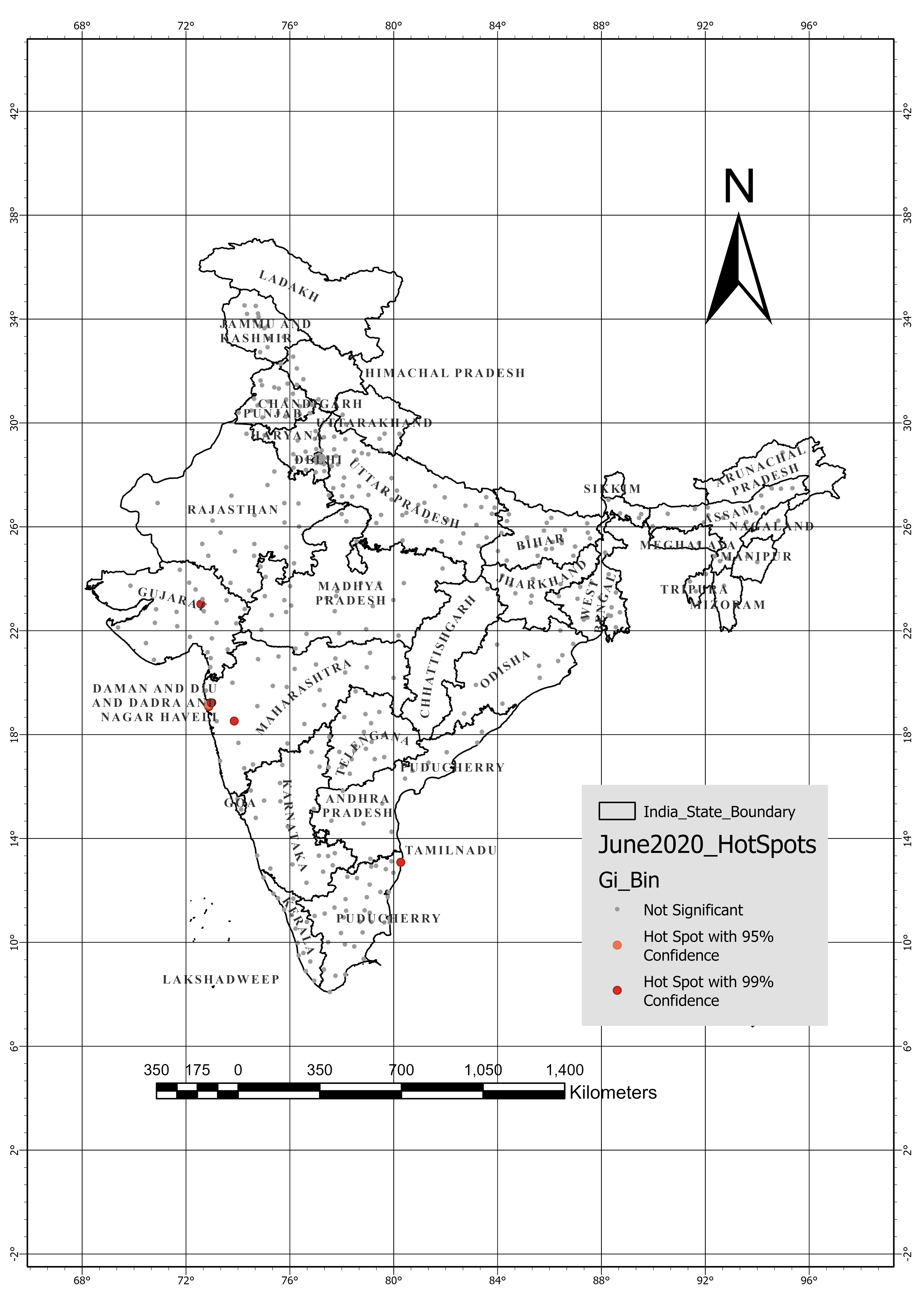

Supplement: Supplementary file 3 — Supplementary Information 3. [file 41598_2023_50933_MOESM3_ESM.zip › June 2020.jpg]

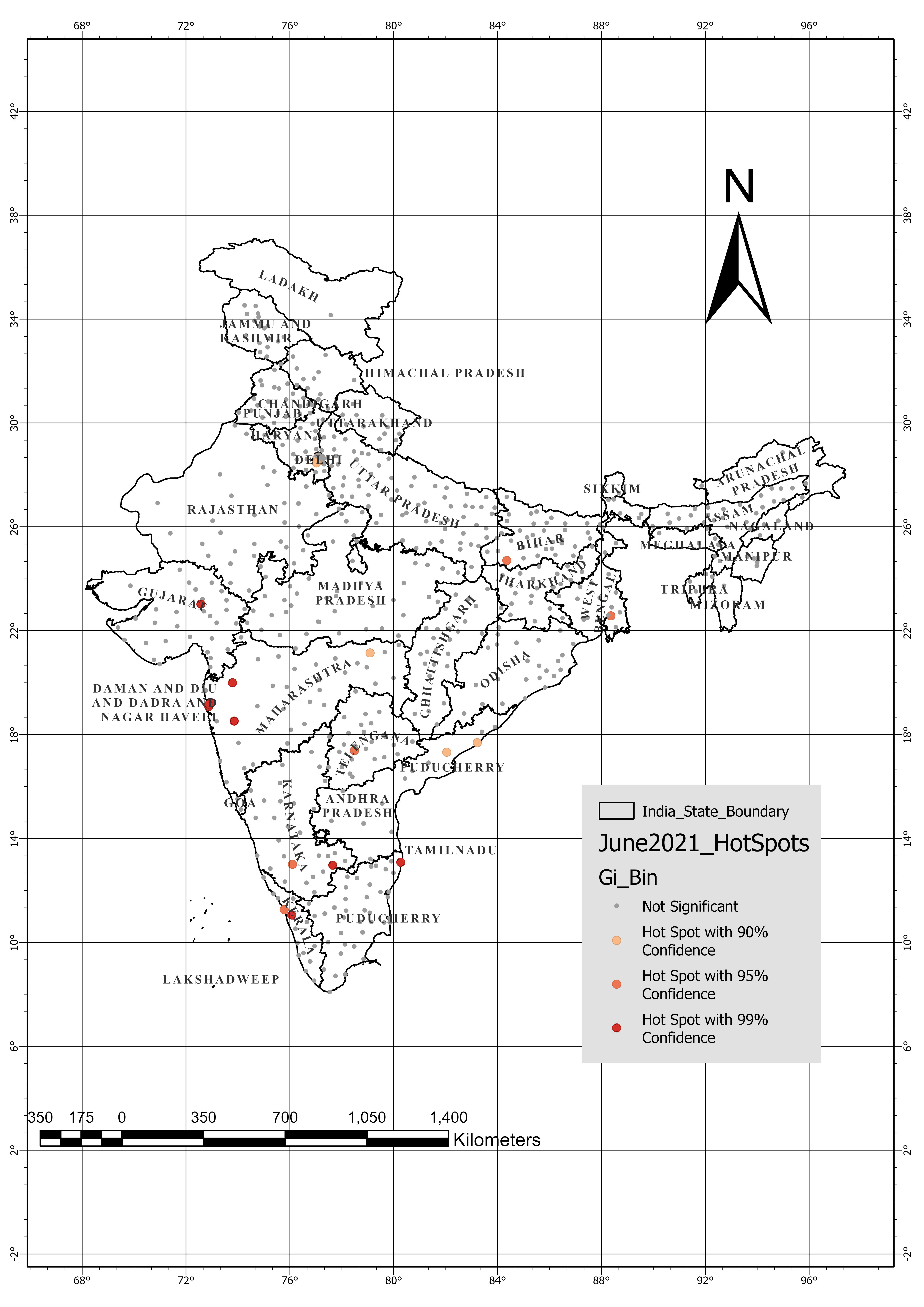

Supplement: Supplementary file 3 — Supplementary Information 3. [file 41598_2023_50933_MOESM3_ESM.zip › June 2021.jpg]

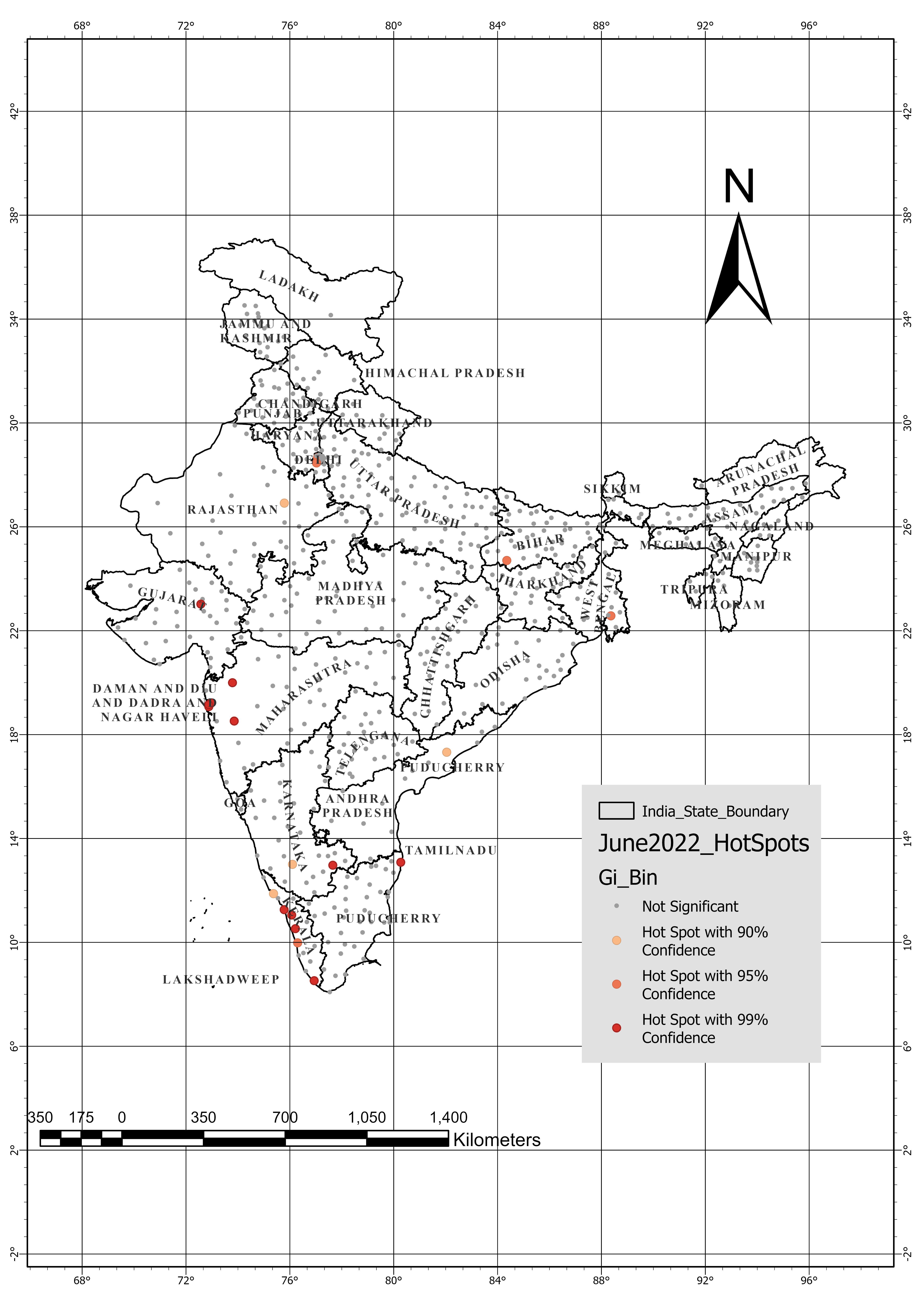

Supplement: Supplementary file 3 — Supplementary Information 3. [file 41598_2023_50933_MOESM3_ESM.zip › June 2022.jpg]

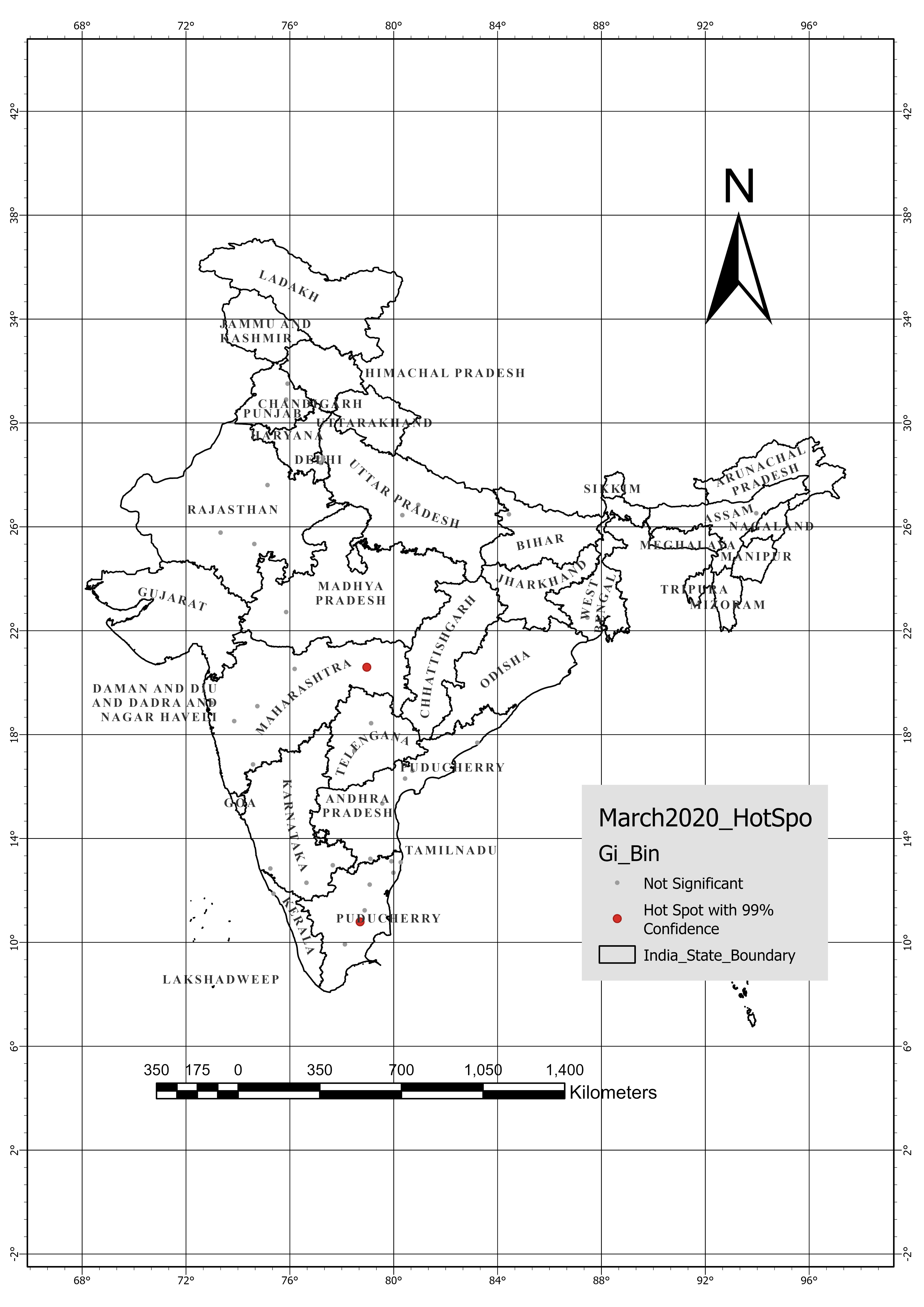

Supplement: Supplementary file 3 — Supplementary Information 3. [file 41598_2023_50933_MOESM3_ESM.zip › March 2020.jpg]

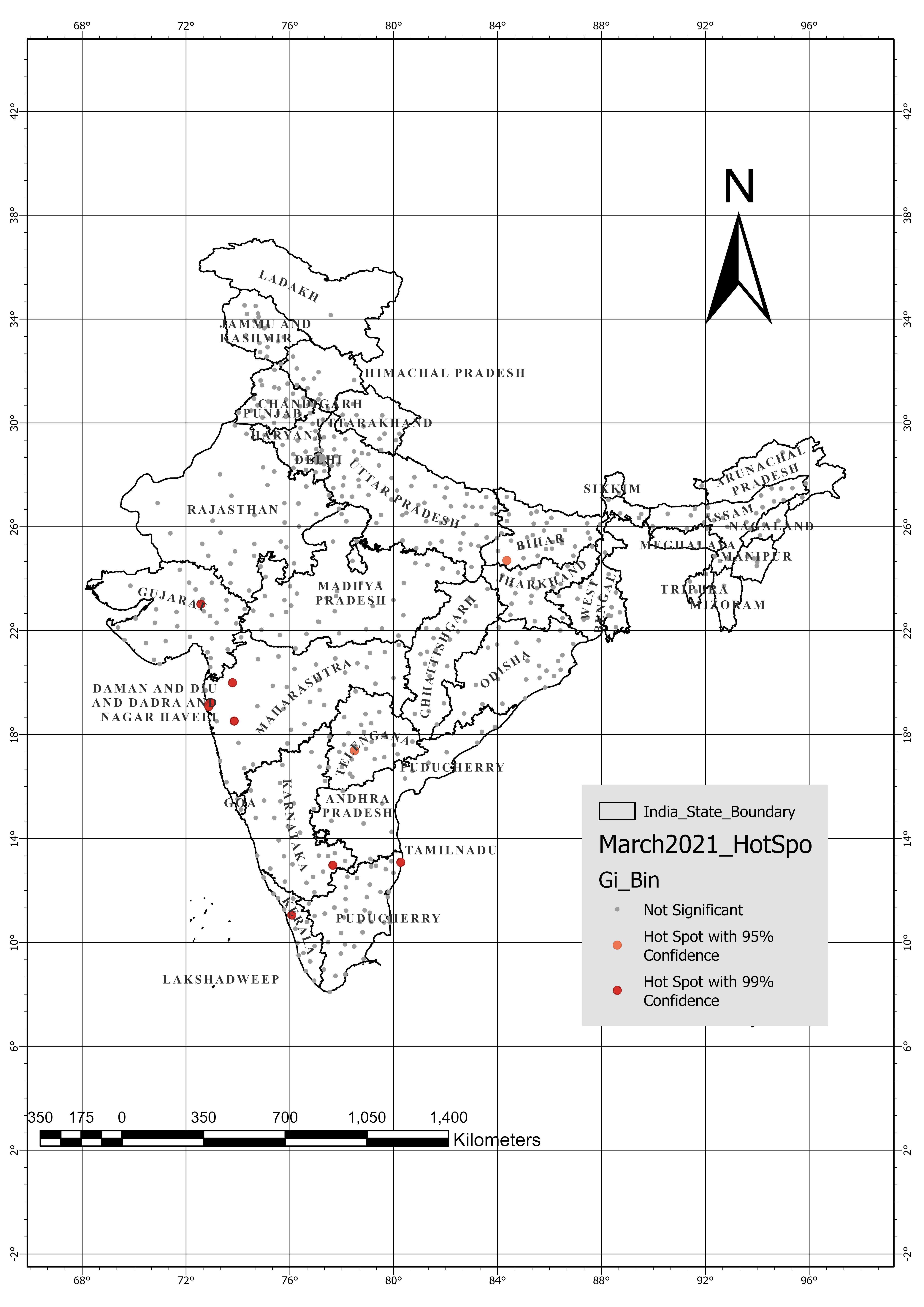

Supplement: Supplementary file 3 — Supplementary Information 3. [file 41598_2023_50933_MOESM3_ESM.zip › March 2021.jpg]

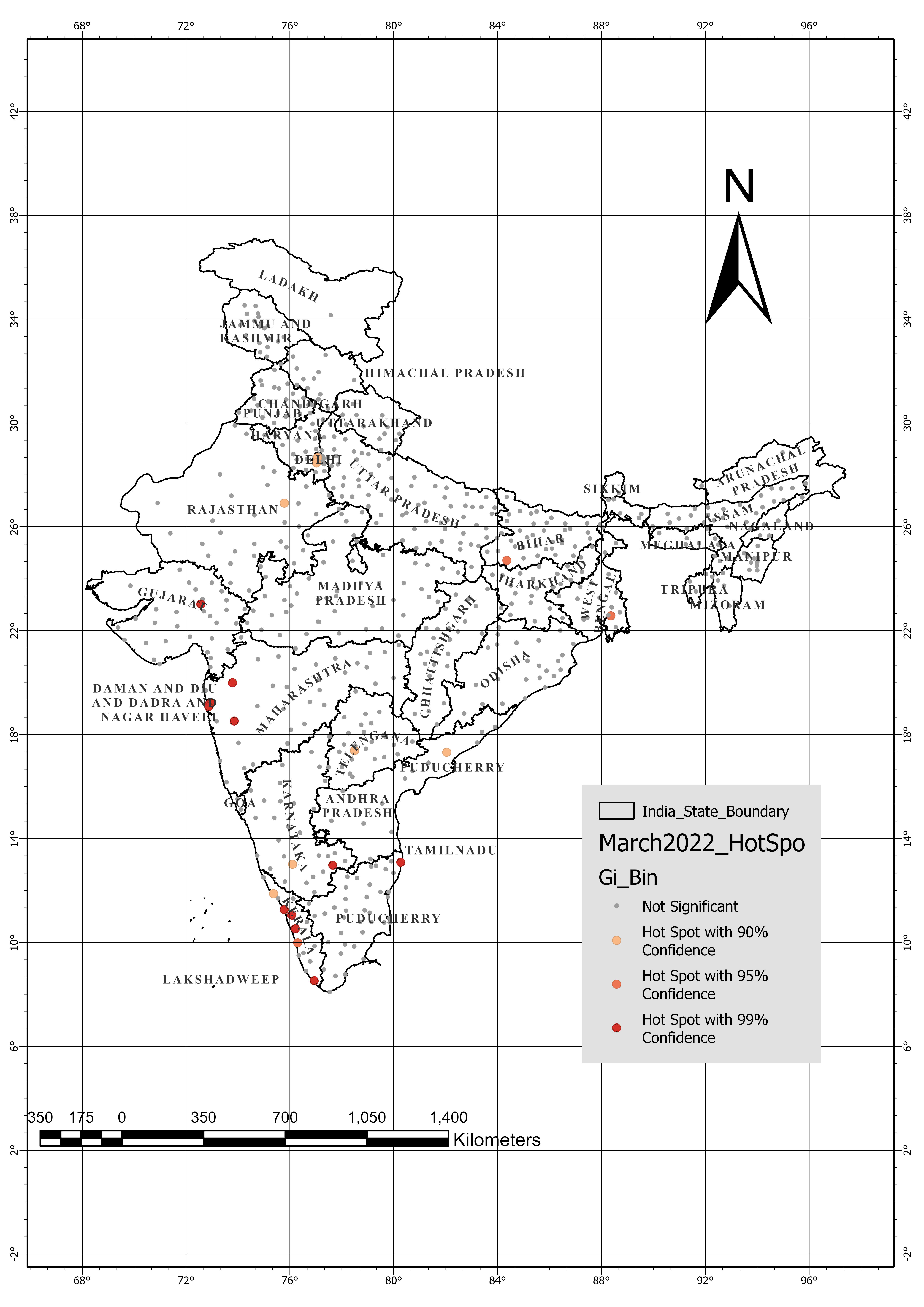

Supplement: Supplementary file 3 — Supplementary Information 3. [file 41598_2023_50933_MOESM3_ESM.zip › March 2022.jpg]

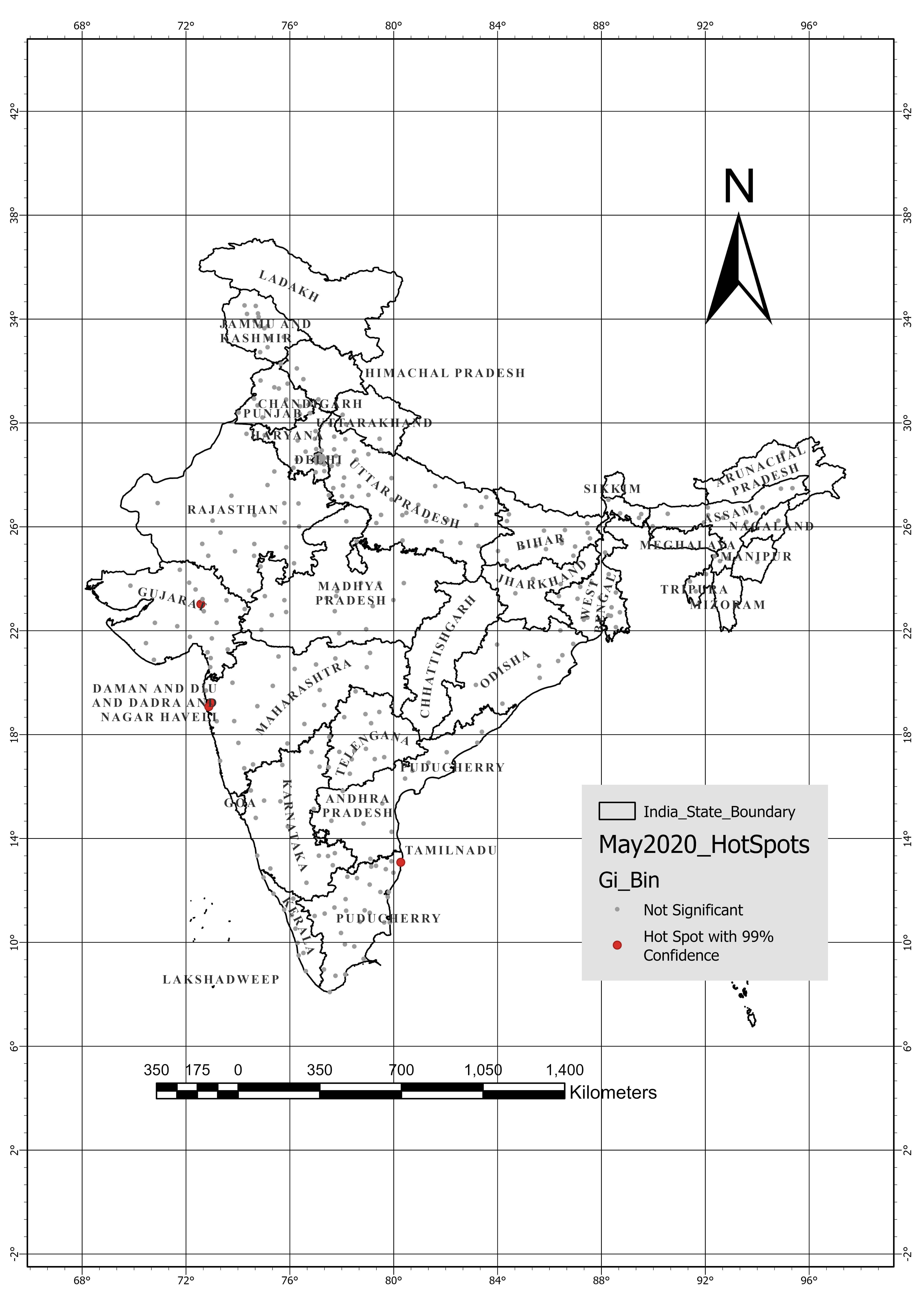

Supplement: Supplementary file 3 — Supplementary Information 3. [file 41598_2023_50933_MOESM3_ESM.zip › May 2020.jpg]

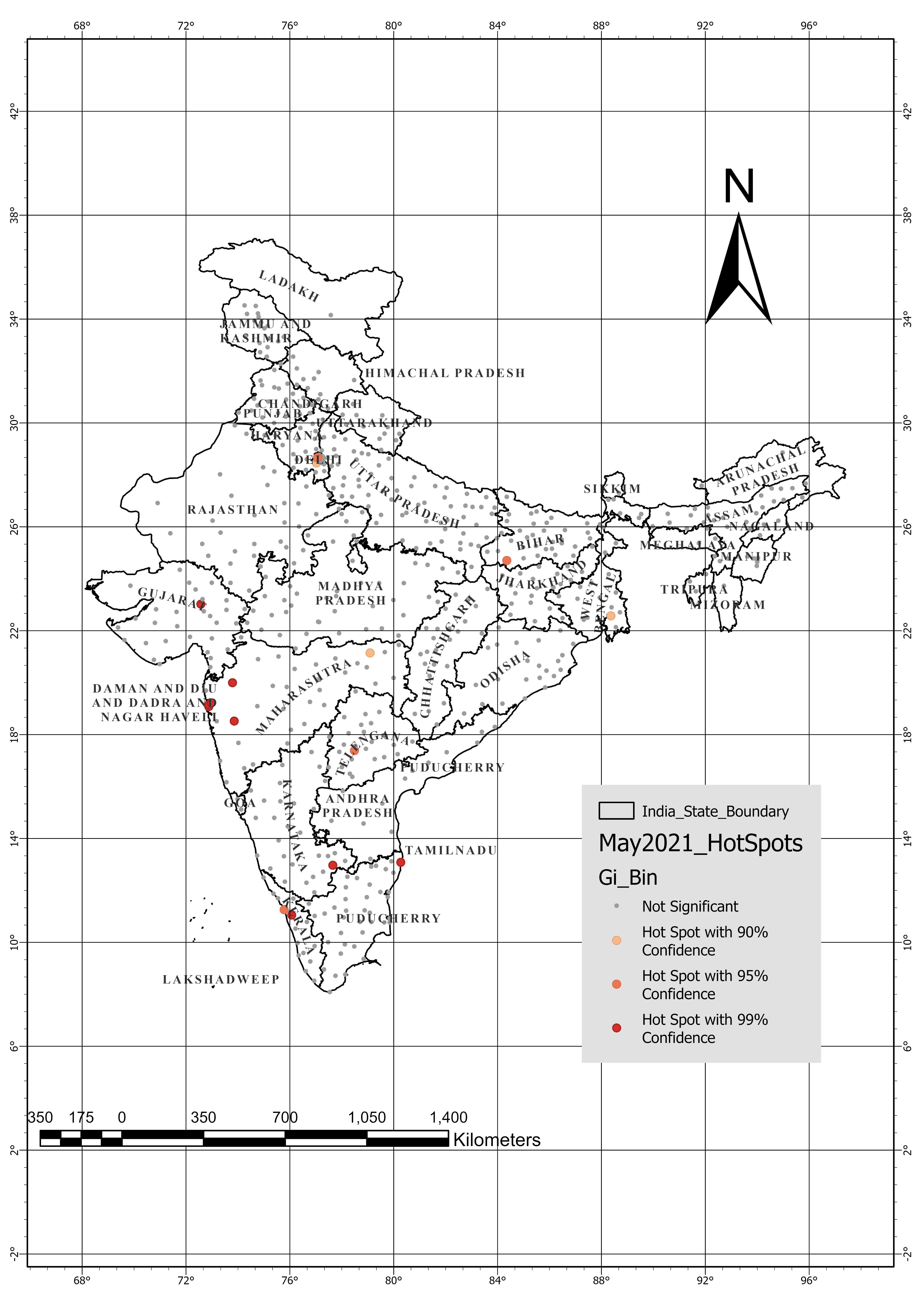

Supplement: Supplementary file 3 — Supplementary Information 3. [file 41598_2023_50933_MOESM3_ESM.zip › May 2021.jpg]

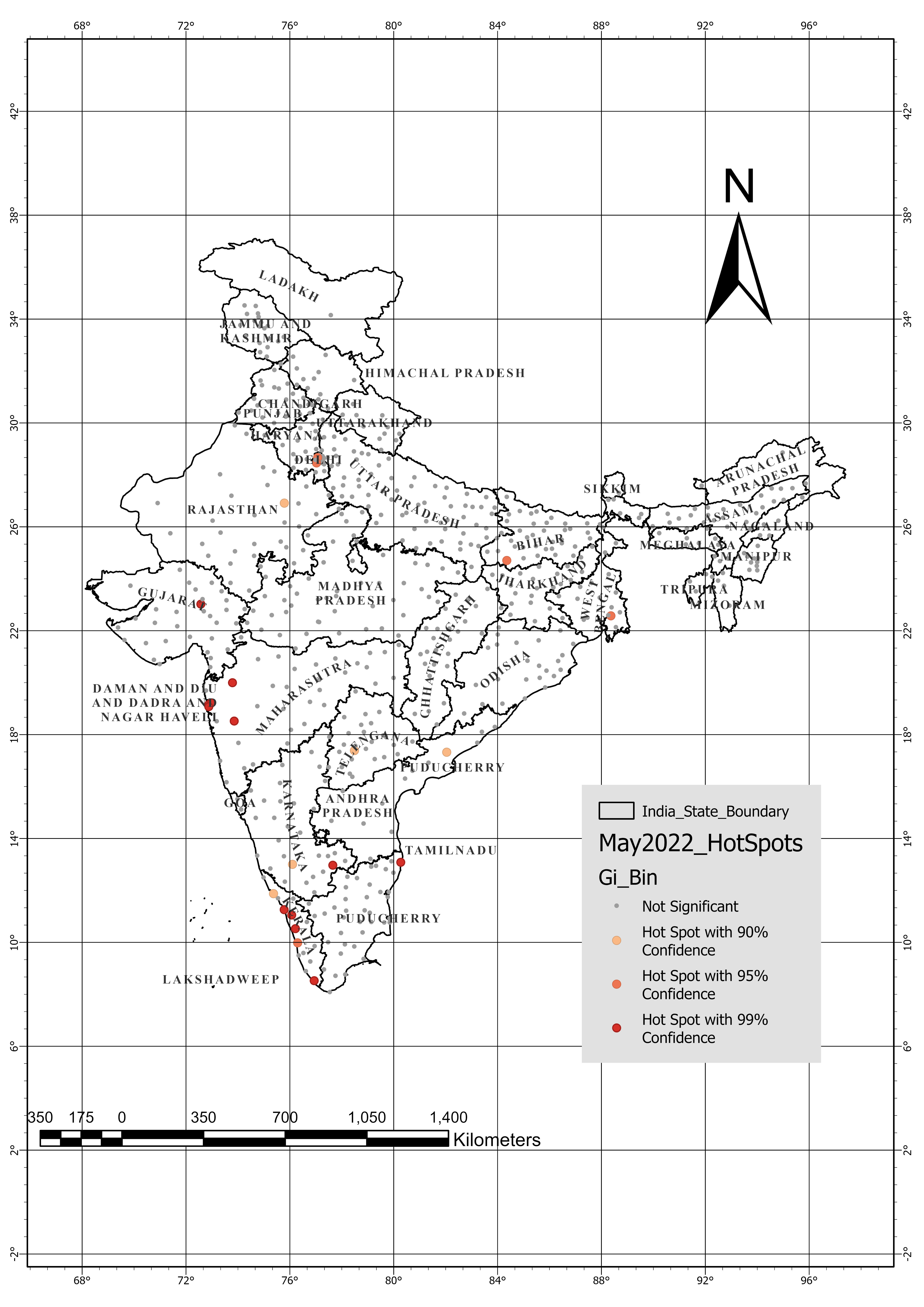

Supplement: Supplementary file 3 — Supplementary Information 3. [file 41598_2023_50933_MOESM3_ESM.zip › May 2022.jpg]

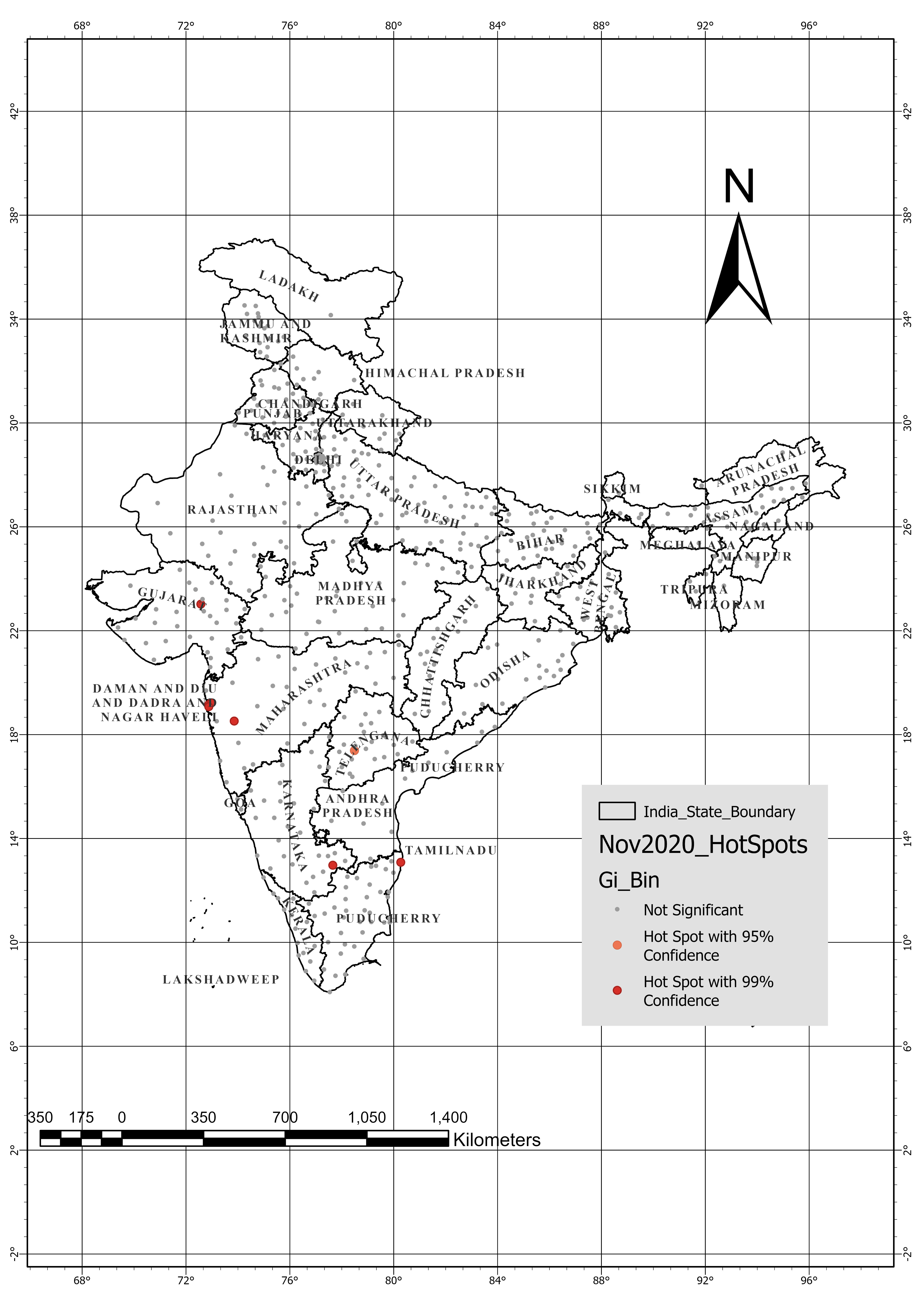

Supplement: Supplementary file 3 — Supplementary Information 3. [file 41598_2023_50933_MOESM3_ESM.zip › November 2020.jpg]

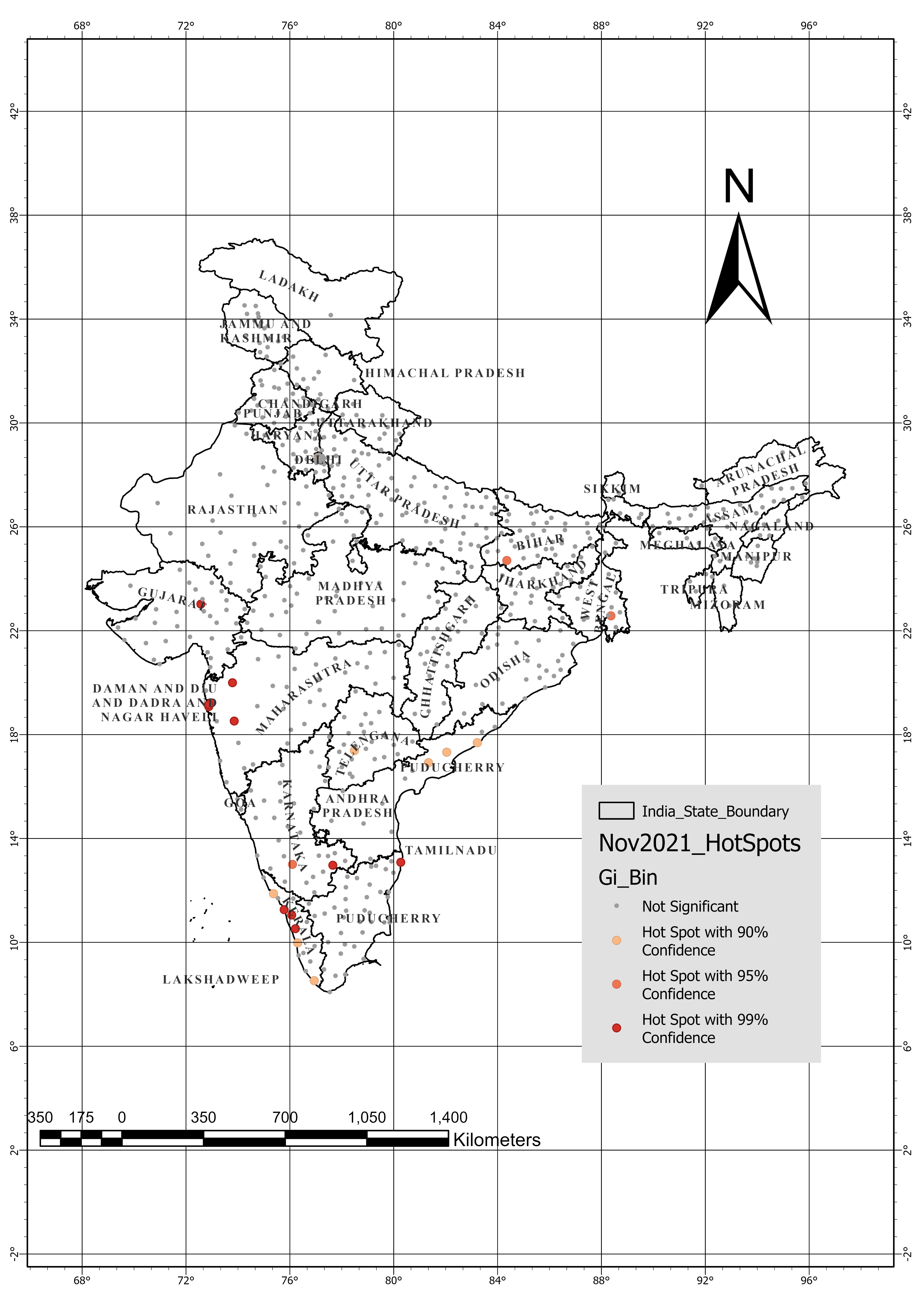

Supplement: Supplementary file 3 — Supplementary Information 3. [file 41598_2023_50933_MOESM3_ESM.zip › November 2021.jpg]

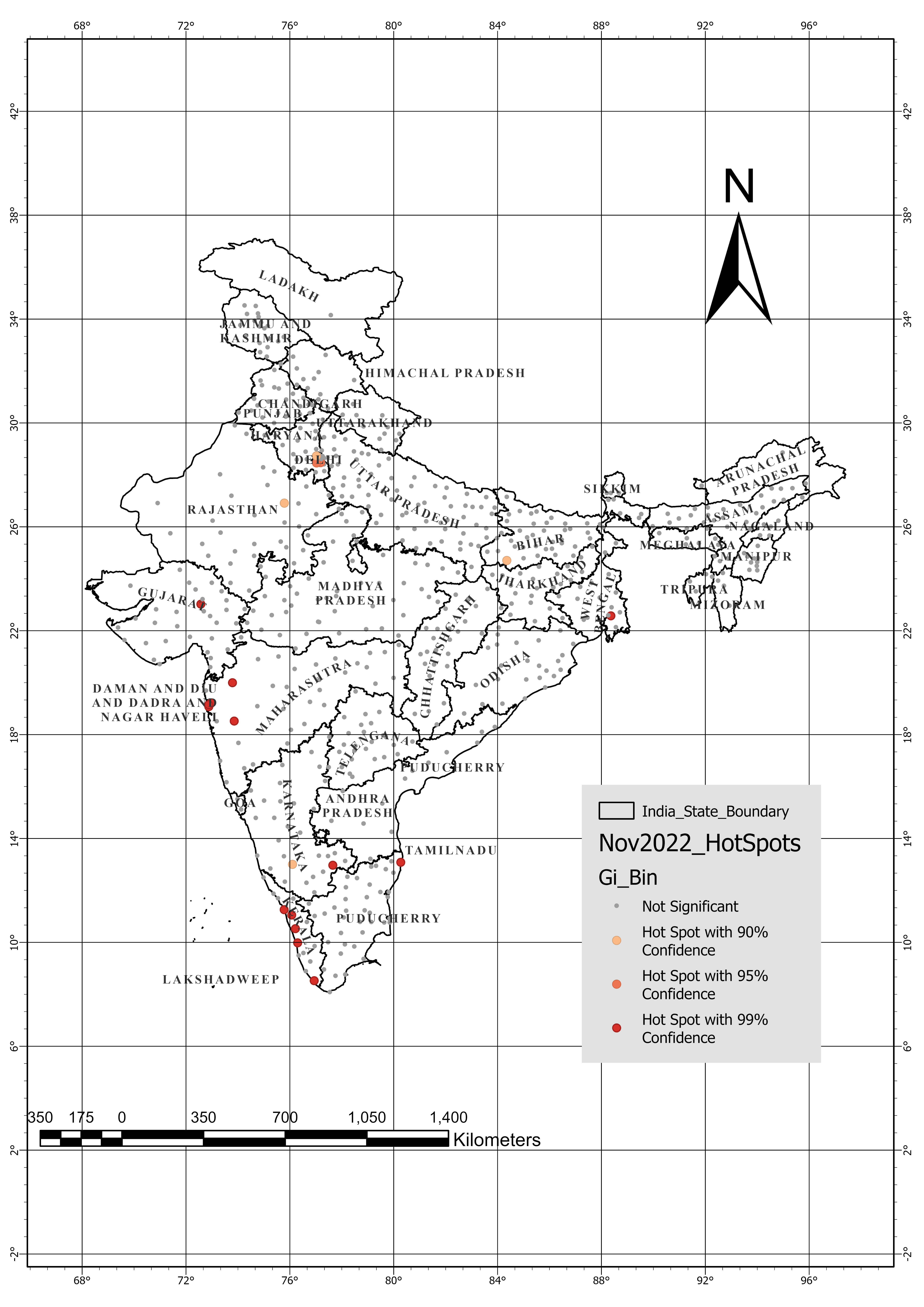

Supplement: Supplementary file 3 — Supplementary Information 3. [file 41598_2023_50933_MOESM3_ESM.zip › November 2022.jpg]

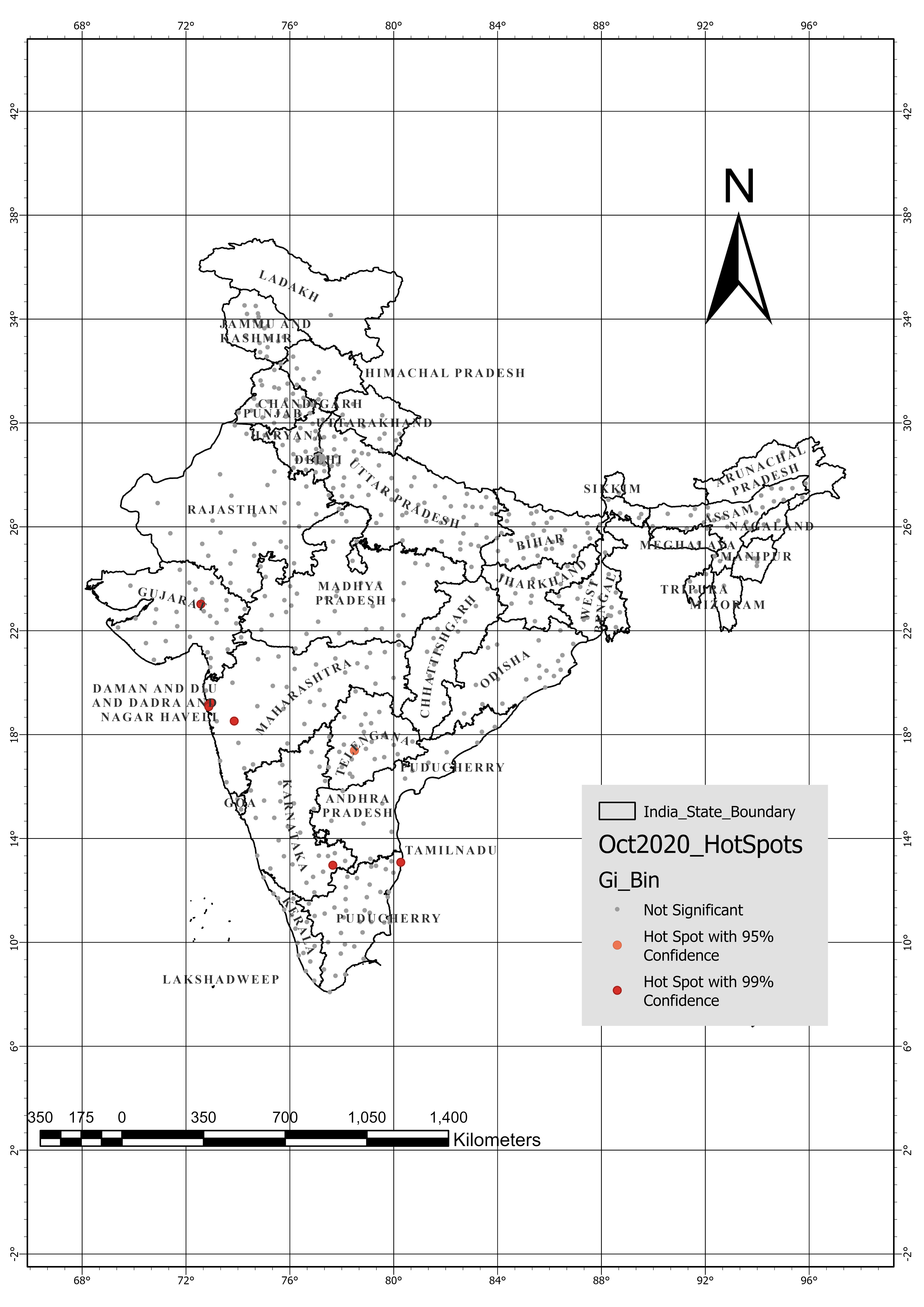

Supplement: Supplementary file 3 — Supplementary Information 3. [file 41598_2023_50933_MOESM3_ESM.zip › October 2020.jpg]

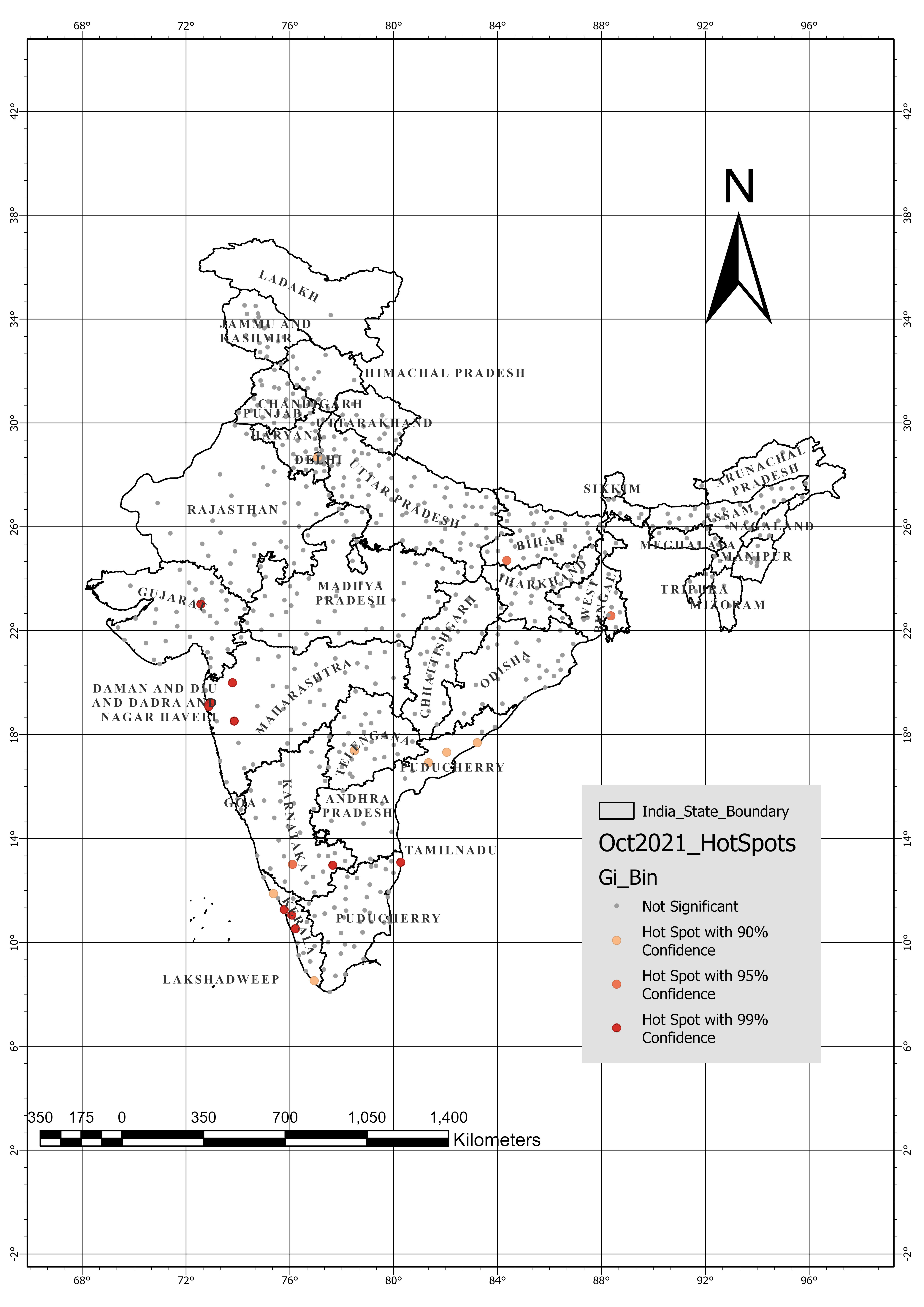

Supplement: Supplementary file 3 — Supplementary Information 3. [file 41598_2023_50933_MOESM3_ESM.zip › October 2021.jpg]

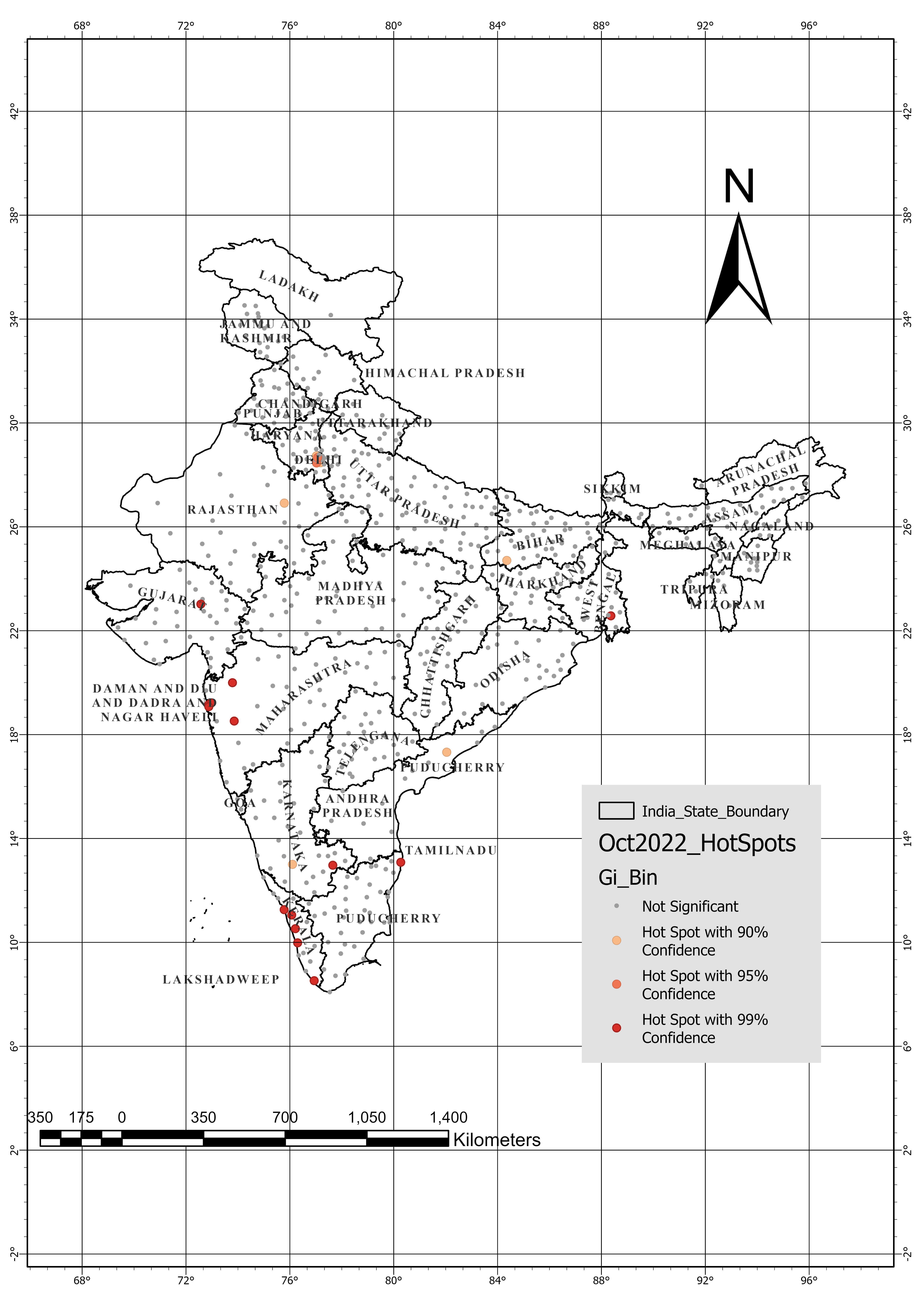

Supplement: Supplementary file 3 — Supplementary Information 3. [file 41598_2023_50933_MOESM3_ESM.zip › October 2022.jpg]
