# Supplementary figures and images for: Tracing the COVID-19 spread pattern in India through a GIS-based spatio-temporal analysis of interconnected clusters (part 2 of 2)
Source: Sci Rep. 2024 Jan 8;14:847. doi: 10.1038/s41598-023-50933-4 (PMC10774287; doi:10.1038/s41598-023-50933-4)

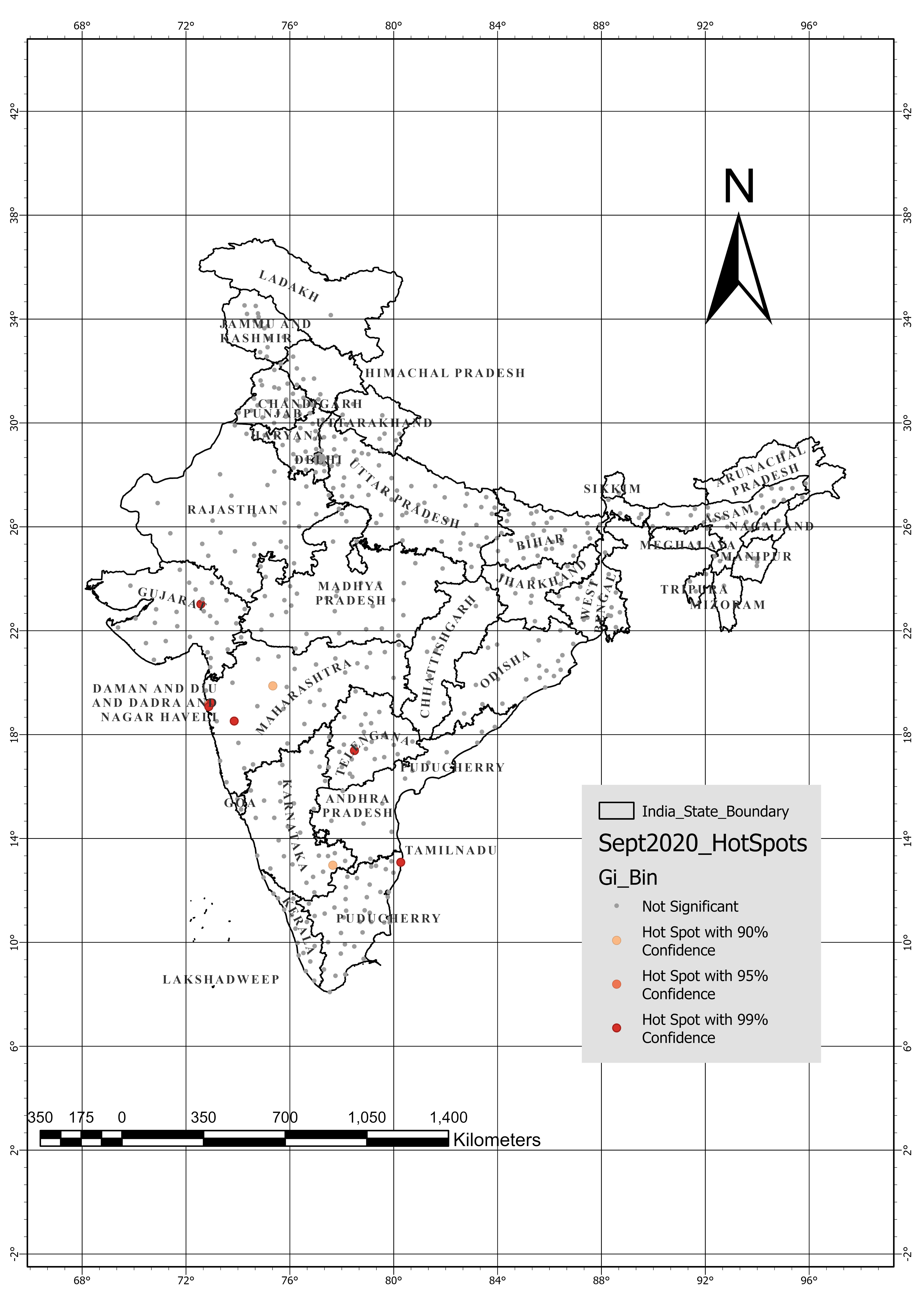

Supplement: Supplementary file 3 — Supplementary Information 3. [file 41598_2023_50933_MOESM3_ESM.zip › September 2020.jpg]

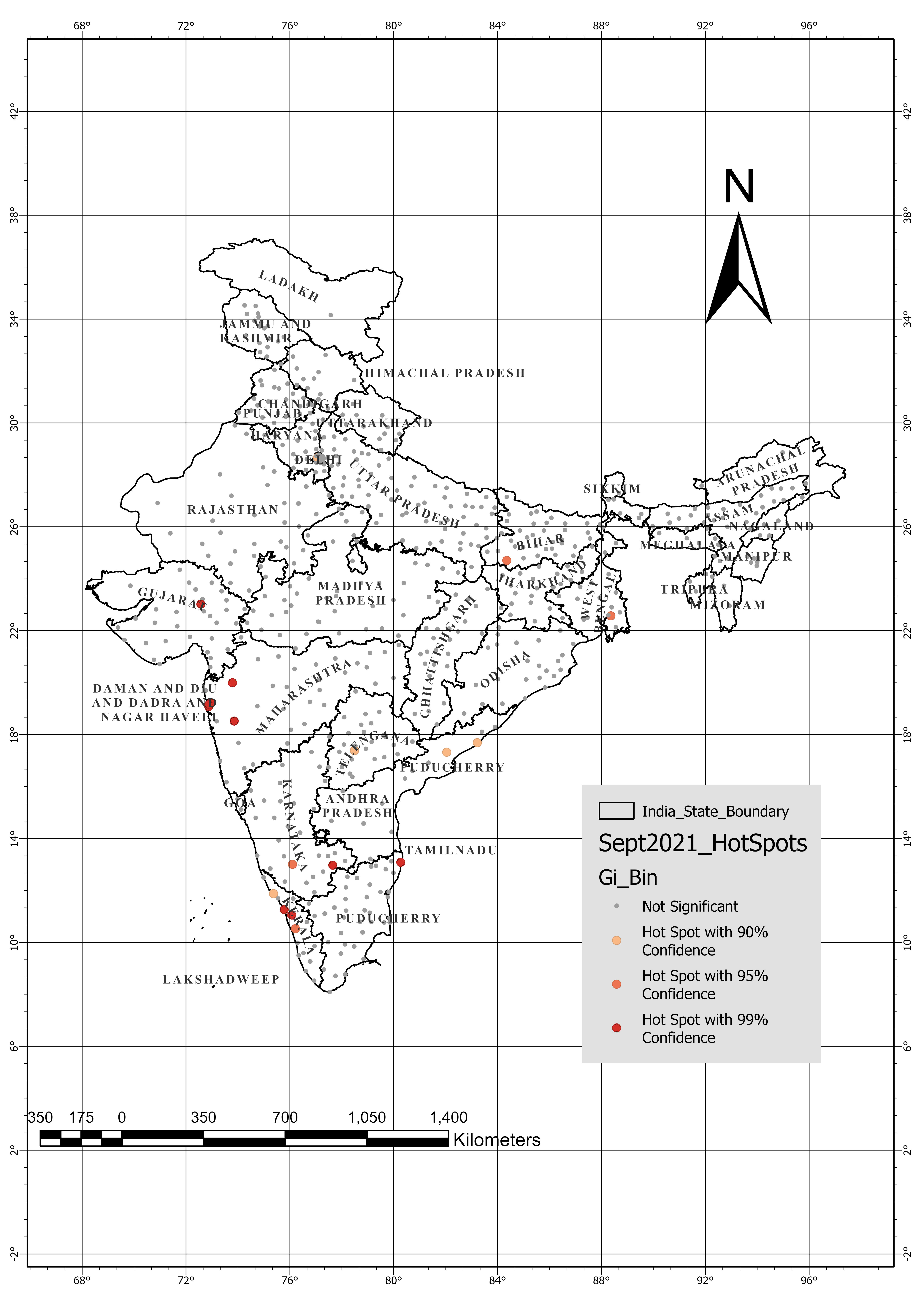

Supplement: Supplementary file 3 — Supplementary Information 3. [file 41598_2023_50933_MOESM3_ESM.zip › September 2021.jpg]

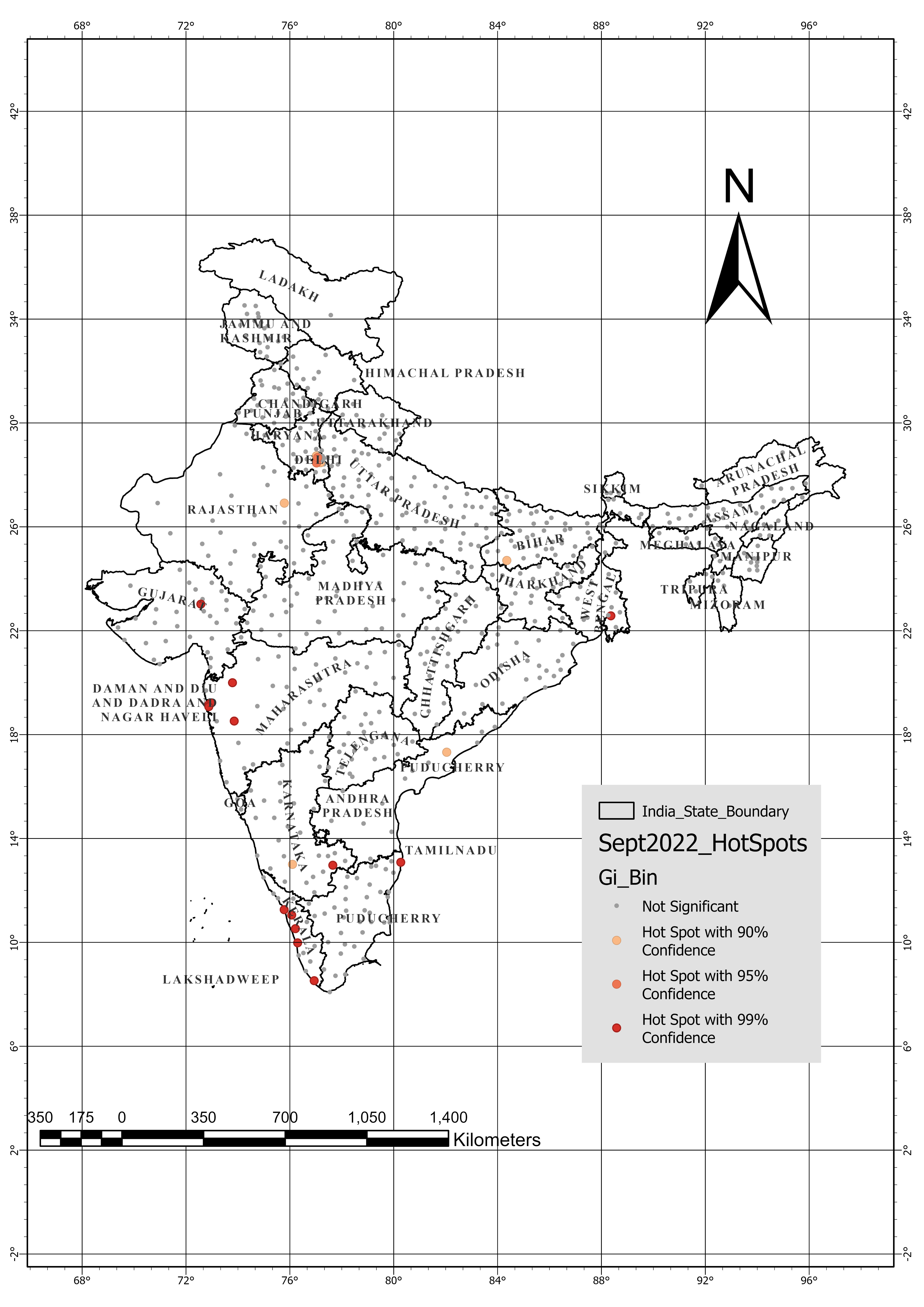

Supplement: Supplementary file 3 — Supplementary Information 3. [file 41598_2023_50933_MOESM3_ESM.zip › September 2022.jpg]

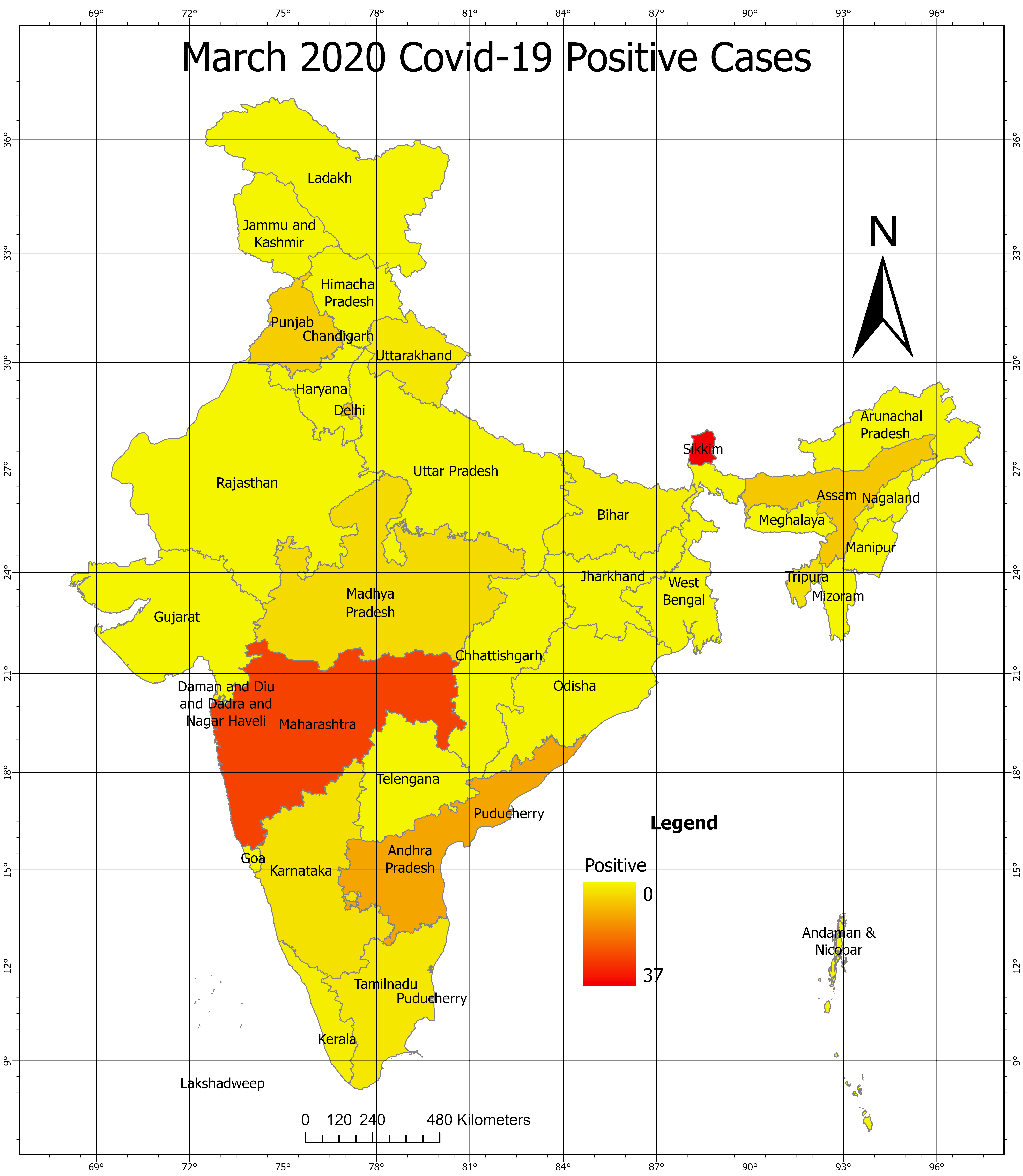

Supplement: Supplementary file 4 — Supplementary Information 4. [file 41598_2023_50933_MOESM4_ESM.zip › a_March 2020.png]

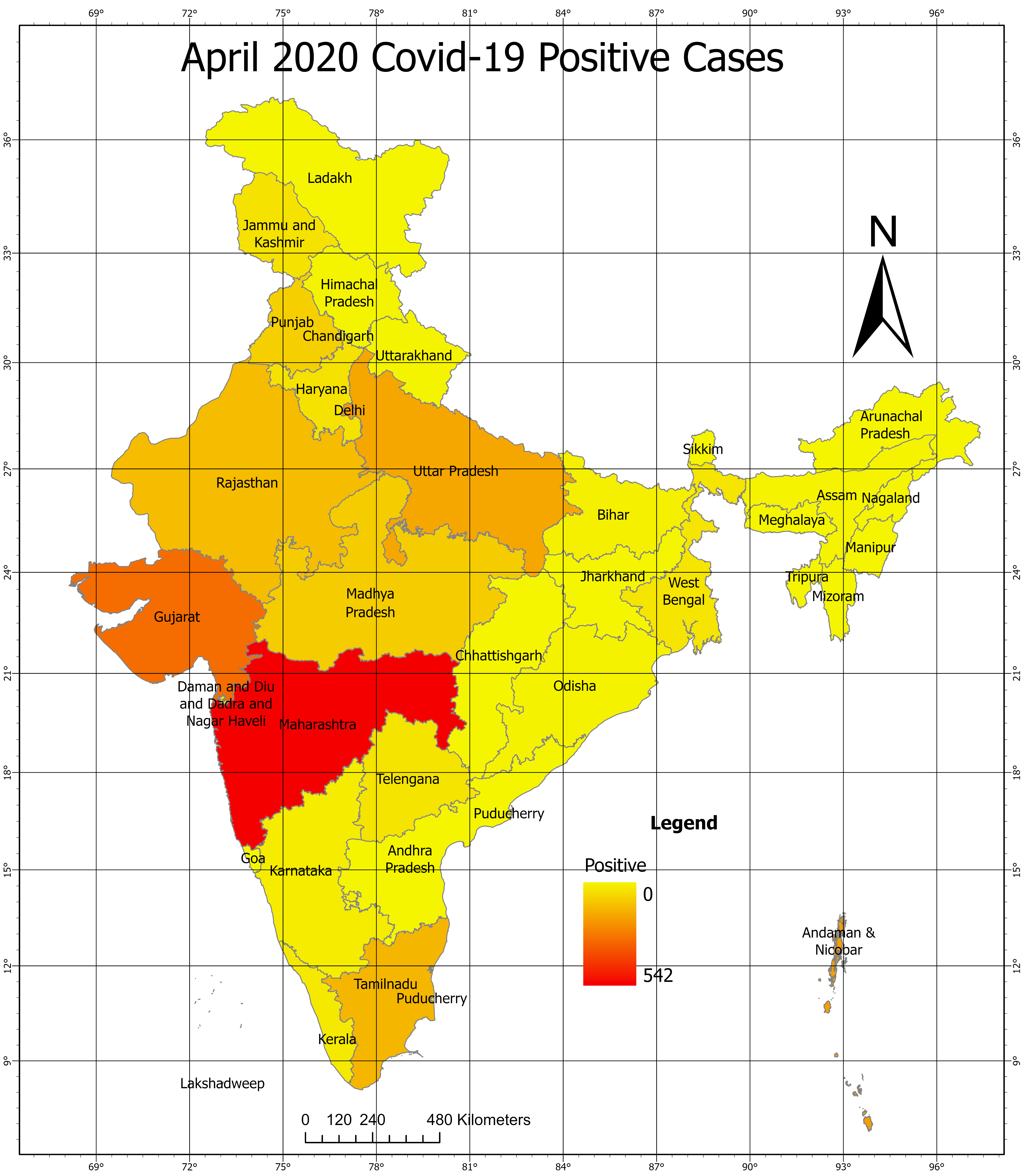

Supplement: Supplementary file 4 — Supplementary Information 4. [file 41598_2023_50933_MOESM4_ESM.zip › b_April 2020.png]

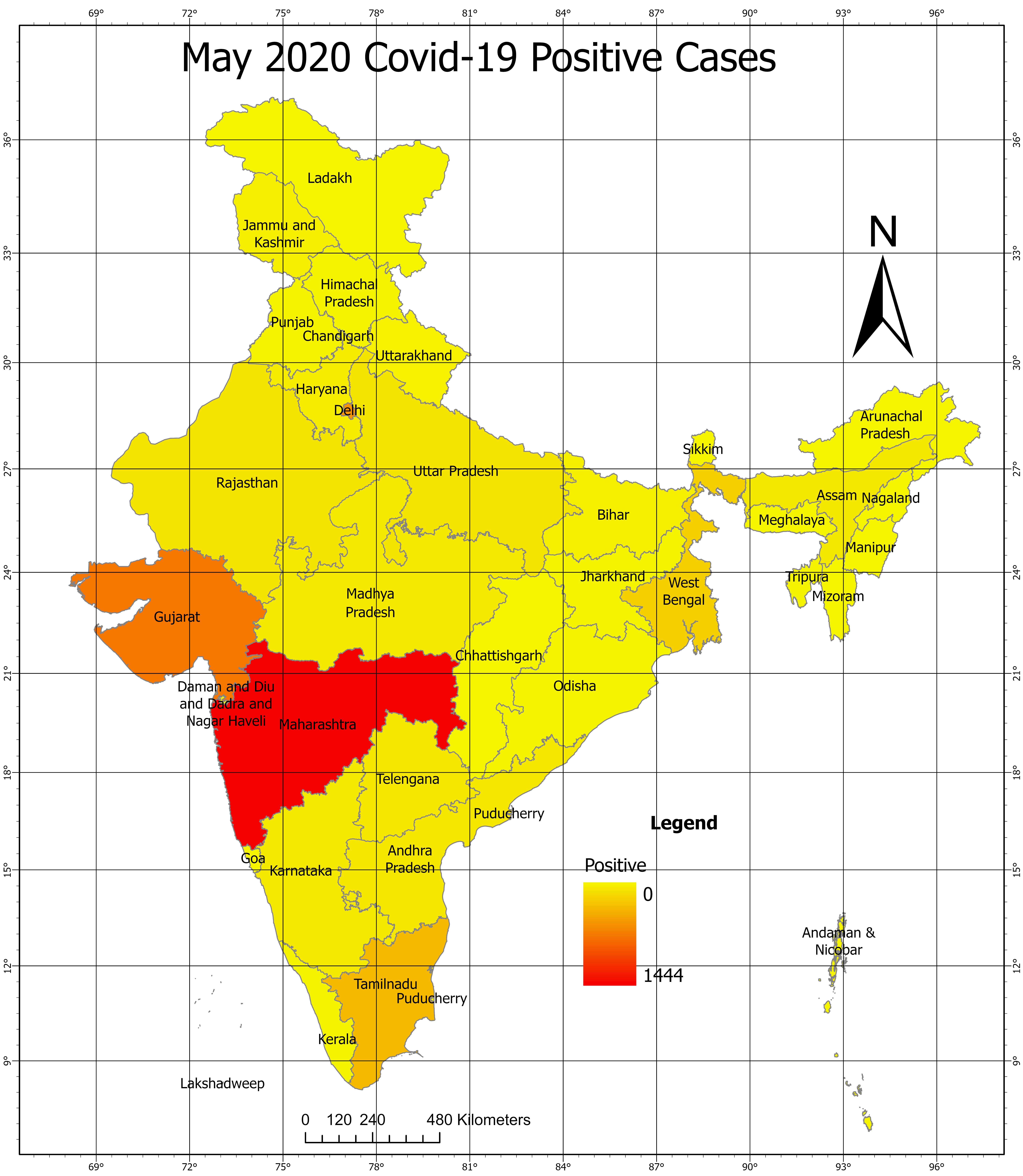

Supplement: Supplementary file 4 — Supplementary Information 4. [file 41598_2023_50933_MOESM4_ESM.zip › c_May 2020.png]

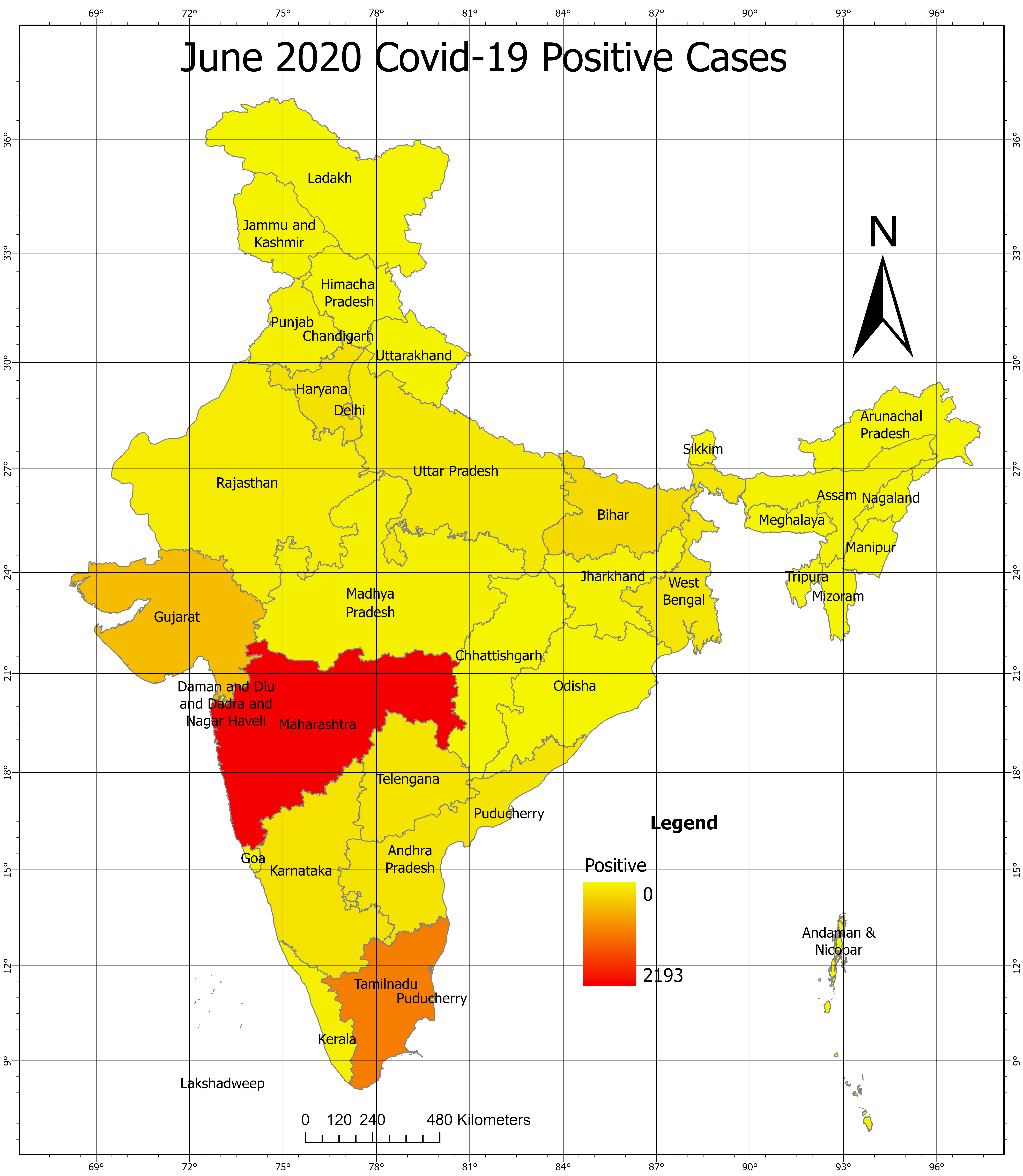

Supplement: Supplementary file 4 — Supplementary Information 4. [file 41598_2023_50933_MOESM4_ESM.zip › d_June 2020.png]

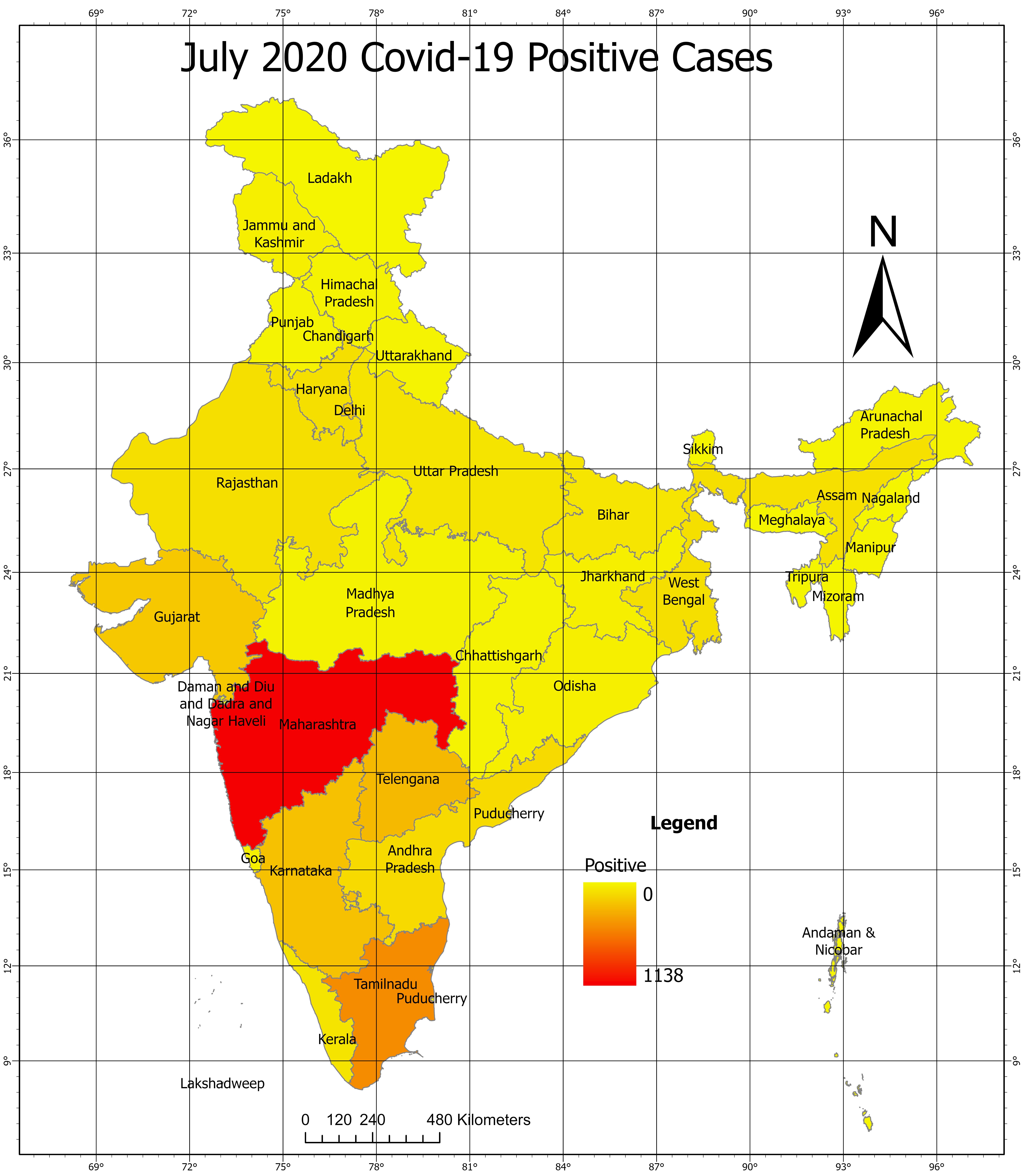

Supplement: Supplementary file 4 — Supplementary Information 4. [file 41598_2023_50933_MOESM4_ESM.zip › e_July 2020.png]

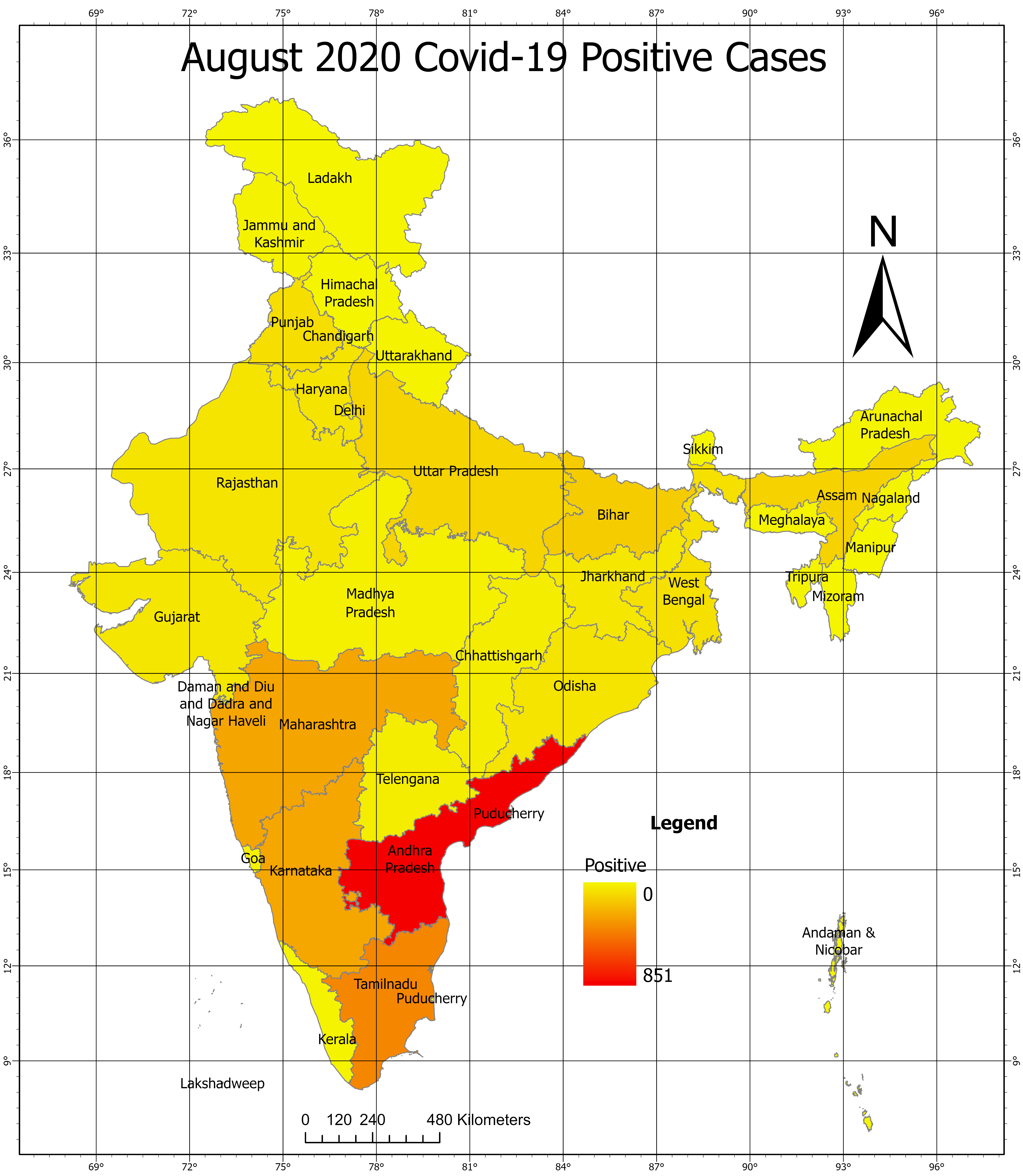

Supplement: Supplementary file 4 — Supplementary Information 4. [file 41598_2023_50933_MOESM4_ESM.zip › f_August 2020.png]

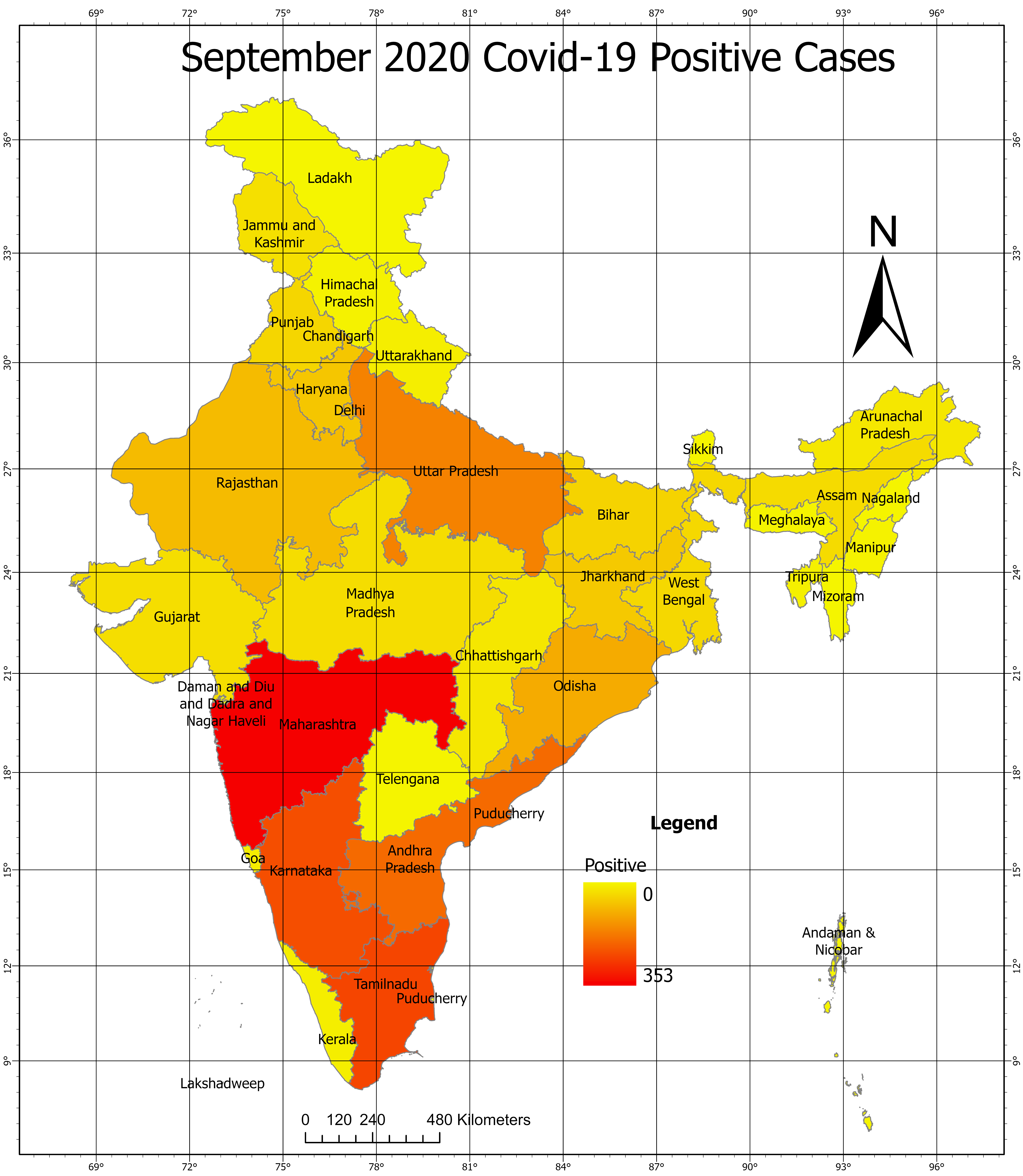

Supplement: Supplementary file 4 — Supplementary Information 4. [file 41598_2023_50933_MOESM4_ESM.zip › g_Sept 2020.png]

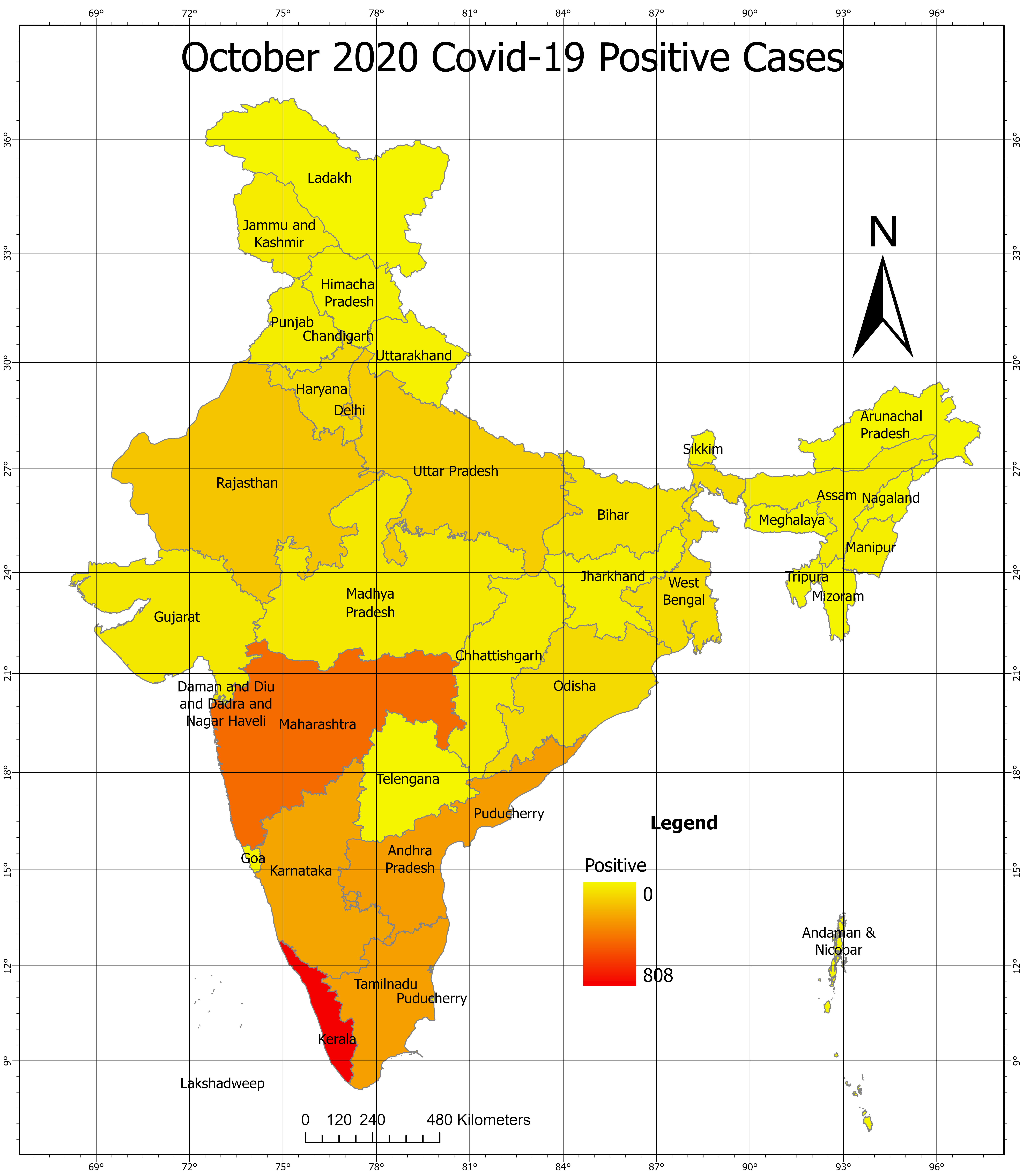

Supplement: Supplementary file 4 — Supplementary Information 4. [file 41598_2023_50933_MOESM4_ESM.zip › h_Oct 2020.png]

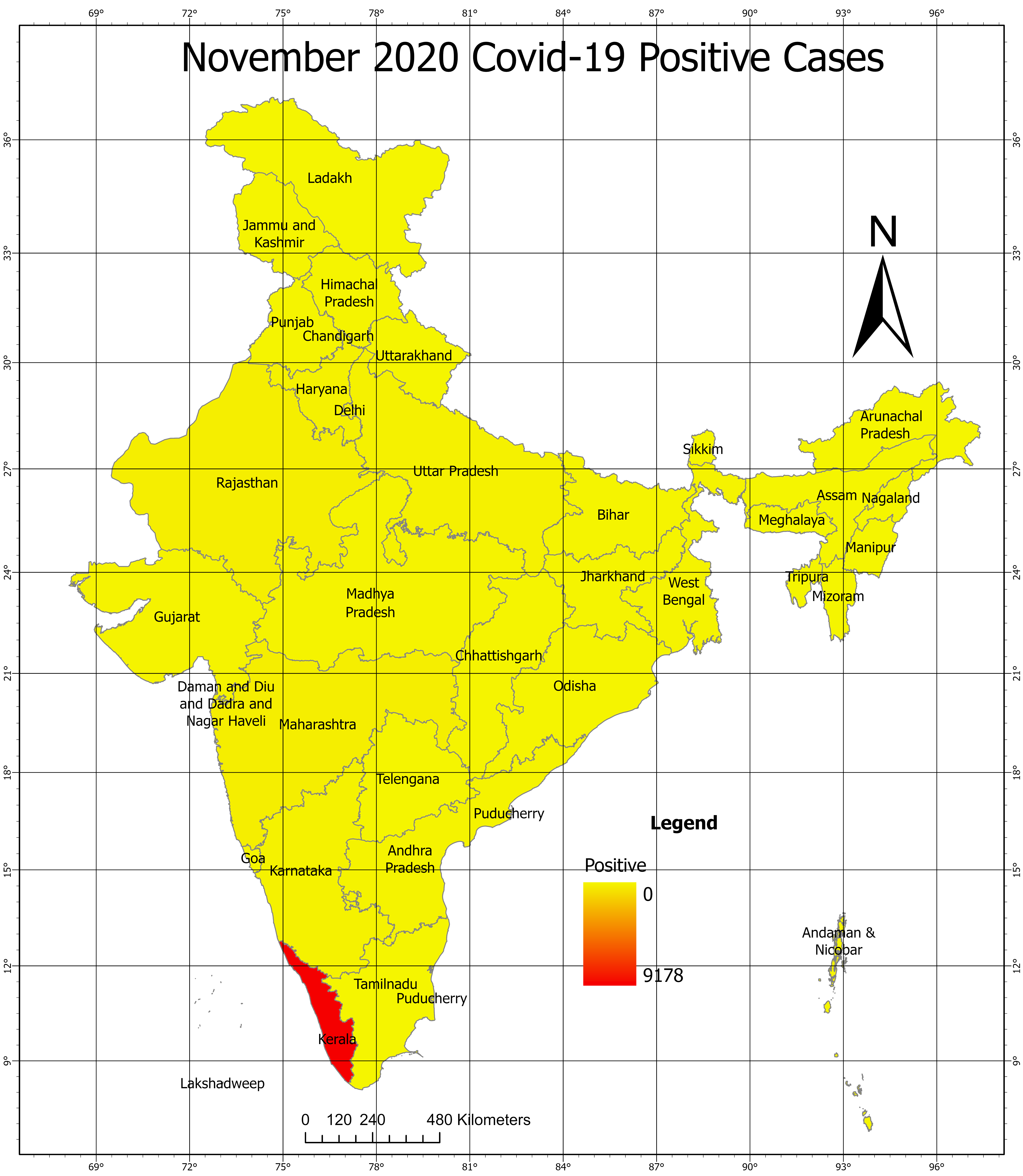

Supplement: Supplementary file 4 — Supplementary Information 4. [file 41598_2023_50933_MOESM4_ESM.zip › i_Nov 2020.png]

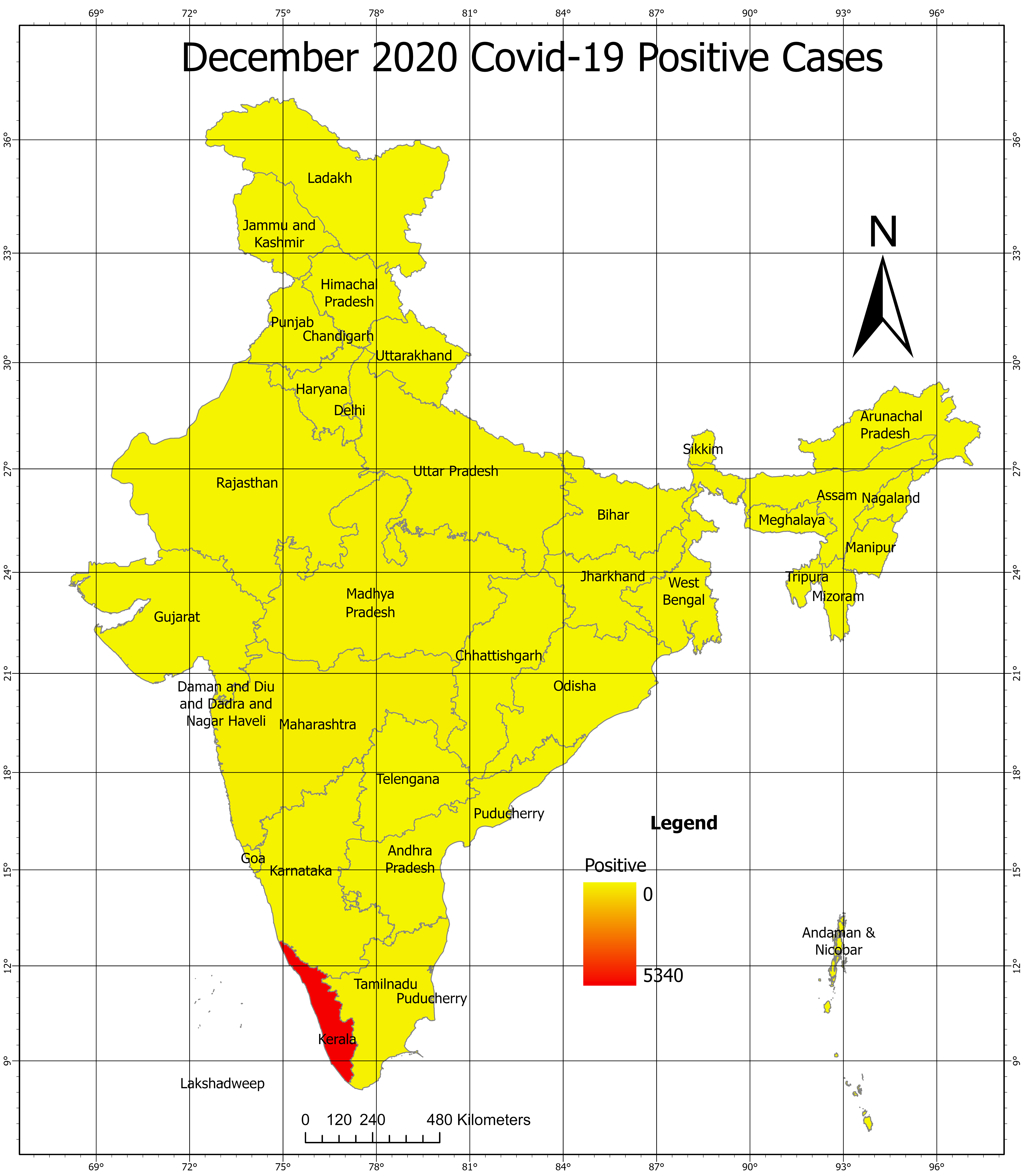

Supplement: Supplementary file 4 — Supplementary Information 4. [file 41598_2023_50933_MOESM4_ESM.zip › j_Dec 2020.png]

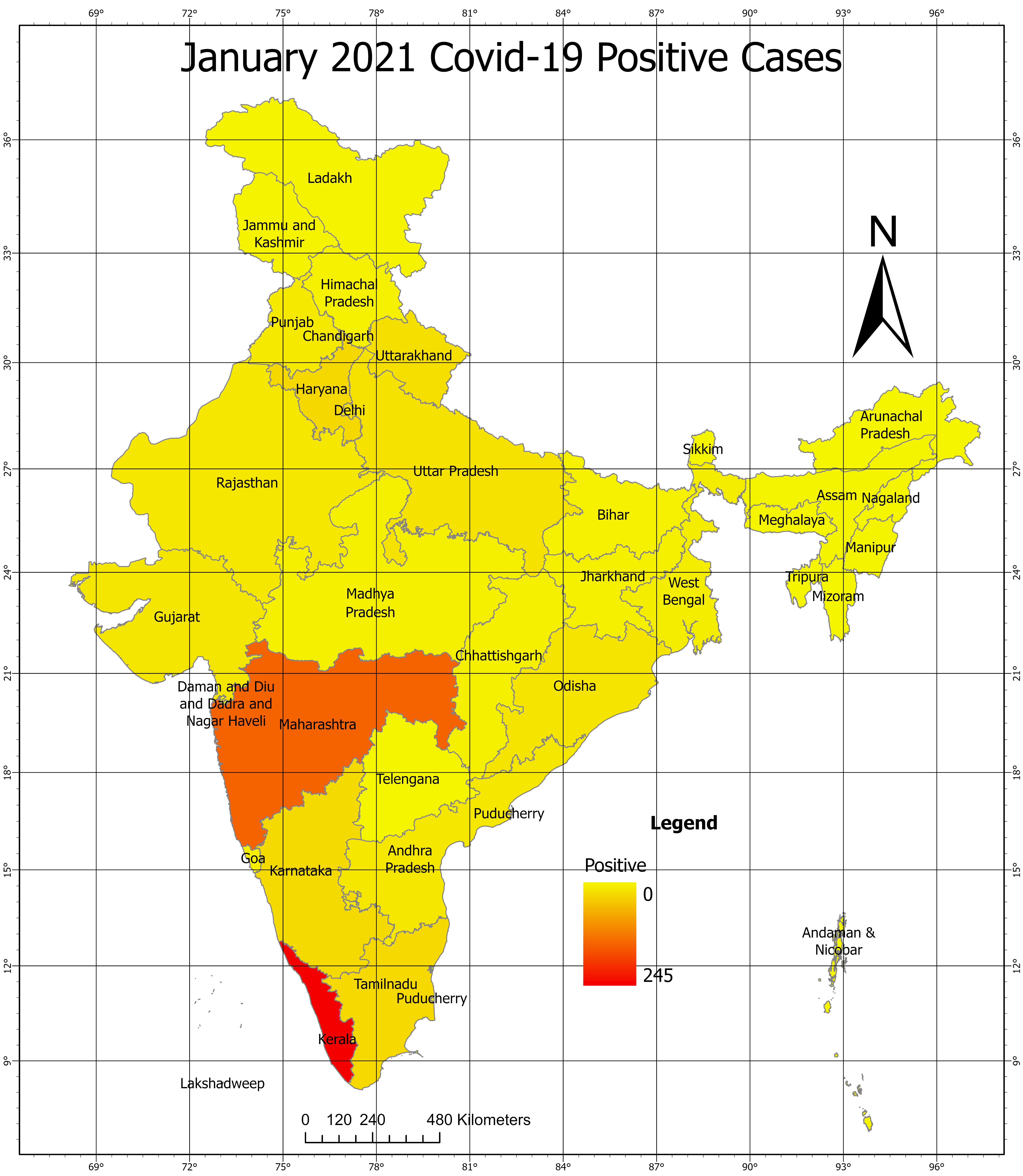

Supplement: Supplementary file 4 — Supplementary Information 4. [file 41598_2023_50933_MOESM4_ESM.zip › k_Jan 2021.png]

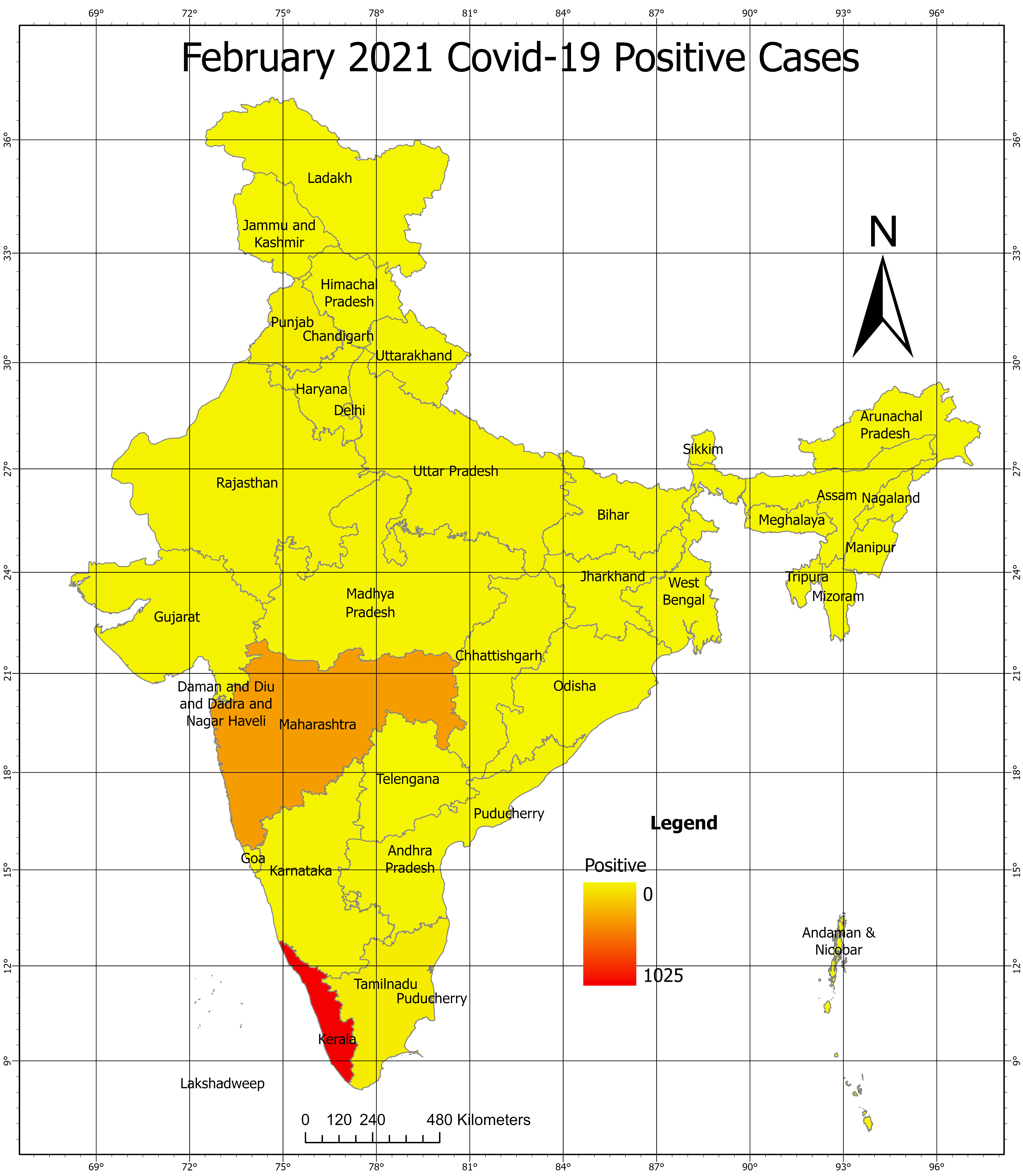

Supplement: Supplementary file 4 — Supplementary Information 4. [file 41598_2023_50933_MOESM4_ESM.zip › l_Feb 2021.png]

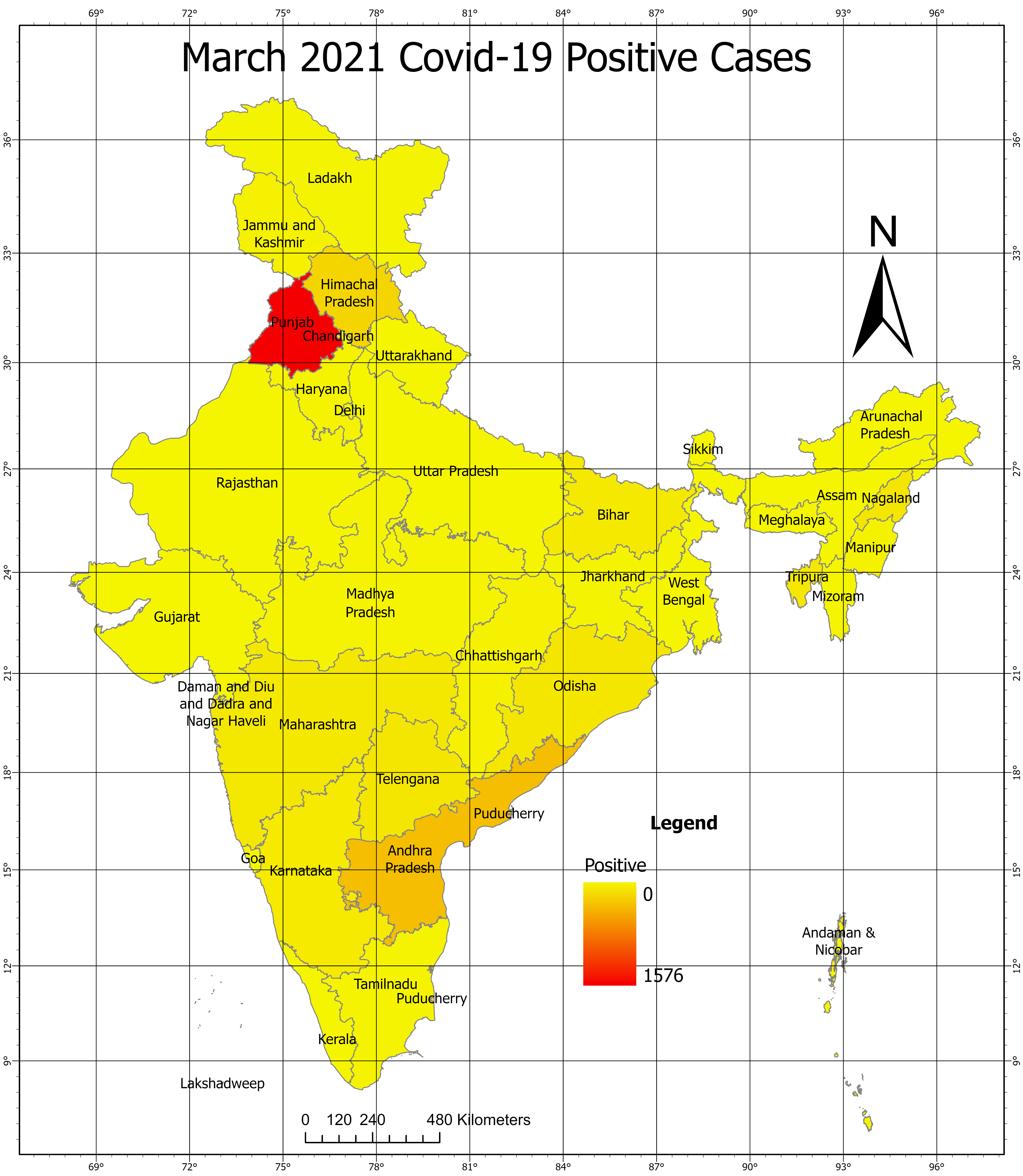

Supplement: Supplementary file 4 — Supplementary Information 4. [file 41598_2023_50933_MOESM4_ESM.zip › m_March 2021.png]

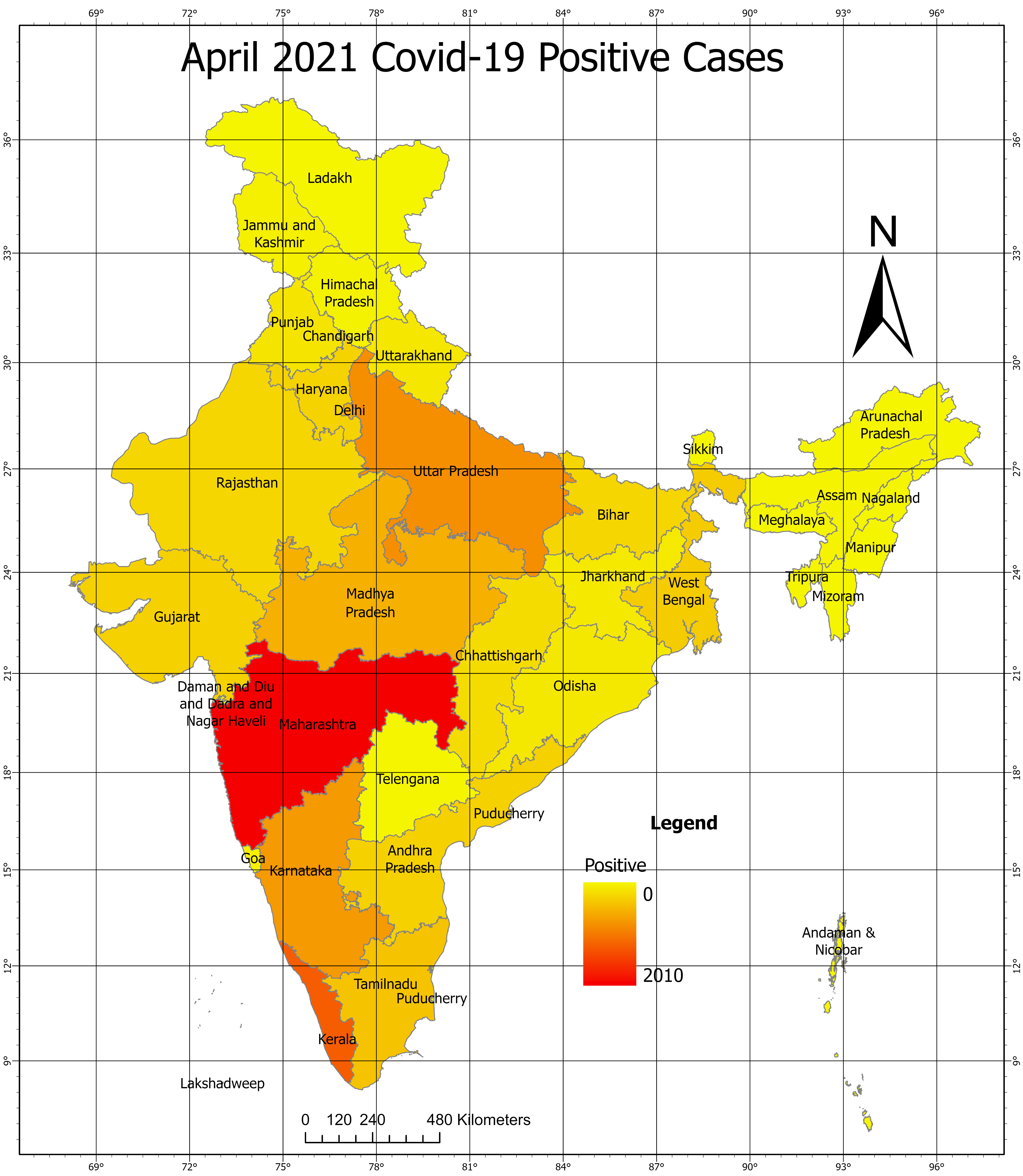

Supplement: Supplementary file 4 — Supplementary Information 4. [file 41598_2023_50933_MOESM4_ESM.zip › n_April 2021.png]

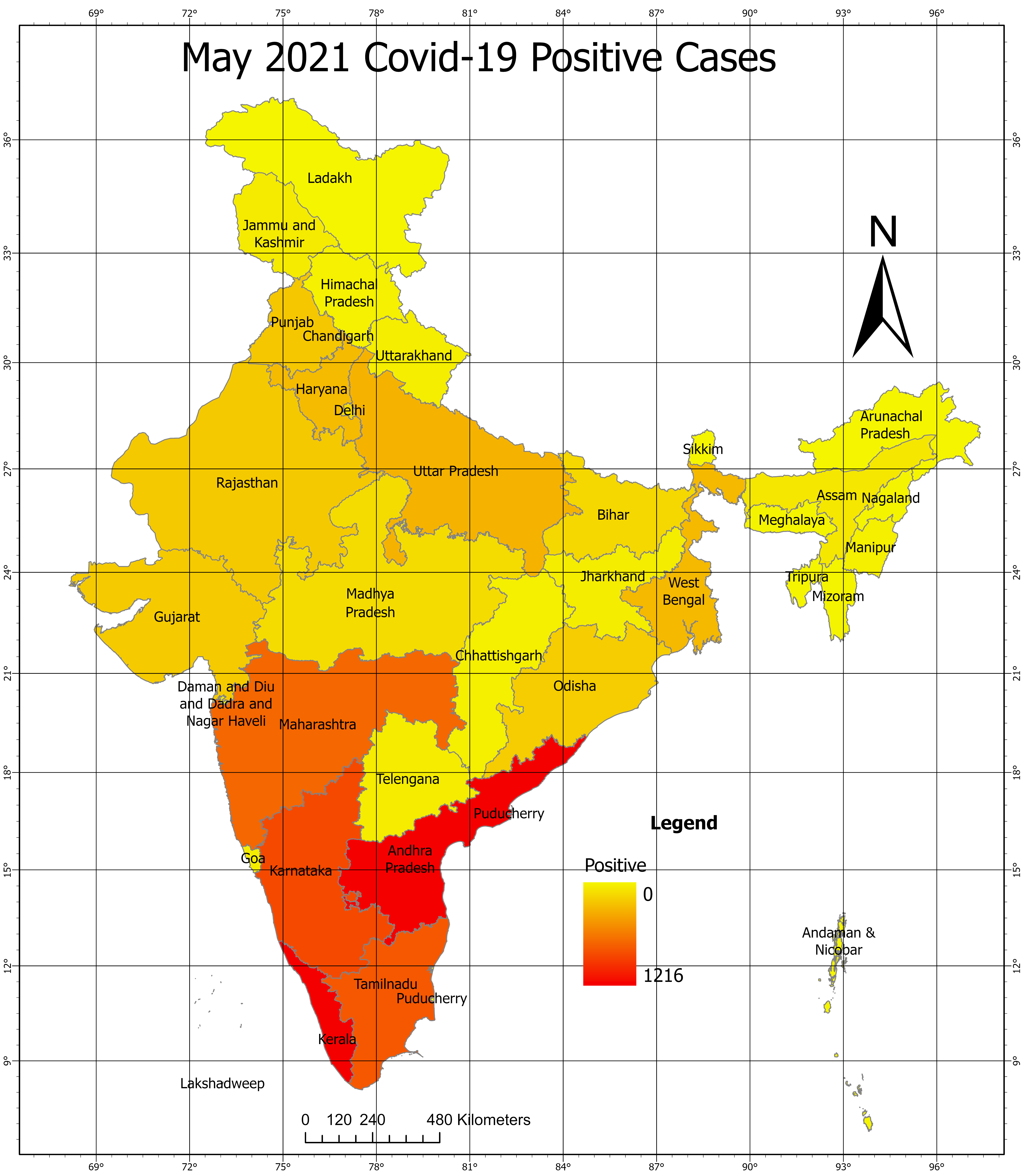

Supplement: Supplementary file 4 — Supplementary Information 4. [file 41598_2023_50933_MOESM4_ESM.zip › o_May 2021.png]

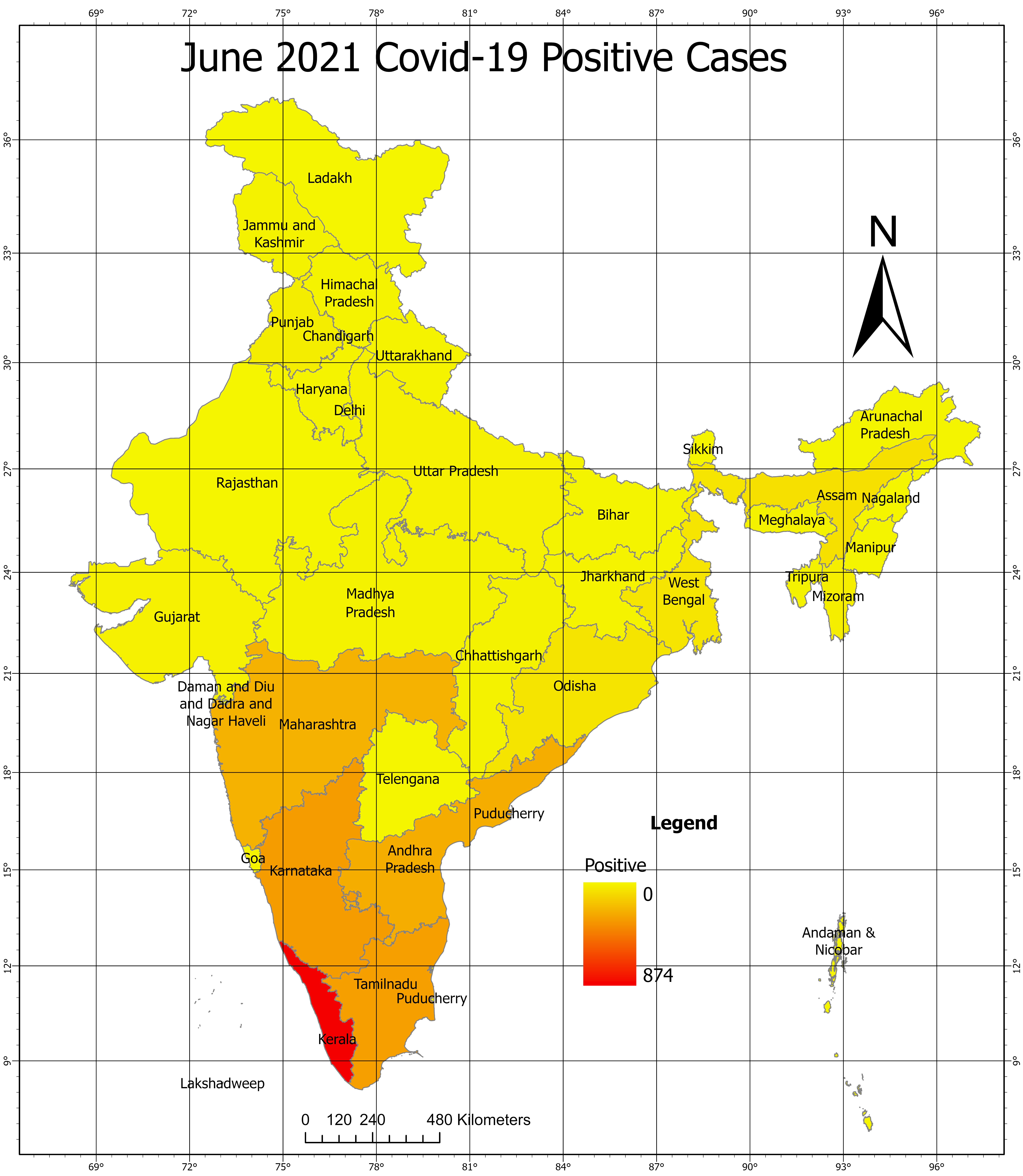

Supplement: Supplementary file 4 — Supplementary Information 4. [file 41598_2023_50933_MOESM4_ESM.zip › p_June 2021.png]

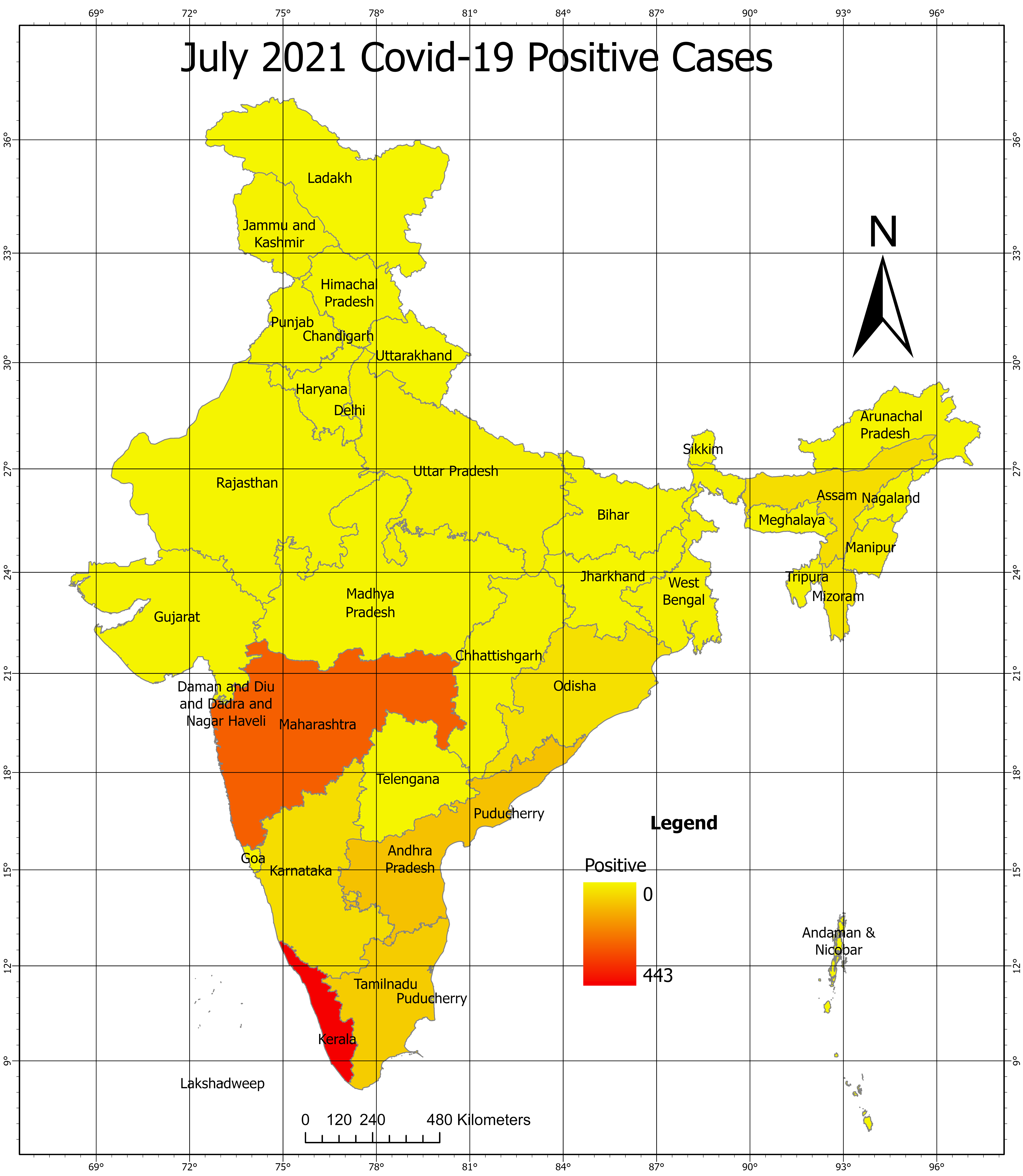

Supplement: Supplementary file 4 — Supplementary Information 4. [file 41598_2023_50933_MOESM4_ESM.zip › q_July 2021.png]

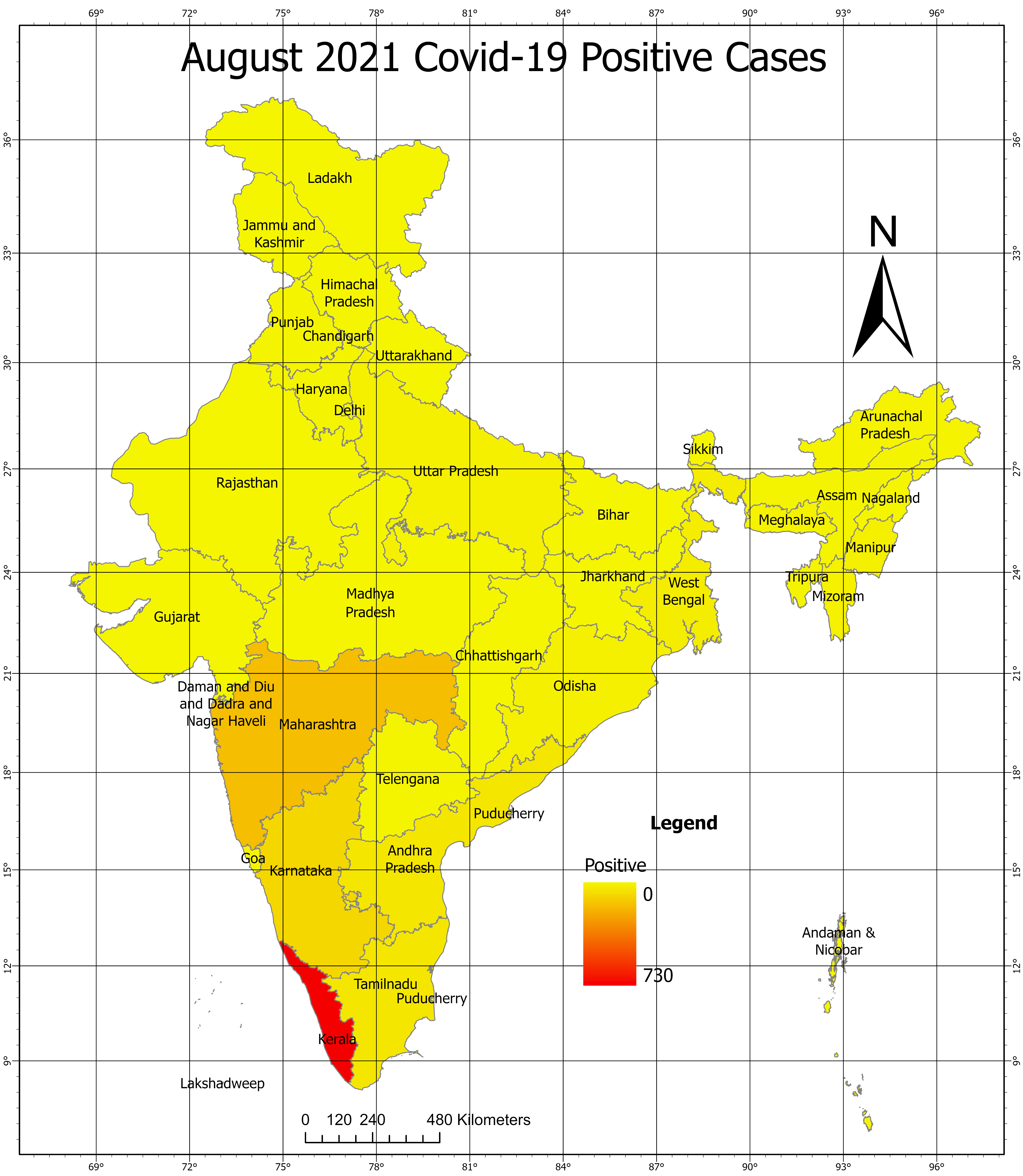

Supplement: Supplementary file 4 — Supplementary Information 4. [file 41598_2023_50933_MOESM4_ESM.zip › r_Aug 2021.png]

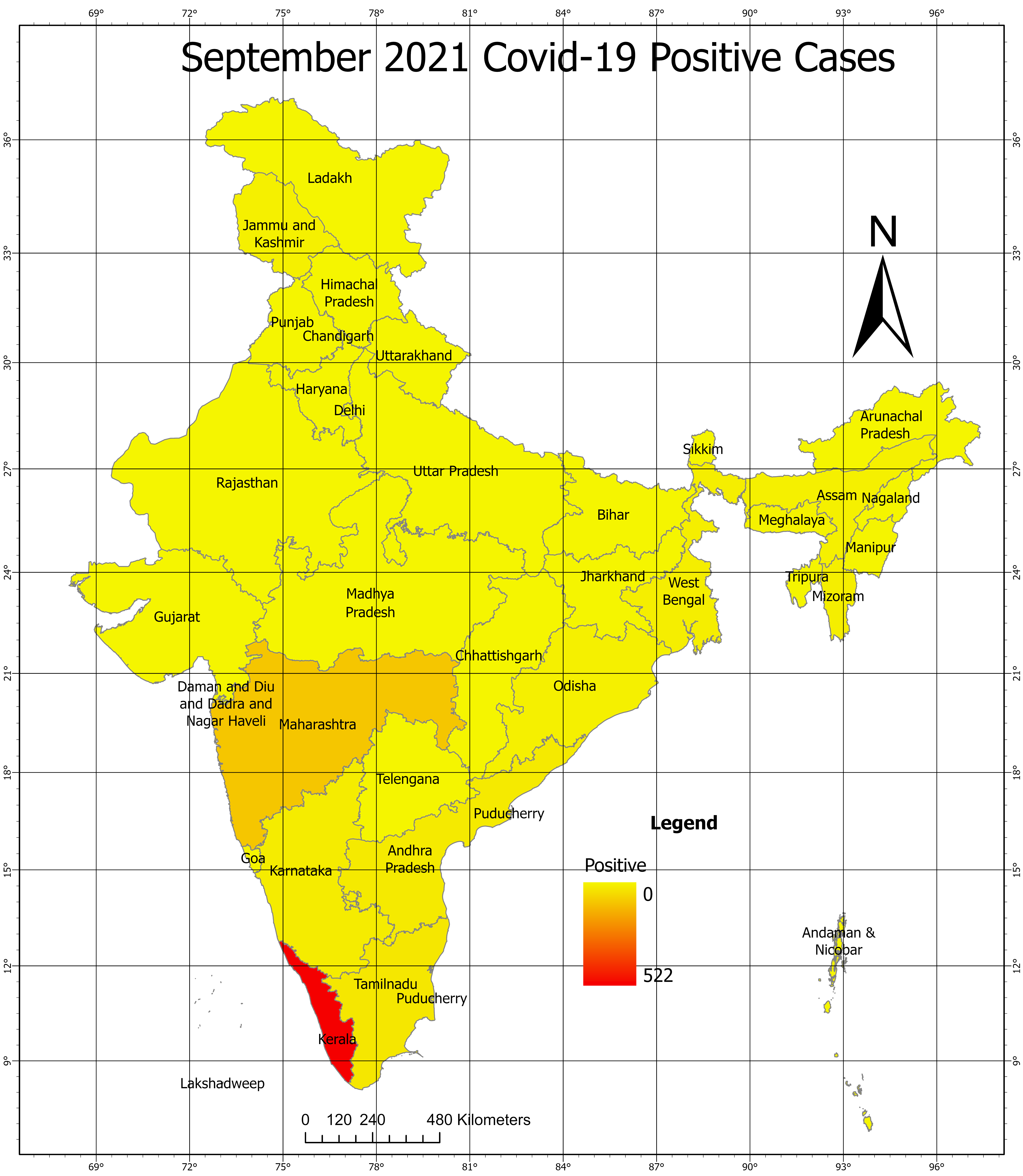

Supplement: Supplementary file 4 — Supplementary Information 4. [file 41598_2023_50933_MOESM4_ESM.zip › s_Sept 2021.png]

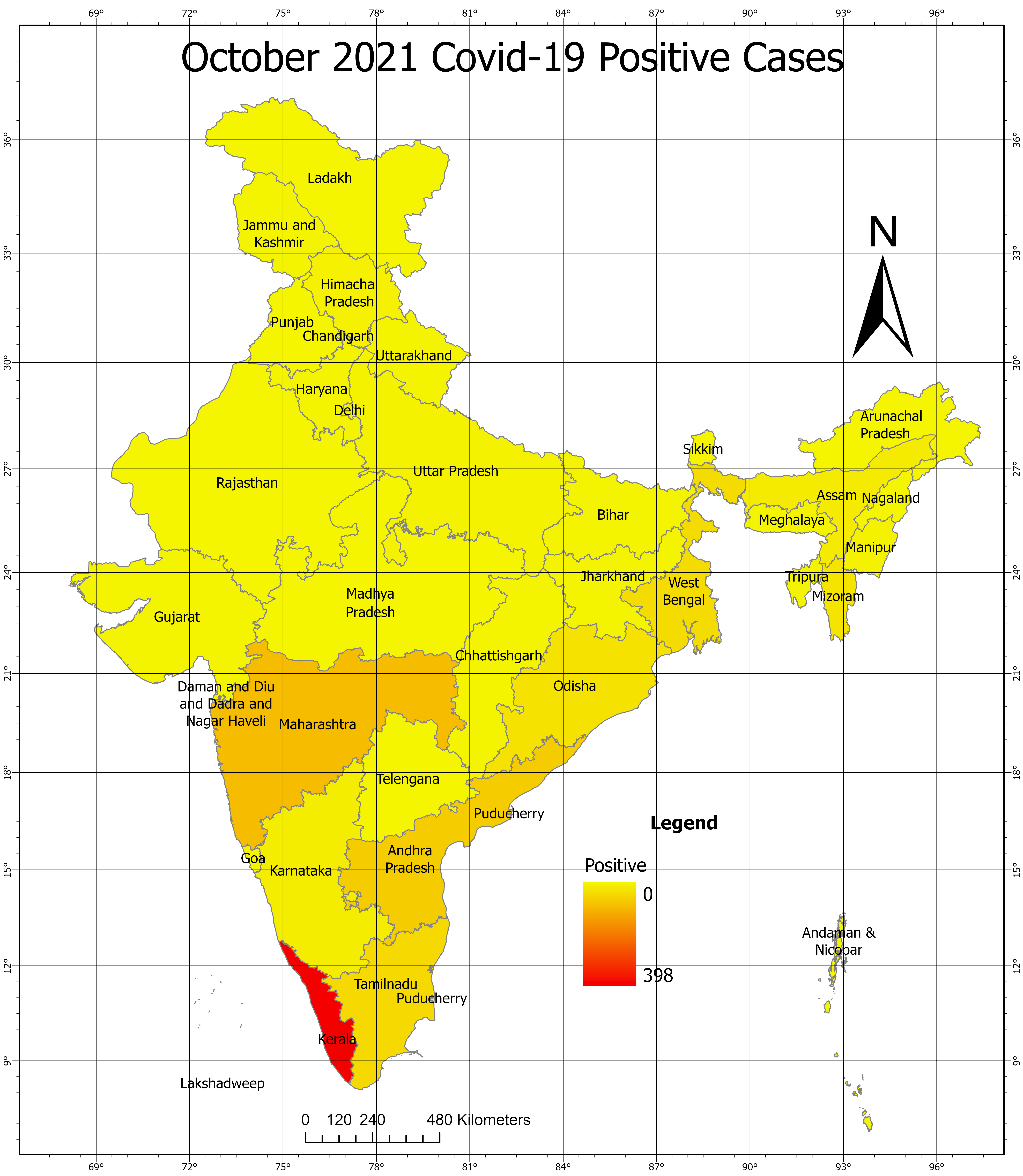

Supplement: Supplementary file 4 — Supplementary Information 4. [file 41598_2023_50933_MOESM4_ESM.zip › t_Oct 2021.png]

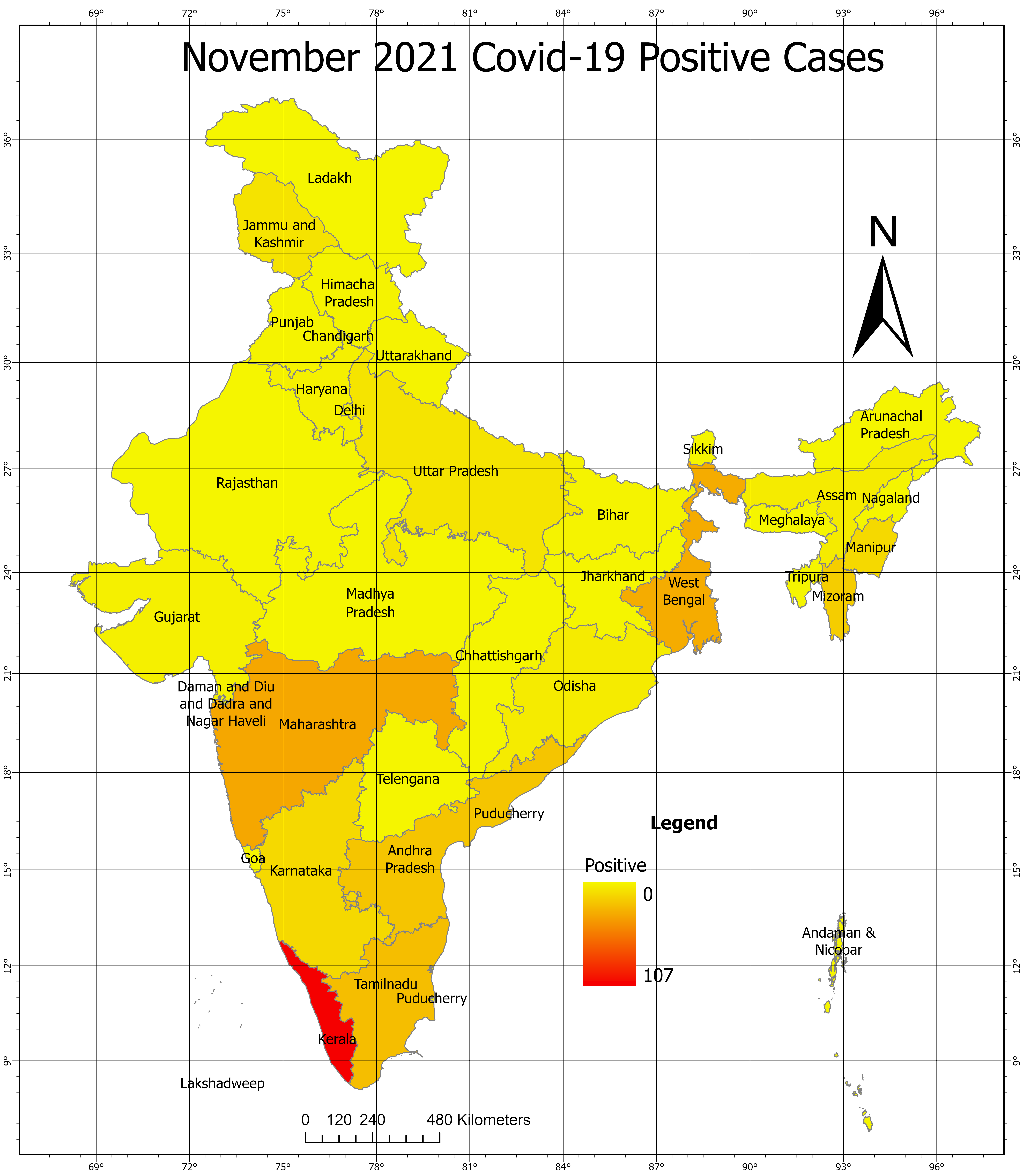

Supplement: Supplementary file 4 — Supplementary Information 4. [file 41598_2023_50933_MOESM4_ESM.zip › u_Nov 2021.png]

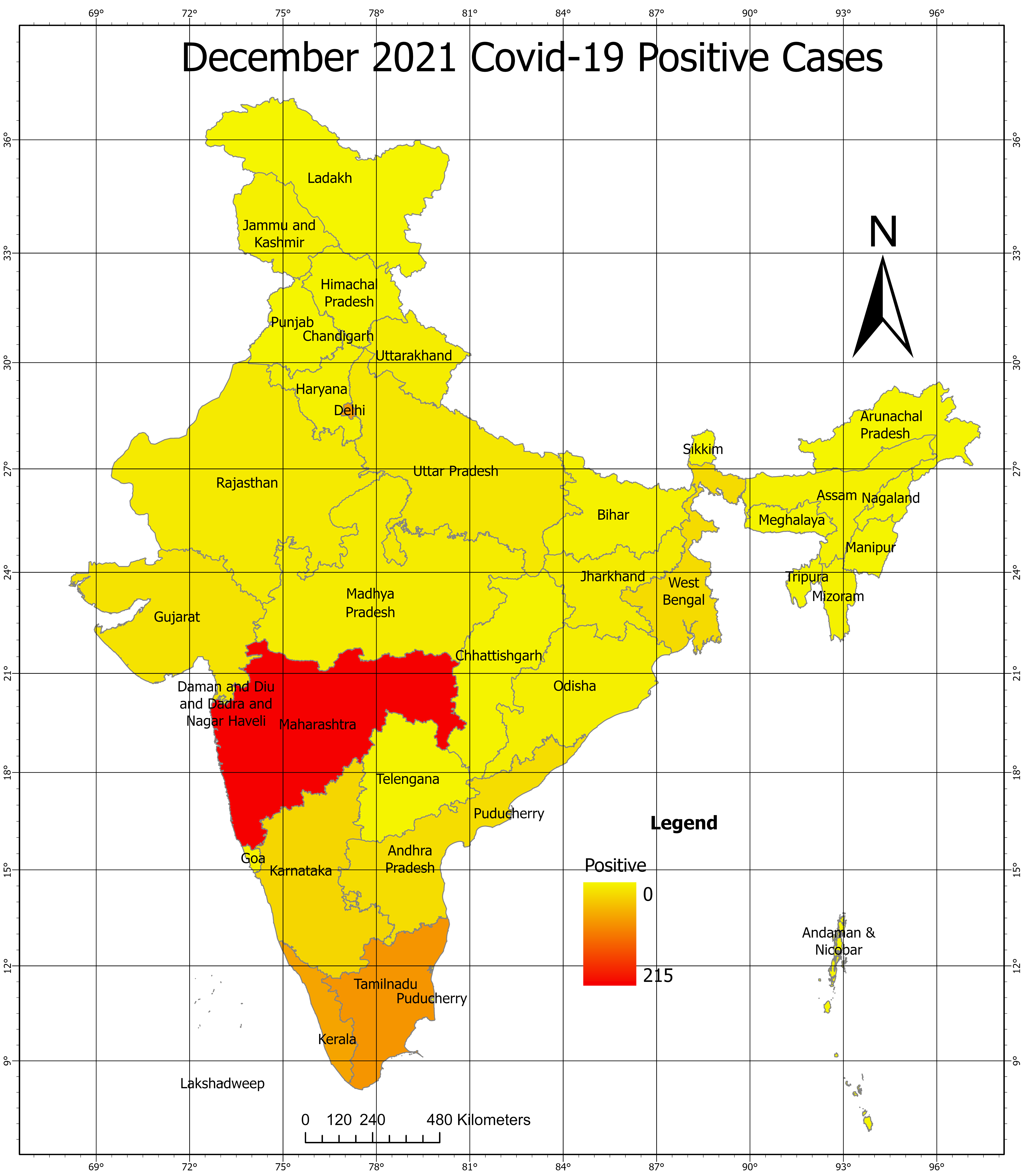

Supplement: Supplementary file 4 — Supplementary Information 4. [file 41598_2023_50933_MOESM4_ESM.zip › v_Dec 2021.png]

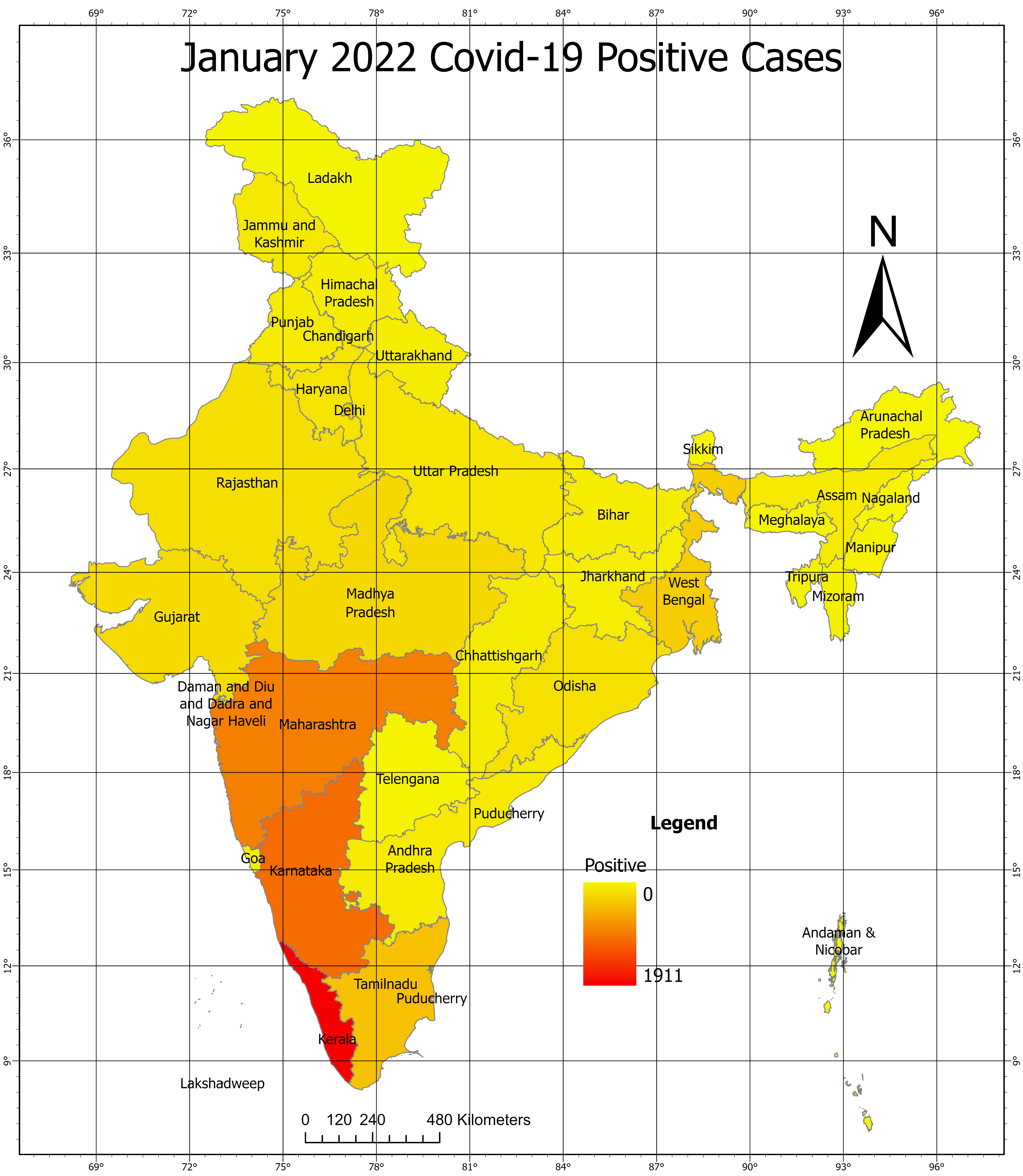

Supplement: Supplementary file 4 — Supplementary Information 4. [file 41598_2023_50933_MOESM4_ESM.zip › w_Jan 2022.png]

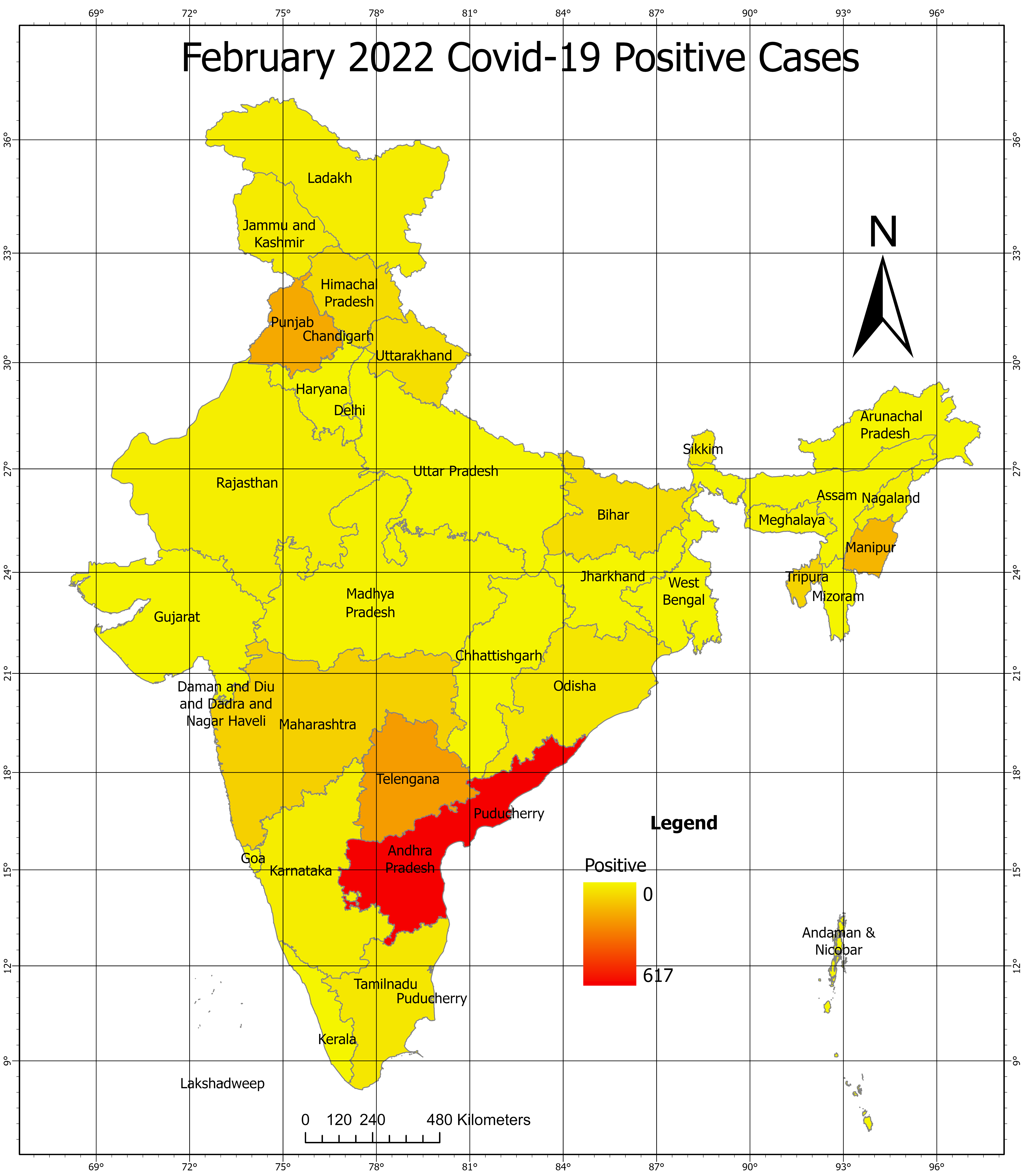

Supplement: Supplementary file 4 — Supplementary Information 4. [file 41598_2023_50933_MOESM4_ESM.zip › x_Feb 2022.png]

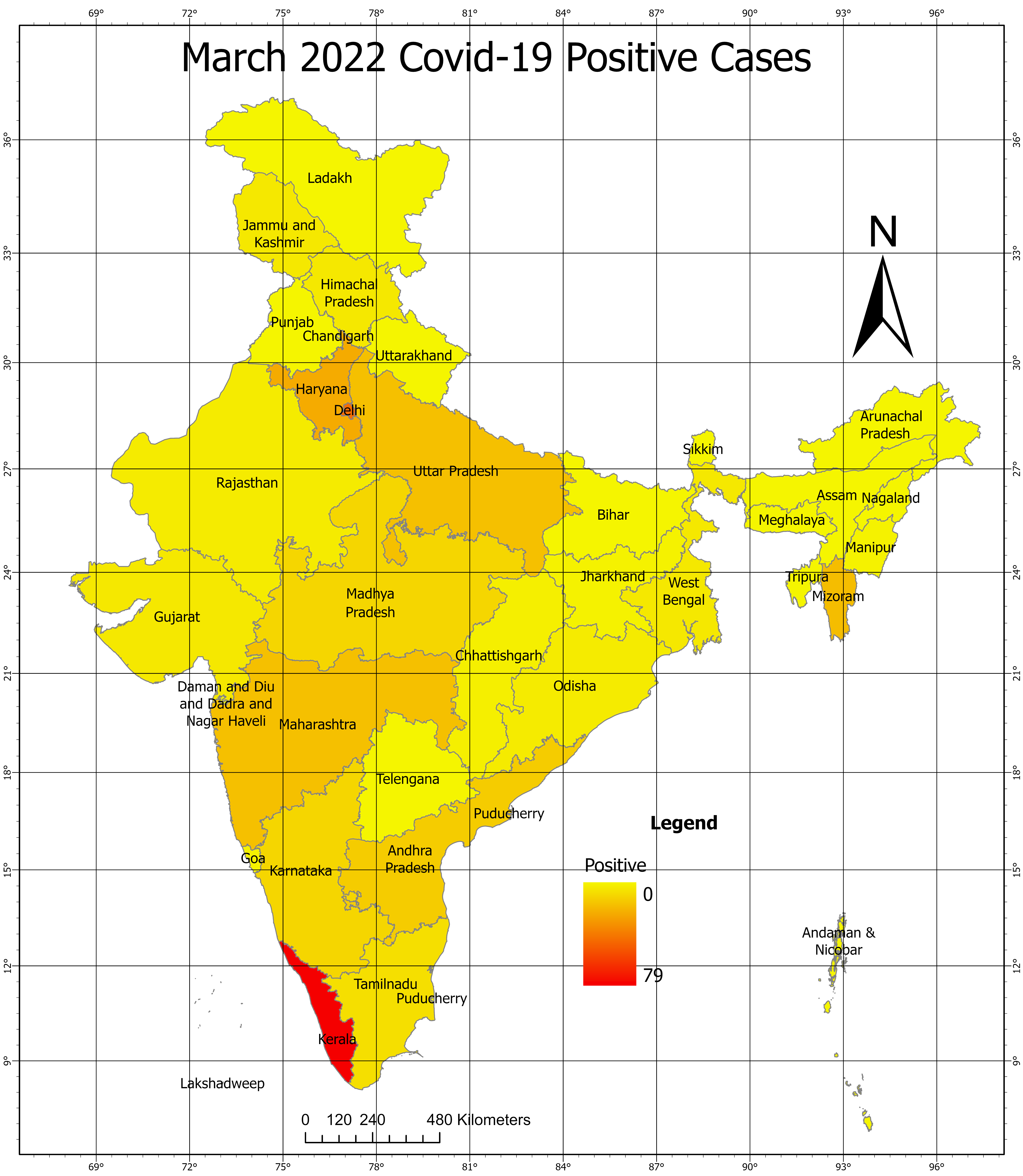

Supplement: Supplementary file 4 — Supplementary Information 4. [file 41598_2023_50933_MOESM4_ESM.zip › y_March 2022.png]

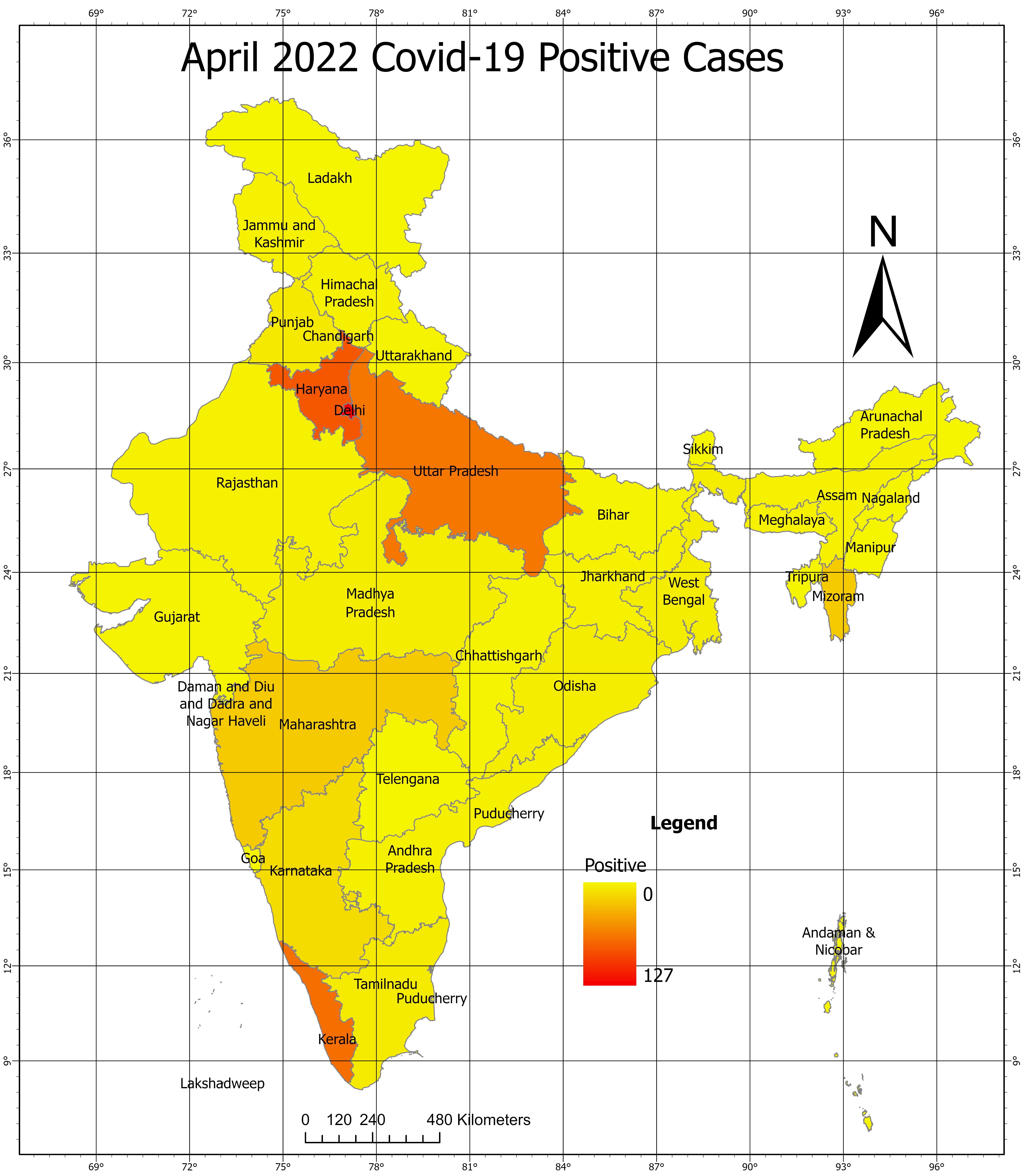

Supplement: Supplementary file 4 — Supplementary Information 4. [file 41598_2023_50933_MOESM4_ESM.zip › z_April 2022.png]

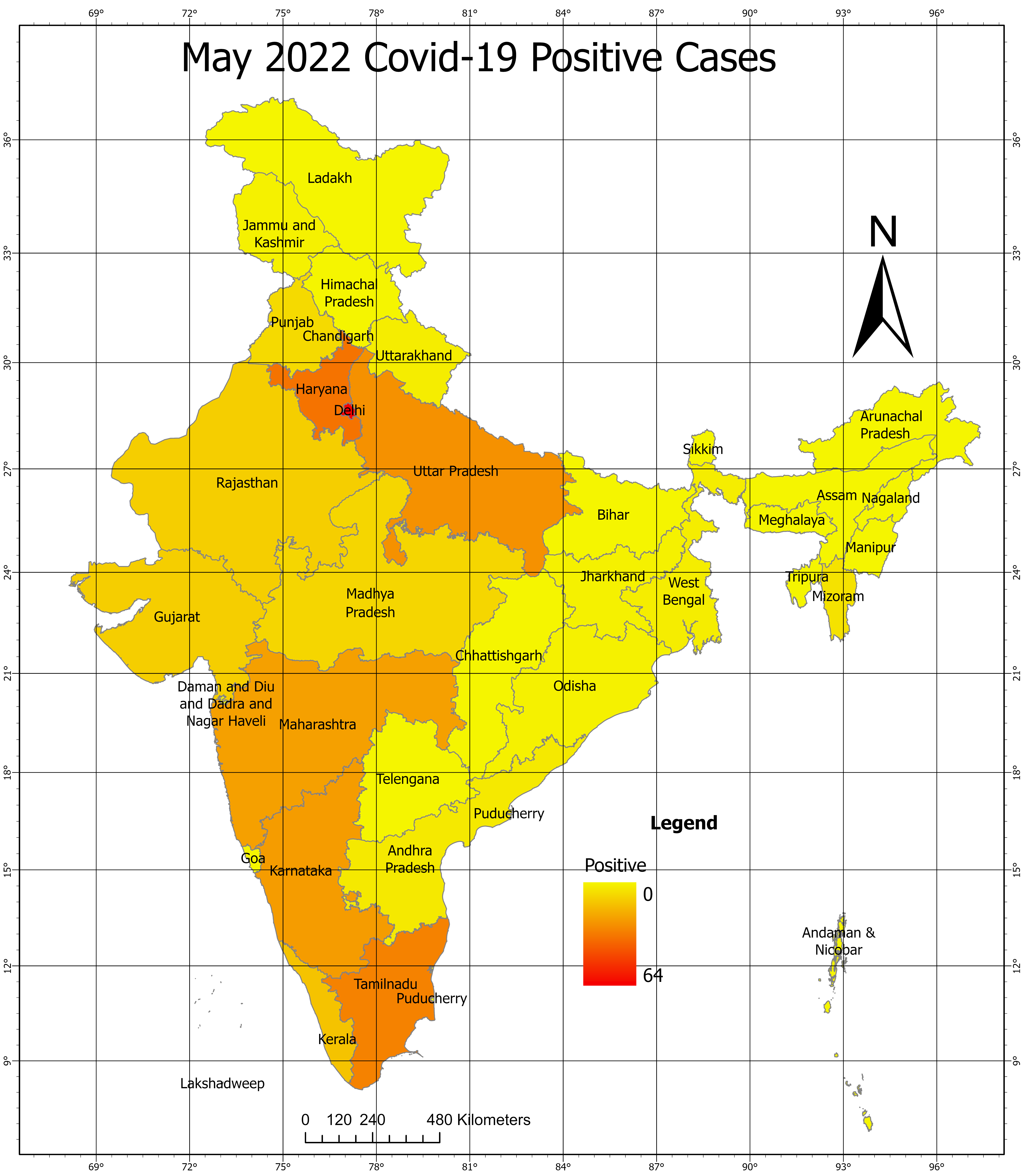

Supplement: Supplementary file 4 — Supplementary Information 4. [file 41598_2023_50933_MOESM4_ESM.zip › za_May 2022.png]

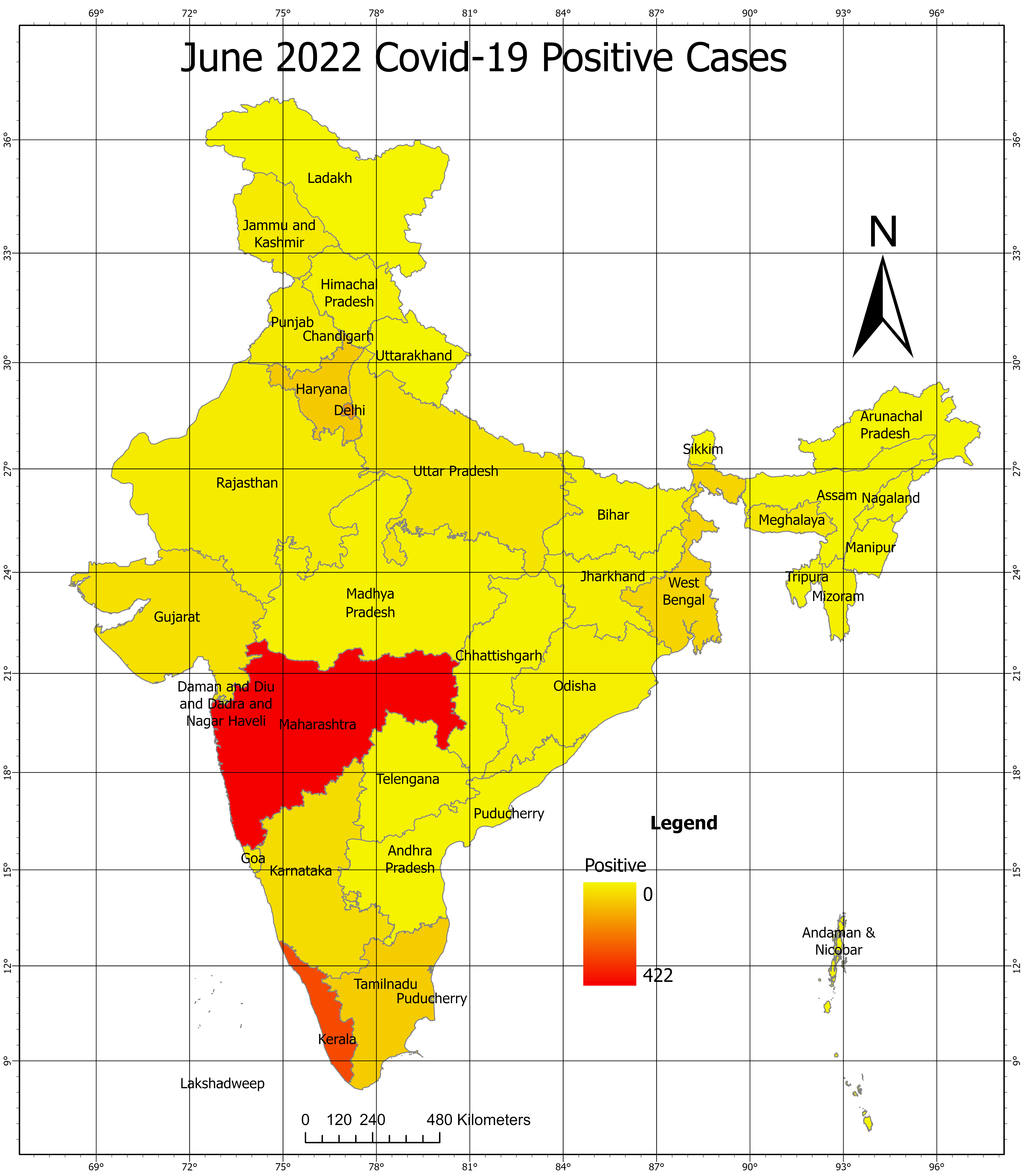

Supplement: Supplementary file 4 — Supplementary Information 4. [file 41598_2023_50933_MOESM4_ESM.zip › zb_June 2022.png]

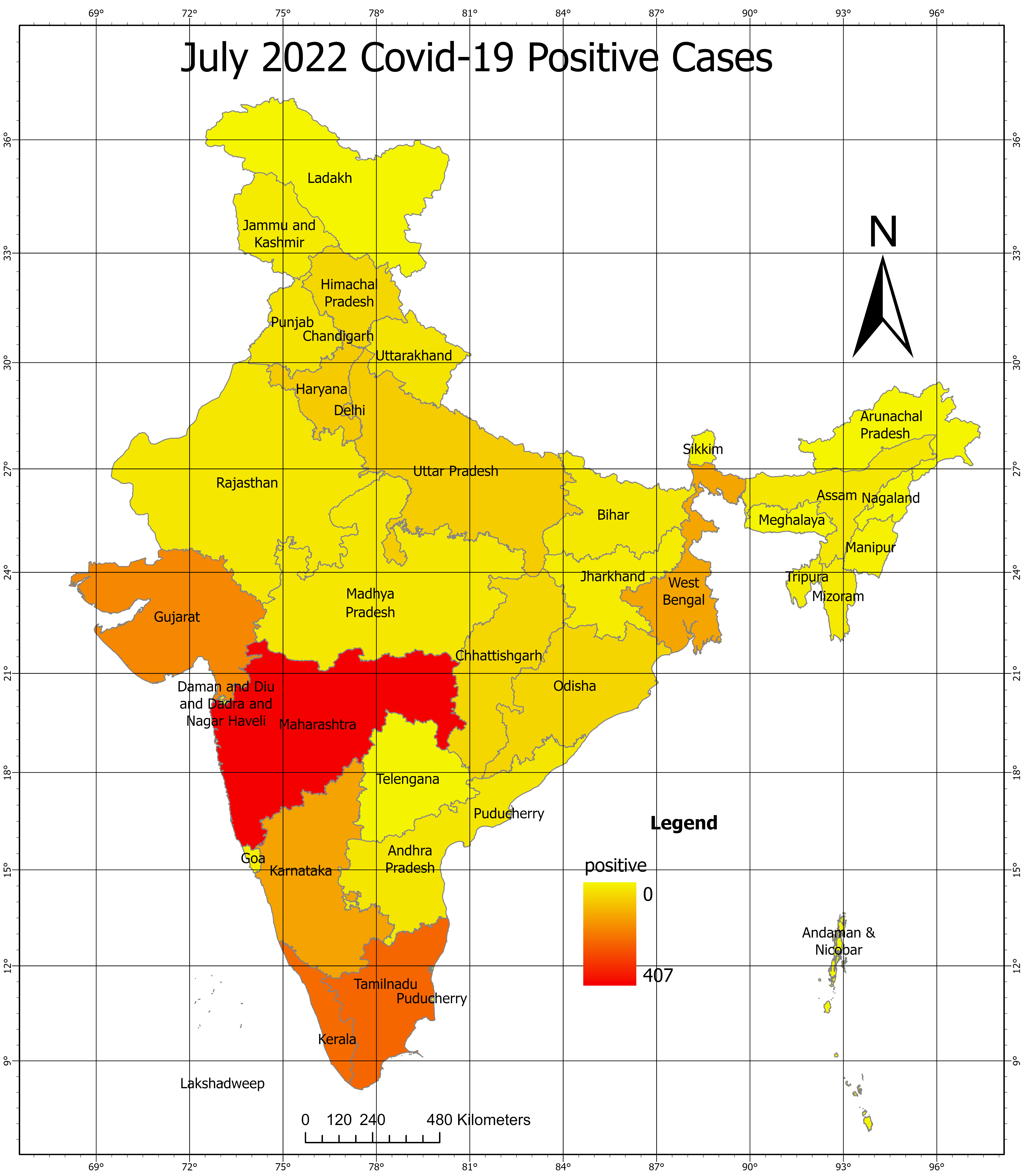

Supplement: Supplementary file 4 — Supplementary Information 4. [file 41598_2023_50933_MOESM4_ESM.zip › zc_July 2022.png]

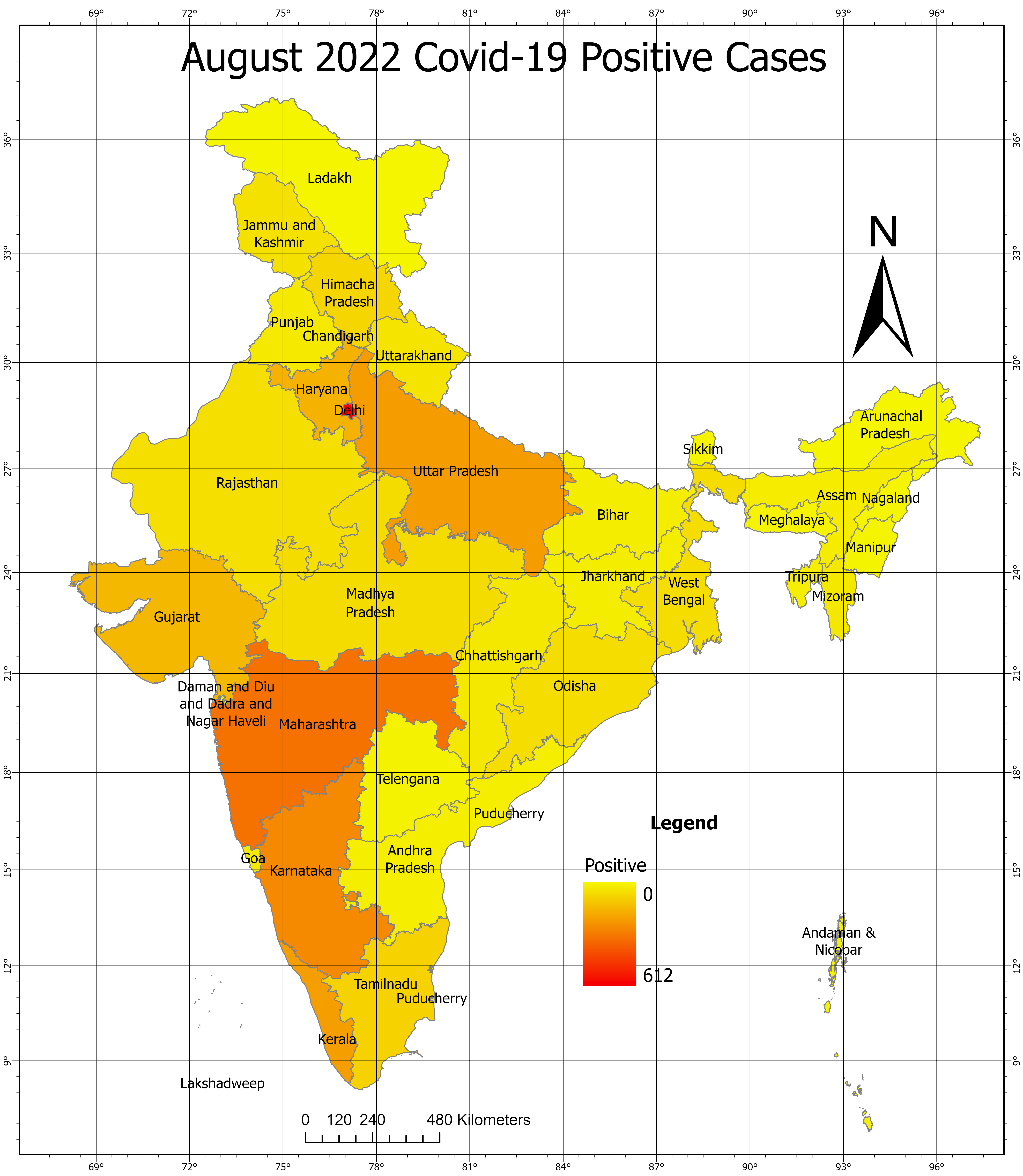

Supplement: Supplementary file 4 — Supplementary Information 4. [file 41598_2023_50933_MOESM4_ESM.zip › zd_Aug 2022.png]

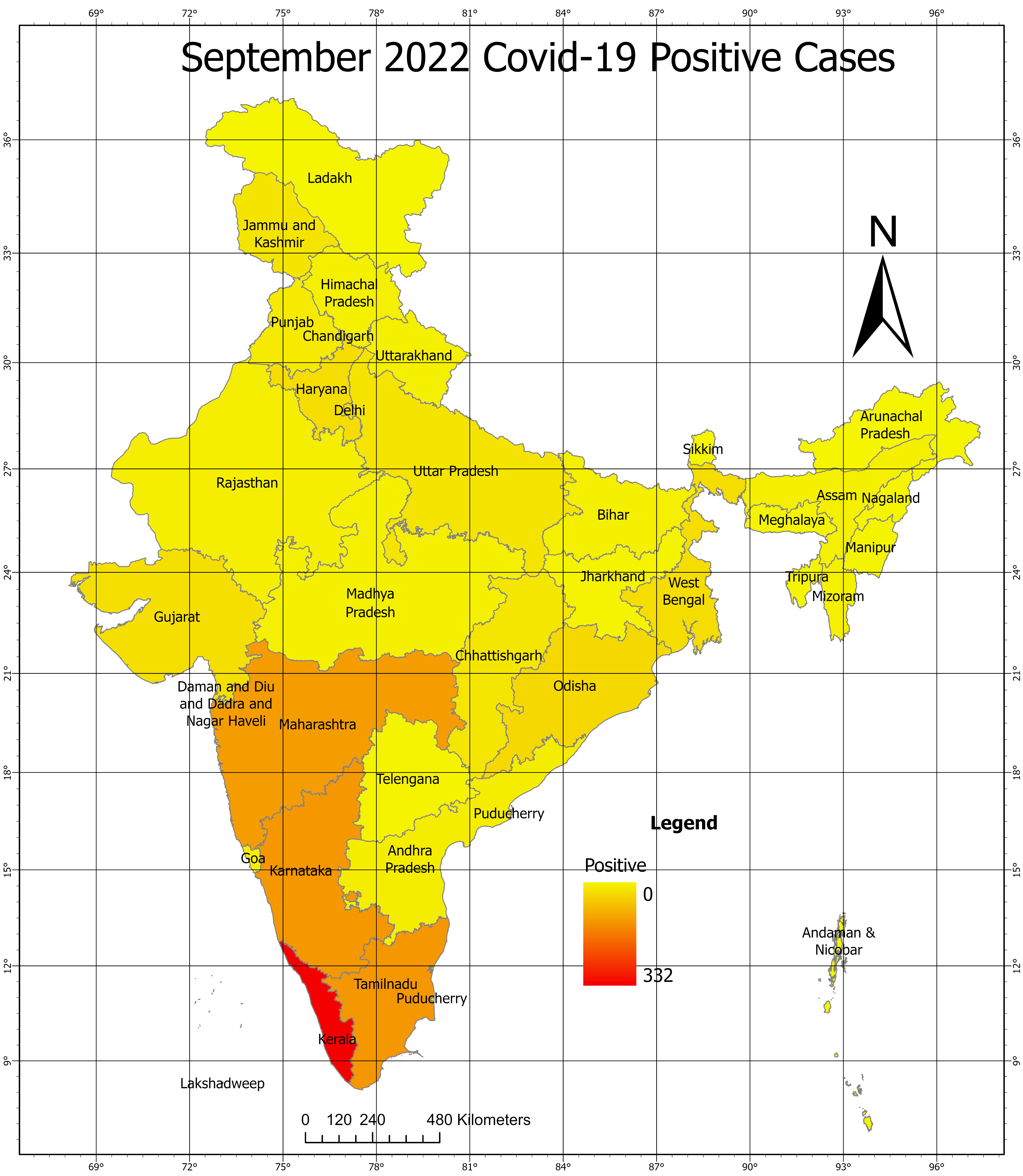

Supplement: Supplementary file 4 — Supplementary Information 4. [file 41598_2023_50933_MOESM4_ESM.zip › ze_Sept 2022.png]

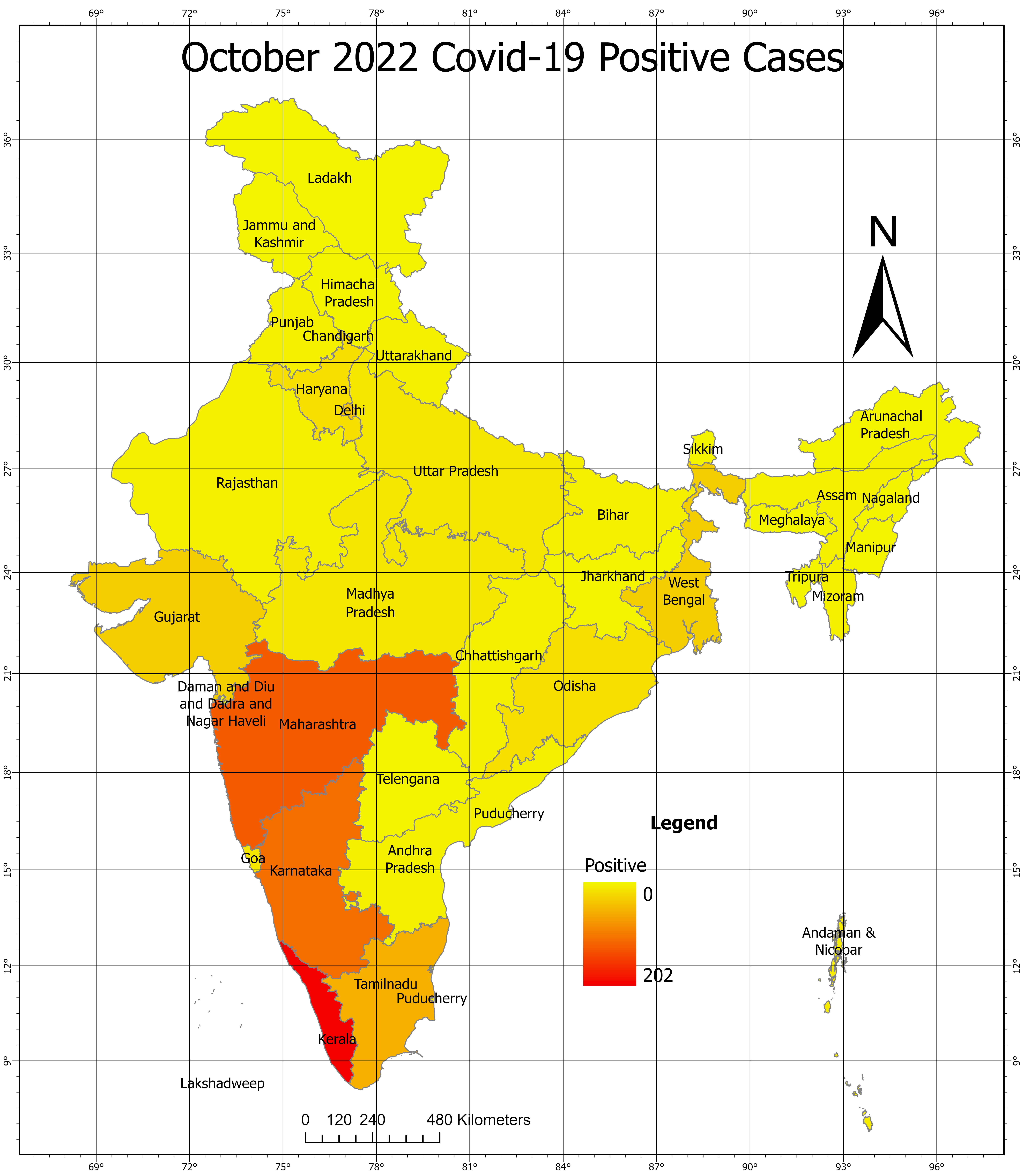

Supplement: Supplementary file 4 — Supplementary Information 4. [file 41598_2023_50933_MOESM4_ESM.zip › zf_Oct 2022.png]

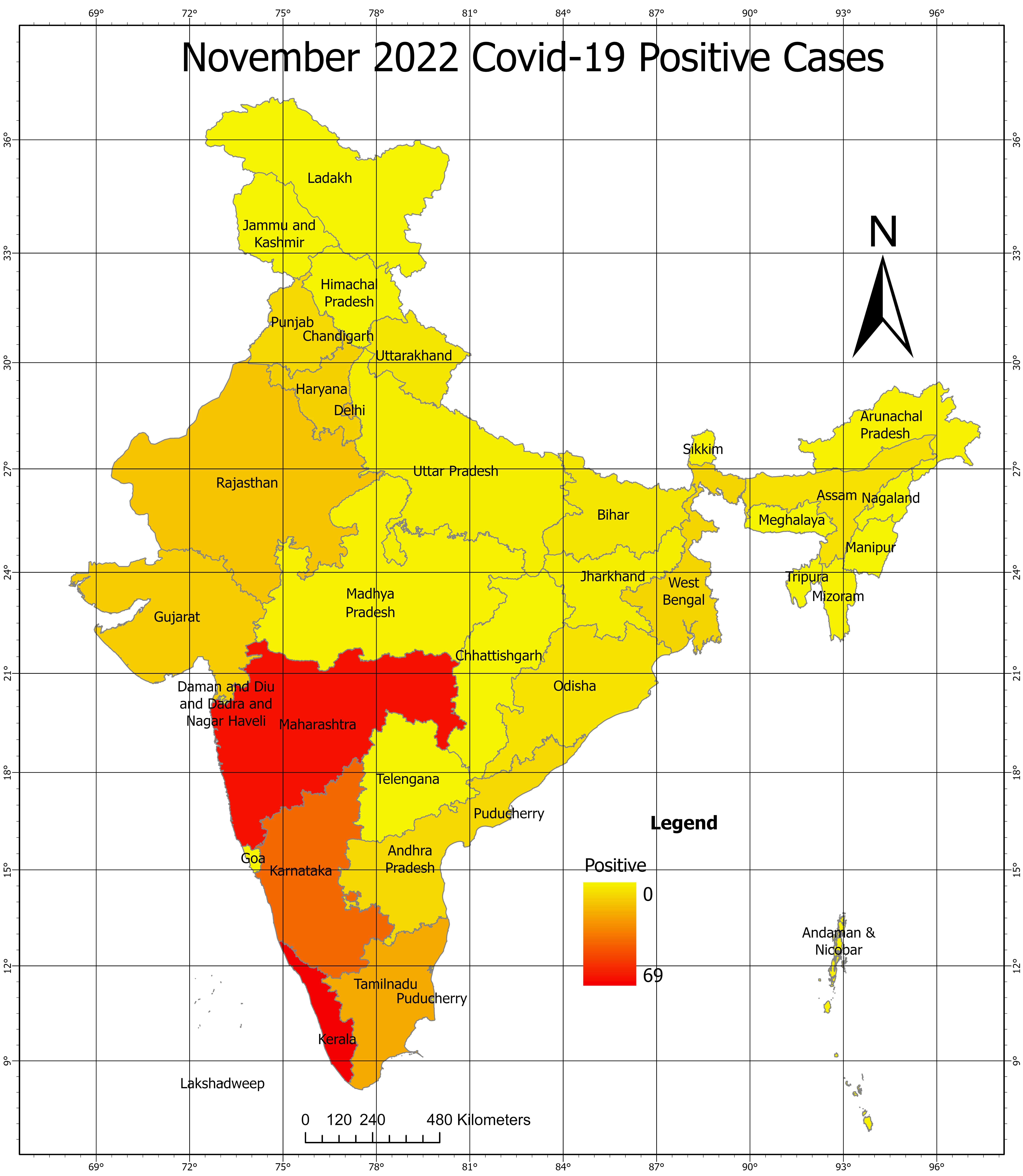

Supplement: Supplementary file 4 — Supplementary Information 4. [file 41598_2023_50933_MOESM4_ESM.zip › zg_Nov 2022.png]

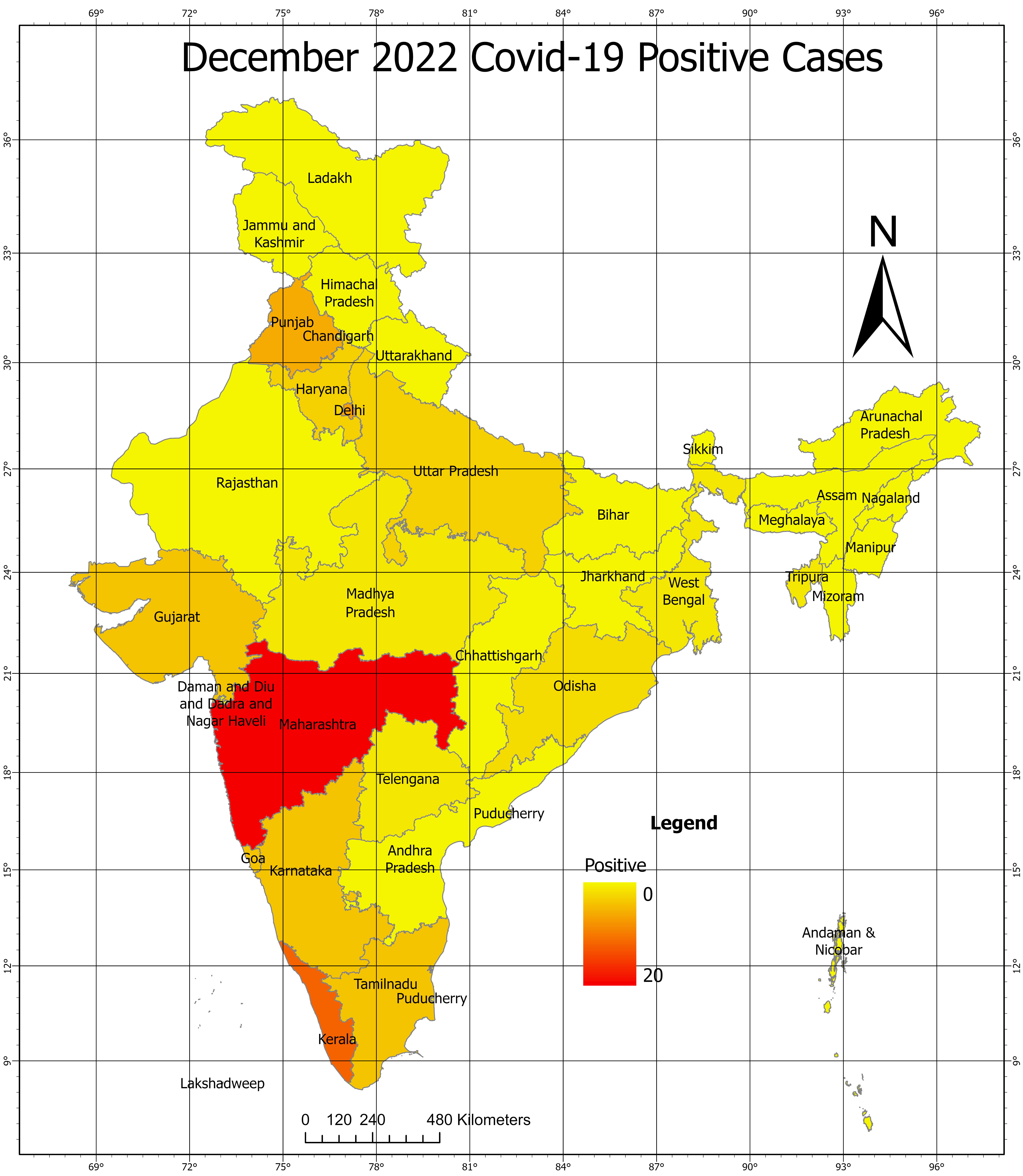

Supplement: Supplementary file 4 — Supplementary Information 4. [file 41598_2023_50933_MOESM4_ESM.zip › zh_Dec 2022.png]
